# Protomers of protein hetero-oligomers tend to resemble each other more than expected

**DOI:** 10.1186/2193-1801-3-680

**Published:** 2014-11-20

**Authors:** Oliviero Carugo

**Affiliations:** Department of Structural and Computational Biology, MFPL, Vienna University, Vienna, Austria; Department of Chemistry, University of Pavia, Pavia, Italy

**Keywords:** Hetero‒oligomers, Homo‒oligomers, Protein‒protein complex, Protein structure, Protomers

## Abstract

A large fraction of the proteome is made by proteins that are not permanently monomeric but form oligomeric assemblies, which can be either homo- or hetero-oligomeric. Here it is described that protomers of hetero-oligomeric proteins tend to resemble each other more than expected. This is verified by comparing the level of similarity of pairs of hetero-oligomeric protein protomers and of pairs of proteins that do not interact with each other. This observation, interesting per se, might reflect the evolution of hetero-oligomers from ancestral homo-oligomers, through gene duplication and paralogs divergence. However, other hypotheses cannot be excluded and the observed structural similarity might result from several causes.

**Electronic supplementary material:**

The online version of this article (doi:10.1186/2193-1801-3-680) contains supplementary material, which is available to authorized users.

## Background

Many proteins associate *in vivo* with other proteins and form supramolecular assemblies, which may contain (i) two or more copies of the same polypeptide chain (ii) two or more polypeptide chains that have different amino acid sequence, (iii) and even other types of biopolymers, like for example RNA.

The reason why proteins are not systematically monomeric is, in general, unknown. In some cases, it is obvious that different protomers of a protein-protein complex are responsible of different biochemical activities. For example, the TATA-binding protein (TBP), together with several transcription factors (TFs) and RNA polymerase II, can form the RNA polymerase II preinitiation complex (Lee and Young 2000): TBP is able to recognize the so-called TATA box, a DNA sequence segment that is found about 30 base pairs upstream of the transcription site in some eukaryotic gene promoters; due to its binding to DNA, TBP is recognized by the TFs and forms a hetero-oligomeric complex that is then recognized by RNA polymerase II; each protomer has then a different function in the final product (Lee and Young 2000). Another example is the [Fe, Ni]-hydrogenase: while the larger subunit of this hetero-dimeric enzyme hosts a bimetallic active site, with an atom of nickel (II/III) and an atom of iron (II), where the reaction 2H+H2 is catalyzed, the smaller subunit contains a series of FeS cluster that form a electron transport chain that brings the electrons towards the electron acceptor cytochrome c3 (Ogata et al. 2009).

In other cases the oligomerization is more puzzling. For example the Cu, Zn-superoxide dismutase is in general a homo-dimeric enzyme, where one active site is present in each of the two identical protomers (Bordo et al. 1994). However, there are also examples of monomeric Cu, Zn-superoxide dismutases that are perfectly functional and both monomeric and dimeric Cu, Zn-superoxide dismutases are expressed in different strains of E. coli (Bordo et al. 1999).

An extreme example of oligomerization is the ribosome, which contains tens of proteins and of RNA molecules both in prokaryotes and eukaryotes (Cech 2000).

Both homo-oligomeric proteins, where the various protomers have the same amino acid sequence like in the dimeric Cu, Zn-superoxide dismutase mentioned above, and hetero-oligomeric proteins, where different chains have different amino acid sequence like in the [Fe, Ni]-hydrogenase mentioned above, are observed in Nature. Moreover, further classifications are possible. For example, one can distinguish permanent complexes from transient complexes: while in the first case, the individual protomers are permanently bound to each other, like the alpha and beta chains of hemoglobin or a antigen-antibody complex, in the second, the protomers form a supramolecular complex only for a limited time period, like in the RNA polymerase II preinitiation complex mentioned above, depending on the experimental/physiological conditions. Another, possible partition between obligate and non-obligate complexes discriminates protein-protein complexes that are the only form in which the protomers can be found in Nature, like the four globins of hemoglobin, from protein-protein complexes, the protomers of which can be found either bound together or separated into individual monomers, like antibodies and antigens.

The importance of protein-protein interactions can hardly be underestimated. It is well accepted that life depends more on the “flexible” proteome than on the rigid “genome” and, in particular, on the interactions of the various biological molecules. It is not surprising that much attention has been devoted, in recent years, to the study and the analysis of protein-protein interaction patterns and networks (Ideker and Krogan 2012). Parallel, several databases have been created to archive any type of information about inter-molecular interactions of biological interest (Orchard 2012) and some of them have been specifically focused on the three-dimensional structures of the biological oligomeric complexes (Levy et al. 2006). Some effort was also devoted to the sequence-based methods of prediction of protein quaternary structures (Carugo 2007b).

Few years ago, it was observed that the two protein molecules that form hetero-dimers tend to have structures quite similar even in the case when their amino acid sequences are very different (Lukatsky et al. 2007). This was interpreted in a theoretical framework where similar structure has larger likelihood to interact (Lukatsky et al. 2006) and this allowed to envisage that many modern protein complexes could have evolved from earlier homo-dimers, through sequence divergence of paralogous genes (Lukatsky et al. 2007). Gene duplication and paralogs evolution was documented previously in archeal chaperonins (Archibald et al. 1999), and, more in general, it was proposed that most interactions between paralogs have been inherited from earlier homodimers and not established after duplication (Ispolatov et al. 2005) and that duplication of homomeric interactions, which results in the formation of paralogous complexes, is a common mechanism for the evolution of complexes (Pereira-Leal et al. 2007).

In the present article, I extend these studies and I analyze the levels of structural similarity between individual protomers in a controlled set of hetero-oligomeric proteins. The main finding is that if protomers A and B interact to form a complex AB, the similarity between the structures of A and B is greater than expected for dimeric, trimeric and tetrameric hetero-oligomers. The expected value is empirically estimated by comparing several pairs of structures of proteins that do not interact one with each other. The structural similarity is estimated (i) by means of the distance on the Proteomic Ramachandran plot (PRplot) (Carugo and Djinović-Carugo 2013), (ii) by superposing the pairs of structures with the software Sheba (Jung and Lee 2000), (iii) by mapping the structural data on the CATH classification of protein structural domains (Sillitoe et al. 2013); and (iv) by mapping the structural data on the SCOP classification of protein structural domains (Andreeva et al. 2008).

## Results

### Distances on the Proteomic Ramachandran plot (PRplot)

The Proteomic Ramachandran Plot (PRplot) is a generalization of the Ramachandran plot, where a protein structure is represented by the average values of the main-chain phi and psi torsion angles (Carugo and Djinović-Carugo 2013). While the Ramachandran plot is used to map, on the phi/psi plane, each amino acid of a single protein, the PRplot is used to map on the phi/psi plane several proteins represented by their average phi and psi angles. It has been observed that protein structures are aligned along a sigmoid curve that goes approximately from phi = -100° and psi =130° to phi = -75° and psi = -50°.

Obviously, two identical structures occupy the same point on the PRplot and two different structures occupy two different points in the PRplot. The distance between two points of the PRplot is therefore a measure of the structural diversity between two structures. It must be observed that two structures with identical secondary structure composition can occupy two points, one close to the other, in the PRplot, even if they have a different fold. However, in my experience, this is a very rare occurrence and, to a first approximation, the distance between two points of the PRplot is a valid approximation of the structural difference between pairs of protein structures (Carugo and Djinović-Carugo 2013). The major advantage of this approach is its computational speed, since the comparison between two protein structures is reduced to the computation of a Euclidean distance in a bi-dimensional plane.

Table 1 shows the average distances between protomers in homo-oligomeric complexes, in hetero-oligomeric complexes, and in a series of datasets of proteins that do not interact one with each other (see Methods for the description of the ensembles of proteins that do not interact with each other). As expected, the dpp values are very close to 0° in homo-oligomers, where the protomers have identical amino acid sequences and hence very similar three-dimensional structures. On average, these dpp values (about 2°) are similar to those observed by comparing alternative models of well ordered, globular protein structures obtained in solution by NMR spectroscopy (Carugo and Djinović-Carugo 2013). On the contrary, the average dpp values are much larger in hetero-oligomers, where the protomers have different amino acid sequences and where, as a consequence, the protomers can be structurally different. Their values range from 44° to 61° and are therefore much smaller than the maximal possible dpp value (254°) and also than the value of 85° that was observed to be the natural separation between different clusters of globular proteins in the PRplot (Carugo and Djinović-Carugo 2013).

**Table 1:** **dpp average values (standard errors in parentheses) in various datasets of protein structures**

Dataset	Dpp (°)
Homo-dimers	2.7(0.1)
Homo-trimers	2.0(0.2)
Homo-tetramers	2.2(0.1)
Hetero-dimers	57.0(3.1)
Hetero-trimers	61.4(4.7)
Hetero-tetramers	43.9(3.4)
Random set 1	72.1(1.9)
Random set 2	74.5(1.9)
Random set 3	72.3(1.9)
Random set 4	74.1(1.9)
Random set 5	73.9(1.9)
Random set 6	76.3(1.9)
Monomer set 1	77.7(1.8)
Monomer set 2	77.2(1.8)
Monomer set 3	81.0(1.9)
Monomer set 4	82.3(1.8)
Monomer set 5	73.5(1.7)
Monomer set 6	64.2(1.6)

It is necessary to analyze the dpp value for proteins different from the protomers of hetero-oligomers in order to give a statistical meaning to the dpp values observed by comparing the protomers of hetero-oligomers. For this reason, a series of sets of pairs of proteins that do not interact have been constructed. Obviously, it is difficult to select proteins that have no chance to be protomers of hetero-oligomers, since no experimental evidence of this property can be found in literature. For this reason, I followed two alternative strategies (see Methods for details). On the one hand, I assumed that a polypeptide chain found in a hetero-oligomeric complex does not interact with a protein chain found in a homo-oligomeric complex. In the second hand, I assumed that a monomeric protein does not interact with another monomeric protein. Therefore, I constructed six sets of 1,000 pairs of random homo-hetero protomers (Random set 1–6) and six sets of 1,000 pairs of monomeric proteins (Monomer set 1–6) (see Additional file 1).

Both of these strategies have weak points. For example, it is possible that two monomeric proteins can form a more or less stable functional complex under certain physiological conditions. However, it is reasonable to suppose that these control datasets are extremely enriched in pairs of non-interacting proteins, with relatively few spurious entries. It is also important to observe that the main consequence of the presence of some erroneous entries in these control datasets is the introduction of a noise that might cause an underestimation of the differences between pairs of protomers of hetero-oligomers and pairs of non-interacting proteins.

Table 1 shows that the average structural divergence of the protomers of hetero-oligomers is smaller than the average structural divergence observed when pairs of non-interacting proteins are compared. While the average dpp values between pair of protomers in hetero-oligomers range from 44° to 61°, they range from 64° to 82° between pairs of non-interacting proteins. This suggests that protomers of hetero-oligomers tend to resemble each other more than proteins that do not interact. In other words, they resemble each other more than expected.

### Superpositions

Amongst the numerous procedures that have been developed and used to compare protein three-dimensional structures, the superposition, in general limited to equivalenced Calpha carbon atom pairs, is certainly the most common (Carugo 2006; Carugo 2007a; Carugo and Pongor 2002). Typically, the quality of a superposition between two sets of Calpha carbon atoms is evaluated with the root-mean-square-distance (rmsd) between equivalenced atom pairs. However, this is not practical, since the rmsd value depends on the dimension of the proteins that are compared (Carugo and Pongor 2001) and it occurs frequently to compare proteins of different dimension. As a consequence, alternative figures of merit must be used. Here, I use the m-scores, which are defined as the ratio between the number of equivalenced Calpha carbon atom pairs and the maximal number of possible equivalences between the two protein structures that are superposed (see Methods for details). The m-scores have a major advantage over the rmsds: they have both a upper and a lower limit, equal to 100.0 and 0.0, respectively, while the rmsd values have a lower limit of 0.0 but lack an upper limit. Obviously, m-score = 100.0 if the two protein structures that are compared are identical and m-score = 0.0 if they cannot be superposed at all.

Here the m-scores were computed with the computer program Sheba. Their average values for various types of proteins are shown in Table 2. As expected, the m-scores values are very close to 100.0 for the homo-oligomeric complexes. Analogously, it is expected that they are much smaller when the two structures that are compared are completely unrelated. The m-score values are close to 19–20 when the two structures belong to monomeric proteins or when to a hetero-oligomeric complex and to a homo-oligomeric complex.

**Table 2:** **Average m-scores values (standard errors in parentheses) in various datasets**

Dataset	m-score
Homo-dimers	98.8 (0.1)
Homo-trimers	99.3 (0.1)
Homo-tetramers	99.0 (0.1)
Hetero-dimers	35.7 (1.4)
Hetero-trimers	34.1 (1.5)
Hetero-tetramers	40.6 (2.0)
Random set 1	19.4 (0.4)
Random set 2	20.1 (0.5)
Random set 3	19.6 (0.5)
Random set 4	20.6 (0.5)
Random set 5	19.7 (0.5)
Random set 6	19.0 (0.4)
Monomer set 1	19.5 (0.4)
Monomer set 2	19.2 (0.4)
Monomer set 3	20.0 (0.4)
Monomer set 4	19.3 (0.4)
Monomer set 5	19.4 (0.4)
Monomer set 6	19.0 (0.4)

The m-scores have intermediate values (close to 35–40) when the two structures that are compared are protomers of the same hetero-oligomeric complex. This clearly indicates that, if two proteins are able to form a stable complex, their structures are more similar than those of two proteins that do not interact to form a stable complex. In agreement with what is described in the previous chapter about the analysis of the dpp values, it can be concluded that two proteins that interact in a hetero-complex tend to more similar to each other than expected.

### Comparisons based on CATH

The CATH database has been established about fifteen years ago as a collection and a classification of protein structural domains (Orengo et al. 1997) and it has been constantly updated (Sillitoe et al. 2013). It is thus not only a mere list of domains but also an elaborated hierarchical classification. The first level of this hierarchy is the “class” and two protein domains are grouped into the same class cluster if they have a similar secondary structure composition, for example essentially alpha, essentially beta, mixed, etc. The second level of the classification is the “architecture” and two protein domains share the same architecture if they have the same secondary structure elements (helices and strands) and if these secondary structure elements have the same reciprocal orientation. The third level of the hierarchy is the “topology” and two protein domains are grouped in the same topology cluster not only if they share the same class and architecture, but also if the connections between their secondary structure elements is similar along the protein sequence. This topology level of classification is the concept that usually is named fold in structural biology: two protein domains have the same fold if they have the same class, the same architecture, and the same topology. Further classification levels that involve evolutionary information and sequence similarity are not considered here.

I limit the attention to the analysis of the types of fold, defined by the topology level of the hierarchical classification of CATH. Table 3 shows the percentage of cases in which the two protein structures that are compared have the same fold. The data of the homo-oligomers are not given in the table since they have obviously the same fold.

**Table 3:** **Percentage of pairs of protein structures in various datasets that have the same or different fold according to the CATH database**

Dataset	Different fold	Same fold
Hetero-dimers	63.3	36.6
Hetero-trimers	66.7	33.3
Hetero-tetramers	54.3	45.7
Random set 1	77.1	22.8
Random set 2	79.9	20.1
Random set 3	83.8	16.2
Random set 4	85.3	14.7
Random set 5	78.9	21.1
Random set 6	82.9	17.1
Monomer set 1	93.8	16.2
Monomer set 2	83.1	16.9
Monomer set 3	72.2	27.8
Monomer set 4	89.6	10.4
Monomer set 5	66.3	33.7
Monomer set 6	69.0	31.0

33%-46% of the pairs of protomers of hetero-oligomers have the same fold. These values are considerably larger than those of non-interacting proteins (10-34%). This clearly suggests that it is more probable that two protomers of the same hetero-oligomeric complex have the same fold than two non-interacting proteins. This result perfectly agrees with the analogous results based on the dpp distances on the proteomic Ramachandran plot and on the m-scores computed after optimal superposition of two protein structures.

### Comparisons based on SCOP

SCOP is another database and classification of protein structural domain. Like CATH (examined in the previous chapter), SCOP adopts a hierarchical classification scheme, albeit different from that of CATH.

The first classification level is the “class”, like in CATH, the second is the “fold” and this corresponds to the topology level of classification of CATH. In SCOP, therefore, there is not an intermediate level of classification between the class and the fold (this intermediate classification level is the architecture node of CATH). After the fold classification level, there are further levels of clustering (“superfamily” and “family”), which include evolutionary information. Moreover, the definition of protein domain is slightly different, with the consequence that the protein domains tend to be larger in SCOP than in CATH.

Here, like for the comparison based on CATH, I limit the attention to the fold level of structural classification of the protein domains. Two structures are then considered to be similar if they have the same fold and different if they do not. Table 4 shows the results of this analysis. The frequency with which two protomers of a hetero-oligomer have the same fold (26-36%) is considerably higher than the frequency with which two non-interacting proteins have the same fold (8-18%). In agreement with the data reported above, this indicates that the protomers of hetero-oligomers tend to be structurally more similar than expected.

**Table 4:** **Percentage of pairs of protein structures in various datasets that have the same or different fold according to the SCOP database**

Dataset	Different fold	Same fold
Hetero-dimers	74.0	26.0
Hetero-triimers	75.9	24.1
Hetero-tetramers	64.4	35.6
Random set 1	89.5	10.5
Random set 2	90.6	8.4
Random set 3	91.4	8.6
Random set 4	90.1	9.9
Random set 5	90.9	9.1
Random set 6	91.1	8.9
Monomer set 1	89.6	10.4
Monomer set 2	83.0	17.0
Monomer set 3	86.2	13.8
Monomer set 4	89.2	10.8
Monomer set 5	81.9	18.1
Monomer set 6	88.2	11.8

## Discussion

The present data indicate that protomers of hetero-oligomers tend to have three-dimensional structures surprisingly similar, much more than pairs of non-interacting proteins. Higher m-scores and lower dpp distances are observed when two protomers of the same hetero-oligomer are compared than when two unrelated and non-interacting proteins are compared. Two protomers of the same hetero-oligomer have the same fold, according to the classifications of CATH and SCOP, more frequently than two unrelated and non-interacting proteins.

This is per se an interesting observation and several possible explanations can be proposed to explicate it, though none of them can be definitely proven just on the basis of data mining analyses.

First, this might depend on the data paucity. In effect, the three-dimensional structures of only few thousands of homo- and hetero-protein protein complexes are available in the databases. This might seem surprising, given the tremendous increase of the number of new protein three-dimensional structures that have been determined in the last few years. However, only a small fraction of these new structural results involve supramolecular assemblies that have never been analyzed before. It is therefore possible that the higher than expected similarity between protomers of hetero-oligomeric complexes is not a genuine feature of Nature but only an observation that depends on the incomplete sampling of the protein universe.

A second possible explanation is that everything is merely casual, in the statistical sense. In other words, there would be no evolutionary of physico-chemical restrictions that make protomers of hetero-oligomers more similar than expected. Although none can confute this hypothesis on the basis of theoretical considerations or on the basis of some clever experiment, most of us will not believe that such a curious observation is only accidental because of mere epistemological considerations.

A physico-chemical approach can also be adopted to propose intriguing hypotheses. For example, it is possible that the protomers of the hetero-oligomers need a similar flexibility and it is reasonable to suppose that a similar flexibility can be provided by similar folds (Marsh and Teichmann 2014). A similar three-dimensional structure might also guarantee comparable thermodynamic stabilities of the two protomers of a hetero-dimer and comparable degradation pathways and rates, ensuring the coupled degradation of the hetero-dimer when the protein must be eliminated.

The surprising similarity between protomers of hetero-oligomers might be the result of a particular evolutionary pathway. A hetero-dimer, for example, might result from a gene duplication and a subsequent divergent evolution of the two genes. At the amino acid sequence level, the homology between the two protomers could be undetectable, though the similarity of the three-dimensional structures of the two protomers could persist. Alternatively, one can hypothesize that one of the protomers of a homo-dimer could be replaced, by mistake, by another protein; the latter one would be probably very similar to the protomer that it replaces and, as a consequence, the two proteins that are found in the newly formed hetero-dimers would be structurally similar. Although these two scenarios do not take into account the chemical mechanisms of the evolutionary processes, they might suggest interesting scientific scenarios.

This hypothesis is supported by previous studies where it was proposed that hetero-dimeric proteins are evolutionary related to earlier homo-dimeric proteins and that structural similarity enhances the interaction propensity of proteins (Archibald et al. 1999; Ispolatov et al. 2005; Lukatsky et al. 2007; Lukatsky et al. 2006; Pereira-Leal et al. 2007). The data presented here agree with this hypothesis. In particular I should mention that the average m-score between protomers of hetero-dimers that belong to the same SCOP superfamily (72.5) is considerably larger than the average m-score between protomers that belong to different SCOP superfamilies (24.4), which is not much higher than the average m-score between proteins that do not interact with each other (around 20, see Table 2).

Obviously, other more or less interesting and reasonable hypotheses can be proposed. However, the important point, here, is that the observation, on a large and controlled dataset, of the relationship between individual protomers of hetero-oligomers is an interesting piece of the emerging mosaic of studies on protein oligomerzation, its evolution and its physicochemical background (Hall et al. 2013; Levy et al. 2012; Levy et al. 2008; Zhang et al. 2013).

## Methods

The three-dimensional structures of the protein-protein complexes were taken from the database 3Dcomplex (Levy et al. 2006) from a precompiled non-redundant list (30% level) and from the Protein Data Bank. (Berman et al. 2000; Bernstein et al. 1977). The attention was limited to dimers, trimers, and tetramers because of the paucity of higher complexes. In order to emphasize the differences between homo- and hetero-oligomers, these complexes were considered to be homo-oligomeric if all their polypeptides chains (two for the dimmers, three for the tetramers, and four for the tetramers) were identical (percentage of sequence identity ≥90%); they were considered to be hetero-oligomeric if each of their polypeptide chains was different from the others (percentage of sequence identity ≤40%). It must be remembered that some of the quaternary status assignments might be wrong. However, this would cause an underestimation of the results presented in this article. The attention was limited to globular proteins, by rejecting membrane proteins with the THMM server (http://www.cbs.dtu.dk/services/TMHMM/; (Krogh et al. 2001)) and coiled-coils protein with the MULTICOIL2 software (Trigg et al. 2011). This was necessary to eliminated oligomers where the similarity between the protomers was implicit in their structural organizations. This resulted in an ensemble of 1837 homo-dimers, 232 homo-trimers, 554 homo-tetramers, 421 hetero-dimers, 107 hetero-trimers, and 38 hetero-tetramers (see Additional file 1).

In order to compare the levels of similarity of the structures of the protomers in oligomeric complexes with the levels of similarity between non-interacting protein pairs, it was necessary to build several, non-redundant control datasets. I followed two strategies.

The first strategy was based on the assumption that a polypeptide chain found in a hetero-oligomeric complex does not interact with a protein chain found in a homo-oligomeric complex. Therefore I took (arbitrarily) the first chain in each hetero-oligomeric complex and the first chain in each homo-oligomeric complex and randomly build six ensembles of pairs of protein structures; each pair is formed by a hetero- and a homo-oligomeric protein chain; and each of the six ensembles contains 1,000 pairs of structures. These datasets were named “Random set 1”, “Random set 2” etc.

The second strategy was based on the assumption that monomeric proteins do not interact with other monomeric proteins to form stable complexes. Therefore, all monomeric proteins (according to the quaternary status annotation of the Protein Data Bank) were downloaded from the Protein Data Bank (Berman et al. 2000; Bernstein et al. 1977). The redundancy was reduced to a percentage of pairwise sequence identity lower than 40% with CD-HIT (Fu et al. 2012). Six datasets of pairs of monomeric proteins were randomly, each with 1,000 pairs of protein structures. These datasets were named “Monomer set 1”, “Monomer set 2” etc.

Some basic statistical descriptors of all the datasets described above are shown in Table 5. The average length, measured by the number of residues, of the proteins examined here is not very variable amongst the various datasets. Homo-oligomers seem to be slightly longer than hetero-oligomers and the twelve control datasets have and average length that is close to both homo- and hetero-oligomers. The percentage of sequence identity is obviously very close to 100% for pair of protomers of homo-oligomers. It is on the contrary very small (7-10%) for all the other datasets, indicating that redundancy was successfully removed.

**Table 5:** **Average length, measured by the number of residues and average percentage of sequence identity computed after Needleman-Wunsch alignment of the sequences of the two proteins**

Data set	Average length	Average % sequence identity
Homo-dimers	250.0 (2.5)	99.8 (0.1)
Homo-trimers	237.4 (4.1)	99.9 (0.1)
Homo-tetramers	268.3 (1.9)	99.9 (0.1)
Hetero-dimers	196.6 (5.6)	8.5 (0.4)
Hetero-trimers	212.5 (7.2)	9.6 (0.5)
Hetero-tetramers	173.8 (6.2)	9.2 (0.5)
Random set 1	233.5 (3.3)	7.2 (0.2)
Random set 2	236.8 (3.4)	7.1 (0.1)
Random set 3	222.8 (3.4)	6.9 (0.1)
Random set 4	228.4 (3.3)	7.3 (0.1)
Random set 5	234.1 (3.3)	7.0 (0.2)
Random set 6	216.5 (3.4)	6.9 (0.1)
Monomer set 1	175.4 (2.3)	7.7 (0.1)
Monomer set 2	186.0 (2.3)	7.8 (0.1)
Monomer set 3	176.1 (2.3)	6.9 (0.1)
Monomer set 4	196.1 (2.4)	8.2 (0.1)
Monomer set 5	174.7 (2.3)	8.5 (0.2)
Monomer set 6	195.3 (2.3)	8.9 (0.2)

Sequence alignments were performed with the Needleman-Wunsch algorithm (Needleman and Wunsch 1970) with the NEELDE program of the EMBOSS software suite with defaults parameters (Rice et al. 2000). Amino acid sequences were extracted from the PDB-formatted files with the STRIDE program (Frishman and Argos 1995). The values of the torsion angles phi and psi were computed with DSSP (Kabsch and Sander 1983). They were averaged with circular statistics techniques (Batschelet 1981) as it was previously described (Carugo and Djinović-Carugo 2013). The distances between two protein structures i and j (dpp_ij_) on phi/psi space of the Proteomic Ramachandran plot (PRplot) were computed as:


Since both torsion angles are periodical quantities with values that can range conventionally from -180° to +180°, the following transformations were applied:


Superpositions between protein three-dimensional structures were performed with Sheba (Jung and Lee 2000), by considering only the Calpha carbon atoms. The degree of similarity between two structures was determined, after their optimal superposition, with the m-scores:


where n_1_ and n_2_ are the number of residues in the two structures and n_ali_ is the number of Calpha carbon atoms that can be aligned.

## Conclusions

It has been shown that protein protomers that form hetero-oligomeric complexes tend to have structures more similar to each other than proteins that do not form this type of supramolecular assemblies. A series of different approaches have contributed to this observation: distances on the proteomic Ramachandran plots, protein structure superpositions, and comparisons based on two domain structure databases (CATH and SCOP).

In agreement with previous studies, it is reasonable to suppose that this surprising similarity between protomers of hetero-oligomeric complexes is due to the evolutionary relationship between hetero-oligomers and earlier homo-oligomers, though gene duplication and paralogs evolution (Archibald et al. 1999; Ispolatov et al. 2005; Lukatsky et al. 2007; Lukatsky et al. 2006; Pereira-Leal et al. 2007). However, in my opinion, further studies are necessary to evaluate the relative importance of evolutionary and physico-chemical restraints on protein structure and dynamics.

## Electronic supplementary material

Additional file 1: **List of the protein crystal structures examined in the present manuscript.** (DOCX 112 KB)

## References

[CR1] Andreeva A, Howorth D, Chandonia JM, Brenner SE, Hubbard TJ, Chothia C, Murzin AG (2008). Data growth and its impact on the SCOP database: new developments. Nucleic Acids Res.

[CR2] Archibald JM, Logsdon JMJ, Doolittle WF (1999). Recurrent paralogy in the evolution of archeal chaperonins. Curr Biol.

[CR3] Batschelet E (1981). Circular Statistics in Biology.

[CR4] Berman HM, Westbrook J, Feng Z, Gilliland G, Bhat TN, Weissig H, Shindyalov IN, Bourne PE (2000). The protein data bank. Nucleic Acids Res.

[CR5] Bernstein FC, Koetzle TF, Williams GJ, Meyer EF, Brice MD, Rodgers JR, Kennard O, Shimanouchi T, Tasumi M (1977). The protein data bank: a computer-based archival file for macromolecular structures. J Mol Biol.

[CR6] Bordo D, Djinović K, Bolognesi M (1994). Conserved patterns in the Cu, Zn superoxide dismutase family. J Mol Biol.

[CR7] Bordo D, Matak D, Djinovic-Carugo K, Rosano C, Pesce A, Bolognesi M, Stroppolo ME, Falconi M, Battistoni A, Desideri A (1999). Evolutionary constraints for dimer formation in prokaryotic Cu, Zn superoxide dismutase. J Mol Biol.

[CR8] Carugo O (2006). Rapid methods for comparing protein structures and scanning structure databases. Curr Bioinformatics.

[CR9] Carugo O (2007). Recent progress in measuring structural similarity between proteins. Curr Protein Pept Sci.

[CR10] Carugo O (2007). A structural proteomics filter: prediction of the quaternary structural type of hetero-oligomeric proteins on the basis of their sequences. J Appl Cryst.

[CR11] Carugo O, Djinović-Carugo K (2013). A proteomic Ramachandran plot (PRplot). Amino Acids.

[CR12] Carugo O, Pongor S (2001). A normalized root-mean-square distance for comparing protein three-dimensional structures. Protein Sci.

[CR13] Carugo O, Pongor S (2002). Recent progress in protein 3D structure comparison. Curr Protein Pept Sci.

[CR14] Cech T (2000). The ribosome is a ribozyme. Science.

[CR15] Frishman D, Argos P (1995). Knowledge-based protein secondary structure assignment. Proteins.

[CR16] Fu L, Niu B, Zhu Z, Wu S, Li W (2012). CD-HIT: accelerated for clustering the next generation sequencing data. Bioinformatics.

[CR17] Hall Z, Gernandez H, Marsh JA, Teichmann SA, Robinson CV (2013). The role of salt bridges, charge density, and subunit flexibility in determining disassembly routes of protein complexes. Structure.

[CR18] Ideker T, Krogan NJ (2012). Differential network biology. Mol Syst Biol.

[CR19] Ispolatov I, Yuryev A, Mazo I, Maslov S (2005). Binding proteins and evolution of homodimers in protein-protein interaction networks. Nucleic Acids Res.

[CR20] Jung J, Lee B (2000). Protein structure alignment using environmental profiles. Protein Eng.

[CR21] Kabsch W, Sander C (1983). Dictionary of protein secondary structure: Pettern recognition of hydrogen-bonded and geometrical features. Biopolymers.

[CR22] Krogh A, Larsson B, von Heijne G, Sonnhammer EL (2001). Predicting transmembrane protein topology with a hidden Markov model: application to complete genomes. J Mol Biol.

[CR23] Lee TI, Young RA (2000). Transcription of eukaryotic protein-coding genes. Annu Rev Genet.

[CR24] Levy ED, Pereira-Leal JB, Chothia C, Teichmann SA (2006). 3D complex: a structural classification of protein complexes. PLoS Comput Biol.

[CR25] Levy ED, Erba EB, Robinson CS, Teichmann SA (2008). Assembly reflects evolution of protein complexes. Nature.

[CR26] Levy ED, De S, Teichmann SA (2012). Cellular crowding imposes global constraints on the chemistry and evolution of proteomes. Proc Natl Acad Sci U S A.

[CR27] Lukatsky DB, Zeldovich KB, Shakhnovich EI (2006). Statistically enhanced self-attraction of random patterns. Phys Rev Lett.

[CR28] Lukatsky DB, Shakhnovich BE, Mintseris J, Shakhnovich EI (2007). Structural similarity enhances interaction propensity of proteins. J Mol Biol.

[CR29] Marsh JA, Teichmann SA (2014). Parallel dynamics and evolution: protein conformational fluctuations and assembly reflect evolutionary changes in sequence and structure. Bioessays.

[CR30] Needleman SB, Wunsch CD (1970). A general method applicable to the search for similarities in the amino acid sequence of two proteins. J Mol Biol.

[CR31] Ogata H, Lubitz W, Higuchi Y (2009). [NiFe] hydrogenases: structural and spectroscopic studies of the reaction mechanism. J Chem Soc Dalton Trans.

[CR32] Orchard S (2012). Molecular interaction databases. Proteomics.

[CR33] Orengo CA, Michie AD, Jones S, Jones DT, Swindells MB, Thornton JM (1997). CATH–a hierarchic classification of protein domain structures. Structure.

[CR34] Pereira-Leal JB, Levy ED, Kamp C, Teichmann SA (2007). Evolution of protein complexes by duplication of homomeric interactions. Genome Biol.

[CR35] Rice P, Longden I, Bleasby A (2000). EMBOSS: the European Molecular Biology Open Software Suite. Trends Genet.

[CR36] Sillitoe I, Cuff AL, Dessailly BH, Dawson NL, Furnham N, Lee D, Lees JG, Lewis TE, Studer RA, Rentzsch R, Yeats C, Thornton JM, Orengo CA (2013). New functional families (FunFams) in CATH to improve the mapping of conserved functional sites to 3D structures. Nucleic Acids Res.

[CR37] Trigg J, Gutwin K, Keating AE, Berger B (2011). Multicoil2: Predicting coiled coils and their oligomerization states from sequence in the twilight zone. PLoS One.

[CR38] Zhang X, Perica T, Teichmann SA (2013). Evolution of protein structures and interactions from the perspective of residue contact networks. Curr Opin Struct Biol.

