# Supplementary material for: Protomers of protein hetero-oligomers tend to resemble each other more than expected
Source: Springerplus. 2014 Nov 20;3:680. doi: 10.1186/2193-1801-3-680 (PMC4447755; doi:10.1186/2193-1801-3-680)
Supplement: Supplementary file 1 — Additional file 1: List of the protein crystal structures examined in the present manuscript. (DOCX 112 KB) [file 40064_2014_1571_MOESM1_ESM.docx]

The identification codes of the oligomers are taken from 3D Complex

The identification codes of the random sets are taken from 3D Complex

The identification codes of the monomer sets are taken from PDB (the list report 6000 pairs of monomers from which, via a random number generator, 6 lists of 1000 pairs of monomers were extracted

Homo-dimers

1 12as.pdb AB;2 137l.pdb AB;3 1a0a.pdb AB;4 1a0g.pdb AB;5 1a19.pdb AB;6 1a1x.pdb AB;7 1a25.pdb AB;8 1a3a_2.pdb BD;9 1a3c.pdb AB;10 1a3q.pdb AB;11 1a4i.pdb AB;12 1a5t.pdb AB;13 1a64.pdb AB;14 1a73.pdb AB;15 1a78.pdb AB;16 1a8l.pdb AB;17 1a8o.pdb AB;18 1a8y.pdb AB;19 1a99_1.pdb AB;20 1aa7.pdb AB;21 1aap.pdb AB;22 1aar.pdb AB;23 1ab4.pdb AB;24 1ab8.pdb AB;25 1ad3.pdb AB;26 1adu.pdb AB;27 1afw.pdb AB;28 1aih_1.pdb AB;29 1ail.pdb AB;30 1ajs.pdb AB;31 1alu.pdb AB;32 1am2.pdb AB;33 1amp.pdb AB;34 1aoc.pdb AB;35 1aoh.pdb AB;36 1aox.pdb AB;37 1aoz.pdb AB;38 1apx_2.pdb CD;39 1aq0.pdb AB;40 1aqt.pdb AB;41 1aqu.pdb AB;42 1at0.pdb AB;43 1au1.pdb AB;44 1au7.pdb AB;45 1aua.pdb AB;46 1auo.pdb AB;47 1avv.pdb AB;48 1awd.pdb AB;49 1awp.pdb AB;50 1ay2.pdb AB;51 1ayf.pdb AB;52 1ayo.pdb AB;53 1azt.pdb AB;54 1azw.pdb AB;55 1b0u.pdb AB;56 1b0x.pdb AB;57 1b2k.pdb AB;58 1b3q.pdb AB;59 1b3t.pdb AB;60 1b43.pdb AB;61 1b49.pdb AC;62 1b5p.pdb AB;63 1b63.pdb AB;64 1b6r.pdb AB;65 1b6u.pdb AB;66 1b74.pdb AB;67 1b76.pdb AB;68 1b8a.pdb AB;69 1b8z.pdb AB;70 1b9h.pdb AB;71 1b9m.pdb AB;72 1bb9.pdb AB;73 1bc2.pdb AB;74 1bcm.pdb AB;75 1bdo.pdb AB;76 1bdy.pdb AB;77 1bec.pdb AB;78 1bf3.pdb AB;79 1bg1.pdb AD;80 1bg6.pdb AB;81 1bg9.pdb AB;82 1bgf.pdb AB;83 1bgp.pdb AB;84 1bhh.pdb AB;85 1bht.pdb AB;86 1bih.pdb AB;87 1bjt.pdb AB;88 1bk5.pdb AB;89 1bkf.pdb AB;90 1bm9_1.pdb AB;91 1bmo.pdb AB;92 1bmt.pdb AB;93 1bo1.pdb AB;94 1bow.pdb AB;95 1bqu.pdb AB;96 1brw.pdb AB;97 1bsl.pdb AB;98 1btk.pdb AB;99 1btn.pdb AB;100 1bv1.pdb AB;101 1bw0.pdb AB;102 1bxg.pdb AB;103 1bxt.pdb AB;104 1byf.pdb AB;105 1byi.pdb AB;106 1byk.pdb AB;107 1bys.pdb AB;108 1byu.pdb AB;109 1c02.pdb AB;110 1c0m_1.pdb AB;111 1c0p.pdb AB;112 1c3g.pdb AB;113 1c3r.pdb AB;114 1c4k.pdb AB;115 1c6o.pdb AB;116 1c76.pdb AB;117 1c7n_3.pdb EF;118 1c7s.pdb AB;119 1c8u.pdb AB;120 1c94.pdb AB;121 1c9o.pdb AB;122 1cbf.pdb AB;123 1cbk.pdb AB;124 1cby.pdb AB;125 1cei.pdb AB;126 1chm.pdb AB;127 1chu.pdb AB;128 1ci4.pdb AB;129 1ci8.pdb AB;130 1cku.pdb AB;131 1cl8.pdb AD;132 1cli_2.pdb BD;133 1clx_2.pdb CD;134 1cmc.pdb AB;135 1cnz.pdb AB;136 1cp2.pdb AB;137 1cq3.pdb AB;138 1cqk.pdb AB;139 1cru.pdb AB;140 1crx.pdb AB;141 1cs6.pdb AB;142 1csg.pdb AB;143 1csh.pdb AB;144 1csn.pdb AB;145 1ct9_2.pdb BC;146 1ctf.pdb AB;147 1ctn.pdb AB;148 1ctt.pdb AB;149 1cu1.pdb AB;150 1cv8.pdb AB;151 1cx8_1.pdb AB;152 1cxq.pdb AB;153 1cy9.pdb AB;154 1cyw.pdb AB;155 1czj.pdb AB;156 1d02.pdb AB;157 1d0c.pdb AB;158 1d0n.pdb AB;159 1d0q.pdb AB;160 1d1m.pdb BA;161 1d2f.pdb AB;162 1d2g.pdb AB;163 1d2o.pdb AB;164 1d2s.pdb AB;165 1d3y.pdb AB;166 1d7b.pdb AB;167 1d7x.pdb AB;168 1d7y.pdb AB;169 1d8h_2.pdb AC;170 1d8u.pdb AB;171 1d9c.pdb AB;172 1db3.pdb AB;173 1dbq.pdb AB;174 1dbt_1.pdb AB;175 1dc1.pdb AB;176 1dcf.pdb AB;177 1dd3_1.pdb AB;178 1ddt.pdb AB;179 1ddz.pdb AB;180 1dek.pdb AB;181 1dfm.pdb AB;182 1dfn.pdb AB;183 1dg1.pdb GH;184 1dhf.pdb AB;185 1div.pdb AB;186 1dj0.pdb AB;187 1dj8_3.pdb EF;188 1djt.pdb AB;189 1dk8.pdb AB;190 1dku.pdb AB;191 1dl5.pdb AB;192 1dle.pdb AB;193 1dlj.pdb AB;194 1dmh.pdb AB;195 1dmu.pdb AC;196 1dnl.pdb AB;197 1dos.pdb AB;198 1dov.pdb AB;199 1dpg.pdb AB;200 1dqe.pdb AB;201 1dqn.pdb AB;202 1dqs.pdb AB;203 1dqw_1.pdb AB;204 1dqz.pdb AB;205 1dto.pdb AB;206 1dv1.pdb AB;207 1dvk.pdb AB;208 1dvp.pdb AB;209 1dxg.pdb AB;210 1dxs.pdb AB;211 1dxy.pdb AB;212 1dys.pdb AB;213 1dyw.pdb AB;214 1dza.pdb AB;215 1dzf.pdb AB;216 1e0b.pdb AB;217 1e19.pdb AB;218 1e1o.pdb AB;219 1e1z.pdb PA;220 1e2k.pdb AB;221 1e3m.pdb AB;222 1e4m.pdb MA;223 1e58.pdb AB;224 1e5r.pdb AB;225 1e6b.pdb AB;226 1e6u.pdb AB;227 1e7l.pdb AB;228 1e7n.pdb AB;229 1e85.pdb AB;230 1e87.pdb AB;231 1e8u.pdb AB;232 1e9g.pdb AB;233 1e9p.pdb AB;234 1eag.pdb AB;235 1eaj.pdb AB;236 1ear.pdb AB;237 1ebf.pdb AB;238 1ecj_1.pdb AB;239 1ecs.pdb AB;240 1ecy.pdb AB;241 1edh.pdb AB;242 1edt.pdb AB;243 1edz.pdb AB;244 1ee8.pdb AB;245 1eeq.pdb AB;246 1efa_1.pdb AB;247 1eg5.pdb AB;248 1ega.pdb AB;249 1egw_2.pdb CD;250 1eh9.pdb AB;251 1ehi.pdb AB;252 1ei5.pdb AB;253 1ei6_1.pdb AD;254 1eje.pdb AB;255 1eke.pdb AB;256 1eku.pdb AB;257 1elu.pdb AB;258 1eny.pdb AB;259 1eo6.pdb AB;260 1epa.pdb AB;261 1epf_2.pdb CD;262 1eqt.pdb AB;263 1ern.pdb AB;264 1erv.pdb AB;265 1es9.pdb AB;266 1esc.pdb AB;267 1ete_2.pdb CD;268 1etx.pdb AB;269 1ev7.pdb AB;270 1evj_1.pdb AC;271 1evl_2.pdb CD;272 1evy.pdb AB;273 1ew3.pdb AB;274 1ew6.pdb AB;275 1ewk.pdb AB;276 1ex0.pdb AB;277 1ex2.pdb AB;278 1ex4.pdb AB;279 1ext.pdb AB;280 1eye.pdb AB;281 1eyv.pdb AB;282 1ez0_1.pdb AD;283 1ezg.pdb AB;284 1ezi.pdb AB;285 1f08.pdb AB;286 1f0k.pdb AB;287 1f0y.pdb AB;288 1f1e.pdb AB;289 1f1m_2.pdb CD;290 1f2d_1.pdb AB;291 1f2v.pdb AB;292 1f3h.pdb AB;293 1f46.pdb AB;294 1f5m.pdb AB;295 1f5v.pdb AB;296 1f6b.pdb AB;297 1f6y.pdb AB;298 1f86.pdb AB;299 1f89.pdb AB;300 1f9z.pdb AB;301 1fbl.pdb AB;302 1fbt.pdb AB;303 1fcg.pdb AB;304 1fd3_1.pdb AC;305 1fe0.pdb AB;306 1few.pdb AB;307 1ff4.pdb AB;308 1ff9.pdb AB;309 1fg7.pdb AB;310 1fgu.pdb AB;311 1fi4.pdb AB;312 1fic.pdb AB;313 1fjj.pdb AB;314 1fjl_2.pdb BC;315 1flg.pdb AB;316 1flk_2.pdb AB;317 1flm.pdb AB;318 1fn9.pdb AB;319 1fno.pdb AB;320 1foc.pdb AB;321 1fp1.pdb DA;322 1fp2.pdb AB;323 1fp3.pdb AB;324 1fp5.pdb AB;325 1fqt.pdb AB;326 1fr8.pdb AB;327 1fs8.pdb AB;328 1fsy.pdb AB;329 1ft9.pdb AB;330 1fvk.pdb AB;331 1fwx_2.pdb CD;332 1fx2.pdb AB;333 1g0s.pdb AB;334 1g29.pdb 12;335 1g2q.pdb AB;336 1g2y_2.pdb BD;337 1g5c_2.pdb CD;338 1g5h_2.pdb CD;339 1g5t.pdb AB;340 1g60.pdb AB;341 1g6u.pdb AB;342 1g8l.pdb AB;343 1g8m.pdb AB;344 1g8q.pdb AB;345 1g8s.pdb AB;346 1g8t.pdb AB;347 1g8x.pdb AB;348 1gcj.pdb AB;349 1gct.pdb AC;350 1gdt.pdb AB;351 1ge7.pdb AB;352 1gfl.pdb AB;353 1gjw.pdb AB;354 1gk6.pdb BA;355 1gmj_1.pdb AB;356 1gml_1.pdb AD;357 1gmv.pdb AB;358 1go8.pdb PA;359 1goi.pdb AB;360 1gpe.pdb AB;361 1gpj.pdb AB;362 1gpr.pdb AB;363 1gpu.pdb AB;364 1gqi.pdb AB;365 1gqn.pdb AB;366 1gqp.pdb AB;367 1gqy.pdb AB;368 1gri.pdb AB;369 1gs5.pdb AB;370 1gt3.pdb AB;371 1gt6.pdb AB;372 1gt9.pdb 12;373 1gte_1.pdb AB;374 1gtw.pdb AB;375 1gu2.pdb AB;376 1gu3.pdb AB;377 1gu7.pdb AB;378 1gud.pdb AB;379 1gve.pdb AB;380 1gvj.pdb AB;381 1gvp.pdb AB;382 1gw0.pdb AB;383 1gwb.pdb AB;384 1gwi.pdb AB;385 1gwn.pdb AC;386 1gwy.pdb AB;387 1gx4.pdb AB;388 1gx5.pdb AB;389 1gxj.pdb AB;390 1gxq.pdb AB;391 1gxr.pdb AB;392 1gxy.pdb AB;393 1gy6.pdb AB;394 1gyo.pdb AB;395 1gyy.pdb AB;396 1gz0_1.pdb AF;397 1gzj.pdb AB;398 1h03.pdb PQ;399 1h16.pdb AB;400 1h1o.pdb AB;401 1h1w.pdb AB;402 1h1y.pdb AB;403 1h2b.pdb AB;404 1h3f.pdb AB;405 1h4g.pdb AB;406 1h4p.pdb AB;407 1h4r.pdb AB;408 1h4v.pdb BA;409 1h54.pdb AB;410 1h5b_2.pdb CD;411 1h65_2.pdb BC;412 1h6o.pdb AB;413 1h6p.pdb AB;414 1h6u.pdb AB;415 1h72.pdb CA;416 1h7b.pdb AB;417 1h7e.pdb AB;418 1h80.pdb AB;419 1h82_2.pdb AC;420 1h99.pdb AB;421 1h9r.pdb AB;422 1hav.pdb AB;423 1hc7_1.pdb AB;424 1hci.pdb AB;425 1hcq_2.pdb EF;426 1hcx.pdb AB;427 1hdh.pdb AB;428 1he7.pdb AB;429 1he9.pdb AB;430 1hei.pdb AB;431 1hf2_1.pdb AB;432 1hg5.pdb AB;433 1hh2.pdb PA;434 1hjr_2.pdb BD;435 1hk7.pdb AB;436 1hkq.pdb AB;437 1hlg.pdb AB;438 1hlo.pdb AB;439 1hn4.pdb AB;440 1hng.pdb AB;441 1hnj.pdb AB;442 1hoz.pdb AB;443 1hpc.pdb AB;444 1hqs.pdb AB;445 1hrh.pdb AB;446 1hru.pdb AB;447 1hsj.pdb AB;448 1hsl.pdb AB;449 1hss_1.pdb AB;450 1hul.pdb AB;451 1hur.pdb AB;452 1huw.pdb AB;453 1hux.pdb AB;454 1hv8.pdb AB;455 1hw1.pdb AB;456 1hw5.pdb AB;457 1hw7.pdb AB;458 1hxp.pdb AB;459 1hyn_2.pdb RS;460 1hyo.pdb AB;461 1hyu.pdb AB;462 1hz4.pdb AB;463 1hzy.pdb AB;464 1i07.pdb AB;465 1i0r.pdb AB;466 1i12_1.pdb AC;467 1i24.pdb AB;468 1i2k.pdb AB;469 1i2p.pdb AB;470 1i2s.pdb AB;471 1i36.pdb AB;472 1i3k.pdb AB;473 1i49.pdb AB;474 1i4j.pdb AB;475 1i4m.pdb AB;476 1i4u.pdb AB;477 1i52.pdb AB;478 1i58.pdb AB;479 1i69.pdb AB;480 1i6l.pdb AB;481 1i75.pdb AB;482 1i7n.pdb AB;483 1i86.pdb AB;484 1i8t.pdb AB;485 1ia9.pdb AB;486 1iag.pdb AB;487 1iam.pdb AB;488 1ic2_1.pdb AB;489 1icr.pdb AB;490 1id1.pdb AB;491 1idr.pdb AB;492 1ied.pdb AB;493 1ig0.pdb AB;494 1ig3.pdb AB;495 1igq_1.pdb AC;496 1ihb.pdb AB;497 1ihk.pdb AB;498 1ii5.pdb AB;499 1ii7.pdb AB;500 1ijy.pdb AB;501 1ilr.pdb 12;502 1iom.pdb AB;503 1ips.pdb AB;504 1iq8.pdb AB;505 1iqc_2.pdb BD;506 1irq.pdb AB;507 1is3.pdb AB;508 1isy.pdb AB;509 1itu.pdb AB;510 1itv.pdb AB;511 1iu8.pdb AB;512 1ivy.pdb AB;513 1ix1.pdb AB;514 1ix2.pdb AB;515 1ix9.pdb AB;516 1ixm.pdb AB;517 1iyb.pdb AB;518 1izm.pdb AB;519 1j0h.pdb AB;520 1j1b.pdb AB;521 1j24.pdb AB;522 1j30.pdb AB;523 1j31_2.pdb CD;524 1j3b.pdb AB;525 1j3m.pdb AB;526 1j5p.pdb AB;527 1j5w.pdb AB;528 1j79.pdb AB;529 1j7n.pdb AB;530 1j8b.pdb AB;531 1j8u.pdb AB;532 1j98.pdb AB;533 1j9l.pdb AB;534 1ja3.pdb AB;535 1jad.pdb AB;536 1jay.pdb AB;537 1jc4_2.pdb CD;538 1jd0.pdb AB;539 1jdf_1.pdb AB;540 1jdw.pdb AB;541 1jer.pdb AB;542 1jfl.pdb AB;543 1jfz_1.pdb AB;544 1jgs.pdb AB;545 1jgt.pdb AB;546 1jhc.pdb AB;547 1jhd.pdb AB;548 1jhg.pdb AB;549 1ji3.pdb AB;550 1jil.pdb AB;551 1jke_1.pdb AD;552 1jkw.pdb AB;553 1jkx_1.pdb AB;554 1jl0.pdb AB;555 1jl9.pdb AB;556 1jlv_1.pdb AB;557 1jlx.pdb AB;558 1jm0_1.pdb AB;559 1jm6.pdb AB;560 1jmj.pdb AB;561 1jmk.pdb CO;562 1jmv_2.pdb CD;563 1joc.pdb AB;564 1jog_2.pdb CD;565 1jpy_1.pdb AB;566 1jr8.pdb AB;567 1js3.pdb AB;568 1jtv.pdb AB;569 1jub.pdb AB;570 1juv.pdb AB;571 1jv1.pdb AB;572 1jya.pdb AB;573 1jye.pdb AB;574 1jys.pdb AB;575 1jzo.pdb AB;576 1k04.pdb AB;577 1k0d_1.pdb AB;578 1k2f.pdb AB;579 1k3e.pdb AB;580 1k3s.pdb AB;581 1k3y.pdb AB;582 1k4i.pdb AB;583 1k4z_2.pdb AB;584 1k51.pdb AB;585 1k55_1.pdb AC;586 1k66.pdb AB;587 1k6d.pdb AB;588 1k94.pdb AB;589 1ka2.pdb AB;590 1ka8_1.pdb AB;591 1kae.pdb AB;592 1kc7.pdb AB;593 1kcm.pdb AB;594 1kcx.pdb AB;595 1kdg.pdb AB;596 1kez_1.pdb AB;597 1kfi.pdb AB;598 1kgn_2.pdb CD;599 1khd_2.pdb BC;600 1khh.pdb AB;601 1kij.pdb AB;602 1kix.pdb AC;603 1kiy.pdb AB;604 1kj1_2.pdb PQ;605 1kjn.pdb AB;606 1kjq.pdb AB;607 1kko.pdb AB;608 1kl1.pdb AB;609 1knq.pdb AB;610 1kny.pdb AB;611 1koa.pdb AB;612 1kob.pdb AB;613 1kpf.pdb AB;614 1kpt.pdb AB;615 1kqp.pdb AB;616 1ksi.pdb AB;617 1kso.pdb AB;618 1ktb.pdb AB;619 1ktj.pdb AB;620 1ktn.pdb AB;621 1ku2.pdb AB;622 1ku7.pdb AD;623 1ku9.pdb AB;624 1kut.pdb AB;625 1kvk.pdb AB;626 1kyq_2.pdb AC;627 1kzq.pdb AB;628 1l0w.pdb AB;629 1l3l_1.pdb BD;630 1l3p.pdb AB;631 1l4i.pdb AB;632 1l5o.pdb AB;633 1l6l_7.pdb MN;634 1l6r.pdb AB;635 1l7d_2.pdb CD;636 1l8d.pdb AB;637 1lc5.pdb AB;638 1lfa.pdb AB;639 1lfb.pdb AB;640 1lh0.pdb AB;641 1lid.pdb AB;642 1lj9.pdb AB;643 1ll2.pdb AB;644 1llf.pdb AB;645 1llm.pdb CD;646 1lm5.pdb AB;647 1lmb.pdb 34;648 1lns.pdb AB;649 1lom.pdb AB;650 1lq1_1.pdb CD;651 1lq9.pdb AB;652 1lql_2.pdb CD;653 1lrh_2.pdb BC;654 1lss_1.pdb AB;655 1lwd.pdb AB;656 1lwj.pdb AB;657 1lxd.pdb AB;658 1lxe.pdb AB;659 1ly1.pdb AB;660 1m0d_2.pdb CD;661 1m0w.pdb AB;662 1m1f.pdb AB;663 1m2d.pdb AB;664 1m3e_1.pdb AB;665 1m4i.pdb AB;666 1m4r.pdb AB;667 1m4z.pdb AB;668 1m6d.pdb AB;669 1m6e.pdb XA;670 1m6k.pdb AB;671 1m6p.pdb AB;672 1m7r.pdb AB;673 1m7y.pdb AB;674 1m98.pdb AB;675 1mby.pdb AB;676 1mdy_2.pdb CD;677 1mh9.pdb AB;678 1mi1.pdb AB;679 1mi3_2.pdb CD;680 1mi8.pdb AB;681 1miw.pdb AB;682 1mjf.pdb AB;683 1mjh.pdb AB;684 1mji.pdb AB;685 1mk4.pdb AB;686 1mka.pdb AB;687 1mkf.pdb AB;688 1mkk.pdb AB;689 1mkz.pdb AB;690 1mld_1.pdb AB;691 1mml.pdb AB;692 1mo9.pdb AB;693 1moe.pdb AB;694 1moq.pdb AB;695 1mp9.pdb AB;696 1mqi.pdb AB;697 1mr8.pdb AB;698 1mrp.pdb AB;699 1mrz.pdb AB;700 1msc.pdb AB;701 1mu4.pdb AB;702 1mun.pdb AB;703 1mus.pdb AF;704 1mv8_2.pdb CD;705 1mvo.pdb AB;706 1mwq.pdb AB;707 1mwv.pdb AB;708 1mxb.pdb AB;709 1mxi.pdb AB;710 1mxr.pdb AB;711 1my7.pdb AB;712 1mz0.pdb AB;713 1mzb.pdb AB;714 1mze.pdb AB;715 1mzg.pdb AB;716 1n1b.pdb AB;717 1n1c.pdb AB;718 1n1d_2.pdb CD;719 1n26.pdb AB;720 1n2a.pdb AB;721 1n2f.pdb AB;722 1n2s.pdb AB;723 1n3l.pdb AB;724 1n55.pdb AB;725 1n57.pdb AB;726 1n69_1.pdb AB;727 1n71_1.pdb AB;728 1n7v.pdb AB;729 1n7z_2.pdb CD;730 1na6.pdb AB;731 1na8.pdb AB;732 1nbc.pdb AB;733 1nbq.pdb AB;734 1nco.pdb AB;735 1nd4.pdb AB;736 1nd6_2.pdb CD;737 1ne2.pdb AB;738 1ne8.pdb AB;739 1neu.pdb AB;740 1nez_2.pdb GH;741 1nf9.pdb AB;742 1nh8.pdb AB;743 1ni5.pdb AB;744 1njh.pdb AB;745 1njr.pdb AB;746 1nkd.pdb AB;747 1nki.pdb AB;748 1nkn_2.pdb CD;749 1nkq_3.pdb EF;750 1nkt.pdb AB;751 1nlr.pdb AB;752 1nms.pdb AB;753 1nn5.pdb AB;754 1nnw.pdb AB;755 1no4_1.pdb AB;756 1now_2.pdb AB;757 1nox.pdb AB;758 1noy.pdb AB;759 1np6.pdb AB;760 1nqd.pdb AB;761 1nri.pdb AB;762 1nrv.pdb AB;763 1ns5.pdb AB;764 1nsj.pdb AB;765 1nsz.pdb AB;766 1nt0.pdb AG;767 1nt3.pdb AB;768 1nv7.pdb AB;769 1nvj_3.pdb EF;770 1nw1.pdb AB;771 1nwp.pdb AB;772 1nww.pdb AB;773 1nxh.pdb AB;774 1nxm.pdb AB;775 1nxu.pdb AB;776 1nxz.pdb AB;777 1ny5.pdb AB;778 1nyr.pdb AB;779 1nzi.pdb AB;780 1o0v.pdb AB;781 1o0w.pdb AB;782 1o12.pdb AB;783 1o1h.pdb AB;784 1o22.pdb AB;785 1o3u.pdb AB;786 1o4w.pdb AB;787 1o4z_2.pdb CD;788 1o50.pdb AB;789 1o57_2.pdb CD;790 1o5h.pdb AB;791 1o5k.pdb AB;792 1o5l.pdb AB;793 1o63.pdb AB;794 1o69.pdb AB;795 1o6a.pdb AB;796 1o75.pdb AB;797 1o7z.pdb AB;798 1o81.pdb AB;799 1o89.pdb AB;800 1o94_1.pdb AB;801 1o9p.pdb AB;802 1oaa.pdb AB;803 1ob9.pdb AB;804 1obb.pdb AB;805 1obf.pdb OP;806 1obo.pdb AB;807 1obx.pdb AC;808 1oc2.pdb AB;809 1ocu.pdb AB;810 1oe5.pdb AB;811 1of3.pdb AB;812 1of8.pdb AB;813 1ofz.pdb AB;814 1og5.pdb AB;815 1ogs.pdb AB;816 1oh0.pdb AB;817 1ohw_2.pdb CD;818 1oi2.pdb AB;819 1oi4.pdb AB;820 1oi6.pdb AB;821 1oio.pdb AB;822 1oj4.pdb AB;823 1ojh_5.pdb IJ;824 1ok3.pdb AB;825 1oke.pdb AB;826 1okh.pdb AB;827 1oki.pdb AB;828 1okj_2.pdb CD;829 1ol0.pdb AB;830 1olq.pdb AB;831 1olz.pdb AB;832 1omz.pdb AB;833 1on2.pdb AB;834 1ooe.pdb AB;835 1oq9.pdb AB;836 1oqz.pdb AB;837 1or4.pdb AB;838 1oru.pdb AB;839 1osy.pdb AB;840 1ot6.pdb AB;841 1otj_2.pdb BC;842 1otk.pdb AB;843 1otv.pdb AB;844 1ou8_2.pdb AB;845 1ov9.pdb AB;846 1ova_2.pdb CD;847 1ovn.pdb AB;848 1ox0.pdb AB;849 1oya.pdb AB;850 1oys.pdb AB;851 1p0x.pdb AB;852 1p35_2.pdb AB;853 1p5t.pdb AB;854 1p5z.pdb BA;855 1p65.pdb AB;856 1p6o.pdb AB;857 1p74.pdb AB;858 1p7h_1.pdb LM;859 1p7n.pdb AB;860 1p9e.pdb AB;861 1p9i.pdb AB;862 1p9o.pdb AB;863 1pc3_2.pdb AB;864 1pc6.pdb AB;865 1pcs.pdb AB;866 1pdg_2.pdb AC;867 1pdo.pdb AB;868 1pea.pdb AB;869 1peq.pdb AB;870 1pfo.pdb AB;871 1pgu.pdb AB;872 1phz.pdb AB;873 1pin.pdb AC;874 1piw.pdb AB;875 1pix.pdb AB;876 1pjq.pdb AB;877 1pkh.pdb AB;878 1pkp.pdb AB;879 1pkv.pdb AB;880 1pl5.pdb AS;881 1pm3.pdb AB;882 1pn0_1.pdb AC;883 1pn2_2.pdb CD;884 1pnv.pdb AB;885 1poc.pdb AB;886 1ppv.pdb AB;887 1pqw.pdb AB;888 1prx.pdb AB;889 1psr.pdb AB;890 1ptm.pdb AB;891 1ptq.pdb AB;892 1puc.pdb AB;893 1pv9.pdb AB;894 1pvd.pdb AB;895 1pvg.pdb AB;896 1pvm.pdb AB;897 1pxf.pdb AB;898 1py9.pdb AB;899 1pzs.pdb AB;900 1pzw.pdb AB;901 1q08.pdb AB;902 1q0q.pdb AB;903 1q18.pdb AB;904 1q2h_1.pdb AB;905 1q2w.pdb AB;906 1q3o.pdb AB;907 1q47.pdb AB;908 1q4r.pdb AB;909 1q67.pdb AB;910 1q6o.pdb AB;911 1q6w_6.pdb KL;912 1q77.pdb AB;913 1q7e.pdb AB;914 1q8b.pdb AB;915 1q8r.pdb AB;916 1q98.pdb AB;917 1q9u.pdb AB;918 1qah.pdb AB;919 1qak.pdb AB;920 1qb2.pdb AB;921 1qd1.pdb AB;922 1qf8.pdb AB;923 1qfh.pdb AB;924 1qh3.pdb AB;925 1qh4_1.pdb AB;926 1qha.pdb AB;927 1qi9.pdb AB;928 1qj4.pdb AB;929 1qjh.pdb AB;930 1qjs.pdb AB;931 1qkd.pdb AB;932 1qkk.pdb AB;933 1qkq.pdb AB;934 1qkr.pdb AB;935 1qks.pdb AB;936 1qlw.pdb AB;937 1qme.pdb AB;938 1qmg_2.pdb CD;939 1qmh.pdb AB;940 1qo2.pdb AB;941 1qo7.pdb AB;942 1qo8.pdb AD;943 1qoj.pdb AB;944 1qou.pdb AB;945 1qoy.pdb AB;946 1qoz.pdb AB;947 1qp8.pdb AB;948 1qpa.pdb AB;949 1qpo_2.pdb CD;950 1qq5.pdb AB;951 1qqf.pdb AB;952 1qqg.pdb AB;953 1qqs.pdb AB;954 1qsd.pdb AB;955 1qup.pdb AB;956 1qvz.pdb AB;957 1qwt.pdb AB;958 1qx4.pdb AB;959 1qxm.pdb AB;960 1qyc.pdb AB;961 1qz1.pdb AB;962 1qz8.pdb AB;963 1qz9.pdb AB;964 1qzf_2.pdb CD;965 1qzq.pdb AB;966 1r0m_2.pdb BD;967 1r0v_2.pdb CD;968 1r11.pdb AB;969 1r12.pdb AB;970 1r1d.pdb AB;971 1r1t.pdb AB;972 1r29.pdb AB;973 1r30.pdb AB;974 1r31.pdb AB;975 1r3s.pdb AB;976 1r43.pdb AB;977 1r4c_3.pdb EF;978 1r4w_2.pdb CD;979 1r59.pdb OX;980 1r5p.pdb AB;981 1r61.pdb AB;982 1r6u.pdb AB;983 1r6w.pdb AB;984 1r7a.pdb AB;985 1r7j.pdb AB;986 1r7l.pdb AB;987 1r89.pdb AB;988 1r8d.pdb AB;989 1r8e.pdb AD;990 1r8g.pdb AB;991 1r8j.pdb AB;992 1r9c.pdb AB;993 1r9d.pdb AB;994 1r9f.pdb AF;995 1rcb.pdb AB;996 1rcq.pdb AB;997 1rcw_2.pdb AC;998 1reg.pdb XY;999 1rfy_1.pdb AB;1000 1rgf.pdb AB;1001 1rhc.pdb AB;1002 1rk4.pdb AB;1003 1rkd.pdb AB;1004 1rkt.pdb AB;1005 1rku.pdb AB;1006 1rmd.pdb AB;1007 1rmr.pdb AB;1008 1rq2.pdb AB;1009 1rqb.pdb AB;1010 1rqg.pdb AB;1011 1rqi.pdb AB;1012 1rrm.pdb AB;1013 1rvg_1.pdb AB;1014 1rw0.pdb AB;1015 1rw6.pdb AB;1016 1ry9_1.pdb AB;1017 1rya.pdb AB;1018 1rz1_1.pdb AB;1019 1rzl.pdb AB;1020 1rzn.pdb AB;1021 1s0p.pdb AB;1022 1s12_1.pdb AD;1023 1s28_2.pdb CD;1024 1s3e.pdb AB;1025 1s3o.pdb AB;1026 1s3z.pdb AB;1027 1s4c_2.pdb BC;1028 1s4k.pdb AB;1029 1s4n.pdb AB;1030 1s7h_2.pdb CD;1031 1s7i.pdb AB;1032 1s7k.pdb AB;1033 1s7y.pdb AB;1034 1s7z.pdb AB;1035 1s96.pdb AB;1036 1s9j.pdb AB;1037 1s9r.pdb AB;1038 1sb7.pdb AB;1039 1sby.pdb AB;1040 1sd4.pdb AB;1041 1se8.pdb AB;1042 1sei.pdb AB;1043 1sek.pdb AB;1044 1sf8_2.pdb BH;1045 1sfn.pdb AB;1046 1sfp.pdb AB;1047 1sfx.pdb AB;1048 1sg0.pdb AB;1049 1sg9_1.pdb AC;1050 1sgm.pdb AB;1051 1sh5.pdb AB;1052 1sh8.pdb AB;1053 1sj1.pdb AB;1054 1sjp.pdb AB;1055 1sjy.pdb AB;1056 1skz.pdb AB;1057 1slm.pdb AB;1058 1smo.pdb AB;1059 1smx.pdb AB;1060 1sny.pdb AB;1061 1so2_2.pdb CD;1062 1sox.pdb AB;1063 1sq5_2.pdb AB;1064 1sqd.pdb AB;1065 1sqe.pdb AB;1066 1sqj.pdb AB;1067 1sqs.pdb AB;1068 1squ.pdb AB;1069 1sr7.pdb AB;1070 1sr9.pdb AB;1071 1sry.pdb AB;1072 1ss4.pdb AB;1073 1su8.pdb AB;1074 1sur.pdb AB;1075 1svp.pdb AB;1076 1sw6.pdb AB;1077 1sww.pdb AB;1078 1sx5.pdb AB;1079 1sxh.pdb AD;1080 1syy.pdb AB;1081 1sz2.pdb AB;1082 1szq.pdb AB;1083 1t06.pdb AB;1084 1t0i.pdb AB;1085 1t11.pdb AB;1086 1t1r.pdb AB;1087 1t1v.pdb AB;1088 1t3c.pdb AB;1089 1t3i.pdb AB;1090 1t3j.pdb AB;1091 1t4b.pdb AB;1092 1t56.pdb AB;1093 1t5b.pdb AB;1094 1t5h.pdb XA;1095 1t5o_2.pdb CD;1096 1t62.pdb AB;1097 1t6f.pdb AB;1098 1t6l.pdb AB;1099 1t6n.pdb AB;1100 1t6s.pdb AB;1101 1t6t.pdb 12;1102 1t70_4.pdb GH;1103 1t82_1.pdb AB;1104 1t8q_2.pdb BC;1105 1t8t.pdb AB;1106 1t92.pdb AB;1107 1t98.pdb AB;1108 1t9b.pdb AB;1109 1t9i.pdb AB;1110 1tbx.pdb AB;1111 1tc1.pdb AB;1112 1tc5_1.pdb AB;1113 1td2.pdb AB;1114 1tdj.pdb AB;1115 1te2.pdb AB;1116 1te5.pdb AB;1117 1ted_1.pdb AB;1118 1tfe.pdb AB;1119 1the.pdb AB;1120 1thf.pdb DA;1121 1tht.pdb AB;1122 1thw.pdb AB;1123 1tj7.pdb AB;1124 1tjc.pdb AB;1125 1tlj.pdb AB;1126 1tlk.pdb AB;1127 1tll.pdb AB;1128 1tm0.pdb AB;1129 1to3.pdb AB;1130 1to6.pdb AB;1131 1to9.pdb AB;1132 1trb.pdb AB;1133 1tu1.pdb AB;1134 1tuh.pdb AB;1135 1tuv.pdb AB;1136 1tuw.pdb AB;1137 1tv8.pdb AB;1138 1tvd.pdb AB;1139 1tvl.pdb AB;1140 1tvn.pdb AB;1141 1twd.pdb AB;1142 1twi_1.pdb AB;1143 1twu.pdb AB;1144 1tx3_2.pdb CD;1145 1txg.pdb AB;1146 1txn.pdb AB;1147 1ty0_1.pdb AB;1148 1tyy.pdb AB;1149 1tz9.pdb AB;1150 1tzb.pdb AB;1151 1tzp.pdb AB;1152 1u07.pdb AB;1153 1u0e.pdb AB;1154 1u0m.pdb AB;1155 1u19.pdb AB;1156 1u20.pdb AB;1157 1u5k.pdb AB;1158 1u60_2.pdb CD;1159 1u6l.pdb AB;1160 1u6z.pdb AB;1161 1u7i.pdb AB;1162 1u7n.pdb AB;1163 1u7z_1.pdb AB;1164 1u8s.pdb AB;1165 1u9d.pdb AB;1166 1u9t.pdb AB;1167 1uaa.pdb AB;1168 1ual.pdb AB;1169 1uaz.pdb AB;1170 1ub0.pdb AB;1171 1ub9.pdb AB;1172 1uby.pdb AB;1173 1uc2.pdb AB;1174 1uc3_5.pdb IJ;1175 1ucr.pdb AB;1176 1ud0_1.pdb AB;1177 1udu.pdb AB;1178 1udv.pdb AB;1179 1ueh.pdb AB;1180 1ufi_1.pdb AB;1181 1ufo_1.pdb AB;1182 1uis.pdb AB;1183 1uiu.pdb AB;1184 1uix.pdb AB;1185 1uk8.pdb AB;1186 1ukk.pdb AB;1187 1ukw.pdb AB;1188 1ulk.pdb AB;1189 1uly.pdb AB;1190 1ump_3.pdb AC;1191 1un8.pdb AB;1192 1uq5.pdb AB;1193 1uqt.pdb AB;1194 1ur5.pdb AC;1195 1urh.pdb AB;1196 1uru.pdb AB;1197 1usc.pdb AB;1198 1usi.pdb AC;1199 1usl_3.pdb AE;1200 1uso.pdb AB;1201 1ut7.pdb AB;1202 1ut8.pdb AB;1203 1utg.pdb AB;1204 1uth.pdb AB;1205 1uty.pdb AB;1206 1uu1_2.pdb CD;1207 1uuh.pdb AB;1208 1uur.pdb AB;1209 1uv7.pdb AB;1210 1uw1.pdb AB;1211 1uwk.pdb AB;1212 1uww.pdb AB;1213 1ux4.pdb AB;1214 1uyt_1.pdb AB;1215 1uz2.pdb XA;1216 1uzb.pdb AB;1217 1v00_1.pdb AC;1218 1v05.pdb AB;1219 1v0d.pdb AB;1220 1v13.pdb AB;1221 1v1p.pdb AB;1222 1v25.pdb AB;1223 1v2z.pdb AB;1224 1v3e.pdb AB;1225 1v3v.pdb AB;1226 1v4e.pdb AB;1227 1v4v.pdb AB;1228 1v58_2.pdb AB;1229 1v59.pdb AB;1230 1v5v.pdb AB;1231 1v6z.pdb AB;1232 1v70.pdb AB;1233 1v71.pdb AB;1234 1v7c_1.pdb AB;1235 1v7l.pdb AB;1236 1v7o.pdb AB;1237 1v7w.pdb AB;1238 1v84.pdb AB;1239 1v8c_1.pdb AB;1240 1v8h.pdb AB;1241 1v96.pdb AB;1242 1v97.pdb AB;1243 1v9y.pdb AB;1244 1vbk.pdb AB;1245 1vc1.pdb AB;1246 1vch_3.pdb AE;1247 1vd6.pdb AB;1248 1vdw.pdb AB;1249 1ve1.pdb AB;1250 1ve2.pdb AB;1251 1ve3_1.pdb AB;1252 1ve9.pdb AB;1253 1vef.pdb AB;1254 1vfp.pdb AB;1255 1vgy.pdb AB;1256 1vh4.pdb AB;1257 1vh5.pdb AB;1258 1vh6.pdb AB;1259 1vhq.pdb AB;1260 1vhx.pdb AB;1261 1vhz.pdb AB;1262 1vi0.pdb AB;1263 1vi2.pdb AB;1264 1vi6_1.pdb AB;1265 1via.pdb AB;1266 1vio.pdb AB;1267 1viu_1.pdb AB;1268 1viv.pdb AB;1269 1vj2.pdb AB;1270 1vjg.pdb AB;1271 1vjh.pdb AB;1272 1vjl.pdb AB;1273 1vjo.pdb AB;1274 1vjq.pdb AB;1275 1vk6.pdb AB;1276 1vka.pdb AB;1277 1vkd_2.pdb CD;1278 1vkh.pdb AB;1279 1vki.pdb AB;1280 1vkp.pdb AB;1281 1vky.pdb AB;1282 1vl4.pdb AB;1283 1vla_2.pdb CD;1284 1vlj.pdb AB;1285 1vlr.pdb AB;1286 1vls.pdb AB;1287 1vlu_2.pdb AB;1288 1vme.pdb AB;1289 1vmg.pdb AB;1290 1vp4.pdb AB;1291 1vp6.pdb AC;1292 1vpb.pdb AB;1293 1vpk.pdb AB;1294 1vpz.pdb AB;1295 1vq0.pdb AB;1296 1vqt.pdb AB;1297 1vqv.pdb AB;1298 1vr0_1.pdb AB;1299 1vr7.pdb AB;1300 1vrb_2.pdb CD;1301 1vrw.pdb AB;1302 1vsc.pdb AB;1303 1vsg.pdb AB;1304 1vz0_4.pdb EF;1305 1vz8_1.pdb AB;1306 1vzi.pdb AB;1307 1w07.pdb AB;1308 1w23.pdb AB;1309 1w25.pdb AB;1310 1w2i.pdb AB;1311 1w2y.pdb AB;1312 1w3b.pdb AB;1313 1w3o.pdb AB;1314 1w53.pdb AB;1315 1w5r.pdb AB;1316 1w68.pdb AB;1317 1w6g.pdb AB;1318 1w8i.pdb AB;1319 1w9c.pdb AB;1320 1wc1_2.pdb BC;1321 1wc9.pdb AB;1322 1wet.pdb AD;1323 1whi.pdb AB;1324 1who.pdb AB;1325 1wiw.pdb AB;1326 1wjg.pdb AB;1327 1wlg.pdb AB;1328 1wlh.pdb AB;1329 1wmz_2.pdb CD;1330 1wpn.pdb AB;1331 1wqw.pdb AB;1332 1wr8.pdb AB;1333 1wtd.pdb AB;1334 1wu7.pdb AB;1335 1wuu_2.pdb CD;1336 1wv2.pdb AB;1337 1wv8.pdb AB;1338 1wvf.pdb AB;1339 1ww1.pdb AB;1340 1wwr_2.pdb CD;1341 1wws_4.pdb GH;1342 1wxx_1.pdb AB;1343 1wy5.pdb AB;1344 1wyz_1.pdb AC;1345 1wz3.pdb AB;1346 1wzd.pdb AB;1347 1x1z.pdb AB;1348 1x2i.pdb AB;1349 1x3m.pdb AB;1350 1x6m_2.pdb BC;1351 1x6v.pdb BA;1352 1x77.pdb AB;1353 1x7d.pdb AB;1354 1x82.pdb AB;1355 1x8z_2.pdb BC;1356 1x92.pdb AB;1357 1x9z.pdb AB;1358 1xb4_1.pdb AB;1359 1xbr.pdb AB;1360 1xcv.pdb AB;1361 1xd7.pdb AB;1362 1xdl_4.pdb YZ;1363 1xdp.pdb AB;1364 1xds.pdb AB;1365 1xe7_2.pdb AC;1366 1xff.pdb AB;1367 1xfs.pdb AB;1368 1xg7.pdb AB;1369 1xgs.pdb AB;1370 1xhk.pdb AB;1371 1xhn_1.pdb AB;1372 1xi3.pdb AB;1373 1xjl.pdb AB;1374 1xk7_1.pdb AB;1375 1xku.pdb AB;1376 1xkz_2.pdb CD;1377 1xma.pdb AB;1378 1xnf.pdb AB;1379 1xnx.pdb AB;1380 1xpc.pdb AB;1381 1xpm_1.pdb AB;1382 1xqa.pdb AB;1383 1xqb.pdb AB;1384 1xrh_4.pdb GH;1385 1xrk.pdb AB;1386 1xs0_1.pdb AB;1387 1xsv.pdb AB;1388 1xto.pdb AB;1389 1xub.pdb AB;1390 1xuu.pdb AB;1391 1xuv_2.pdb AB;1392 1xv2_1.pdb AB;1393 1xv8.pdb AB;1394 1xvh.pdb AB;1395 1xvi.pdb AB;1396 1xvs.pdb AB;1397 1xw8.pdb AB;1398 1xwm.pdb AB;1399 1xx6.pdb AB;1400 1xxu_1.pdb AC;1401 1xzw.pdb AB;1402 1y0b_2.pdb CD;1403 1y0g_1.pdb AB;1404 1y0h.pdb AB;1405 1y0u.pdb AB;1406 1y0z.pdb AB;1407 1y2o.pdb AB;1408 1y4j.pdb AB;1409 1y5h.pdb AB;1410 1y5m.pdb AB;1411 1y6v.pdb AB;1412 1y6x.pdb AB;1413 1y71.pdb AB;1414 1y7m.pdb AB;1415 1y7p_1.pdb AC;1416 1y7t.pdb AB;1417 1y88.pdb AB;1418 1y97.pdb AB;1419 1y9b.pdb AB;1420 1y9w.pdb AB;1421 1yav.pdb AB;1422 1yb2.pdb AB;1423 1yb5.pdb AB;1424 1ybe.pdb AB;1425 1ycc.pdb AB;1426 1ycg_1.pdb AB;1427 1ydw.pdb AB;1428 1yem.pdb AB;1429 1yer.pdb AB;1430 1yf5.pdb LA;1431 1yfu.pdb AB;1432 1yg2.pdb AB;1433 1yhc.pdb AB;1434 1yiz.pdb AB;1435 1yj5_1.pdb AB;1436 1yla.pdb AB;1437 1yln.pdb AB;1438 1ylq.pdb AB;1439 1ylx.pdb AB;1440 1ynf_2.pdb BD;1441 1yoa.pdb AB;1442 1yoz.pdb AB;1443 1ypq.pdb AB;1444 1ypt.pdb AB;1445 1yr0_2.pdb CD;1446 1yrb.pdb AB;1447 1yre_2.pdb CD;1448 1yrx_2.pdb AC;1449 1ys7.pdb AB;1450 1ysj.pdb AB;1451 1yta_1.pdb AB;1452 1yuz.pdb AB;1453 1yv9.pdb AB;1454 1yw6.pdb AB;1455 1ywq.pdb AB;1456 1yz3.pdb AB;1457 1z05.pdb AB;1458 1z0p.pdb AB;1459 1z24.pdb AB;1460 1z2l.pdb AB;1461 1z45.pdb AB;1462 1z5b.pdb AB;1463 1z63.pdb AB;1464 1z6b_1.pdb AB;1465 1z72.pdb AB;1466 1z84.pdb AB;1467 1z9h_1.pdb AB;1468 1zbo.pdb AB;1469 1zbr.pdb AB;1470 1zbs.pdb AB;1471 1zc6.pdb AB;1472 1zcs.pdb AB;1473 1zd3.pdb AB;1474 1zed.pdb AB;1475 1zee.pdb AB;1476 1zh8.pdb AB;1477 1zii.pdb AB;1478 1zjc.pdb AB;1479 1zk8.pdb AB;1480 1zkd.pdb AB;1481 1zke_3.pdb DE;1482 1zkp_1.pdb AC;1483 1zm1.pdb AB;1484 1zmb_3.pdb EF;1485 1zme.pdb CD;1486 1zno.pdb AB;1487 1znp_1.pdb AB;1488 1zp6.pdb AB;1489 1zps.pdb AB;1490 1zq9.pdb AB;1491 1zro.pdb AB;1492 1zrs.pdb AB;1493 1zso.pdb AB;1494 1ztd.pdb AB;1495 1zup.pdb AB;1496 1zva.pdb AB;1497 1zvp_1.pdb AD;1498 1zww.pdb AB;1499 1zyb.pdb AB;1500 1zym.pdb AB;1501 256b.pdb AB;1502 2a15.pdb AB;1503 2a26_1.pdb AB;1504 2a2j.pdb AB;1505 2a33.pdb AB;1506 2a35.pdb AB;1507 2a3q.pdb AB;1508 2a4x.pdb AB;1509 2a5l.pdb AB;1510 2a61_1.pdb AB;1511 2a6a.pdb AB;1512 2a6c_1.pdb AB;1513 2a84.pdb AB;1514 2a99.pdb AB;1515 2a9s.pdb AB;1516 2a9u.pdb AB;1517 2aa4.pdb AB;1518 2ab0.pdb AB;1519 2aca.pdb AB;1520 2ae2.pdb AB;1521 2aee.pdb AB;1522 2aeu.pdb AB;1523 2af4.pdb CD;1524 2aib.pdb AB;1525 2aj7.pdb AB;1526 2ajr.pdb AB;1527 2ako_1.pdb AB;1528 2akz.pdb AB;1529 2amx.pdb AB;1530 2aq6.pdb AB;1531 2arc.pdb AB;1532 2asf.pdb AB;1533 2aua.pdb AB;1534 2auw.pdb AB;1535 2avd.pdb AB;1536 2avn.pdb AB;1537 2axo.pdb AB;1538 2axp.pdb AB;1539 2axy_1.pdb AB;1540 2ayl.pdb AB;1541 2ayu.pdb AB;1542 2az0.pdb AB;1543 2b06.pdb AB;1544 2b0a.pdb AB;1545 2b0c.pdb AB;1546 2b0j.pdb AB;1547 2b1y.pdb AB;1548 2b3n.pdb AB;1549 2b48.pdb AB;1550 2b5a_2.pdb CD;1551 2b5g.pdb AB;1552 2b67_2.pdb CD;1553 2b69.pdb AB;1554 2b7o.pdb AB;1555 2b8m.pdb AB;1556 2b9c.pdb AB;1557 2bas.pdb AB;1558 2bay_2.pdb BC;1559 2bb3_1.pdb AB;1560 2bdr.pdb AB;1561 2bdt.pdb AB;1562 2be3.pdb AB;1563 2bf9.pdb AB;1564 2bgc_3.pdb FG;1565 2bgw.pdb AB;1566 2bgx.pdb AB;1567 2bif.pdb AB;1568 2bko.pdb AB;1569 2bkw.pdb AB;1570 2bll.pdb AB;1571 2bm5.pdb AB;1572 2bnk.pdb AB;1573 2bnx.pdb AB;1574 2bop.pdb AD;1575 2bpl_2.pdb BC;1576 2bz1.pdb AB;1577 2c2a.pdb AB;1578 2c2i.pdb AB;1579 2c42.pdb AB;1580 2c4j_1.pdb AB;1581 2c4v.pdb AB;1582 2c5a.pdb AB;1583 2c62.pdb AB;1584 2c9l.pdb YZ;1585 2c9v.pdb AF;1586 2ca1.pdb AB;1587 2car.pdb AB;1588 2cb5.pdb AB;1589 2cc0.pdb AB;1590 2cg6.pdb AB;1591 2ch5_1.pdb AB;1592 2ch7.pdb AB;1593 2cme_4.pdb HG;1594 2cn1.pdb AB;1595 2cn4.pdb AB;1596 2co3.pdb AB;1597 2cpg_1.pdb AB;1598 2csu.pdb AB;1599 2ctz.pdb AB;1600 2cu6.pdb AB;1601 2cvd_2.pdb BC;1602 2cwk.pdb AB;1603 2cwz_2.pdb CD;1604 2cxk_2.pdb CD;1605 2cy5.pdb AB;1606 2cy9.pdb AB;1607 2cye_2.pdb BD;1608 2cyy.pdb AB;1609 2d13_2.pdb BD;1610 2d4z.pdb AB;1611 2d6y.pdb AB;1612 2d8d.pdb AB;1613 2dek.pdb AB;1614 2dj5.pdb AB;1615 2dp9.pdb AB;1616 2dpi.pdb AD;1617 2ds5.pdb AB;1618 2dt5.pdb AB;1619 2dtr.pdb AB;1620 2dw6_1.pdb AB;1621 2e2c.pdb AB;1622 2e7s_4.pdb GH;1623 2ejn.pdb AB;1624 2esr.pdb AB;1625 2ess.pdb AB;1626 2euc.pdb AB;1627 2eul_1.pdb AB;1628 2evr.pdb AB;1629 2ewn.pdb AB;1630 2f02.pdb AB;1631 2f1f.pdb AB;1632 2f1k_1.pdb AB;1633 2f1l.pdb AB;1634 2f22.pdb AB;1635 2f2e.pdb AB;1636 2f3x.pdb AB;1637 2f48.pdb AB;1638 2f4l_2.pdb CD;1639 2f5g.pdb AB;1640 2f62.pdb AB;1641 2f6s.pdb AB;1642 2f6u.pdb AB;1643 2f7f.pdb AB;1644 2f8f.pdb AB;1645 2f96.pdb AB;1646 2f9h.pdb AB;1647 2f9w.pdb AB;1648 2fa1.pdb AB;1649 2fb1_1.pdb AB;1650 2fbh.pdb AB;1651 2fbi.pdb AB;1652 2fbk.pdb AB;1653 2fbl.pdb AB;1654 2fbn.pdb AB;1655 2fca.pdb AB;1656 2fd5.pdb AB;1657 2fdo.pdb BA;1658 2fe1.pdb AB;1659 2fex_1.pdb AB;1660 2ffc.pdb AB;1661 2ffe.pdb AB;1662 2ffg.pdb AB;1663 2ffs.pdb AB;1664 2fhq.pdb AB;1665 2fiq_1.pdb AB;1666 2fit.pdb AB;1667 2fiu.pdb AB;1668 2fl4.pdb AB;1669 2fml.pdb AB;1670 2fmt.pdb AB;1671 2fn0.pdb AB;1672 2fno_1.pdb AB;1673 2fnu.pdb AB;1674 2fo7.pdb AB;1675 2fp1.pdb AB;1676 2fpn.pdb AB;1677 2fpr.pdb AB;1678 2frh.pdb AB;1679 2fsw.pdb AB;1680 2ftr.pdb AB;1681 2fue.pdb AB;1682 2fur.pdb AB;1683 2fyi_3.pdb AD;1684 2fzf.pdb AB;1685 2fzt.pdb AB;1686 2g0t.pdb AB;1687 2g28.pdb AB;1688 2g39.pdb AB;1689 2g3a.pdb AB;1690 2g3p.pdb AB;1691 2g40.pdb AB;1692 2g5c_2.pdb BD;1693 2g63_1.pdb AB;1694 2g6t.pdb AB;1695 2g6w.pdb AB;1696 2g7g.pdb AB;1697 2g7l.pdb AB;1698 2g7s.pdb AB;1699 2g8l.pdb AB;1700 2g8o.pdb AB;1701 2ga1.pdb AB;1702 2gan.pdb AB;1703 2gau.pdb AB;1704 2gb3_1.pdb AB;1705 2gbo.pdb AB;1706 2gci_1.pdb AB;1707 2gcq.pdb AB;1708 2gec.pdb AB;1709 2gen.pdb AB;1710 2gh1.pdb AB;1711 2ghr.pdb AB;1712 2ghv.pdb EC;1713 2gi3.pdb AB;1714 2gib.pdb AB;1715 2giy.pdb AB;1716 2gj4.pdb AB;1717 2gj8_2.pdb CD;1718 2glz.pdb AB;1719 2gtd_3.pdb EF;1720 2gud.pdb AB;1721 2gup.pdb AB;1722 2gv8.pdb AB;1723 2gwg.pdb AB;1724 2gx9.pdb AB;1725 2gyq.pdb AB;1726 2gys.pdb AB;1727 2gz1.pdb AB;1728 2h2n.pdb AB;1729 2h9d_2.pdb CD;1730 2ha2.pdb AB;1731 2hbo.pdb AB;1732 2hbv.pdb AB;1733 2hck.pdb AB;1734 2hft.pdb AB;1735 2hhf.pdb AB;1736 2hhm.pdb AB;1737 2hiq.pdb AB;1738 2hjs.pdb AB;1739 2hlj.pdb AB;1740 2hmp.pdb AB;1741 2hoe.pdb AB;1742 2hox_1.pdb AB;1743 2hq9.pdb AB;1744 2hqv.pdb AB;1745 2hqx.pdb AB;1746 2hrc.pdb AB;1747 2hu7.pdb AB;1748 2hxv.pdb AB;1749 2hyd.pdb AB;1750 2hzb_2.pdb CD;1751 2i02.pdb AB;1752 2i3o_2.pdb CD;1753 2i4j.pdb AB;1754 2i7r.pdb AB;1755 2icy_1.pdb AB;1756 2id3.pdb AB;1757 2iid_2.pdb BD;1758 2iim.pdb AB;1759 2iiz.pdb AB;1760 2ijd_1.pdb 1A;1761 2ilk.pdb AB;1762 2io8.pdb AB;1763 2ipr.pdb AB;1764 2iqq.pdb AB;1765 2it9_1.pdb AB;1766 2iuw.pdb AB;1767 2ixp_2.pdb BD;1768 2j0n.pdb AB;1769 2j0w.pdb AB;1770 2j6b.pdb AB;1771 2jhf.pdb AB;1772 2msb.pdb AB;1773 2nac.pdb AB;1774 2nlv.pdb AB;1775 2nml.pdb AB;1776 2nmz.pdb AB;1777 2nrf.pdb AB;1778 2nrh.pdb AB;1779 2nul.pdb AB;1780 2nvn.pdb AB;1781 2nyc.pdb AB;1782 2nyr.pdb AB;1783 2o99_1.pdb AD;1784 2odk_1.pdb AB;1785 2or4.pdb AB;1786 2otx.pdb AB;1787 2p8i_1.pdb AB;1788 2paq.pdb AB;1789 2pg2.pdb AB;1790 2pgd.pdb AB;1791 2pgi.pdb AB;1792 2phn.pdb AB;1793 2pk8.pdb AB;1794 2q3m.pdb AB;1795 2q3t.pdb AB;1796 2q47.pdb AB;1797 2q48.pdb AB;1798 2q4n.pdb AB;1799 2sas.pdb AB;1800 2scp.pdb AB;1801 2shk.pdb AB;1802 2spc.pdb AB;1803 2tgi.pdb AB;1804 2vhb.pdb AB;1805 2vsg.pdb AB;1806 2wea.pdb AB;1807 3bam.pdb AB;1808 3cd4.pdb AB;1809 3cro.pdb LR;1810 3dap.pdb AB;1811 3dni.pdb AB;1812 3eip.pdb AB;1813 3grs.pdb AB;1814 3hts.pdb BD;1815 3il8.pdb AB;1816 3ink.pdb CD;1817 3lyn.pdb AB;1818 3mag.pdb AB;1819 3pvi.pdb AB;1820 3sdh.pdb AB;1821 3ssi.pdb AB;1822 3vub.pdb AB;1823 4ake.pdb AB;1824 4cpv.pdb AB;1825 4dfr.pdb AB;1826 4hb1.pdb AB;1827 4mt2.pdb AB;1828 4ovo.pdb AB;1829 4pro_1.pdb AB;1830 5csm.pdb AB;1831 5hpg.pdb AB;1832 5p21.pdb AB;1833 5rub.pdb AB;1834 6tmn.pdb EA;1835 7odc.pdb AB;1836 9gaf.pdb AC;1837 9wga_1.pdb AB;

Hetero-dimers

1 1a22.pdb AB;2 1a2x.pdb AB;3 1a9n_2.pdb CD;4 1acb.pdb EI;5 1ais.pdb AB;6 1aro.pdb PL;7 1as4.pdb AB;8 1aui.pdb AB;9 1ava_2.pdb BD;10 1avf_2.pdb QJ;11 1avw.pdb AB;12 1awc.pdb AB;13 1ay7.pdb AB;14 1b0n.pdb AB;15 1b34.pdb AB;16 1b6c_3.pdb EF;17 1bh9.pdb AB;18 1blx.pdb AB;19 1bmq.pdb AB;20 1bpl.pdb AB;21 1bqp_1.pdb AB;22 1br1_1.pdb AB;23 1buh.pdb AB;24 1bun.pdb AB;25 1bvn.pdb PT;26 1bzq_2.pdb BN;27 1c1y.pdb AB;28 1c5x.pdb BA;29 1cf7.pdb AB;30 1ci6.pdb BA;31 1cka.pdb AB;32 1clv.pdb AI;33 1cmi_2.pdb BD;34 1cpb.pdb AB;35 1cse.pdb EI;36 1cxz.pdb AB;37 1d2z_2.pdb CD;38 1d3b_2.pdb CD;39 1d4t.pdb AB;40 1d4x.pdb AG;41 1dce_2.pdb CD;42 1dev_1.pdb AB;43 1dj7.pdb AB;44 1dkf.pdb AB;45 1dn1.pdb AB;46 1dpj.pdb AB;47 1ds6.pdb AB;48 1dtd.pdb AB;49 1dy9_1.pdb AC;50 1dzb_1.pdb AX;51 1e44.pdb AB;52 1e50_1.pdb BA;53 1e96.pdb AB;54 1eay_1.pdb AC;55 1eg4.pdb AP;56 1egp.pdb AB;57 1eja.pdb AB;58 1elr.pdb AB;59 1elw_1.pdb AC;60 1em8_2.pdb CD;61 1emu.pdb AB;62 1euv.pdb AB;63 1ewy_1.pdb AC;64 1f0c.pdb AB;65 1f2t.pdb AB;66 1f34.pdb AB;67 1f3u_3.pdb EF;68 1f3v.pdb AB;69 1f45.pdb AB;70 1f59_1.pdb AC;71 1f5q_2.pdb CD;72 1f60.pdb AB;73 1fc2.pdb CD;74 1fcd_1.pdb AC;75 1fle.pdb EI;76 1fm0.pdb DE;77 1foe_2.pdb CD;78 1fq1.pdb AB;79 1fqk_2.pdb CD;80 1fqv_3.pdb EF;81 1fr2.pdb AB;82 1fs0.pdb EG;83 1fyh_2.pdb DE;84 1g0y.pdb RI;85 1g4u.pdb SR;86 1g6v.pdb AK;87 1g73_1.pdb AC;88 1g8k_3.pdb EF;89 1gc1_1.pdb GC;90 1gh6.pdb AB;91 1gk9.pdb AB;92 1gka.pdb AB;93 1gl1_1.pdb AI;94 1gl4.pdb AB;95 1go3_1.pdb EF;96 1gxc_1.pdb AB;97 1gxd_1.pdb AC;98 1gzh_1.pdb AB;99 1gzs_2.pdb CD;100 1h1v.pdb AG;101 1h32.pdb AB;102 1h3o_1.pdb AB;103 1h6k_2.pdb BY;104 1h9h.pdb EI;105 1hcn.pdb AB;106 1hdm.pdb AB;107 1he1_1.pdb AC;108 1he8.pdb AB;109 1hfe_2.pdb TM;110 1hr6_3.pdb EF;111 1hx1.pdb AB;112 1hxm_3.pdb EF;113 1i2m_2.pdb CD;114 1i31.pdb AP;115 1i8l_2.pdb BD;116 1iar.pdb AB;117 1ibr_2.pdb CD;118 1ihj_1.pdb AD;119 1iil_1.pdb AE;120 1itb.pdb AB;121 1ixs.pdb AB;122 1izn_1.pdb AB;123 1j2j.pdb AB;124 1jat.pdb AB;125 1jb7.pdb AB;126 1jdh.pdb AB;127 1jeq.pdb AB;128 1jey.pdb AB;129 1jiw.pdb PI;130 1jk9_2.pdb CD;131 1jkg.pdb AB;132 1jma.pdb AB;133 1jqj_1.pdb AC;134 1jql.pdb AB;135 1jrr.pdb AP;136 1jsd.pdb AB;137 1jtd.pdb AB;138 1jv2.pdb AB;139 1k8r.pdb AB;140 1k93_2.pdb BE;141 1ka9.pdb HF;142 1kfu.pdb LS;143 1kgc.pdb DE;144 1ki1_2.pdb CD;145 1klf_3.pdb EF;146 1kmh.pdb AB;147 1kps_2.pdb CD;148 1krl_2.pdb DC;149 1ksh.pdb AB;150 1ktp.pdb AB;151 1ku6.pdb AB;152 1kvd_2.pdb CD;153 1kxv_1.pdb AC;154 1kyf.pdb AP;155 1l0a.pdb AB;156 1l4d.pdb AB;157 1l6x.pdb AB;158 1lb6.pdb AB;159 1lck.pdb AB;160 1ldj.pdb AB;161 1lfd_1.pdb AB;162 1lpb.pdb AB;163 1lqv_2.pdb BD;164 1lsh.pdb AB;165 1luc.pdb AB;166 1lvb_1.pdb AC;167 1lyw_2.pdb CD;168 1m2t.pdb AB;169 1m2v.pdb AB;170 1m9x_2.pdb BC;171 1ma9.pdb AB;172 1mbx_2.pdb BD;173 1mhm.pdb BA;174 1mhw_1.pdb AC;175 1mju.pdb LH;176 1mq8_1.pdb AB;177 1mqk.pdb LH;178 1mr1_2.pdb BC;179 1mtp.pdb AB;180 1mvf_1.pdb AE;181 1n0l_1.pdb AB;182 1n0w.pdb AB;183 1n12_2.pdb CD;184 1n1j.pdb AB;185 1nbf_2.pdb BC;186 1nf3_1.pdb AC;187 1ngm_2.pdb EF;188 1nkp_1.pdb AB;189 1nln.pdb AB;190 1nlt.pdb AB;191 1nme.pdb AB;192 1nmu_1.pdb AB;193 1npe.pdb AB;194 1nq7.pdb AB;195 1nql.pdb AB;196 1nrj.pdb AB;197 1ntv.pdb AB;198 1nu9_2.pdb DF;199 1nw9.pdb AB;200 1o5e.pdb LH;201 1o7n.pdb AB;202 1o94_3.pdb EF;203 1o97.pdb CD;204 1oai.pdb AB;205 1oc0.pdb AB;206 1oe9.pdb AB;207 1oey_4.pdb DM;208 1oj5.pdb AB;209 1ols.pdb AB;210 1oo0.pdb AB;211 1op9.pdb AB;212 1oph.pdb AB;213 1or0_2.pdb CD;214 1or7_2.pdb BF;215 1owf.pdb AB;216 1oxk_1.pdb AB;217 1p16_1.pdb AC;218 1p2j.pdb AI;219 1p5v.pdb AB;220 1p6a.pdb AB;221 1pcx.pdb AB;222 1pdk.pdb AB;223 1pfb.pdb AB;224 1pfx.pdb CL;225 1pid_1.pdb AB;226 1pk1_1.pdb AB;227 1pqz.pdb AB;228 1puf.pdb AB;229 1pvh_1.pdb AB;230 1pxv_1.pdb AC;231 1q40_2.pdb CD;232 1q7l_2.pdb CD;233 1qa9_1.pdb BA;234 1qav.pdb AB;235 1qbk.pdb BC;236 1qd6_2.pdb BD;237 1qge.pdb DE;238 1qtx.pdb AB;239 1r0r.pdb EI;240 1r17_2.pdb BD;241 1r1k.pdb DA;242 1r27_1.pdb AB;243 1r8o.pdb AB;244 1r8s.pdb AE;245 1rdq.pdb EI;246 1req_1.pdb AB;247 1rj9.pdb AB;248 1rke.pdb AB;249 1rp3_1.pdb AB;250 1rrp_1.pdb AB;251 1s1q_2.pdb CD;252 1s6v_1.pdb AB;253 1s70.pdb AB;254 1scj.pdb AB;255 1sdd.pdb AB;256 1sdx.pdb AE;257 1sgh.pdb AB;258 1sgp.pdb EI;259 1shw.pdb AB;260 1shy.pdb AB;261 1spp.pdb BA;262 1sq0.pdb AB;263 1sq2.pdb LN;264 1sqk.pdb AB;265 1srq_2.pdb CD;266 1stf.pdb EI;267 1sv0_2.pdb BD;268 1svd.pdb AM;269 1svx.pdb AB;270 1syx_1.pdb AB;271 1t0h.pdb AB;272 1t15.pdb AB;273 1t3l.pdb AB;274 1t6b.pdb XY;275 1t6g_2.pdb BD;276 1t7r.pdb AB;277 1ta3.pdb AB;278 1tdq.pdb BA;279 1te1.pdb AB;280 1tf0.pdb AB;281 1tgs.pdb ZI;282 1tk5.pdb AB;283 1tmq.pdb AB;284 1tnr.pdb AR;285 1toq_3.pdb EF;286 1tqy_3.pdb EF;287 1tue_5.pdb KL;288 1twb_2.pdb BD;289 1tx4.pdb AB;290 1tye_3.pdb EF;291 1u00.pdb AP;292 1u0s.pdb YA;293 1u8c.pdb AB;294 1uad_2.pdb BD;295 1ugh.pdb EI;296 1umw_1.pdb AE;297 1unl_2.pdb BE;298 1us7.pdb AB;299 1usu.pdb AB;300 1uw4_1.pdb AB;301 1uzx.pdb AB;302 1v74.pdb AB;303 1vet.pdb AB;304 1vf6_1.pdb AC;305 1vg0.pdb AB;306 1vlf_1.pdb MN;307 1vrs_2.pdb BE;308 1vyt_1.pdb AE;309 1w9q_2.pdb BS;310 1wmh.pdb AB;311 1wq1.pdb RG;312 1wui.pdb SL;313 1x3z.pdb AB;314 1xb2.pdb AB;315 1xcg_1.pdb AB;316 1xd3_1.pdb AB;317 1xew.pdb XY;318 1xka.pdb LC;319 1xl3_2.pdb BD;320 1xoc.pdb AB;321 1xou.pdb AB;322 1xtg.pdb AB;323 1xzp.pdb AB;324 1y43.pdb AB;325 1y64.pdb AB;326 1yc5.pdb AB;327 1ycs.pdb AB;328 1ykh.pdb AB;329 1yro_1.pdb AB;330 1yvb.pdb AI;331 1yvh.pdb AB;332 1z0j.pdb AB;333 1z3e.pdb AB;334 1z5y.pdb DE;335 1z8u_2.pdb CD;336 1z92.pdb AB;337 1zbd.pdb AB;338 1zbx.pdb AB;339 1zc3_1.pdb AD;340 1zm9_2.pdb CD;341 1zun.pdb AB;342 2a1j.pdb AB;343 2a42.pdb AB;344 2a5t.pdb AB;345 2a9k.pdb AB;346 2aho.pdb AB;347 2ai4.pdb AB;348 2ajf_1.pdb AE;349 2apo.pdb AB;350 2as5_1.pdb NF;351 2axi.pdb AB;352 2ayo.pdb AB;353 2b59.pdb AB;354 2b5u_1.pdb AB;355 2bcg.pdb GY;356 2bex_2.pdb BD;357 2bf8.pdb AB;358 2bkr.pdb AB;359 2bp3_1.pdb AT;360 2btf.pdb AP;361 2buo.pdb AT;362 2bz6.pdb HL;363 2c1n_1.pdb AC;364 2c2l_1.pdb AE;365 2c35_4.pdb GH;366 2c5l_2.pdb BD;367 2c5w.pdb BA;368 2c7n_4.pdb GH;369 2ch4_1.pdb AW;370 2cho_2.pdb BD;371 2clo.pdb AB;372 2cnz.pdb AB;373 2cz1.pdb AB;374 2d7c_2.pdb BD;375 2dg5_1.pdb AB;376 2e2d.pdb AC;377 2f2c.pdb AB;378 2f2l.pdb AX;379 2f69.pdb AB;380 2f6m_2.pdb CD;381 2fcw.pdb AB;382 2ff4_1.pdb AE;383 2fge_2.pdb BE;384 2fh5.pdb AB;385 2fhz.pdb AB;386 2flu.pdb XP;387 2fp4.pdb AB;388 2ftx.pdb AB;389 2fu5_2.pdb BC;390 2fun_1.pdb AB;391 2g2u.pdb AB;392 2g30.pdb AP;393 2g38_1.pdb AB;394 2gfa_2.pdb BD;395 2gol_1.pdb AB;396 2gsm_1.pdb AB;397 2h26.pdb AB;398 2h6f.pdb AB;399 2hqs_4.pdb FG;400 2hth.pdb AB;401 2ibg_1.pdb AF;402 2ido_2.pdb CD;403 2iy5.pdb AB;404 2kin.pdb AB;405 2nnu.pdb AB;406 2npt_2.pdb BC;407 2nz8.pdb AB;408 2o25_1.pdb AD;409 2omy.pdb AB;410 2oq1.pdb AB;411 2oza.pdb AB;412 2p54.pdb AB;413 2pcd_3.pdb CO;414 2rln.pdb SE;415 3fap.pdb AB;416 3fru_2.pdb CD;417 3pro_1.pdb AC;418 3ygs.pdb CP;419 4cpa_1.pdb AI;420 4sgb.pdb EI;421 8pch.pdb AP;

Homo-trimers

1 1a0s.pdb PQR;2 1a12.pdb ABC;3 1a3d.pdb ABC;4 1af6.pdb ABC;5 1ahs.pdb ABC;6 1aly.pdb ABC;7 1am7.pdb ABC;8 1avq.pdb ABC;9 1avy_3.pdb ABC;10 1ax8.pdb ABC;11 1b47.pdb ABC;12 1b77.pdb ABC;13 1bh0.pdb ABC;14 1ble.pdb ABC;15 1bpo.pdb ABC;16 1brt.pdb ABC;17 1btg.pdb ABC;18 1bvp_2.pdb 456;19 1c09.pdb ABC;20 1c4t.pdb ABC;21 1c5e.pdb ABC;22 1c9k.pdb ABC;23 1cbq.pdb ABC;24 1cfm.pdb ABC;25 1cg6.pdb ABC;26 1coi.pdb ABC;27 1cos.pdb ABC;28 1cun.pdb ABC;29 1d5f.pdb ABC;30 1dcs.pdb ABC;31 1df4.pdb ABC;32 1dg6.pdb ABC;33 1dun.pdb ABC;34 1duv.pdb GHI;35 1e20.pdb ABC;36 1e3h.pdb ABC;37 1ed1.pdb ABC;38 1ef8.pdb ABC;39 1ek9.pdb ABC;40 1ekq_2.pdb ABC;41 1el6.pdb ABC;42 1ep5.pdb BAC;43 1euw.pdb ABC;44 1f3g.pdb ABC;45 1f44.pdb ADG;46 1f7l.pdb ABC;47 1fgj_1.pdb ABC;48 1fui_2.pdb DEF;49 1fxz.pdb ABC;50 1g5g_2.pdb DEF;51 1gbn.pdb ABC;52 1ge8.pdb ABC;53 1gyg_2.pdb ABC;54 1h7z.pdb ABC;55 1h9m_1.pdb ABC;56 1ha0.pdb ABC;57 1hg4_2.pdb BEF;58 1hlq.pdb ABC;59 1hm9_2.pdb ABC;60 1hne.pdb EAC;61 1htn.pdb ABC;62 1hx6.pdb ABC;63 1hxx.pdb ABC;64 1iby_1.pdb ABC;65 1icj.pdb ABC;66 1idp.pdb ABC;67 1ij2.pdb ABC;68 1iro.pdb ABC;69 1iv3_1.pdb ABC;70 1j5s.pdb ABC;71 1jcd.pdb ABC;72 1ji7.pdb ABC;73 1js0.pdb ABC;74 1js1.pdb XYZ;75 1jxz.pdb ABC;76 1k4m.pdb ABC;77 1k6f_2.pdb DEF;78 1khx.pdb ABC;79 1ki9.pdb ABC;80 1kke.pdb ABC;81 1knc.pdb ABC;82 1ko7_1.pdb ABC;83 1kwg.pdb ABC;84 1l1s.pdb ABC;85 1lkt_1.pdb ABC;86 1lua.pdb ABC;87 1m0l.pdb ABC;88 1m3y_1.pdb ABC;89 1m65.pdb ABC;90 1mg1.pdb ABC;91 1mr7_1.pdb ABC;92 1n2m_2.pdb DEF;93 1nkv.pdb ABC;94 1nmt.pdb ABC;95 1nog.pdb ABC;96 1nxj_2.pdb ABC;97 1o51.pdb ABC;98 1o6r.pdb ABC;99 1o6u.pdb ACE;100 1o7k.pdb ABC;101 1o91.pdb ABC;102 1ocy.pdb ABC;103 1od4.pdb ABC;104 1og6.pdb ABC;105 1oms.pdb ABC;106 1otg.pdb ABC;107 1oy9.pdb ABC;108 1p2z.pdb ABC;109 1p32.pdb ABC;110 1p5q.pdb ABC;111 1p9h.pdb ABC;112 1pf5.pdb ABC;113 1pm4.pdb ABC;114 1ppr.pdb MNO;115 1pwb.pdb ABC;116 1q14.pdb ABC;117 1qdn.pdb ABC;118 1qhd.pdb ABC;119 1qiu_2.pdb DEF;120 1qj8.pdb ABC;121 1qlm.pdb ABC;122 1qmu.pdb ABC;123 1qp1.pdb ABC;124 1qre.pdb ABC;125 1qu9.pdb ABC;126 1qvr.pdb ABC;127 1qwg.pdb ABC;128 1rer.pdb ABC;129 1rgx.pdb ABC;130 1rhg.pdb ABC;131 1rj8_2.pdb EGF;132 1rq0.pdb ABC;133 1rv1.pdb ABC;134 1rwt_2.pdb BFG;135 1rwz.pdb ABC;136 1s2e_2.pdb ABC;137 1s7m_2.pdb DEF;138 1s7o.pdb ABC;139 1sed.pdb ABC;140 1sg4.pdb ABC;141 1slq_1.pdb ABC;142 1stz.pdb ABC;143 1szo_1.pdb ABC;144 1tgg.pdb ABC;145 1tsr.pdb ABC;146 1tui.pdb ABC;147 1tul.pdb ABC;148 1tyx.pdb ABC;149 1u5h.pdb ABC;150 1u5x.pdb ABC;151 1u7g.pdb ABC;152 1uae.pdb ABC;153 1ufy.pdb ABC;154 1uku.pdb ABC;155 1urz_1.pdb ABC;156 1usq_2.pdb ABC;157 1uuy.pdb ABC;158 1uvj.pdb ABC;159 1uxa.pdb ABC;160 1uyj_2.pdb ABC;161 1v0e_2.pdb DEF;162 1v1i.pdb ABC;163 1v8d.pdb ABC;164 1vkj.pdb ABC;165 1vkm_2.pdb DEF;166 1vl0.pdb ABC;167 1vmh.pdb ABC;168 1w15.pdb ABC;169 1w58.pdb 1AB;170 1wbh.pdb ABC;171 1wm3.pdb ABC;172 1wp1_1.pdb ABC;173 1wud.pdb ABD;174 1ww9.pdb ABC;175 1wyy_2.pdb ABC;176 1xs1_1.pdb ABC;177 1y4m.pdb ABC;178 1yb0.pdb ABC;179 1ybf.pdb ABC;180 1yf9.pdb ABC;181 1ylf.pdb ABC;182 1ynb.pdb ABC;183 1yqf_1.pdb ABC;184 1ysr.pdb ABC;185 1yu0.pdb ABC;186 1yx1.pdb ABC;187 1zpv.pdb ABC;188 1zru.pdb ABC;189 1zvb.pdb ABC;190 2a5z.pdb ABC;191 2a7k_2.pdb DEF;192 2aal_1.pdb ABC;193 2ae8_1.pdb ABC;194 2aeb_2.pdb ABC;195 2arh.pdb ABC;196 2ayn.pdb ABC;197 2ba2.pdb ABC;198 2bi0.pdb ABC;199 2bw4.pdb ABC;200 2cav.pdb ABC;201 2cro.pdb ABC;202 2cz4.pdb ABC;203 2e2a.pdb ABC;204 2ebo.pdb ABC;205 2f0c.pdb ABC;206 2f5k_2.pdb BDF;207 2f6q.pdb ABC;208 2fb5.pdb ABC;209 2fef.pdb ABC;210 2fgq.pdb XAB;211 2fql.pdb ABC;212 2gdg.pdb ABC;213 2gfq.pdb ABC;214 2gr8_1.pdb ACD;215 2gvh.pdb ABC;216 2h6l.pdb ABC;217 2hew.pdb FAB;218 2hrr.pdb ABC;219 2hx0.pdb ABC;220 2ie7.pdb ABC;221 2igt.pdb ABC;222 2nww.pdb ABC;223 2p2l.pdb ABC;224 2por.pdb ABC;225 2q4i.pdb ABC;226 2tdt.pdb ABC;227 2tnf.pdb ABC;228 3cla.pdb ABC;229 3csu.pdb ABC;230 3pnp.pdb ABC;231 4bcl.pdb ABC;232 8prn.pdb ABC;

Hetero-trimers

1 1a02.pdb NFJ;2 1a2y.pdb ABC;3 1adq.pdb ALH;4 1bgx.pdb TLH;5 1cjt.pdb ABC;6 1cl7.pdb LHI;7 1cul.pdb ABC;8 1d3d.pdb ABH;9 1dee_2.pdb CDG;10 1e6j.pdb HLP;11 1egj.pdb LHA;12 1ept.pdb CAB;13 1ezx.pdb ACB;14 1fbv.pdb ACB;15 1fe8_3.pdb CJN;16 1fns.pdb LHA;17 1fqj_1.pdb ABC;18 1frt.pdb ACB;19 1fsk_4.pdb JKL;20 1g3n_1.pdb ABC;21 1gg6.pdb BCA;22 1got.pdb ABG;23 1gux.pdb ABE;24 1h0d.pdb ABC;25 1hia_1.pdb ABI;26 1icf_2.pdb CDJ;27 1ikn.pdb ACD;28 1iqd.pdb ABC;29 1j1d_2.pdb DEF;30 1jfi.pdb ABC;31 1jhl.pdb HAL;32 1jps.pdb LHT;33 1jrh.pdb LHI;34 1jsu.pdb ABC;35 1jwy.pdb ABC;36 1k5d_1.pdb ABC;37 1k5n.pdb ABC;38 1kig.pdb HIL;39 1kk8.pdb ABC;40 1lk3_2.pdb BMI;41 1ltx.pdb ABR;42 1lvm_1.pdb AEC;43 1m27.pdb ABC;44 1m63_1.pdb ABC;45 1m93.pdb ABC;46 1mf8.pdb ABC;47 1mhh_2.pdb CDF;48 1mhp_1.pdb AHL;49 1mmf_2.pdb LEM;50 1n4q_2.pdb CDN;51 1n8z.pdb ABC;52 1nb5_2.pdb BJR;53 1nby.pdb ABC;54 1nex_2.pdb CDF;55 1nl0.pdb LHG;56 1nmb.pdb NLH;57 1nsn.pdb LHS;58 1o5d.pdb LHT;59 1oaz_1.pdb AHL;60 1omw.pdb ABG;61 1ors.pdb ABC;62 1osp.pdb LHO;63 1p22.pdb ABC;64 1pby.pdb ABC;65 1pkq_1.pdb ABE;66 1q16.pdb ABC;67 1r4m_3.pdb EFK;68 1re3_1.pdb ABC;69 1rh5.pdb ABC;70 1rk8.pdb ABC;71 1rzh.pdb LMH;72 1s78_1.pdb ACD;73 1sr4.pdb ABC;74 1sy6.pdb LHA;75 1t08.pdb ABC;76 1tco.pdb ABC;77 1tmc.pdb ABC;78 1tqb.pdb ABC;79 1tt5_1.pdb ABE;80 1u6g.pdb ABC;81 1uvq.pdb ABC;82 1v7m_1.pdb LHV;83 1v7p.pdb ABC;84 1w36_2.pdb EFG;85 1wa5.pdb ABC;86 1wej.pdb LHF;87 1xhm.pdb ABC;88 1xkp.pdb ABC;89 1xme.pdb ABC;90 1za3_2.pdb RLH;91 1ze3.pdb CDH;92 2a2q.pdb LHT;93 2adf.pdb AHL;94 2aze.pdb ABC;95 2cch_1.pdb ABE;96 2ey4_1.pdb AEC;97 2f2a.pdb ABC;98 2f66_1.pdb ABC;99 2fnj.pdb ABC;100 2h9e.pdb HLC;101 2j88.pdb AHL;102 2jel.pdb LHP;103 2mys.pdb ABC;104 2nym_1.pdb ABC;105 2trc.pdb BGP;106 2vir.pdb ABC;107 4ubp.pdb ABC;

Homo-tetramers

1 1a0c.pdb ABCD;2 1a0j.pdb ABCD;3 1a17.pdb ABCD;4 1a2z.pdb ABCD;5 1a6j.pdb ABCD;6 1a79.pdb ABCD;7 1a7b.pdb BDAC;8 1abb.pdb ABCD;9 1agq.pdb ABCD;10 1aie.pdb ABCD;11 1an7.pdb ABCD;12 1at3.pdb ABCD;13 1aug.pdb ABCD;14 1auv.pdb ABCD;15 1av1.pdb ABCD;16 1ay9.pdb ABCD;17 1ayr.pdb ABCD;18 1b01.pdb ABGH;19 1b1z.pdb ABCD;20 1b25.pdb ABCD;21 1b2p.pdb ABCD;22 1b4w.pdb ABCD;23 1b5e.pdb ABCD;24 1b65_1.pdb ABCD;25 1b9b.pdb ABCD;26 1b9c.pdb ABCD;27 1bbp.pdb ABCD;28 1bdb.pdb ABCD;29 1bdf.pdb ABCD;30 1bhg.pdb ABCD;31 1bkb.pdb ABCD;32 1bsm.pdb ABCD;33 1bwn.pdb ABCD;34 1c2b.pdb ABCD;35 1c3c.pdb ABCD;36 1c3k.pdb ABCD;37 1c4p.pdb ABCD;38 1c7g.pdb ABCD;39 1c8b.pdb ABCD;40 1cbl.pdb ABCD;41 1cbm.pdb ABCD;42 1ce9.pdb ABCD;43 1cf2.pdb PROQ;44 1cfr.pdb ABCD;45 1cg2.pdb ABCD;46 1cjx.pdb ABCD;47 1cs1.pdb ABCD;48 1cuk.pdb ABCD;49 1cvi.pdb ABCD;50 1d1j.pdb ABCD;51 1d1s.pdb ABCD;52 1d1z.pdb ABCD;53 1d2e.pdb ABCD;54 1d7u.pdb ABCD;55 1d8l.pdb ABCD;56 1d8w.pdb ABCD;57 1dgf.pdb ABCD;58 1dir.pdb ABCD;59 1doh.pdb ABCD;60 1dq9.pdb ABCD;61 1dqi.pdb ABCD;62 1dru.pdb ABCD;63 1dxx.pdb ABCD;64 1dyo.pdb ABCD;65 1e2t_1.pdb ABCD;66 1e4e.pdb ABCD;67 1e5d.pdb ABCD;68 1e65.pdb ABCD;69 1eb8.pdb ABCD;70 1ec8.pdb ABCD;71 1ecf.pdb ABCD;72 1ecm.pdb ABCD;73 1eep_1.pdb ABCD;74 1eg1.pdb ACBD;75 1eg7.pdb ABCD;76 1eia.pdb ABCD;77 1ems.pdb ABCD;78 1epx.pdb ABCD;79 1eqr.pdb ABCD;80 1ev4.pdb ACDB;81 1ezj.pdb ABCD;82 1ezw.pdb ABCD;83 1f07.pdb ABCD;84 1f1x.pdb ABCD;85 1f2l.pdb ABCD;86 1f38.pdb ABCD;87 1f39.pdb ABCD;88 1f74.pdb ACBD;89 1f8e.pdb ABCD;90 1f8f.pdb ABCD;91 1f8m.pdb ABCD;92 1f9j.pdb ABCD;93 1fa2.pdb ABCD;94 1fe6.pdb ABCD;95 1fiu.pdb ABCD;96 1flo.pdb ABCD;97 1fmc.pdb ABCD;98 1fnu.pdb ABCD;99 1frp.pdb ABCD;100 1fsg.pdb ACBD;101 1ftr.pdb ABCD;102 1fua.pdb ABCD;103 1fuj.pdb ABCD;104 1fxo_2.pdb EFGH;105 1fzd_2.pdb EFGH;106 1g1j.pdb ABCD;107 1g2z.pdb ABCD;108 1g5y.pdb ABCD;109 1gg1.pdb ABCD;110 1ggx.pdb ABCD;111 1gjo.pdb ABCD;112 1gkj.pdb ABCD;113 1gkm.pdb ABCD;114 1gkr.pdb ABCD;115 1gmw.pdb ABCD;116 1gp9.pdb ABCD;117 1gpm.pdb ABCD;118 1gqg.pdb ABCD;119 1gr0.pdb ABCD;120 1gr7.pdb ABCD;121 1gtd.pdb ABCD;122 1gvf.pdb ABCD;123 1gy8.pdb ABCD;124 1gz5.pdb ABCD;125 1gz6.pdb ABCD;126 1gz7.pdb ABCD;127 1h21.pdb ABCD;128 1h3y.pdb ABCD;129 1h5q_3.pdb IJKL;130 1h6d_2.pdb EFGH;131 1h8g.pdb ABCD;132 1h8p.pdb ABCD;133 1h9x.pdb ABCD;134 1hcj_2.pdb ABCD;135 1hjx.pdb ABCD;136 1hvv.pdb ABCD;137 1hyh.pdb ABCD;138 1hyl.pdb ABCD;139 1i3d.pdb ABCD;140 1i6p.pdb ABCD;141 1ik6.pdb ABCD;142 1inl.pdb ABCD;143 1ith.pdb ABCD;144 1ixc.pdb ABCD;145 1j1j.pdb ABCD;146 1j2g.pdb ABCD;147 1j2r.pdb ABCD;148 1j3w.pdb ABCD;149 1j4n.pdb ABCD;150 1jaf.pdb ABCD;151 1jg8.pdb ABCD;152 1jkm.pdb ABCD;153 1jl5.pdb ABCD;154 1jof_1.pdb ABCD;155 1jpm.pdb ABCD;156 1jqn.pdb ABCD;157 1jqp.pdb ABCD;158 1jr7.pdb ABCD;159 1js2.pdb ABCD;160 1jvm.pdb ABCD;161 1jyl.pdb ABCD;162 1jz7.pdb ABCD;163 1k1e_3.pdb IJKL;164 1k1f_1.pdb ABCD;165 1k6y.pdb ABCD;166 1k7w.pdb ABCD;167 1k92.pdb ABCD;168 1kam.pdb ABCD;169 1kaw.pdb ABCD;170 1kbj.pdb ABCD;171 1klo.pdb ABCD;172 1kol.pdb ABCD;173 1kor.pdb ABCD;174 1l3a.pdb ABCD;175 1l3c.pdb ABCD;176 1l3i_2.pdb ABEF;177 1l5r.pdb ABCD;178 1l5s.pdb ABCD;179 1l7l.pdb ABCD;180 1ldd.pdb ABCD;181 1ldf.pdb ABCD;182 1li4.pdb ABCD;183 1lk5.pdb ABCD;184 1llu_1.pdb ABCD;185 1lo7.pdb ABCD;186 1lt8.pdb ABCD;187 1ltq.pdb ABCD;188 1lw7.pdb ABCD;189 1lxj.pdb ABCD;190 1m1l.pdb ABCD;191 1m3k.pdb ABCD;192 1m3w.pdb ABCD;193 1me8.pdb ABCD;194 1mjo.pdb ABCD;195 1mkm.pdb ABCD;196 1mou.pdb ABCD;197 1moy.pdb ABCD;198 1mp4.pdb ABCD;199 1mpx.pdb ABCD;200 1mpy.pdb ABCD;201 1msp.pdb ABCD;202 1mv5.pdb ABCD;203 1mvk_3.pdb IJKL;204 1mw5.pdb ABCD;205 1mxf.pdb ABCD;206 1n1q.pdb ABCD;207 1n7h.pdb ABCD;208 1n93.pdb XABC;209 1n9p.pdb ABCD;210 1naw.pdb ABCD;211 1nba.pdb ABCD;212 1nc7.pdb ABCD;213 1nfj.pdb ABCD;214 1nhk.pdb RLAB;215 1nls.pdb ABCD;216 1nm3.pdb ABCD;217 1nqk.pdb ABCD;218 1nsi.pdb ABCD;219 1nsw.pdb ABCD;220 1nul.pdb ABCD;221 1nuq.pdb ABCD;222 1nw2_2.pdb EFGH;223 1nyt.pdb ABCD;224 1o04_2.pdb EFGH;225 1o0s.pdb ABCD;226 1o1x.pdb ABCD;227 1o26.pdb ABCD;228 1o54.pdb ABCD;229 1o58.pdb ABCD;230 1o6e.pdb ABCD;231 1o6z.pdb ABCD;232 1o7j.pdb ABCD;233 1o7l.pdb ABCD;234 1o84.pdb ABCD;235 1o9l.pdb ABCD;236 1o9y.pdb ABCD;237 1oa8.pdb ABCD;238 1ocv.pdb ABCD;239 1oe0.pdb ABCD;240 1oez.pdb WXYZ;241 1oft.pdb ABCD;242 1oi0.pdb ABCD;243 1oih.pdb ABCD;244 1ojr.pdb ABCD;245 1oko.pdb ABCD;246 1olp.pdb ABCD;247 1on0.pdb ABCD;248 1ooz.pdb ABCD;249 1ope.pdb ABCD;250 1orr.pdb ABCD;251 1orv.pdb ABCD;252 1ou0.pdb ABCD;253 1ovm.pdb ABCD;254 1ovo_2.pdb ABCD;255 1oyn.pdb ABCD;256 1ozf.pdb ABCD;257 1ozh.pdb ABCD;258 1p1f.pdb ABCD;259 1p1j.pdb ABCD;260 1p5b.pdb ABCD;261 1p80.pdb ABCD;262 1p8j_2.pdb EFGH;263 1pbi.pdb ABCD;264 1pe1.pdb ABCD;265 1pff.pdb ABCD;266 1pj5.pdb ABCD;267 1pk8_1.pdb ABCD;268 1pl4.pdb ABCD;269 1pl8_2.pdb ABCD;270 1pqh.pdb ABCD;271 1pv1.pdb ABCD;272 1px8.pdb ABCD;273 1pyg.pdb ABCD;274 1pym.pdb ABCD;275 1q0a.pdb ABCD;276 1q15.pdb ACBD;277 1q3g_2.pdb EFGH;278 1q43_1.pdb ABCD;279 1q4s.pdb ABCD;280 1q4t.pdb ABCD;281 1q4u.pdb ABCD;282 1q5n.pdb ABCD;283 1q5y.pdb ABCD;284 1q8f.pdb ABCD;285 1qb4.pdb ABCD;286 1qfx.pdb ABCD;287 1qhx.pdb ABCD;288 1qki_1.pdb ABCD;289 1qm4.pdb ABCD;290 1qma.pdb ABCD;291 1qnn.pdb ABCD;292 1qrq.pdb ABCD;293 1qrz.pdb ABCD;294 1qsg_2.pdb EFGH;295 1qsm.pdb ABCD;296 1qvc.pdb ABCD;297 1qw2.pdb ABCD;298 1qwh.pdb ABCD;299 1qx8.pdb ABCD;300 1qyn.pdb ABCD;301 1rcu.pdb ABCD;302 1rec.pdb ABCD;303 1rfz.pdb ABCD;304 1rg9.pdb ABCD;305 1rh4.pdb ABCD;306 1ris.pdb ABCD;307 1rlz.pdb ABCD;308 1rmt.pdb ABCD;309 1ro7.pdb ABCD;310 1rtw.pdb ABCD;311 1ru0.pdb ABCD;312 1rv3.pdb ABCD;313 1rye.pdb ABCD;314 1ryi_2.pdb ABCD;315 1ryw_2.pdb EFGH;316 1s5a.pdb ABCD;317 1s5u_1.pdb ABCD;318 1s98.pdb ABCD;319 1sc6.pdb ABCD;320 1sff.pdb ABCD;321 1sjw.pdb ABCD;322 1snd.pdb ABCD;323 1su1.pdb ABCD;324 1suw.pdb ABCD;325 1svv.pdb ABCD;326 1sy7.pdb ABCD;327 1t0b_2.pdb EFGH;328 1t1d.pdb ABCD;329 1t2d.pdb ABCD;330 1t3n.pdb ABEF;331 1t3u.pdb ABCD;332 1tbu.pdb ABCD;333 1tk9.pdb ABCD;334 1tlf.pdb ABCD;335 1tlt.pdb ABCD;336 1tq8_2.pdb ABEF;337 1trr_1.pdb ABGH;338 1tvx.pdb ABCD;339 1tzj.pdb ABCD;340 1u1i.pdb ABCD;341 1u69.pdb ABCD;342 1u8v.pdb ABCD;343 1u9y.pdb ABCD;344 1ub3.pdb ABCD;345 1udd.pdb ABCD;346 1udr.pdb ABCD;347 1ue1.pdb ABCD;348 1uf5.pdb ABCD;349 1ufh.pdb ABCD;350 1uh5.pdb ABCD;351 1uhv.pdb ABCD;352 1uj2.pdb ABCD;353 1ujq.pdb ABCD;354 1uld_1.pdb DABC;355 1um0.pdb ABCD;356 1umy.pdb ABCD;357 1unk.pdb ABCD;358 1up7_1.pdb ABCD;359 1upa.pdb ABCD;360 1use.pdb ABCD;361 1usx.pdb ABCD;362 1utb.pdb ABCD;363 1uuj.pdb ABCD;364 1uwu.pdb ABCD;365 1uxt.pdb ABCD;366 1uzm.pdb ABCD;367 1v37.pdb ABCD;368 1v4g.pdb ABCD;369 1v5e.pdb ABCD;370 1v6t.pdb ABCD;371 1v72.pdb ABCD;372 1v8p_2.pdb EFGH;373 1v8q.pdb ABCD;374 1v9z.pdb ABCD;375 1vbo_1.pdb DABC;376 1vcn.pdb ABCD;377 1vco.pdb ABCD;378 1vdd.pdb ABCD;379 1vdk.pdb ABCD;380 1vh1.pdb ABCD;381 1vim.pdb ABCD;382 1vj0.pdb ABCD;383 1vjp.pdb ABCD;384 1vk8.pdb ABCD;385 1vkn.pdb ABCD;386 1vl6.pdb ABCD;387 1vp2.pdb ABCD;388 1vq3.pdb ABCD;389 1vr6.pdb ABCD;390 1vzy.pdb ABCD;391 1w0m_1.pdb ABCD;392 1w30.pdb ABCD;393 1w3i.pdb ABCD;394 1w6u.pdb ABCD;395 1w9y.pdb ABCD;396 1wiy.pdb ABCD;397 1wlu.pdb ABCD;398 1wn6.pdb ABCD;399 1wok.pdb ABCD;400 1wpw.pdb ABCD;401 1ws8.pdb ABCD;402 1wtl.pdb ABCD;403 1wty.pdb ABCD;404 1wvg.pdb ABCD;405 1wvl.pdb ABGH;406 1wwh.pdb ABCD;407 1wyi.pdb ABCD;408 1x99.pdb ABCD;409 1x9g.pdb ABCD;410 1xar.pdb ABCD;411 1xcf.pdb ABCD;412 1xdi.pdb ABCD;413 1xea.pdb ABCD;414 1xg5.pdb ABCD;415 1xi9.pdb ABCD;416 1xkl.pdb ABCD;417 1xl4.pdb ABCD;418 1xm3.pdb ABCD;419 1xo0.pdb ABGH;420 1xpj.pdb ABCD;421 1xpp.pdb ABCD;422 1xq4.pdb ABCD;423 1xtt.pdb ABCD;424 1xu9.pdb ABCD;425 1xva.pdb ABCD;426 1xwr.pdb ABCD;427 1y1a.pdb ABCD;428 1y1l.pdb ABCD;429 1y1o.pdb ABCD;430 1y7b.pdb ABCD;431 1y82.pdb ABCD;432 1y9i.pdb ABCD;433 1yak_2.pdb ABCD;434 1ybh.pdb ABCD;435 1ycy.pdb ABCD;436 1yde_2.pdb EFGH;437 1ydg_2.pdb EFGH;438 1yfz.pdb ABCD;439 1ygy.pdb ABCD;440 1ynr.pdb ABCD;441 1yp2.pdb ABCD;442 1yqh.pdb ABCD;443 1yvk.pdb ABCD;444 1yxb_2.pdb EFGH;445 1z0s.pdb ABCD;446 1z1b.pdb ABMN;447 1z41.pdb ABCD;448 1z7a_1.pdb ABCD;449 1z94_2.pdb ABEF;450 1z9w.pdb ABCD;451 1zai.pdb ABCD;452 1zfj.pdb ABCD;453 1zk4.pdb ABCD;454 1zmt.pdb ABCD;455 1zpd.pdb ABEF;456 1zq7.pdb ABCD;457 1zr4.pdb ABED;458 1ztc.pdb ABCD;459 2a2u.pdb ABCD;460 2a6n.pdb ABCD;461 2a7w_2.pdb EFGH;462 2aaf.pdb ABCD;463 2ac0.pdb ABCD;464 2ady.pdb ABGH;465 2ae6.pdb ABCD;466 2ag5.pdb ABCD;467 2ahu.pdb ABCD;468 2aio.pdb ABCD;469 2ark_2.pdb ABEF;470 2av9_2.pdb CHIK;471 2avp.pdb ABCD;472 2awi_2.pdb CDKL;473 2az5.pdb ABCD;474 2b0l_2.pdb ABCD;475 2b3z.pdb ABCD;476 2b5d.pdb XABC;477 2b82.pdb ABCD;478 2b8t.pdb ABCD;479 2bb2.pdb ABCD;480 2bde.pdb ABCD;481 2bhn_1.pdb ABCD;482 2bj7.pdb ABCD;483 2bnm.pdb ABCD;484 2bo4_1.pdb ABEF;485 2bwv.pdb ABCD;486 2c12_1.pdb ABCD;487 2c31.pdb ABCD;488 2c4n.pdb ABCD;489 2cay.pdb ABCD;490 2chh.pdb ABCD;491 2cm3.pdb ABCD;492 2cvz.pdb ABCD;493 2cx3.pdb ABCD;494 2d29.pdb ABCD;495 2d32.pdb ABCD;496 2d3e.pdb ABCD;497 2d45.pdb ABCD;498 2dft.pdb ABCD;499 2dvt.pdb ABCD;500 2eia.pdb ABCD;501 2esn.pdb ACBD;502 2ewo_3.pdb EHKL;503 2f01.pdb ABCD;504 2f06.pdb ABCD;505 2f36.pdb ABCD;506 2f3d.pdb ABCD;507 2f5v.pdb ABCD;508 2f6k.pdb ABCD;509 2f8a.pdb ABCD;510 2fn3.pdb ABCD;511 2fr5.pdb ABCD;512 2fs2.pdb ABCD;513 2ftw.pdb ABCD;514 2fuj.pdb ABCD;515 2g50_2.pdb EFGH;516 2g7o.pdb ABCD;517 2g82_1.pdb OPQR;518 2gf6_1.pdb ABCD;519 2ghp_2.pdb EFGH;520 2glk.pdb ABCD;521 2glt.pdb ABCD;522 2gm4.pdb ABLM;523 2gqv.pdb ABCD;524 2gus.pdb ABCD;525 2hcb.pdb ABCD;526 2hek.pdb ABCD;527 2hmf.pdb ABCD;528 2hx5.pdb ABCD;529 2i52_2.pdb ABEF;530 2iba.pdb ABCD;531 2idj.pdb ABCD;532 2ij9.pdb ABCD;533 2ixc.pdb ABCD;534 2mlt.pdb ABCD;535 2nlz.pdb ABCD;536 2ntk.pdb ABCD;537 2nuj.pdb ABCD;538 2nw8.pdb ABCD;539 2o23.pdb ABCD;540 2oat.pdb ABCD;541 2p06.pdb ABCD;542 2p3r_1.pdb ABEG;543 2plh.pdb ABCD;544 2pva.pdb ABCD;545 2q41.pdb ABCD;546 2rmc.pdb ACEG;547 2toh.pdb ABCD;548 3gpd.pdb RGAB;549 3mcg.pdb 12AB;550 3pfk.pdb ABCD;551 6ldh.pdb ABCD;552 6q21.pdb ABCD;553 7gpb.pdb ABCD;554 7pcy.pdb ABCD;

Hetero-tetramers

1 1ar1.pdb ABCD;2 1ca0_1.pdb BCDA;3 1cho.pdb GIFE;4 1dan.pdb LHTU;5 1eys.pdb CLMH;6 1fak.pdb LHTI;7 1g9m.pdb GCLH;8 1gl2.pdb ABCD;9 1hqr.pdb ABDC;10 1hxy.pdb ABDC;11 1im3_3.pdb ILJK;12 1jl4.pdb ABDC;13 1kb5.pdb ALHB;14 1kf6_2.pdb MNOP;15 1kg0.pdb ABCD;16 1klg.pdb ABDC;17 1l9b.pdb LMHC;18 1lm8.pdb BCVH;19 1m56_1.pdb ABCD;20 1n7s.pdb ABCD;21 1nek.pdb ABCD;22 1nfd_1.pdb ABEF;23 1nh2.pdb ABDC;24 1nvp.pdb ABDC;25 1p7q.pdb ADBC;26 1qhh.pdb ABCD;27 1qkz.pdb LHAP;28 1r5i_1.pdb ABDC;29 1riw.pdb ABCD;30 1rjl.pdb ACBD;31 1txv.pdb ABLH;32 1w63_1.pdb ABMS;33 1xiw_1.pdb ABCD;34 1ytf.pdb ABDC;35 2b5i.pdb ABCD;36 2bcj.pdb ABGQ;37 2gc7_2.pdb EFGH;38 2i5n.pdb CHLM;

Random set 1

1 12as.pdb B 1a0a.pdb B;2 1a12.pdb B 1a25.pdb B;3 1a2z.pdb B 1a64.pdb B;4 1aa7.pdb B 1ab8.pdb B;5 1adu.pdb B 1aih_1.pdb B;6 1aoh.pdb B 1apx_2.pdb D;7 1aro.pdb L 1au7.pdb B;8 1auo.pdb B 1avw.pdb B;9 1ayf.pdb B 1azw.pdb B;10 1b1z.pdb B 1b3t.pdb B;11 1b6u.pdb B 1b77.pdb B;12 1b9c.pdb B 1bb9.pdb B;13 1bdb.pdb B 1bg6.pdb B;14 1bi7.pdb B 1bkb.pdb B;15 1bml.pdb B 1bow.pdb B;16 1btk.pdb B 1bui.pdb B;17 1bw0.pdb B 1byi.pdb B;18 1bzq N 1c3c.pdb B;19 1c76.pdb B 1c7s.pdb B;20 1c9o.pdb B 1cbq.pdb B;21 1chm.pdb B 1ci4.pdb B;22 1cku.pdb B 1clx_2.pdb D;23 1cnz.pdb B 1cru.pdb B;24 1ctt.pdb B 1cun.pdb B;25 1cxp.pdb C 1d02.pdb B;26 1d2s.pdb B 1d3d.pdb B;27 1d6r.pdb I 1d8h_2.pdb C;28 1d9c.pdb B 1dcf.pdb B;29 1dfn.pdb B 1dhf.pdb B;30 1dj7.pdb B 1dkg.pdb B;31 1dov.pdb B 1dq9.pdb B;32 1dqz.pdb B 1dun.pdb B;33 1dvp.pdb B 1dyo.pdb B;34 1e1z.pdb A 1e3h.pdb B;35 1e50 A 1e6j.pdb L;36 1eak C 1eb8.pdb B;37 1ecm.pdb B 1edt.pdb B;38 1eeq.pdb B 1efu.pdb B;39 1ei5.pdb B 1eja.pdb B;40 1eku.pdb B 1ems.pdb B;41 1ern.pdb B 1esc.pdb B;42 1ev7.pdb B 1ew3.pdb B;43 1ewy C 1eyv.pdb B;44 1f0c.pdb B 1f1m_2.pdb D;45 1f2u.pdb B 1f3g.pdb B;46 1f5v.pdb B 1f6m_2.pdb F;47 1f8e.pdb B 1f9z.pdb B;48 1fbt.pdb B 1fe0.pdb B;49 1fiu.pdb B 1flg.pdb B;50 1fm0.pdb E 1fo1.pdb B;51 1fr2.pdb B 1frt.pdb C;52 1fsg.pdb C 1fua.pdb B;53 1fwx D 1g0s.pdb B;54 1g5g E 1g60.pdb B;55 1g8k F 1g8x.pdb B;56 1gg6.pdb C 1ghq.pdb B;57 1gka.pdb B 1gl2.pdb B;58 1gmv.pdb B 1gpe.pdb B;59 1gr0.pdb B 1gt3.pdb B;60 1gtw.pdb B 1gve.pdb B;61 1gxc B 1gxq.pdb B;62 1gyg B 1gz5.pdb B;63 1gzj.pdb B 1h1w.pdb B;64 1h4p.pdb B 1h5b_2.pdb D;65 1h6o.pdb B 1h7z.pdb B;66 1hbx E 1hcj_2.pdb B;67 1he1 C 1hf2_1.pdb B;68 1hh2.pdb A 1hle.pdb B;69 1hqr.pdb B 1hru.pdb B;70 1htr.pdb P 1hvv.pdb B;71 1hyl.pdb B 1hyr.pdb A;72 1i0r.pdb B 1i2k.pdb B;73 1i31.pdb P 1i4m.pdb B;74 1i85.pdb B 1ia9.pdb B;75 1ibr D 1id1.pdb B;76 1ii7.pdb B 1ijy.pdb B;77 1iom.pdb B 1ire.pdb B;78 1isy.pdb B 1ivy.pdb B;79 1j1b.pdb B 1j2g.pdb B;80 1j3b.pdb B 1j5s.pdb B;81 1jay.pdb B 1jcd.pdb B;82 1jdp.pdb B 1jey.pdb B;83 1jg8.pdb B 1jhl.pdb A;84 1jkj B 1jl0.pdb B;85 1jlv B 1jmk.pdb O;86 1jqp.pdb B 1jrh.pdb H;87 1js3.pdb B 1jub.pdb B;88 1jvm.pdb B 1jye.pdb B;89 1k3e.pdb B 1k4m.pdb B;90 1k5n.pdb B 1k8r.pdb B;91 1kc7.pdb B 1kcm.pdb B;92 1kfi.pdb B 1kgy_1.pdb D;93 1ki1 D 1kjn.pdb B;94 1knc.pdb B 1ko6.pdb C;95 1kor.pdb B 1ksh.pdb B;96 1kut.pdb B 1kwg.pdb B;97 1l0a.pdb B 1l3a.pdb B;98 1l3p.pdb B 1l6r.pdb B;99 1ldf.pdb B 1lfb.pdb B;100 1lj9.pdb B 1llm.pdb D;101 1lq9.pdb B 1lqv_2.pdb D;102 1ltx.pdb B 1lw7.pdb B;103 1lxd.pdb B 1m0w.pdb B;104 1m3w.pdb B 1m4u.pdb A;105 1m6d.pdb B 1m7y.pdb B;106 1mg1.pdb B 1mhm.pdb A;107 1miw.pdb B 1mju.pdb H;108 1mkf.pdb B 1mml.pdb B;109 1mpy.pdb B 1mr1_2.pdb C;110 1msc.pdb B 1mv5.pdb B;111 1my7.pdb B 1mzb.pdb B;112 1n12 D 1n1q.pdb B;113 1n2m E 1n71_1.pdb B;114 1naw.pdb B 1nbf_2.pdb C;115 1nco.pdb B 1neu.pdb B;116 1ni4.pdb B 1njr.pdb B;117 1nkt.pdb B 1nls.pdb B;118 1nme.pdb B 1nog.pdb B;119 1nrj.pdb B 1nsj.pdb B;120 1nt2.pdb B 1nv7.pdb B;121 1nxj B 1nxz.pdb B;122 1o0s.pdb B 1o1x.pdb B;123 1o4w.pdb B 1o5d.pdb H;124 1o6z.pdb B 1o7l.pdb B;125 1o89.pdb B 1o9l.pdb B;126 1obx.pdb C 1ocu.pdb B;127 1oe9.pdb B 1oft.pdb B;128 1og6.pdb B 1oi6.pdb B;129 1oki.pdb B 1olp.pdb B;130 1omw.pdb B 1ooz.pdb B;131 1oru.pdb B 1osy.pdb B;132 1ou0.pdb B 1ovm.pdb B;133 1ox0.pdb B 1ozf.pdb B;134 1p35 B 1p5v.pdb B;135 1p74.pdb B 1p9e.pdb B;136 1pdo.pdb B 1peq.pdb B;137 1pgr F 1piw.pdb B;138 1pk1 B 1pl5.pdb S;139 1pqw.pdb B 1ptm.pdb B;140 1pv1.pdb B 1pwb.pdb B;141 1q0q.pdb B 1q16.pdb B;142 1q40 D 1q4t.pdb B;143 1q67.pdb B 1q8f.pdb B;144 1qc7.pdb B 1qdn.pdb B;145 1qh3.pdb B 1qi9.pdb B;146 1qks.pdb B 1qlw.pdb B;147 1qmu.pdb B 1qo8.pdb D;148 1qoz.pdb B 1qqg.pdb B;149 1qup.pdb B 1qvz.pdb B;150 1qx4.pdb B 1qz8.pdb B;151 1r1k.pdb A 1r29.pdb B;152 1r4m F 1r61.pdb B;153 1r7a.pdb B 1r8j.pdb B;154 1re3 B 1rer.pdb B;155 1rgf.pdb B 1ris.pdb B;156 1rmd.pdb B 1ro7.pdb B;157 1rqi.pdb B 1ru0.pdb B;158 1rw0.pdb B 1ryi_2.pdb B;159 1s1q D 1s3o.pdb B;160 1s5a.pdb B 1s7h_2.pdb D;161 1sbb.pdb B 1sc1.pdb B;162 1sdd.pdb B 1sek.pdb B;163 1sfp.pdb B 1sgm.pdb B;164 1skz.pdb B 1smo.pdb B;165 1sox.pdb B 1sqe.pdb B;166 1stz.pdb B 1sur.pdb B;167 1svx.pdb B 1sy6.pdb H;168 1sz2.pdb B 1t0h.pdb B;169 1t3l.pdb B 1t4b.pdb B;170 1t5o D 1t6n.pdb B;171 1t9i.pdb B 1taf.pdb B;172 1tco.pdb B 1te2.pdb B;173 1tf0.pdb B 1tht.pdb B;174 1tm0.pdb B 1to3.pdb B;175 1tqb.pdb B 1ttw.pdb B;176 1tvn.pdb B 1twd.pdb B;177 1txg.pdb B 1ty0_1.pdb B;178 1tz9.pdb B 1u0m.pdb B;179 1u6g.pdb B 1u7g.pdb B;180 1u8s.pdb B 1uad_2.pdb D;181 1udd.pdb B 1udv.pdb B;182 1ufo B 1uis.pdb B;183 1ujq.pdb B 1ulk.pdb B;184 1unn.pdb B 1uqt.pdb B;185 1us7.pdb B 1usq_2.pdb B;186 1uuj.pdb B 1uv7.pdb B;187 1uwk.pdb B 1uxt.pdb B;188 1uzb.pdb B 1v13.pdb B;189 1v4v.pdb B 1v5v.pdb B;190 1v72.pdb B 1v7p.pdb B;191 1vbk.pdb B 1vch_3.pdb E;192 1vdw.pdb B 1vef.pdb B;193 1vg0.pdb B 1vhx.pdb B;194 1vj2.pdb B 1vjo.pdb B;195 1vka.pdb B 1vkn.pdb B;196 1vme.pdb B 1vp2.pdb B;197 1vpz.pdb B 1vr0_1.pdb B;198 1vrs E 1vzi.pdb B;199 1w36 F 1w53.pdb B;200 1w6g.pdb B 1w9e.pdb B;201 1who.pdb B 1wiy.pdb B;202 1wmh.pdb B 1wok.pdb B;203 1wq1.pdb G 1wu7.pdb B;204 1ww1.pdb B 1wwr_2.pdb D;205 1wyi.pdb B 1x1z.pdb B;206 1x8z C 1x9g.pdb B;207 1xcf.pdb B 1xdi.pdb B;208 1xdt.pdb R 1xg5.pdb B;209 1xk7 B 1xku.pdb B;210 1xm3.pdb B 1xoc.pdb B;211 1xs0 B 1xtg.pdb B;212 1xuv B 1xvi.pdb B;213 1xwr.pdb B 1y0h.pdb B;214 1y4m.pdb B 1y6k.pdb R;215 1y7m.pdb B 1y9b.pdb B;216 1ycc.pdb B 1ycs.pdb B;217 1yer.pdb B 1yg2.pdb B;218 1yj5 B 1ynb.pdb B;219 1yrb.pdb B 1ys7.pdb B;220 1yu0.pdb B 1yw6.pdb B;221 1z3e.pdb B 1z5b.pdb B;222 1z84.pdb B 1z9w.pdb B;223 1zbo.pdb B 1zd3.pdb B;224 1zke E 1zmb_3.pdb F;225 1zp6.pdb B 1zq9.pdb B;226 1zvp D 1zym.pdb B;227 2a2q.pdb H 2a42.pdb B;228 2a5t.pdb B 2a7w_2.pdb F;229 2aca.pdb B 2ae6.pdb B;230 2af4.pdb D 2aib.pdb B;231 2arh.pdb B 2asf.pdb B;232 2avp.pdb B 2axp.pdb B;233 2ayo.pdb B 2b0c.pdb B;234 2b5g.pdb B 2b69.pdb B;235 2b9c.pdb B 2bbk.pdb J;236 2bgw.pdb B 2bi0.pdb B;237 2bky.pdb B 2bnx.pdb B;238 2bp3 T 2bz1.pdb B;239 2c4n.pdb B 2c5w.pdb A;240 2ca1.pdb B 2cch_1.pdb B;241 2cn4.pdb B 2cro.pdb B;242 2cvz.pdb B 2cy9.pdb B;243 2d6y.pdb B 2d9q.pdb B;244 2dp9.pdb B 2dvt.pdb B;245 2e2d.pdb C 2ess.pdb B;246 2f1f.pdb B 2f2a.pdb B;247 2f3d.pdb B 2f5v.pdb B;248 2f96.pdb B 2f9y.pdb B;249 2fbk.pdb B 2fd5.pdb B;250 2fex B 2fgq.pdb A;251 2fml.pdb B 2fnj.pdb B;252 2fp4.pdb B 2fs2.pdb B;253 2fzt.pdb B 2g2u.pdb B;254 2g40.pdb B 2g6w.pdb B;255 2g7s.pdb B 2gb3_1.pdb B;256 2gh1.pdb B 2gi3.pdb B;257 2gj7.pdb F 2gol_1.pdb B;258 2gyq.pdb B 2gzd.pdb B;259 2h7z.pdb B 2hbv.pdb B;260 2hew.pdb A 2hmf.pdb B;261 2hu7.pdb B 2hxv.pdb B;262 2i4j.pdb B 2ibg_1.pdb F;263 2ijd A 2io8.pdb B;264 2ixc.pdb B 2j6b.pdb B;265 2jhf.pdb B 2nac.pdb B;266 2nul.pdb B 2nyc.pdb B;267 2o23.pdb B 2omy.pdb B;268 2pg2.pdb B 2phn.pdb B;269 2pva.pdb B 2q48.pdb B;270 2rln.pdb E 2tdt.pdb B;271 3cla.pdb B 3dni.pdb B;272 3grs.pdb B 3lyn.pdb B;273 4ake.pdb B 4cpv.pdb B;274 4sgb.pdb I 5rub.pdb B;275 12as.pdb B 1a2y.pdb B;276 1a2z.pdb B 1a8o.pdb B;277 1a73.pdb B 1ad3.pdb B;278 1adu.pdb B 1am2.pdb B;279 1ais.pdb B 1aqt.pdb B;280 1aoh.pdb B 1aui.pdb B;281 1auo.pdb B 1axi.pdb B;282 1avz.pdb B 1b0u.pdb B;283 1b1z.pdb B 1b5p.pdb B;284 1b49.pdb C 1b8m.pdb B;285 1b6u.pdb B 1bcm.pdb B;286 1bdb.pdb B 1bhg.pdb B;287 1bgf.pdb B 1blx.pdb B;288 1bml.pdb B 1brw.pdb B;289 1bqp B 1bvn.pdb T;290 1btk.pdb B 1byu.pdb B;291 1bzq N 1c4z.pdb B;292 1c3k.pdb B 1c94.pdb B;293 1c9o.pdb B 1cf7.pdb B;294 1cd9.pdb B 1cjt.pdb B;295 1chm.pdb B 1cnt_2.pdb 3;296 1cnz.pdb B 1csn.pdb B;297 1cs1.pdb B 1cvs.pdb B;298 1cxp.pdb C 1d1z.pdb B;299 1d0q.pdb B 1d4x.pdb G;300 1d2s.pdb B 1d8w.pdb B;301 1d9c.pdb B 1dev_1.pdb B;302 1dd3 B 1div.pdb B;303 1dj7.pdb B 1dmu.pdb C;304 1dl5.pdb B 1dqn.pdb B;305 1dov.pdb B 1dvk.pdb B;306 1dvp.pdb B 1e0b.pdb B;307 1dyw.pdb B 1e4e.pdb B;308 1e50 A 1e96.pdb B;309 1e7n.pdb B 1ec8.pdb B;310 1eak C 1eep_1.pdb B;311 1eeq.pdb B 1egp.pdb B;312 1eg4.pdb P 1eke.pdb B;313 1eku.pdb B 1epf_2.pdb D;314 1eo6.pdb B 1euv.pdb B;315 1ern.pdb B 1ewr.pdb B;316 1ewy C 1ezx.pdb C;317 1ezg.pdb B 1f2l.pdb B;318 1f2u.pdb B 1f46.pdb B;319 1f3u F 1f7l.pdb B;320 1f5v.pdb B 1fbl.pdb B;321 1fbt.pdb B 1fgj_1.pdb B;322 1fe8 J 1flo.pdb B;323 1fm0.pdb E 1fq1.pdb B;324 1fp1.pdb A 1fs2.pdb B;325 1fr2.pdb B 1fvk.pdb B;326 1fwx D 1g3n_1.pdb B;327 1g1j.pdb B 1g73_1.pdb C;328 1g8k F 1gdt.pdb B;329 1gc1 C 1gk1_2.pdb B;330 1gg6.pdb C 1gml_1.pdb D;331 1gmv.pdb B 1gqi.pdb B;332 1gpm.pdb B 1gtd.pdb B;333 1gtw.pdb B 1gwi.pdb B;334 1gvn.pdb B 1gxy.pdb B;335 1gxc B 1gzh_1.pdb B;336 1gzj.pdb B 1h3f.pdb B;337 1h21.pdb B 1h6d_2.pdb F;338 1h6o.pdb B 1h9m_1.pdb B;339 1h8g.pdb B 1hcx.pdb B;340 1hbx E 1hg5.pdb B;341 1hh2.pdb A 1hng.pdb B;342 1hlo.pdb B 1hss_1.pdb B;343 1htr.pdb P 1hxm_3.pdb F;344 1hw7.pdb B 1hzh.pdb K;345 1hyl.pdb B 1i2s.pdb B;346 1i31.pdb P 1i75.pdb B;347 1i52.pdb B 1iar.pdb B;348 1ibr D 1igq_1.pdb C;349 1ied.pdb B 1ilr.pdb 2;350 1ii7.pdb B 1is3.pdb B;351 1isy.pdb B 1iyb.pdb B;352 1ix2.pdb B 1j30.pdb B;353 1j3b.pdb B 1j9l.pdb B;354 1j7n.pdb B 1jd0.pdb B;355 1jay.pdb B 1jfz_1.pdb B;356 1jg8.pdb B 1jk4.pdb B;357 1ji7.pdb B 1jl9.pdb B;358 1jlv B 1jps.pdb H;359 1joc.pdb B 1js0.pdb B;360 1jqp.pdb B 1jv2.pdb B;361 1jvm.pdb B 1k0d_1.pdb B;362 1jys.pdb B 1k55_1.pdb C;363 1k5n.pdb B 1kae.pdb B;364 1k94.pdb B 1kdg.pdb B;365 1kc7.pdb B 1khx.pdb B;366 1ki1 D 1klg.pdb B;367 1kk8.pdb B 1kob.pdb B;368 1kor.pdb B 1ktz.pdb B;369 1ktb.pdb B 1kyq_2.pdb C;370 1kut.pdb B 1l3l_1.pdb D;371 1l3p.pdb B 1lb6.pdb B;372 1l7d D 1li4.pdb B;373 1lj9.pdb B 1log.pdb B;374 1lm8.pdb C 1lss_1.pdb B;375 1lq9.pdb B 1lx5.pdb B;376 1lxd.pdb B 1m2t.pdb B;377 1m1l.pdb B 1m63_1.pdb B;378 1m6d.pdb B 1mby.pdb B;379 1m9x C 1mi1.pdb B;380 1mg1.pdb B 1mka.pdb B;381 1mkf.pdb B 1moy.pdb B;382 1mo9.pdb B 1mrp.pdb B;383 1msc.pdb B 1mwv.pdb B;384 1mvk J 1mzn_1.pdb B;385 1my7.pdb B 1n2f.pdb B;386 1n2m E 1n93.pdb A;387 1n7h.pdb B 1nby.pdb B;388 1nco.pdb B 1ngm_2.pdb F;389 1nf3 C 1nkn_2.pdb D;390 1ni4.pdb B 1nmb.pdb L;391 1nme.pdb B 1nqd.pdb B;392 1nox.pdb B 1nsz.pdb B;393 1nt2.pdb B 1nw2_2.pdb F;394 1nvm B 1nyt.pdb B;395 1nxj B 1o3u.pdb B;396 1o4w.pdb B 1o6a.pdb B;397 1o5h.pdb B 1o81.pdb B;398 1o89.pdb B 1oaz_1.pdb H;399 1oa8.pdb B 1od4.pdb B;400 1obx.pdb C 1og5.pdb B;401 1og6.pdb B 1ok3.pdb B;402 1oio.pdb B 1olz.pdb B;403 1omw.pdb B 1or0_2.pdb D;404 1ope.pdb B 1otj_2.pdb C;405 1oru.pdb B 1owf.pdb B;406 1ox0.pdb B 1p27.pdb B;407 1p0x.pdb B 1p6a.pdb B;408 1p74.pdb B 1pc6.pdb B;409 1p9o.pdb B 1pff.pdb B;410 1pdo.pdb B 1pjq.pdb B;411 1pk1 B 1poc.pdb B;412 1pm3.pdb B 1puc.pdb B;413 1pv1.pdb B 1pym.pdb B;414 1pxv C 1q2w.pdb B;415 1q0q.pdb B 1q5y.pdb B;416 1q67.pdb B 1qav.pdb B;417 1q98.pdb B 1qfx.pdb B;418 1qh3.pdb B 1qkd.pdb B;419 1qj8.pdb B 1qme.pdb B;420 1qks.pdb B 1qoy.pdb B;421 1qoz.pdb B 1qsm.pdb B;422 1qre.pdb B 1qwh.pdb B;423 1qx4.pdb B 1r0v_2.pdb D;424 1qzq.pdb B 1r3s.pdb B;425 1r1k.pdb A 1r6w.pdb B;426 1r7a.pdb B 1rcq.pdb B;427 1r8s.pdb E 1rfz.pdb B;428 1rgf.pdb B 1rkd.pdb B;429 1rj9.pdb B 1rq2.pdb B;430 1rmd.pdb B 1rvg_1.pdb B;431 1rw0.pdb B 1rzr_1.pdb C;432 1rz1 B 1s4k.pdb B;433 1s5a.pdb B 1s96.pdb B;434 1s7m E 1scf_1.pdb B;435 1sbb.pdb B 1sfn.pdb B;436 1sfp.pdb B 1sjp.pdb B;437 1sh5.pdb B 1sny.pdb B;438 1sox.pdb B 1sr9.pdb B;439 1sqs.pdb B 1svd.pdb M;440 1stz.pdb B 1syy.pdb B;441 1sz2.pdb B 1t2d.pdb B;442 1t11.pdb B 1t5b.pdb B;443 1t5o D 1t8q_2.pdb C;444 1t70 H 1tbx.pdb B;445 1t9i.pdb B 1tej.pdb B;446 1tf0.pdb B 1tlj.pdb B;447 1tj7.pdb B 1toq_3.pdb F;448 1tqb.pdb B 1tuv.pdb B;449 1tue L 1tx3_2.pdb D;450 1tvn.pdb B 1tyy.pdb B;451 1tz9.pdb B 1u5k.pdb B;452 1u19.pdb B 1u7z_1.pdb B;453 1u8s.pdb B 1uby.pdb B;454 1uaz.pdb B 1uf5.pdb B;455 1udd.pdb B 1uj2.pdb B;456 1ujq.pdb B 1un8.pdb B;457 1um0.pdb B 1uru.pdb B;458 1us7.pdb B 1utg.pdb B;459 1ut7.pdb B 1uw1.pdb B;460 1uuj.pdb B 1uz2.pdb A;461 1uzb.pdb B 1v3e.pdb B;462 1v1p.pdb B 1v70.pdb B;463 1v72.pdb B 1v8q.pdb B;464 1v8c B 1vd6.pdb B;465 1vbk.pdb B 1vfp.pdb B;466 1vg0.pdb B 1vio.pdb B;467 1vi0.pdb B 1vk6.pdb B;468 1vka.pdb B 1vlf_1.pdb N;469 1vl0.pdb B 1vp7_1.pdb B;470 1vme.pdb B 1vrb_2.pdb D;471 1vrs E 1w2i.pdb B;472 1w07.pdb B 1w63_1.pdb B;473 1w6g.pdb B 1wc9.pdb B;474 1wa5.pdb B 1wlh.pdb B;475 1who.pdb B 1wpw.pdb B;476 1wq1.pdb G 1wve.pdb C;477 1wud.pdb B 1wxx_1.pdb B;478 1wyi.pdb B 1x77.pdb B;479 1x3m.pdb B 1xb2.pdb B;480 1x8z C 1xds.pdb B;481 1xdt.pdb R 1xi9.pdb B;482 1xgs.pdb B 1xl4.pdb B;483 1xm3.pdb B 1xqa.pdb B;484 1xpj.pdb B 1xu9.pdb B;485 1xs0 B 1xwm.pdb B;486 1xwr.pdb B 1y2o.pdb B;487 1y0z.pdb B 1y71.pdb B;488 1y7m.pdb B 1yb5.pdb B;489 1yak B 1ydg_2.pdb F;490 1ycc.pdb B 1yiz.pdb B;491 1yj5 B 1ypt.pdb B;492 1ynr.pdb B 1yta_1.pdb B;493 1yu0.pdb B 1z0p.pdb B;494 1yxb F 1z6b_1.pdb B;495 1z3e.pdb B 1zbd.pdb B;496 1zbo.pdb B 1zjc.pdb B;497 1zed.pdb B 1zno.pdb B;498 1zp6.pdb B 1ztd.pdb B;499 1zrs.pdb B 2a1j.pdb B;500 1zvp D 2a5l.pdb B;501 2a5t.pdb B 2aaf.pdb B;502 2a99.pdb B 2aee.pdb B;503 2af4.pdb D 2akz.pdb B;504 2aj7.pdb B 2av9_2.pdb H;505 2arh.pdb B 2ayn.pdb B;506 2ayo.pdb B 2b4j.pdb B;507 2b0l B 2b8m.pdb B;508 2b9c.pdb B 2be3.pdb B;509 2bcn.pdb B 2bko.pdb B;510 2bgw.pdb B 2bop.pdb D;511 2bp3 T 2c31.pdb B;512 2c12 B 2c9l.pdb Z;513 2ca1.pdb B 2cjt_2.pdb D;514 2ch5 B 2ctz.pdb B;515 2cn4.pdb B 2cyy.pdb B;516 2cz1.pdb B 2dex.pdb A;517 2d6y.pdb B 2e2a.pdb B;518 2e2d.pdb C 2ey4_1.pdb E;519 2eul B 2f2e.pdb B;520 2f1f.pdb B 2f66_1.pdb B;521 2f69.pdb B 2fb1_1.pdb B;522 2f96.pdb B 2fe1.pdb B;523 2fex B 2fit.pdb B;524 2fhq.pdb B 2fnu.pdb B;525 2fml.pdb B 2ftr.pdb B;526 2ftw.pdb B 2g38_1.pdb B;527 2fzt.pdb B 2g7l.pdb B;528 2g7s.pdb B 2gec.pdb B;529 2gc7 F 2gib.pdb B;530 2gh1.pdb B 2gr8_1.pdb C;531 2gsm B 2h26.pdb B;532 2gyq.pdb B 2hck.pdb B;533 2hew.pdb A 2hqv.pdb B;534 2hoe.pdb B 2hzb_2.pdb D;535 2hu7.pdb B 2id3.pdb B;536 2idj.pdb B 2iqq.pdb B;537 2ijd A 2j88.pdb H;538 2jhf.pdb B 2npt_2.pdb C;539 2nlz.pdb B 2nyr.pdb B;540 2nul.pdb B 2or4.pdb B;541 2otx.pdb B 2pk8.pdb B;542 2pg2.pdb B 2q4i.pdb B;543 2rln.pdb E 2vir.pdb B;544 2tnf.pdb B 3fap.pdb B;545 3cla.pdb B 3mcg.pdb 2;546 3pfk.pdb B 4hb1.pdb B;547 4ake.pdb B 6q21.pdb B;548 1a17.pdb B 1a8y.pdb B;549 1a3a D 1adq.pdb L;550 1a78.pdb B 1ajs.pdb B;551 1af6.pdb B 1aqu.pdb B;552 1ajs.pdb B 1auo.pdb B;553 1as4.pdb B 1ay2.pdb B;554 1auv.pdb B 1b0x.pdb B;555 1awc.pdb B 1b4w.pdb B;556 1b25.pdb B 1b8z.pdb B;557 1b4w.pdb B 1bdb.pdb B;558 1b9e.pdb B 1bhh.pdb B;559 1bdf.pdb B 1bm9_1.pdb B;560 1bgp.pdb B 1bqu.pdb B;561 1bmo.pdb B 1bvp_2.pdb 5;562 1bqu.pdb B 1bzq_2.pdb N;563 1bwn.pdb B 1c5e.pdb B;564 1c02.pdb B 1c9k.pdb B;565 1c3r.pdb B 1ce9.pdb B;566 1ca0 C 1cjx.pdb B;567 1ce9.pdb B 1cnz.pdb B;568 1cl7.pdb H 1ct9_2.pdb C;569 1coi.pdb B 1cx8_1.pdb B;570 1cs6.pdb B 1d1j.pdb B;571 1cxq.pdb B 1d5f.pdb B;572 1d1j.pdb B 1d9c.pdb B;573 1d7b.pdb B 1df4.pdb B;574 1dan.pdb H 1dj0.pdb B;575 1ddt.pdb B 1dle.pdb B;576 1dj8 F 1dqs.pdb B;577 1dle.pdb B 1dvp.pdb B;578 1dru.pdb B 1e19.pdb B;579 1dxg.pdb B 1e4m.pdb A;580 1dza.pdb B 1e85.pdb B;581 1e58.pdb B 1ecf.pdb B;582 1e85.pdb B 1eeq.pdb B;583 1ecs.pdb B 1egw_2.pdb D;584 1eer.pdb B 1ekq_2.pdb B;585 1eg5.pdb B 1ep3.pdb B;586 1el6.pdb B 1euw.pdb B;587 1ep3.pdb B 1ewy_1.pdb C;588 1evj C 1f02.pdb T;589 1ex0.pdb B 1f2t.pdb B;590 1ezi.pdb B 1f3v.pdb B;591 1f2v.pdb B 1f86.pdb B;592 1f3v.pdb B 1fbt.pdb B;593 1f8f.pdb B 1fgu.pdb B;594 1fbv.pdb C 1flt.pdb W;595 1few.pdb B 1fp2.pdb B;596 1fmc.pdb B 1fs8.pdb B;597 1fp2.pdb B 1fwx_2.pdb D;598 1fsk K 1g4u.pdb R;599 1fx2.pdb B 1g8e.pdb B;600 1g29.pdb 2 1gcj.pdb B;601 1g8l.pdb B 1gk6.pdb A;602 1gcj.pdb B 1gmv.pdb B;603 1gkj.pdb B 1gqn.pdb B;604 1gmw.pdb B 1gte_1.pdb B;605 1gpq.pdb B 1gvp.pdb B;606 1gu2.pdb B 1gy6.pdb B;607 1gvp.pdb B 1gzj.pdb B;608 1gyo.pdb B 1h3o_1.pdb B;609 1gzs D 1h6k_2.pdb Y;610 1h2b.pdb B 1h8p.pdb B;611 1h6p.pdb B 1hdh.pdb B;612 1h8p.pdb B 1hh2.pdb A;613 1he7.pdb B 1hnj.pdb B;614 1hia B 1htn.pdb B;615 1hlq.pdb B 1hwg.pdb B;616 1hul.pdb B 1hzy.pdb B;617 1hwg.pdb B 1i31.pdb P;618 1i12 C 1i7b.pdb A;619 1i36.pdb B 1ib1_1.pdb B;620 1i58.pdb B 1ig0.pdb B;621 1iby B 1im3_3.pdb L;622 1ig0.pdb B 1isy.pdb B;623 1ips.pdb B 1izm.pdb B;624 1itb.pdb B 1j31_2.pdb D;625 1ix9.pdb B 1j8b.pdb B;626 1j3k.pdb B 1jdf_1.pdb B;627 1j8b.pdb B 1jg8.pdb B;628 1jdw.pdb B 1jk9_2.pdb D;629 1jgs.pdb B 1jlt.pdb B;630 1jil.pdb B 1jof_1.pdb B;631 1jlx.pdb B 1js1.pdb Y;632 1jof B 1jvm.pdb B;633 1jsd.pdb B 1k1e_3.pdb J;634 1jw9.pdb D 1k5d_1.pdb B;635 1jz7.pdb B 1ka2.pdb B;636 1k66.pdb B 1kez_1.pdb B;637 1ka2.pdb B 1ki1_2.pdb D;638 1kfu.pdb S 1klo.pdb B;639 1ki9.pdb B 1kol.pdb B;640 1kke.pdb B 1ktj.pdb B;641 1kpf.pdb B 1kzq.pdb B;642 1ktj.pdb B 1l3p.pdb B;643 1l0o.pdb B 1lc5.pdb B;644 1l4d.pdb B 1lid.pdb B;645 1l7l.pdb B 1lmb.pdb 4;646 1lk3 M 1lt8.pdb B;647 1lmb.pdb 4 1lxd.pdb B;648 1lua.pdb B 1m2v.pdb B;649 1lxe.pdb B 1m65.pdb B;650 1m1n B 1ma9.pdb B;651 1m6e.pdb A 1mi3_2.pdb D;652 1ma9.pdb B 1mkf.pdb B;653 1mjf.pdb B 1mp4.pdb B;654 1mkk.pdb B 1mrz.pdb B;655 1moe.pdb B 1mvo.pdb B;656 1msp.pdb B 1n0l_1.pdb B;657 1mvo.pdb B 1n2m_2.pdb E;658 1n1b.pdb B 1n9p.pdb B;659 1n2s.pdb B 1nc7.pdb B;660 1n7s.pdb B 1nf9.pdb B;661 1nd4.pdb B 1nkp_1.pdb B;662 1nf9.pdb B 1nme.pdb B;663 1nkv.pdb B 1nqk.pdb B;664 1nms.pdb B 1nt0.pdb G;665 1noy.pdb B 1nvp.pdb B;666 1nt3.pdb B 1nzi.pdb B;667 1nvp.pdb B 1o4w.pdb B;668 1o0v.pdb B 1o6e.pdb B;669 1o4z D 1o84.pdb B;670 1o5k.pdb B 1oaa.pdb B;671 1o8b.pdb B 1oe0.pdb B;672 1oaa.pdb B 1og6.pdb B;673 1oey M 1ok7.pdb B;674 1ogs.pdb B 1oms.pdb B;675 1oj4.pdb B 1oph.pdb B;676 1omz.pdb B 1otk.pdb B;677 1oph.pdb B 1ox0.pdb B;678 1ou8 B 1p2j.pdb I;679 1oxk B 1p6o.pdb B;680 1p16 C 1pbi.pdb B;681 1p7h M 1pfo.pdb B;682 1pbi.pdb B 1pk1_1.pdb B;683 1pgu.pdb B 1ppr.pdb N;684 1pk8 B 1puf.pdb B;685 1pm4.pdb B 1py1_1.pdb B;686 1pv9.pdb B 1q3g_2.pdb F;687 1py1 B 1q67.pdb B;688 1q43 B 1qb2.pdb B;689 1q6o.pdb B 1qge.pdb E;690 1q9u.pdb B 1qja.pdb B;691 1qh4 B 1qmg_2.pdb D;692 1qja.pdb B 1qoz.pdb B;693 1qnn.pdb B 1qtn.pdb B;694 1qp1.pdb B 1qwt.pdb B;695 1qrq.pdb B 1r0m_2.pdb D;696 1qx8.pdb B 1r43.pdb B;697 1r0m D 1r7a.pdb B;698 1r4w D 1rcu.pdb B;699 1r7j.pdb B 1rg9.pdb B;700 1r9c.pdb B 1rjl.pdb C;701 1rgx.pdb B 1rqb.pdb B;702 1rjl.pdb C 1rw0.pdb B;703 1rrm.pdb B 1s0p.pdb B;704 1rw6.pdb B 1s4n.pdb B;705 1rzh.pdb M 1s7o.pdb B;706 1s5u B 1scj.pdb B;707 1s7o.pdb B 1sfp.pdb B;708 1sdx.pdb E 1sjw.pdb B;709 1sfx.pdb B 1so2_2.pdb D;710 1sh8.pdb B 1squ.pdb B;711 1spp.pdb A 1svp.pdb B;712 1squ.pdb B 1sz2.pdb B;713 1sw6.pdb B 1t3c.pdb B;714 1sz6.pdb B 1t5h.pdb A;715 1t15.pdb B 1t7r.pdb B;716 1t62.pdb B 1tc1.pdb B;717 1t7r.pdb B 1tf0.pdb B;718 1td2.pdb B 1tlk.pdb B;719 1tfe.pdb B 1tq8_2.pdb B;720 1tjc.pdb B 1tuh.pdb B;721 1tqy F 1tx4.pdb B;722 1tuh.pdb B 1tz9.pdb B;723 1txn.pdb B 1u5x.pdb B;724 1tzb.pdb B 1u8c.pdb B;725 1u1i.pdb B 1ub0.pdb B;726 1u8v.pdb B 1ufh.pdb B;727 1ub0.pdb B 1ujq.pdb B;728 1ufy.pdb B 1unh.pdb B;729 1uk8.pdb B 1urz_1.pdb B;730 1ump C 1ut8.pdb B;731 1usc.pdb B 1uw4_1.pdb B;732 1ut8.pdb B 1uzb.pdb B;733 1uwu.pdb B 1v3v.pdb B;734 1uzm.pdb B 1v71.pdb B;735 1v25.pdb B 1v8d.pdb B;736 1v74.pdb B 1vdd.pdb B;737 1v8d.pdb B 1vg0.pdb B;738 1ve1.pdb B 1viu_1.pdb B;739 1vgy.pdb B 1vk8.pdb B;740 1vi2.pdb B 1vl4.pdb B;741 1vkd D 1vpb.pdb B;742 1vl4.pdb B 1vrs_2.pdb E;743 1vq0.pdb B 1w2w_2.pdb F;744 1vrw.pdb B 1w68.pdb B;745 1w0m B 1wbh.pdb B;746 1w6s.pdb B 1wlu.pdb B;747 1wbh.pdb B 1wq1.pdb G;748 1wmi.pdb B 1wvf.pdb B;749 1wqw.pdb B 1wy5.pdb B;750 1wui.pdb L 1x3z.pdb B;751 1wyu B 1xb4_1.pdb B;752 1x3z.pdb B 1xdt.pdb R;753 1xcg B 1xiw_1.pdb B;754 1xe7 C 1xly.pdb B;755 1xhk.pdb B 1xpm_1.pdb B;756 1xma.pdb B 1xub.pdb B;757 1xpm B 1xwr.pdb B;758 1xv2 B 1y43.pdb B;759 1xx6.pdb B 1y7b.pdb B;760 1y14.pdb B 1yav.pdb B;761 1y7p C 1ydw.pdb B;762 1yav.pdb B 1yj5_1.pdb B;763 1yf5.pdb A 1yqf_1.pdb B;764 1ykh.pdb B 1ytf.pdb B;765 1yoa.pdb B 1yz3.pdb B;766 1yuz.pdb B 1z72.pdb B;767 1yz3.pdb B 1zbo.pdb B;768 1z8u D 1zk4.pdb B;769 1zbr.pdb B 1znp_1.pdb B;770 1zee.pdb B 1zru.pdb B;771 1zpd.pdb B 2a26_1.pdb B;772 1zru.pdb B 2a5t.pdb B;773 2a2u.pdb B 2aal_1.pdb B;774 2a5z.pdb B 2aeu.pdb B;775 2a9k.pdb B 2ajf_1.pdb E;776 2ag5.pdb B 2avd.pdb B;777 2ajf E 2ayo.pdb B;778 2awi D 2b59.pdb B;779 2ayu.pdb B 2b8t.pdb B;780 2b1y.pdb B 2bde.pdb B;781 2ba2.pdb B 2bkr.pdb B;782 2bde.pdb B 2bp3_1.pdb T;783 2bll.pdb B 2c35_4.pdb H;784 2bpl C 2c9v.pdb F;785 2c1n C 2ch7.pdb B;786 2car.pdb B 2cu6.pdb B;787 2ch7.pdb B 2cz1.pdb B;788 2cwk.pdb B 2dft.pdb B;789 2cz4.pdb B 2e2c.pdb B;790 2d7c D 2evr.pdb B;791 2e7s H 2f2l.pdb X;792 2evr.pdb B 2f69.pdb B;793 2f3x.pdb B 2fb5.pdb B;794 2f6k.pdb B 2fef.pdb B;795 2f9h.pdb B 2fhz.pdb B;796 2ff4 E 2fo7.pdb B;797 2fhz.pdb B 2ftw.pdb B;798 2fpn.pdb B 2g39.pdb B;799 2ftx.pdb B 2g7o.pdb B;800 2g0t.pdb B 2gci_1.pdb B;801 2g82 P 2giy.pdb B;802 2gci B 2gsm_1.pdb B;803 2gj8 D 2h2n.pdb B;804 2gtd F 2hek.pdb B;805 2gys.pdb B 2hox_1.pdb B;806 2hft.pdb B 2i02.pdb B;807 2hox B 2idj.pdb B;808 2i52 B 2it9_1.pdb B;809 2ido D 2jel.pdb H;810 2ilk.pdb B 2nml.pdb B;811 2kin.pdb B 2nz8.pdb B;812 2nml.pdb B 2otx.pdb B;813 2o25 D 2plh.pdb B;814 2oza.pdb B 2q4n.pdb B;815 2pgd.pdb B 2toh.pdb B;816 2rmc.pdb C 3fru_2.pdb D;817 2toh.pdb B 3pfk.pdb B;818 3gtu D 4mt2.pdb B;819 3pnp.pdb B 6rlx.pdb B;820 1a12.pdb B 1a5t.pdb B;821 1a73.pdb B 1ab4.pdb B;822 1aa7.pdb B 1aie.pdb B;823 1ais.pdb B 1aoz.pdb B;824 1aoh.pdb B 1au1.pdb B;825 1aro.pdb L 1avv.pdb B;826 1avz.pdb B 1azt.pdb B;827 1ayf.pdb B 1b3q.pdb B;828 1b49.pdb C 1b76.pdb B;829 1b6u.pdb B 1bb1.pdb B;830 1b9c.pdb B 1bg1.pdb D;831 1bgf.pdb B 1bk5.pdb B;832 1bi7.pdb B 1bou.pdb B;833 1bqp B 1buh.pdb B;834 1btk.pdb B 1byf.pdb B;835 1bw0.pdb B 1c2b.pdb B;836 1c3k.pdb B 1c7n_3.pdb F;837 1c76.pdb B 1cbm.pdb B;838 1cd9.pdb B 1chu.pdb B;839 1chm.pdb B 1clv.pdb I;840 1cku.pdb B 1cqk.pdb B;841 1cs1.pdb B 1cul.pdb B;842 1ctt.pdb B 1czj.pdb B;843 1d0q.pdb B 1d3b_2.pdb D;844 1d2s.pdb B 1d7y.pdb B;845 1d6r.pdb I 1dce_2.pdb D;846 1dd3 B 1dgf.pdb B;847 1dfn.pdb B 1dkf.pdb B;848 1dl5.pdb B 1dpj.pdb B;849 1dov.pdb B 1dto.pdb B;850 1dqz.pdb B 1dy9_1.pdb C;851 1dyw.pdb B 1e2t_1.pdb B;852 1e1z.pdb A 1e6b.pdb B;853 1e7n.pdb B 1eay_1.pdb C;854 1eak C 1edh.pdb B;855 1ecm.pdb B 1efn.pdb B;856 1eg4.pdb P 1eiy.pdb B;857 1ei5.pdb B 1em8_2.pdb D;858 1eo6.pdb B 1es9.pdb B;859 1ern.pdb B 1evy.pdb B;860 1ev7.pdb B 1eys.pdb L;861 1ezg.pdb B 1f1e.pdb B;862 1f0c.pdb B 1f3d.pdb H;863 1f3u F 1f6b.pdb B;864 1f5v.pdb B 1f9j.pdb B;865 1f8e.pdb B 1fd3_1.pdb C;866 1fe8 J 1fle.pdb I;867 1fiu.pdb B 1fnu.pdb B;868 1fp1.pdb A 1frp.pdb B;869 1fr2.pdb B 1ftr.pdb B;870 1fsg.pdb C 1fzd_2.pdb F;871 1g1j.pdb B 1g5y.pdb B;872 1g5g E 1g8t.pdb B;873 1gc1 C 1gh6.pdb B;874 1gg6.pdb C 1gl1_1.pdb I;875 1gka.pdb B 1gp9.pdb B;876 1gpm.pdb B 1gs5.pdb B;877 1gr0.pdb B 1gux.pdb B;878 1gvn.pdb B 1gxj.pdb B;879 1gxc B 1gz0_4.pdb E;880 1gyg B 1h1v.pdb G;881 1h21.pdb B 1h54.pdb B;882 1h4p.pdb B 1h7e.pdb B;883 1h8g.pdb B 1hci.pdb B;884 1hbx E 1hei.pdb B;885 1he1 C 1hl6.pdb B;886 1hlo.pdb B 1hrh.pdb B;887 1hqr.pdb B 1hv8.pdb B;888 1hw7.pdb B 1hyo.pdb B;889 1hyl.pdb B 1i24.pdb B;890 1i0r.pdb B 1i4j.pdb B;891 1i52.pdb B 1i8t.pdb B;892 1i85.pdb B 1icr.pdb B;893 1ied.pdb B 1ij2.pdb B;894 1ii7.pdb B 1iqd.pdb B;895 1iom.pdb B 1iv3_1.pdb B;896 1ix2.pdb B 1j24.pdb B;897 1j1b.pdb B 1j5p.pdb B;898 1j7n.pdb B 1jc4_2.pdb D;899 1jay.pdb B 1jer.pdb B;900 1jdp.pdb B 1jhg.pdb B;901 1ji7.pdb B 1jkx_1.pdb B;902 1jkj B 1jmj.pdb B;903 1joc.pdb B 1jr8.pdb B;904 1jqp.pdb B 1jtv.pdb B;905 1js3.pdb B 1jya.pdb B;906 1jys.pdb B 1k4i.pdb B;907 1k3e.pdb B 1k7w.pdb B;908 1k94.pdb B 1kcg.pdb B;909 1kc7.pdb B 1kgn_2.pdb D;910 1kfi.pdb B 1kj1_2.pdb Q;911 1kk8.pdb B 1knz_2.pdb D;912 1knc.pdb B 1krl_2.pdb C;913 1ktb.pdb B 1kvk.pdb B;914 1kut.pdb B 1l2w_2.pdb D;915 1l0a.pdb B 1l6l_7.pdb N;916 1l7d D 1lfa.pdb B;917 1ldf.pdb B 1llf.pdb B;918 1lm8.pdb C 1lqs.pdb S;919 1lq9.pdb B 1lvm_1.pdb E;920 1ltx.pdb B 1m0l.pdb B;921 1m1l.pdb B 1m4r.pdb B;922 1m3w.pdb B 1m7r.pdb B;923 1m9x C 1mhh_2.pdb D;924 1mg1.pdb B 1mjo.pdb B;925 1miw.pdb B 1mmf_2.pdb E;926 1mo9.pdb B 1mqk.pdb H;927 1mpy.pdb B 1mus.pdb F;928 1mvk J 1mz0.pdb B;929 1my7.pdb B 1n1j.pdb B;930 1n12 D 1n69_1.pdb B;931 1n7h.pdb B 1nbc.pdb B;932 1naw.pdb B 1nek.pdb B;933 1nf3 C 1njh.pdb B;934 1ni4.pdb B 1nlr.pdb B;935 1nkt.pdb B 1no4_1.pdb B;936 1nox.pdb B 1nsi.pdb B;937 1nrj.pdb B 1nuq.pdb B;938 1nvm B 1nxu.pdb B;939 1nxj B 1o1h.pdb B;940 1o0s.pdb B 1o58.pdb B;941 1o5h.pdb B 1o7k.pdb B;942 1o6z.pdb B 1o97.pdb D;943 1oa8.pdb B 1oc2.pdb B;944 1obx.pdb C 1of8.pdb B;945 1oe9.pdb B 1oi4.pdb B;946 1oio.pdb B 1ol0.pdb B;947 1oki.pdb B 1ooe.pdb B;948 1ope.pdb B 1osp.pdb H;949 1oru.pdb B 1ova_2.pdb D;950 1ou0.pdb B 1oyv.pdb B;951 1p0x.pdb B 1p5t.pdb B;952 1p35 B 1p8j_2.pdb F;953 1p9o.pdb B 1pea.pdb B;954 1pdo.pdb B 1pin.pdb C;955 1pgr F 1pl4.pdb B;956 1pm3.pdb B 1psr.pdb B;957 1pqw.pdb B 1pvm.pdb B;958 1pxv C 1q15.pdb C;959 1q0q.pdb B 1q4s.pdb B;960 1q40 D 1q8b.pdb B;961 1q98.pdb B 1qdl.pdb B;962 1qc7.pdb B 1qhx.pdb B;963 1qj8.pdb B 1qlm.pdb B;964 1qks.pdb B 1qo7.pdb B;965 1qmu.pdb B 1qqf.pdb B;966 1qre.pdb B 1qvr.pdb B;967 1qup.pdb B 1qz1.pdb B;968 1qzq.pdb B 1r27_1.pdb B;969 1r1k.pdb A 1r5p.pdb B;970 1r4m F 1r8g.pdb B;971 1r8s.pdb E 1req_1.pdb B;972 1re3 B 1rhg.pdb B;973 1rj9.pdb B 1rmt.pdb B;974 1rmd.pdb B 1rtw.pdb B;975 1rqi.pdb B 1rye.pdb B;976 1rz1 B 1s3e.pdb B;977 1s1q D 1s78_1.pdb C;978 1s7m E 1sby.pdb B;979 1sbb.pdb B 1sei.pdb B;980 1sdd.pdb B 1sgh.pdb B;981 1sh5.pdb B 1slu.pdb B;982 1skz.pdb B 1sqd.pdb B;983 1sqs.pdb B 1su8.pdb B;984 1stz.pdb B 1sxh.pdb D;985 1svx.pdb B 1t0b_2.pdb F;986 1t11.pdb B 1t3u.pdb B;987 1t3l.pdb B 1t6l.pdb B;988 1t70 H 1tab.pdb I;989 1t9i.pdb B 1te1.pdb B;990 1tco.pdb B 1thf.pdb A;991 1tj7.pdb B 1tnr.pdb R;992 1tm0.pdb B 1tt5_1.pdb B;993 1tue L 1twb_2.pdb D;994 1tvn.pdb B 1txy.pdb B;995 1txg.pdb B 1u0e.pdb B;996 1u19.pdb B 1u75.pdb B;997 1u6g.pdb B 1uaa.pdb B;998 1uaz.pdb B 1udu.pdb B;999 1udd.pdb B 1uhv.pdb B;1000 1ufo B 1uld_1.pdb A;1001 1um0.pdb B 1uq5.pdb B;

Random set 2

1 12as.pdb B 1a0c.pdb B;2 1a12.pdb B 1a2k_1.pdb B;3 1a73.pdb B 1a7b.pdb D;4 1aa7.pdb B 1abb.pdb B;5 1adu.pdb B 1ail.pdb B;6 1aoh.pdb B 1apy.pdb B;7 1aro.pdb L 1aua.pdb B;8 1avz.pdb B 1awp.pdb B;9 1ayf.pdb B 1b01.pdb B;10 1b1z.pdb B 1b43.pdb B;11 1b6u.pdb B 1b7y.pdb B;12 1b9c.pdb B 1bbp.pdb B;13 1bgf.pdb B 1bh0.pdb B;14 1bi7.pdb B 1bkf.pdb B;15 1bml.pdb B 1bpl.pdb B;16 1btk.pdb B 1bun.pdb B;17 1bw0.pdb B 1byk.pdb B;18 1c3k.pdb B 1c4p.pdb B;19 1c76.pdb B 1c8b.pdb B;20 1c9o.pdb B 1cby.pdb B;21 1chm.pdb B 1ci6.pdb A;22 1cku.pdb B 1cmc.pdb B;23 1cs1.pdb B 1csg.pdb B;24 1ctt.pdb B 1cv8.pdb B;25 1cxp.pdb C 1d0c.pdb B;26 1d2s.pdb B 1d3y.pdb B;27 1d6r.pdb I 1d8l.pdb B;28 1dd3 B 1dee_2.pdb D;29 1dfn.pdb B 1dhk.pdb B;30 1dj7.pdb B 1dku.pdb B;31 1dov.pdb B 1dqe.pdb B;32 1dqz.pdb B 1duv.pdb H;33 1dyw.pdb B 1dzf.pdb B;34 1e1z.pdb A 1e3m.pdb B;35 1e50 A 1e6u.pdb B;36 1eak C 1ebd.pdb B;37 1ecm.pdb B 1edz.pdb B;38 1eg4.pdb P 1ega.pdb B;39 1ei5.pdb B 1eje.pdb B;40 1eku.pdb B 1emu.pdb B;41 1ern.pdb B 1ete_2.pdb D;42 1ev7.pdb B 1ew6.pdb B;43 1ezg.pdb B 1ezs.pdb B;44 1f0c.pdb B 1f1x.pdb B;45 1f2u.pdb B 1f3h.pdb B;46 1f5v.pdb B 1f6y.pdb B;47 1f8e.pdb B 1fa2.pdb B;48 1fe8 J 1ff9.pdb B;49 1fiu.pdb B 1flk_2.pdb B;50 1fm0.pdb E 1foc.pdb B;51 1fr2.pdb B 1fs0.pdb G;52 1fsg.pdb C 1fui_2.pdb E;53 1g1j.pdb B 1g2y_2.pdb D;54 1g5g E 1g6u.pdb B;55 1g8k F 1g9m.pdb C;56 1gg6.pdb C 1gjo.pdb B;57 1gka.pdb B 1gl4.pdb B;58 1gpm.pdb B 1gpu.pdb B;59 1gr0.pdb B 1gt6.pdb B;60 1gtw.pdb B 1gvf.pdb B;61 1gxc B 1gxr.pdb B;62 1gyg B 1gz6.pdb B;63 1h21.pdb B 1h2s.pdb B;64 1h4p.pdb B 1h5q_3.pdb J;65 1h6o.pdb B 1h80.pdb B;66 1hbx E 1hcn.pdb B;67 1he1 C 1hfe_2.pdb M;68 1hlo.pdb B 1hn4.pdb B;69 1hqr.pdb B 1hsj.pdb B;70 1htr.pdb P 1hw1.pdb B;71 1hyl.pdb B 1hyu.pdb B;72 1i0r.pdb B 1i2m_2.pdb D;73 1i52.pdb B 1i6l.pdb B;74 1i85.pdb B 1iag.pdb B;75 1ibr D 1idp.pdb B;76 1ii7.pdb B 1ik6.pdb B;77 1iom.pdb B 1iro.pdb B;78 1ix2.pdb B 1ixm.pdb B;79 1j1b.pdb B 1j2j.pdb B;80 1j3b.pdb B 1j5w.pdb B;81 1jay.pdb B 1jch.pdb B;82 1jdp.pdb B 1jfi.pdb B;83 1ji7.pdb B 1jjc.pdb B;84 1jkj B 1jl4.pdb B;85 1jlv B 1jmv_2.pdb D;86 1jqp.pdb B 1jro_2.pdb F;87 1js3.pdb B 1juv.pdb B;88 1jys.pdb B 1jzo.pdb B;89 1k3e.pdb B 1k4z_2.pdb B;90 1k5n.pdb B 1k92.pdb B;91 1kc7.pdb B 1kcx.pdb B;92 1kfi.pdb B 1khd_2.pdb C;93 1kk8.pdb B 1kl1.pdb B;94 1knc.pdb B 1ko7_1.pdb B;95 1kor.pdb B 1ksi.pdb B;96 1kut.pdb B 1kxv_1.pdb C;97 1l0a.pdb B 1l3c.pdb B;98 1l7d D 1l8d.pdb B;99 1ldf.pdb B 1lfd_1.pdb B;100 1lj9.pdb B 1llu_1.pdb B;101 1lq9.pdb B 1lrh_2.pdb C;102 1ltx.pdb B 1lwd.pdb B;103 1m1l.pdb B 1m2d.pdb B;104 1m3w.pdb B 1m4z.pdb B;105 1m6d.pdb B 1m93.pdb B;106 1mg1.pdb B 1mhp_1.pdb H;107 1miw.pdb B 1mk2.pdb B;108 1mo9.pdb B 1mou.pdb B;109 1mpy.pdb B 1mr7_1.pdb B;110 1msc.pdb B 1mv8_2.pdb D;111 1my7.pdb B 1mze.pdb B;112 1n12 D 1n26.pdb B;113 1n7h.pdb B 1n7z_2.pdb D;114 1naw.pdb B 1nbq.pdb B;115 1nco.pdb B 1nex_2.pdb D;116 1ni4.pdb B 1nkd.pdb B;117 1nkt.pdb B 1nlt.pdb B;118 1nox.pdb B 1npe.pdb B;119 1nrj.pdb B 1nsn.pdb H;120 1nt2.pdb B 1nvi.pdb E;121 1nxj B 1ny5.pdb B;122 1o0s.pdb B 1o22.pdb B;123 1o5h.pdb B 1o63.pdb B;124 1o6z.pdb B 1o7n.pdb B;125 1o89.pdb B 1o9p.pdb B;126 1obx.pdb C 1ocv.pdb B;127 1oe9.pdb B 1ofu.pdb B;128 1oio.pdb B 1ojh_5.pdb J;129 1oki.pdb B 1olq.pdb B;130 1omw.pdb B 1op3.pdb H;131 1oru.pdb B 1ot6.pdb B;132 1ou0.pdb B 1ovn.pdb B;133 1p0x.pdb B 1p1j.pdb B;134 1p35 B 1p5z.pdb A;135 1p74.pdb B 1p9h.pdb B;136 1pdo.pdb B 1pf5.pdb B;137 1pgr F 1pix.pdb B;138 1pm3.pdb B 1pn2_2.pdb D;139 1pqw.pdb B 1ptq.pdb B;140 1pv1.pdb B 1px8.pdb B;141 1q0q.pdb B 1q18.pdb B;142 1q40 D 1q4u.pdb B;143 1q98.pdb B 1qah.pdb B;144 1qc7.pdb B 1qf8.pdb B;145 1qh3.pdb B 1qiu_2.pdb E;146 1qks.pdb B 1qm4.pdb B;147 1qmu.pdb B 1qoj.pdb B;148 1qre.pdb B 1qsd.pdb B;149 1qup.pdb B 1qw2.pdb B;150 1qx4.pdb B 1qz9.pdb B;151 1r1k.pdb A 1r30.pdb B;152 1r4m F 1r6t.pdb B;153 1r8s.pdb E 1r9f.pdb F;154 1re3 B 1rew.pdb B;155 1rgf.pdb B 1riw.pdb B;156 1rmd.pdb B 1rp3_1.pdb B;157 1rqi.pdb B 1rv1.pdb B;158 1rz1 B 1rzn.pdb B;159 1s1q D 1s3z.pdb B;160 1s5a.pdb B 1s7i.pdb B;161 1sbb.pdb B 1sc3.pdb B;162 1sdd.pdb B 1sf8_2.pdb H;163 1sh5.pdb B 1shy.pdb B;164 1skz.pdb B 1smx.pdb B;165 1sox.pdb B 1sqj.pdb B;166 1stz.pdb B 1suw.pdb B;167 1svx.pdb B 1sy7.pdb B;168 1t11.pdb B 1t1r.pdb B;169 1t3l.pdb B 1t4h.pdb B;170 1t5o D 1t6s.pdb B;171 1t9i.pdb B 1taw.pdb B;172 1tco.pdb B 1te5.pdb B;173 1tj7.pdb B 1tk9.pdb B;174 1tm0.pdb B 1to6.pdb B;175 1tqb.pdb B 1tu1.pdb B;176 1tvn.pdb B 1twi_1.pdb B;177 1txg.pdb B 1tye_3.pdb F;178 1u19.pdb B 1u2g.pdb B;179 1u6g.pdb B 1u7i.pdb B;180 1u8s.pdb B 1uae.pdb B;181 1udd.pdb B 1ue1.pdb B;182 1ufo B 1uiu.pdb B;183 1um0.pdb B 1umy.pdb B;184 1unn.pdb B 1ur5.pdb C;185 1us7.pdb B 1usu.pdb B;186 1uuj.pdb B 1uvj.pdb B;187 1uwk.pdb B 1uyj_2.pdb B;188 1v1p.pdb B 1v2z.pdb B;189 1v4v.pdb B 1v6t.pdb B;190 1v72.pdb B 1v7w.pdb B;191 1vbk.pdb B 1vcn.pdb B;192 1vdw.pdb B 1vet.pdb B;193 1vi0.pdb B 1via.pdb B;194 1vj2.pdb B 1vjp.pdb B;195 1vka.pdb B 1vkp.pdb B;196 1vme.pdb B 1vp4.pdb B;197 1vpz.pdb B 1vr6.pdb B;198 1w07.pdb B 1w23.pdb B;199 1w36 F 1w58.pdb A;200 1w6g.pdb B 1w9q_2.pdb S;201 1who.pdb B 1wjg.pdb B;202 1wmh.pdb B 1wp1_1.pdb B;203 1wud.pdb B 1wv2.pdb B;204 1ww1.pdb B 1wws_4.pdb H;205 1wyi.pdb B 1x2i.pdb B;206 1x8z C 1x9z.pdb B;207 1xcf.pdb B 1xdl_4.pdb Z;208 1xgs.pdb B 1xhn_1.pdb B;209 1xk7 B 1xkz_2.pdb D;210 1xm3.pdb B 1xou.pdb B;211 1xs0 B 1xto.pdb B;212 1xuv B 1xvs.pdb B;213 1y0z.pdb B 1y1l.pdb B;214 1y4m.pdb B 1y6v.pdb B;215 1y7m.pdb B 1y9i.pdb B;216 1ycc.pdb B 1ycy.pdb B;217 1yer.pdb B 1ygy.pdb B;218 1ynr.pdb B 1yp2.pdb B;219 1yrb.pdb B 1ysj.pdb B;220 1yu0.pdb B 1ywq.pdb B;221 1z3e.pdb B 1z5y.pdb E;222 1z84.pdb B 1za3_2.pdb L;223 1zed.pdb B 1zh8.pdb B;224 1zke E 1zme.pdb D;225 1zp6.pdb B 1zr4.pdb B;226 1zvp D 256b.pdb B;227 2a2q.pdb H 2a4k.pdb B;228 2a99.pdb B 2a9u.pdb B;229 2aca.pdb B 2ae8_1.pdb B;230 2af4.pdb D 2aio.pdb B;231 2arh.pdb B 2aua.pdb B;232 2avp.pdb B 2axy_1.pdb B;233 2b0l B 2b3z.pdb B;234 2b5g.pdb B 2b7o.pdb B;235 2b9c.pdb B 2bcg.pdb Y;236 2bgw.pdb B 2bif.pdb B;237 2bky.pdb B 2bo4_1.pdb B;238 2c12 B 2c2i.pdb B;239 2c4n.pdb B 2c62.pdb B;240 2ca1.pdb B 2cg6.pdb B;241 2cn4.pdb B 2csu.pdb B;242 2cvz.pdb B 2cye_2.pdb D;243 2d6y.pdb B 2dek.pdb B;244 2dp9.pdb B 2dw6_1.pdb B;245 2eul B 2ewo_3.pdb H;246 2f1f.pdb B 2f2c.pdb B;247 2f3d.pdb B 2f62.pdb B;248 2f96.pdb B 2fa1.pdb B;249 2fbk.pdb B 2fdo.pdb A;250 2fhq.pdb B 2fiq_1.pdb B;251 2fml.pdb B 2fno_1.pdb B;252 2fp4.pdb B 2fsw.pdb B;253 2fzt.pdb B 2g30.pdb P;254 2g40.pdb B 2g7g.pdb B;255 2gc7 F 2gdg.pdb B;256 2gh1.pdb B 2gia.pdb B;257 2gj7.pdb F 2gqv.pdb B;258 2gyq.pdb B 2h1c.pdb B;259 2h7z.pdb B 2hcb.pdb B;260 2hoe.pdb B 2hqs_4.pdb G;261 2hu7.pdb B 2hyd.pdb B;262 2i4j.pdb B 2icy_1.pdb B;263 2ijd A 2ipr.pdb B;264 2ixc.pdb B 2j7p.pdb B;265 2nlz.pdb B 2nnu.pdb B;266 2nul.pdb B 2nym_1.pdb B;267 2o23.pdb B 2oq1.pdb B;268 2pg2.pdb B 2pjr.pdb B;269 2pva.pdb B 2q4g.pdb W;270 2tnf.pdb B 2vhb.pdb B;271 3cla.pdb B 3eip.pdb B;272 3grs.pdb B 3mag.pdb B;273 4ake.pdb B 4dfr.pdb B;274 4sgb.pdb I 6ldh.pdb B;275 1a12.pdb B 1a3q.pdb B;276 1a2z.pdb B 1a8y.pdb B;277 1a73.pdb B 1adq.pdb L;278 1adu.pdb B 1am7.pdb B;279 1ais.pdb B 1aqu.pdb B;280 1aro.pdb L 1avf_2.pdb J;281 1auo.pdb B 1ay2.pdb B;282 1avz.pdb B 1b0x.pdb B;283 1b1z.pdb B 1b63.pdb B;284 1b49.pdb C 1b8z.pdb B;285 1b9c.pdb B 1bec.pdb B;286 1bdb.pdb B 1bhh.pdb B;287 1bgf.pdb B 1bm9_1.pdb B;288 1bml.pdb B 1bsl.pdb B;289 1bqp B 1bvp_2.pdb 5;290 1bw0.pdb B 1c0p.pdb B;291 1bzq N 1c5e.pdb B;292 1c3k.pdb B 1c9k.pdb B;293 1c9o.pdb B 1cfm.pdb B;294 1cd9.pdb B 1cjx.pdb B;295 1cku.pdb B 1cpb.pdb B;296 1cnz.pdb B 1ct9_2.pdb C;297 1cs1.pdb B 1cx8_1.pdb B;298 1cxp.pdb C 1d2e.pdb B;299 1d0q.pdb B 1d5f.pdb B;300 1d6r.pdb I 1dbt_1.pdb B;301 1d9c.pdb B 1df4.pdb B;302 1dd3 B 1dj0.pdb B;303 1dj7.pdb B 1dn1.pdb B;304 1dl5.pdb B 1dqs.pdb B;305 1dqz.pdb B 1dxy.pdb B;306 1dvp.pdb B 1e19.pdb B;307 1dyw.pdb B 1e4m.pdb A;308 1e50 A 1e9g.pdb B;309 1e7n.pdb B 1ecf.pdb B;310 1ecm.pdb B 1ef8.pdb B;311 1eeq.pdb B 1egw_2.pdb D;312 1eg4.pdb P 1ekq_2.pdb B;313 1eku.pdb B 1ept.pdb A;314 1eo6.pdb B 1euw.pdb B;315 1ev7.pdb B 1ext.pdb B;316 1ewy C 1f02.pdb T;317 1ezg.pdb B 1f2t.pdb B;318 1f2u.pdb B 1f51_2.pdb D;319 1f3u F 1f86.pdb B;320 1f8e.pdb B 1fcd_1.pdb C;321 1fbt.pdb B 1fgu.pdb B;322 1fe8 J 1flt.pdb W;323 1fm0.pdb E 1fqj_1.pdb B;324 1fp1.pdb A 1fs8.pdb B;325 1fsg.pdb C 1fxz.pdb B;326 1fwx D 1g4u.pdb R;327 1g1j.pdb B 1g8e.pdb B;328 1g8k F 1ge7.pdb B;329 1gc1 C 1gk6.pdb A;330 1gka.pdb B 1goi.pdb B;331 1gmv.pdb B 1gqn.pdb B;332 1gpm.pdb B 1gte_1.pdb B;333 1gtw.pdb B 1gwn.pdb C;334 1gvn.pdb B 1gy6.pdb B;335 1gyg B 1h16.pdb B;336 1gzj.pdb B 1h3o_1.pdb B;337 1h21.pdb B 1h6k_2.pdb Y;338 1h6o.pdb B 1h9r.pdb B;339 1h8g.pdb B 1hdh.pdb B;340 1he1 C 1hk7.pdb B;341 1hh2.pdb A 1hnj.pdb B;342 1hlo.pdb B 1htn.pdb B;343 1htr.pdb P 1hxp.pdb B;344 1hw7.pdb B 1hzy.pdb B;345 1i0r.pdb B 1i49.pdb B;346 1i31.pdb P 1i7b.pdb A;347 1i52.pdb B 1ib1_1.pdb B;348 1ibr D 1ihb.pdb B;349 1ied.pdb B 1im3_3.pdb L;350 1iom.pdb B 1itv.pdb B;351 1isy.pdb B 1izm.pdb B;352 1ix2.pdb B 1j31_2.pdb D;353 1j3b.pdb B 1ja3.pdb B;354 1j7n.pdb B 1jdf_1.pdb B;355 1jdp.pdb B 1jhd.pdb B;356 1jg8.pdb B 1jk9_2.pdb D;357 1ji7.pdb B 1jlt.pdb B;358 1jlv B 1jpy_1.pdb B;359 1joc.pdb B 1js1.pdb Y;360 1js3.pdb B 1jxp.pdb B;361 1jvm.pdb B 1k1e_3.pdb J;362 1jys.pdb B 1k5d_1.pdb B;363 1k5n.pdb B 1kam.pdb B;364 1k94.pdb B 1kez_1.pdb B;365 1kfi.pdb B 1kix.pdb C;366 1ki1 D 1klo.pdb B;367 1kk8.pdb B 1kol.pdb B;368 1kor.pdb B 1ku2.pdb B;369 1ktb.pdb B 1kzq.pdb B;370 1l0a.pdb B 1l5r.pdb B;371 1l3p.pdb B 1lc5.pdb B;372 1l7d D 1lid.pdb B;373 1lj9.pdb B 1lom.pdb B;374 1lm8.pdb C 1lt8.pdb B;375 1ltx.pdb B 1lyw_2.pdb D;376 1lxd.pdb B 1m2v.pdb B;377 1m1l.pdb B 1m65.pdb B;378 1m6d.pdb B 1mcw.pdb M;379 1m9x C 1mi3_2.pdb D;380 1miw.pdb B 1ml0.pdb D;381 1mkf.pdb B 1mp4.pdb B;382 1mo9.pdb B 1mrz.pdb B;383 1msc.pdb B 1mxb.pdb B;384 1mvk J 1n0l_1.pdb B;385 1n12 D 1n55.pdb B;386 1n2m E 1n9p.pdb B;387 1n7h.pdb B 1nc7.pdb B;388 1nco.pdb B 1nh2.pdb B;389 1nf3 C 1nkp_1.pdb B;390 1nkt.pdb B 1nn5.pdb B;391 1nme.pdb B 1nqk.pdb B;392 1nox.pdb B 1nt0.pdb G;393 1nt2.pdb B 1nw9.pdb B;394 1nvm B 1nzi.pdb B;395 1o0s.pdb B 1o54.pdb B;396 1o4w.pdb B 1o6e.pdb B;397 1o5h.pdb B 1o84.pdb B;398 1o89.pdb B 1ob9.pdb B;399 1oa8.pdb B 1oe0.pdb B;400 1oe9.pdb B 1oi0.pdb B;401 1og6.pdb B 1ok7.pdb B;402 1oio.pdb B 1oms.pdb B;403 1omw.pdb B 1or4.pdb B;404 1ope.pdb B 1otk.pdb B;405 1ou0.pdb B 1oyn.pdb B;406 1ox0.pdb B 1p2j.pdb I;407 1p0x.pdb B 1p6o.pdb B;408 1p74.pdb B 1pcs.pdb B;409 1p9o.pdb B 1pfo.pdb B;410 1pgr F 1pkq_1.pdb B;411 1pk1 B 1ppr.pdb N;412 1pm3.pdb B 1puf.pdb B;413 1pv1.pdb B 1pzs.pdb B;414 1pxv C 1q3g_2.pdb F;415 1q40 D 1q7e.pdb B;416 1q67.pdb B 1qb2.pdb B;417 1q98.pdb B 1qge.pdb E;418 1qh3.pdb B 1qki_1.pdb B;419 1qj8.pdb B 1qmg_2.pdb D;420 1qmu.pdb B 1qpo_2.pdb D;421 1qoz.pdb B 1qtn.pdb B;422 1qre.pdb B 1qwt.pdb B;423 1qx4.pdb B 1r11.pdb B;424 1qzq.pdb B 1r43.pdb B;425 1r4m F 1r8d.pdb B;426 1r7a.pdb B 1rcu.pdb B;427 1r8s.pdb E 1rg9.pdb B;428 1rgf.pdb B 1rke.pdb B;429 1rj9.pdb B 1rqb.pdb B;430 1rqi.pdb B 1ry9_1.pdb B;431 1rw0.pdb B 1s0p.pdb B;432 1rz1 B 1s4n.pdb B;433 1s5a.pdb B 1s98.pdb B;434 1s7m E 1scj.pdb B;435 1sdd.pdb B 1sg4.pdb B;436 1sfp.pdb B 1sjw.pdb B;437 1sh5.pdb B 1so2_2.pdb D;438 1sox.pdb B 1srq_2.pdb D;439 1sqs.pdb B 1svp.pdb B;440 1svx.pdb B 1t06.pdb B;441 1sz2.pdb B 1t3c.pdb B;442 1t11.pdb B 1t5h.pdb A;443 1t5o D 1t8t.pdb B;444 1t70 H 1tc1.pdb B;445 1tco.pdb B 1th8.pdb B;446 1tf0.pdb B 1tlk.pdb B;447 1tj7.pdb B 1tq8_2.pdb B;448 1tqb.pdb B 1tuw.pdb B;449 1tue L 1tx4.pdb B;450 1txg.pdb B 1u00.pdb P;451 1tz9.pdb B 1u5x.pdb B;452 1u19.pdb B 1u8c.pdb B;453 1u8s.pdb B 1uc2.pdb B;454 1uaz.pdb B 1ufh.pdb B;455 1ufo B 1uku.pdb B;456 1ujq.pdb B 1unh.pdb B;457 1um0.pdb B 1urz_1.pdb B;458 1us7.pdb B 1uth.pdb B;459 1ut7.pdb B 1uw4_1.pdb B;460 1uwk.pdb B 1v05.pdb B;461 1uzb.pdb B 1v3v.pdb B;462 1v1p.pdb B 1v71.pdb B;463 1v72.pdb B 1v96.pdb B;464 1v8c B 1vdd.pdb B;465 1vdw.pdb B 1vh5.pdb B;466 1vg0.pdb B 1viu_1.pdb B;467 1vi0.pdb B 1vk8.pdb B;468 1vka.pdb B 1vlj.pdb B;469 1vl0.pdb B 1vpb.pdb B;470 1vpz.pdb B 1vyt_1.pdb E;471 1vrs E 1w2w_2.pdb F;472 1w07.pdb B 1w68.pdb B;473 1w6g.pdb B 1wdk.pdb B;474 1wa5.pdb B 1wlu.pdb B;475 1wmh.pdb B 1wtd.pdb B;476 1wq1.pdb G 1wvf.pdb B;477 1wud.pdb B 1wy5.pdb B;478 1wyi.pdb B 1x79.pdb B;479 1x3m.pdb B 1xb4_1.pdb B;480 1xcf.pdb B 1xex.pdb B;481 1xdt.pdb R 1xiw_1.pdb B;482 1xgs.pdb B 1xly.pdb B;483 1xm3.pdb B 1xqb.pdb B;484 1xpj.pdb B 1xub.pdb B;485 1xuv B 1xzw.pdb B;486 1xwr.pdb B 1y43.pdb B;487 1y0z.pdb B 1y7b.pdb B;488 1y7m.pdb B 1ybe.pdb B;489 1yak B 1ydw.pdb B;490 1yer.pdb B 1yln.pdb B;491 1yj5 B 1yqf_1.pdb B;492 1ynr.pdb B 1ytf.pdb B;493 1yu0.pdb B 1z0s.pdb B;494 1yxb F 1z72.pdb B;495 1z84.pdb B 1zc3_1.pdb D;496 1zbo.pdb B 1zk4.pdb B;497 1zed.pdb B 1znp_1.pdb B;498 1zp6.pdb B 1zun.pdb B;499 1zrs.pdb B 2a26_1.pdb B;500 2a2q.pdb H 2a6c_1.pdb B;501 2a5t.pdb B 2aal_1.pdb B;502 2a99.pdb B 2aeu.pdb B;503 2af4.pdb D 2amx.pdb B;504 2aj7.pdb B 2avd.pdb B;505 2avp.pdb B 2aze.pdb B;506 2ayo.pdb B 2b59.pdb B;507 2b0l B 2b8t.pdb B;508 2b9c.pdb B 2bex_2.pdb D;509 2bcn.pdb B 2bkr.pdb B;510 2bky.pdb B 2bw3.pdb B;511 2bp3 T 2c35_4.pdb H;512 2c12 B 2c9v.pdb F;513 2ca1.pdb B 2clo.pdb B;514 2ch5 B 2cu6.pdb B;515 2cvz.pdb B 2d29.pdb B;516 2cz1.pdb B 2dft.pdb B;517 2d6y.pdb B 2e2c.pdb B;518 2e2d.pdb C 2f01.pdb B;519 2eul B 2f2l.pdb X;520 2f3d.pdb B 2f6s.pdb B;521 2f69.pdb B 2fb5.pdb B;522 2f96.pdb B 2fef.pdb B;523 2fex B 2fiu.pdb B;524 2fhq.pdb B 2fo7.pdb B;525 2fp4.pdb B 2fuj.pdb B;526 2ftw.pdb B 2g39.pdb B;527 2fzt.pdb B 2g7o.pdb B;528 2g7s.pdb B 2gen.pdb B;529 2gc7 F 2giy.pdb B;530 2gj7.pdb F 2gus.pdb B;531 2gsm B 2h2n.pdb B;532 2gyq.pdb B 2hek.pdb B;533 2hew.pdb A 2hqx.pdb B;534 2hoe.pdb B 2i02.pdb B;535 2i4j.pdb B 2igt.pdb B;536 2idj.pdb B 2it9_1.pdb B;537 2ijd A 2jel.pdb H;538 2jhf.pdb B 2nrf.pdb B;539 2nlz.pdb B 2nz8.pdb B;540 2o23.pdb B 2p3r_1.pdb B;541 2otx.pdb B 2plh.pdb B;542 2pg2.pdb B 2q4n.pdb B;543 2rln.pdb E 2vsg.pdb B;544 2tnf.pdb B 3fru_2.pdb D;545 3grs.pdb B 3sdh.pdb B;546 3pfk.pdb B 4mt2.pdb B;547 4ake.pdb B 6rlx.pdb B;548 1a17.pdb B 1a99_1.pdb B;549 1a3a D 1adu.pdb B;550 1aap.pdb B 1amp.pdb B;551 1af6.pdb B 1ar1.pdb B;552 1ajs.pdb B 1auv.pdb B;553 1as4.pdb B 1ay7.pdb B;554 1auv.pdb B 1b1z.pdb B;555 1ayo.pdb B 1b65_1.pdb B;556 1b25.pdb B 1b9b.pdb B;557 1b4w.pdb B 1bdf.pdb B;558 1b9e.pdb B 1bht.pdb B;559 1bdf.pdb B 1bml.pdb B;560 1bih.pdb B 1bsm.pdb B;561 1bmo.pdb B 1bvy.pdb F;562 1bqu.pdb B 1c02.pdb B;563 1bwn.pdb B 1c5x.pdb A;564 1c02.pdb B 1c9o.pdb B;565 1c7c.pdb B 1cfr.pdb B;566 1ca0 C 1cka.pdb B;567 1ce9.pdb B 1coi.pdb B;568 1cl7.pdb H 1ctf.pdb B;569 1coi.pdb B 1cxp.pdb C;570 1cu1.pdb B 1d2f.pdb B;571 1cxq.pdb B 1d5l.pdb C;572 1d1j.pdb B 1dan.pdb H;573 1d7b.pdb B 1df9.pdb B;574 1dan.pdb H 1dj7.pdb B;575 1dg1.pdb H 1dnl.pdb B;576 1dj8 F 1dqw_1.pdb B;577 1dle.pdb B 1dxg.pdb B;578 1dru.pdb B 1e1h.pdb B;579 1dxg.pdb B 1e50_1.pdb A;580 1e20.pdb B 1e9p.pdb B;581 1e58.pdb B 1ecj_1.pdb B;582 1e85.pdb B 1eer.pdb B;583 1ecs.pdb B 1eh9.pdb B;584 1eer.pdb B 1eku.pdb B;585 1ei6 D 1epx.pdb B;586 1el6.pdb B 1ev4.pdb C;587 1ep3.pdb B 1ex0.pdb B;588 1evj C 1f07.pdb B;589 1ex0.pdb B 1f2u.pdb B;590 1f0k.pdb B 1f59_1.pdb C;591 1f2v.pdb B 1f89.pdb B;592 1f3v.pdb B 1fbv.pdb C;593 1f8f.pdb B 1fi4.pdb B;594 1fbv.pdb C 1fm0.pdb E;595 1fjj.pdb B 1fqk_2.pdb D;596 1fmc.pdb B 1fse_2.pdb F;597 1fp2.pdb B 1fx2.pdb B;598 1fsk K 1g4y.pdb R;599 1fx2.pdb B 1g8k_3.pdb F;600 1g5h D 1ge8.pdb B;601 1g8l.pdb B 1gk9.pdb B;602 1gcj.pdb B 1gmw.pdb B;603 1gkj.pdb B 1gqp.pdb B;604 1gmw.pdb B 1gtw.pdb B;605 1gr7.pdb B 1gwy.pdb B;606 1gu2.pdb B 1gy8.pdb B;607 1gvp.pdb B 1gzs_2.pdb D;608 1gyo.pdb B 1h3y.pdb B;609 1gzs D 1h6o.pdb B;610 1h4r.pdb B 1h9x.pdb B;611 1h6p.pdb B 1hdm.pdb B;612 1h8p.pdb B 1hia_1.pdb B;613 1he7.pdb B 1hoz.pdb B;614 1hia B 1htr.pdb P;615 1hqs.pdb B 1hxx.pdb B;616 1hul.pdb B 1i07.pdb B;617 1hwg.pdb B 1i36.pdb B;618 1i12 C 1i7n.pdb B;619 1i36.pdb B 1ibr_2.pdb D;620 1i86.pdb B 1ihj_1.pdb D;621 1iby B 1inl.pdb B;622 1ig0.pdb B 1itb.pdb B;623 1ips.pdb B 1izn_1.pdb B;624 1itb.pdb B 1j3b.pdb B;625 1j1d E 1jad.pdb B;626 1j3k.pdb B 1jdh.pdb B;627 1j8b.pdb B 1jgs.pdb B;628 1jdw.pdb B 1jke_1.pdb D;629 1jgs.pdb B 1jlv_1.pdb B;630 1jkm.pdb B 1jqj_1.pdb C;631 1jlx.pdb B 1js2.pdb B;632 1jof B 1jw9.pdb D;633 1jsd.pdb B 1k1f_1.pdb B;634 1jw9.pdb D 1k5n.pdb B;635 1k3s.pdb B 1kaw.pdb B;636 1k66.pdb B 1kf6_2.pdb N;637 1ka2.pdb B 1ki9.pdb B;638 1kfu.pdb S 1kmh.pdb B;639 1ki9.pdb B 1kor.pdb B;640 1knq.pdb B 1ku6.pdb B;641 1kpf.pdb B 1kzy.pdb B;642 1ktj.pdb B 1l4d.pdb B;643 1l0o.pdb B 1lck.pdb B;644 1l4d.pdb B 1lj9.pdb B;645 1ldj.pdb B 1lp1.pdb B;646 1lk3 M 1ltq.pdb B;647 1lmb.pdb 4 1lxe.pdb B;648 1lua.pdb B 1m3e_1.pdb B;649 1lxe.pdb B 1m6d.pdb B;650 1m3y B 1mdy_2.pdb D;651 1m6e.pdb A 1mi8.pdb B;652 1ma9.pdb B 1mkk.pdb B;653 1mjf.pdb B 1mp9.pdb B;654 1mkk.pdb B 1msc.pdb B;655 1mq8 B 1mxf.pdb B;656 1msp.pdb B 1n0w.pdb B;657 1mvo.pdb B 1n2s.pdb B;658 1n1b.pdb B 1na6.pdb B;659 1n2s.pdb B 1nco.pdb B;660 1nb5 J 1nh8.pdb B;661 1nd4.pdb B 1nkq_3.pdb F;662 1nf9.pdb B 1nms.pdb B;663 1nkv.pdb B 1nql.pdb B;664 1nms.pdb B 1nt2.pdb B;665 1nrv.pdb B 1nwp.pdb B;666 1nt3.pdb B 1o04_2.pdb F;667 1nvp.pdb B 1o4z_2.pdb D;668 1o0v.pdb B 1o6r.pdb B;669 1o4z D 1o89.pdb B;670 1o75.pdb B 1obb.pdb B;671 1o8b.pdb B 1oe5.pdb B;672 1oaa.pdb B 1ogs.pdb B;673 1oey M 1oke.pdb B;674 1ogs.pdb B 1omw.pdb B;675 1okj D 1or7_2.pdb F;676 1omz.pdb B 1otv.pdb B;677 1oph.pdb B 1oxk_1.pdb B;678 1ou8 B 1p2z.pdb B;679 1oxk B 1p74.pdb B;680 1p5b.pdb B 1pcx.pdb B;681 1p7h M 1pfx.pdb L;682 1pbi.pdb B 1pk8_1.pdb B;683 1pgu.pdb B 1ppv.pdb B;684 1pk8 B 1pv1.pdb B;685 1pqz.pdb B 1pzw.pdb B;686 1pv9.pdb B 1q3o.pdb B;687 1py1 B 1q6o.pdb B;688 1q43 B 1qb4.pdb B;689 1q6o.pdb B 1qh3.pdb B;690 1qd1.pdb B 1qkk.pdb B;691 1qh4 B 1qmh.pdb B;692 1qja.pdb B 1qp1.pdb B;693 1qnn.pdb B 1qtx.pdb B;694 1qp1.pdb B 1qx4.pdb B;695 1quq.pdb B 1r12.pdb B;696 1qx8.pdb B 1r4c_3.pdb F;697 1r0m D 1r7j.pdb B;698 1r4w D 1rcw_2.pdb C;699 1r7j.pdb B 1rgf.pdb B;700 1rec.pdb B 1rkt.pdb B;701 1rgx.pdb B 1rqg.pdb B;702 1rjl.pdb C 1rw6.pdb B;703 1rrm.pdb B 1s12_1.pdb D;704 1rw6.pdb B 1s5a.pdb B;705 1s28 D 1s9j.pdb B;706 1s5u B 1sd4.pdb B;707 1s7o.pdb B 1sfx.pdb B;708 1sdx.pdb E 1sjy.pdb B;709 1sfx.pdb B 1sox.pdb B;710 1slm.pdb B 1sry.pdb B;711 1spp.pdb A 1svv.pdb B;712 1squ.pdb B 1sz6.pdb B;713 1sw6.pdb B 1t3i.pdb B;714 1sz6.pdb B 1t5o_2.pdb D;715 1t3m.pdb B 1t92.pdb B;716 1t62.pdb B 1tc5_1.pdb B;717 1t7r.pdb B 1tfe.pdb B;718 1td2.pdb B 1tll.pdb B;719 1tfe.pdb B 1tqb.pdb B;720 1tmc.pdb B 1tv8.pdb B;721 1tqy F 1tx6_1.pdb B;722 1tuh.pdb B 1tzb.pdb B;723 1txn.pdb B 1u60_2.pdb D;724 1tzb.pdb B 1u8s.pdb B;725 1u6l.pdb B 1uc3_5.pdb J;726 1u8v.pdb B 1ufi_1.pdb B;727 1ub0.pdb B 1uk8.pdb B;728 1ufy.pdb B 1unk.pdb B;729 1uk8.pdb B 1us7.pdb B;730 1up7 B 1uty.pdb B;731 1usc.pdb B 1uwg.pdb L;732 1ut8.pdb B 1uzm.pdb B;733 1uwu.pdb B 1v4e.pdb B;734 1uzm.pdb B 1v72.pdb B;735 1v58 B 1v97.pdb B;736 1v74.pdb B 1vdk.pdb B;737 1v8d.pdb B 1vgy.pdb B;738 1ve1.pdb B 1viv.pdb B;739 1vgy.pdb B 1vka.pdb B;740 1vjg.pdb B 1vlr.pdb B;741 1vkd D 1vpk.pdb B;742 1vl4.pdb B 1vrw.pdb B;743 1vq0.pdb B 1w2y.pdb B;744 1vrw.pdb B 1w6g.pdb B;745 1w3b.pdb B 1wej.pdb H;746 1w6s.pdb B 1wm3.pdb B;747 1wbh.pdb B 1wqw.pdb B;748 1wmi.pdb B 1wvg.pdb B;749 1wqw.pdb B 1wyi.pdb B;750 1ww9.pdb B 1x7d.pdb B;751 1wyu B 1xbr.pdb B;752 1x3z.pdb B 1xe7_2.pdb C;753 1xcg B 1xjl.pdb B;754 1xe7 C 1xm3.pdb B;755 1xka.pdb C 1xrh_4.pdb H;756 1xma.pdb B 1xuu.pdb B;757 1xpm B 1xx6.pdb B;758 1xv2 B 1y44.pdb B;759 1xx6.pdb B 1y7m.pdb B;760 1y5h.pdb B 1ybf.pdb B;761 1y7p C 1yem.pdb B;762 1yav.pdb B 1ykh.pdb B;763 1yf5.pdb A 1yqh.pdb B;764 1ykh.pdb B 1yu0.pdb B;765 1yre D 1z1b.pdb B;766 1yuz.pdb B 1z7a_1.pdb B;767 1yz3.pdb B 1zbr.pdb B;768 1z8u D 1zk8.pdb B;769 1zbr.pdb B 1zp6.pdb B;770 1zkp C 1zup.pdb B;771 1zpd.pdb B 2a2j.pdb B;772 1zru.pdb B 2a5z.pdb B;773 2a2u.pdb B 2ab0.pdb B;774 2a5z.pdb B 2af4.pdb D;775 2adf.pdb H 2apo.pdb B;776 2ag5.pdb B 2avn.pdb B;777 2ajf E 2ayu.pdb B;778 2awi D 2b5a_2.pdb D;779 2ayu.pdb B 2b9c.pdb B;780 2b5i.pdb B 2bf8.pdb B;781 2ba2.pdb B 2bkw.pdb B;782 2bde.pdb B 2bpl_2.pdb C;783 2bll.pdb B 2c42.pdb B;784 2bpl C 2ca1.pdb B;785 2c4v.pdb B 2cm3.pdb B;786 2car.pdb B 2cvd_2.pdb C;787 2ch7.pdb B 2cz4.pdb B;788 2cwk.pdb B 2dg5_1.pdb B;789 2cz4.pdb B 2e2d.pdb C;790 2dpi.pdb D 2f02.pdb B;791 2e7s H 2f36.pdb B;792 2evr.pdb B 2f6k.pdb B;793 2f3x.pdb B 2fbh.pdb B;794 2f6k.pdb B 2fex_1.pdb B;795 2fbl.pdb B 2fl4.pdb B;796 2ff4 E 2fp1.pdb B;797 2fhz.pdb B 2ftx.pdb B;798 2fpn.pdb B 2g3a.pdb B;799 2ftx.pdb B 2g7s.pdb B;800 2g50 F 2gf6_1.pdb B;801 2g82 P 2gj4.pdb B;802 2gci B 2gtd_3.pdb F;803 2gj8 D 2h6f.pdb B;804 2gtd F 2hew.pdb A;805 2h9d D 2hrc.pdb B;806 2hft.pdb B 2i3o_2.pdb D;807 2hox B 2ido_2.pdb D;808 2i52 B 2iuw.pdb B;809 2ido D 2jhf.pdb B;810 2ixp D 2nrh.pdb B;811 2kin.pdb B 2nzu.pdb L;812 2nml.pdb B 2oza.pdb B;813 2o25 D 2por.pdb B;814 2oza.pdb B 2rln.pdb E;815 2q3m.pdb B 2wea.pdb B;816 2rmc.pdb C 3gpd.pdb G;817 2toh.pdb B 3pnp.pdb B;818 3gtu D 4ovo.pdb B;819 3pnp.pdb B 6tmn.pdb A;820 1a2z.pdb B 1a79.pdb B;821 1a73.pdb B 1ab8.pdb B;822 1aa7.pdb B 1aih_1.pdb B;823 1ais.pdb B 1apx_2.pdb D;824 1aoh.pdb B 1au7.pdb B;825 1auo.pdb B 1awd.pdb B;826 1avz.pdb B 1azw.pdb B;827 1ayf.pdb B 1b3t.pdb B;828 1b49.pdb C 1b77.pdb B;829 1b6u.pdb B 1bb9.pdb B;830 1bdb.pdb B 1bgx.pdb L;831 1bgf.pdb B 1bkb.pdb B;832 1bi7.pdb B 1bow.pdb B;833 1bqp B 1bui.pdb B;834 1btk.pdb B 1byi.pdb B;835 1bzq N 1c4k.pdb B;836 1c3k.pdb B 1c7s.pdb B;837 1c76.pdb B 1cbq.pdb B;838 1cd9.pdb B 1ci4.pdb B;839 1chm.pdb B 1clx_2.pdb D;840 1cnz.pdb B 1cse.pdb I;841 1cs1.pdb B 1cun.pdb B;842 1ctt.pdb B 1d02.pdb B;843 1d0q.pdb B 1d3d.pdb B;844 1d2s.pdb B 1d8h_2.pdb C;845 1d9c.pdb B 1ddz.pdb B;846 1dd3 B 1dhf.pdb B;847 1dfn.pdb B 1dkg.pdb B;848 1dl5.pdb B 1dq9.pdb B;849 1dov.pdb B 1dun.pdb B;850 1dvp.pdb B 1dzb_1.pdb X;851 1dyw.pdb B 1e3h.pdb B;852 1e1z.pdb A 1e6j.pdb L;853 1e7n.pdb B 1eb8.pdb B;854 1eak C 1edt.pdb B;855 1eeq.pdb B 1eg7.pdb B;856 1eg4.pdb P 1eja.pdb B;857 1ei5.pdb B 1ems.pdb B;858 1eo6.pdb B 1esc.pdb B;859 1ern.pdb B 1ew3.pdb B;860 1ewy C 1ezj.pdb B;861 1ezg.pdb B 1f1m_2.pdb D;862 1f0c.pdb B 1f3g.pdb B;863 1f3u F 1f6m_2.pdb F;864 1f5v.pdb B 1f9z.pdb B;865 1fbt.pdb B 1ff4.pdb B;866 1fe8 J 1flg.pdb B;867 1fiu.pdb B 1fo1.pdb B;868 1fp1.pdb A 1frt.pdb C;869 1fr2.pdb B 1fua.pdb B;870 1fwx D 1g2q.pdb B;871 1g1j.pdb B 1g60.pdb B;872 1g5g E 1g8x.pdb B;873 1gc1 C 1ghq.pdb B;874 1gg6.pdb C 1gl2.pdb B;875 1gmv.pdb B 1gpr.pdb B;876 1gpm.pdb B 1gt3.pdb B;877 1gr0.pdb B 1gve.pdb B;878 1gvn.pdb B 1gxq.pdb B;879 1gxc B 1gz5.pdb B;880 1gzj.pdb B 1h2k.pdb S;881 1h21.pdb B 1h5b_2.pdb D;882 1h4p.pdb B 1h7z.pdb B;883 1h8g.pdb B 1hcj_2.pdb B;884 1hbx E 1hf2_1.pdb B;885 1hh2.pdb A 1hm9_2.pdb B;886 1hlo.pdb B 1hru.pdb B;887 1hqr.pdb B 1hvv.pdb B;888 1hw7.pdb B 1hyr.pdb A;889 1hyl.pdb B 1i2k.pdb B;890 1i31.pdb P 1i69.pdb B;891 1i52.pdb B 1ia9.pdb B;892 1i85.pdb B 1id1.pdb B;893 1ied.pdb B 1ijy.pdb B;894 1ii7.pdb B 1ire.pdb B;895 1isy.pdb B 1ixc.pdb B;896 1ix2.pdb B 1j2g.pdb B;897 1j1b.pdb B 1j5s.pdb B;898 1j7n.pdb B 1jcd.pdb B;899 1jay.pdb B 1jey.pdb B;900 1jg8.pdb B 1jiw.pdb I;901 1ji7.pdb B 1jl0.pdb B;902 1jkj B 1jmk.pdb O;903 1joc.pdb B 1jrh.pdb H;904 1jqp.pdb B 1jub.pdb B;905 1jvm.pdb B 1jzd.pdb B;906 1jys.pdb B 1k4m.pdb B;907 1k3e.pdb B 1k8r.pdb B;908 1k94.pdb B 1kcm.pdb B;909 1kc7.pdb B 1kgy_1.pdb D;910 1ki1 D 1kko.pdb B;911 1kk8.pdb B 1ko6.pdb C;912 1knc.pdb B 1ksh.pdb B;913 1ktb.pdb B 1kwg.pdb B;914 1kut.pdb B 1l3a.pdb B;915 1l3p.pdb B 1l7v.pdb B;916 1l7d D 1lfb.pdb B;917 1ldf.pdb B 1llm.pdb D;918 1lm8.pdb C 1lqv_2.pdb D;919 1lq9.pdb B 1lw7.pdb B;920 1lxd.pdb B 1m27.pdb B;921 1m1l.pdb B 1m4u.pdb A;922 1m3w.pdb B 1m7y.pdb B;923 1m9x C 1mhm.pdb A;924 1mg1.pdb B 1mju.pdb H;925 1mkf.pdb B 1moq.pdb B;926 1mo9.pdb B 1mr1_2.pdb C;927 1mpy.pdb B 1mv5.pdb B;928 1mvk J 1mzb.pdb B;929 1my7.pdb B 1n1q.pdb B;930 1n2m E 1n7v.pdb B;931 1n7h.pdb B 1nbf_2.pdb C;932 1naw.pdb B 1neu.pdb B;933 1nf3 C 1njr.pdb B;934 1ni4.pdb B 1nls.pdb B;935 1nme.pdb B 1np6.pdb B;936 1nox.pdb B 1nsj.pdb B;937 1nrj.pdb B 1nv7.pdb B;938 1nvm B 1nxz.pdb B;939 1nxj B 1o1x.pdb B;940 1o4w.pdb B 1o5l.pdb B;941 1o5h.pdb B 1o7l.pdb B;942 1o6z.pdb B 1o9l.pdb B;943 1oa8.pdb B 1ocu.pdb B;944 1obx.pdb C 1oft.pdb B;945 1og6.pdb B 1oj5.pdb B;946 1oio.pdb B 1olp.pdb B;947 1oki.pdb B 1ooz.pdb B;948 1ope.pdb B 1osy.pdb B;949 1oru.pdb B 1ovm.pdb B;950 1ox0.pdb B 1p1f.pdb B;951 1p0x.pdb B 1p5v.pdb B;952 1p35 B 1p9e.pdb B;953 1p9o.pdb B 1peq.pdb B;954 1pdo.pdb B 1piw.pdb B;955 1pk1 B 1pn0_1.pdb C;956 1pm3.pdb B 1ptm.pdb B;957 1pqw.pdb B 1pwb.pdb B;958 1pxv C 1q16.pdb B;959 1q0q.pdb B 1q4t.pdb B;960 1q67.pdb B 1qa9_1.pdb A;961 1q98.pdb B 1qdn.pdb B;962 1qc7.pdb B 1qi9.pdb B;963 1qj8.pdb B 1qlw.pdb B;964 1qks.pdb B 1qo8.pdb D;965 1qoz.pdb B 1qrz.pdb B;966 1qre.pdb B 1qvz.pdb B;967 1qup.pdb B 1qz8.pdb B;968 1qzq.pdb B 1r29.pdb B;969 1r1k.pdb A 1r61.pdb B;970 1r7a.pdb B 1r9d.pdb B;971 1r8s.pdb E 1rer.pdb B;972 1re3 B 1ris.pdb B;973 1rj9.pdb B 1ro7.pdb B;974 1rmd.pdb B 1ru0.pdb B;975 1rw0.pdb B 1rzl.pdb B;976 1rz1 B 1s3o.pdb B;977 1s1q D 1s7h_2.pdb D;978 1s7m E 1sc1.pdb B;979 1sbb.pdb B 1sek.pdb B;980 1sfp.pdb B 1shw.pdb B;981 1sh5.pdb B 1smo.pdb B;982 1skz.pdb B 1sqe.pdb B;983 1sqs.pdb B 1sur.pdb B;984 1stz.pdb B 1sy6.pdb H;985 1sz2.pdb B 1t1d.pdb B;986 1t11.pdb B 1t4b.pdb B;987 1t3l.pdb B 1t6n.pdb B;988 1t70 H 1taf.pdb B;989 1t9i.pdb B 1te2.pdb B;990 1tf0.pdb B 1tk5.pdb B;991 1tj7.pdb B 1to3.pdb B;992 1tm0.pdb B 1ttw.pdb B;993 1tue L 1twd.pdb B;994 1tvn.pdb B 1ty0_1.pdb B;995 1tz9.pdb B 1u20.pdb B;996 1u19.pdb B 1u7g.pdb B;997 1u6g.pdb B 1uad_2.pdb D;998 1uaz.pdb B 1udv.pdb B;999 1udd.pdb B 1uis.pdb B;1000 1ujq.pdb B 1umw_1.pdb E;

Random set 3

1 12as.pdb B 1a0g.pdb B;2 1a12.pdb B 1a2x.pdb B;3 1a73.pdb B 1a8l.pdb B;4 1aa7.pdb B 1acb.pdb I;5 1ais.pdb B 1aly.pdb B;6 1aoh.pdb B 1aq0.pdb B;7 1aro.pdb L 1aug.pdb B;8 1avz.pdb B 1ax8.pdb B;9 1ayf.pdb B 1b0n.pdb B;10 1b49.pdb C 1b5f.pdb B;11 1b6u.pdb B 1b8a.pdb B;12 1b9c.pdb B 1bc2.pdb B;13 1bgf.pdb B 1bh9.pdb B;14 1bi7.pdb B 1ble.pdb B;15 1bqp B 1brt.pdb B;16 1btk.pdb B 1bv1.pdb B;17 1bw0.pdb B 1bys.pdb B;18 1c3k.pdb B 1c4t.pdb B;19 1c76.pdb B 1c8u.pdb B;20 1cd9.pdb B 1cf2.pdb R;21 1chm.pdb B 1ci8.pdb B;22 1cku.pdb B 1cmi_2.pdb D;23 1cs1.pdb B 1csh.pdb B;24 1ctt.pdb B 1cvi.pdb B;25 1d0q.pdb B 1d1s.pdb B;26 1d2s.pdb B 1d4t.pdb B;27 1d6r.pdb I 1d8u.pdb B;28 1dd3 B 1dek.pdb B;29 1dfn.pdb B 1dir.pdb B;30 1dl5.pdb B 1dmh.pdb B;31 1dov.pdb B 1dqi.pdb B;32 1dqz.pdb B 1dv1.pdb B;33 1dyw.pdb B 1dzi.pdb B;34 1e1z.pdb A 1e44.pdb B;35 1e7n.pdb B 1e8u.pdb B;36 1eak C 1ebf.pdb B;37 1ecm.pdb B 1ee8.pdb B;38 1eg4.pdb P 1egj.pdb H;39 1ei5.pdb B 1ek9.pdb B;40 1eo6.pdb B 1epa.pdb B;41 1ern.pdb B 1etx.pdb B;42 1ev7.pdb B 1ewk.pdb B;43 1ezg.pdb B 1ezw.pdb B;44 1f0c.pdb B 1f2d_1.pdb B;45 1f3u F 1f45.pdb B;46 1f5v.pdb B 1f74.pdb C;47 1f8e.pdb B 1fak.pdb H;48 1fe8 J 1fg7.pdb B;49 1fiu.pdb B 1flm.pdb B;50 1fp1.pdb A 1fp5.pdb B;51 1fr2.pdb B 1fs1.pdb B;52 1fsg.pdb C 1fuj.pdb B;53 1g1j.pdb B 1g2z.pdb B;54 1g5g E 1g6v.pdb K;55 1gc1 C 1gct.pdb C;56 1gg6.pdb C 1gjw.pdb B;57 1gka.pdb B 1gmj_1.pdb B;58 1gpm.pdb B 1gqg.pdb B;59 1gr0.pdb B 1gt9.pdb 2;60 1gvn.pdb B 1gwb.pdb B;61 1gxc B 1gxs.pdb B;62 1gyg B 1gz7.pdb B;63 1h21.pdb B 1h32.pdb B;64 1h4p.pdb B 1h65_2.pdb C;65 1h8g.pdb B 1h9h.pdb I;66 1hbx E 1hcq_2.pdb F;67 1he1 C 1hg4_2.pdb E;68 1hlo.pdb B 1hne.pdb A;69 1hqr.pdb B 1hsl.pdb B;70 1hw7.pdb B 1hx6.pdb B;71 1hyl.pdb B 1hz4.pdb B;72 1i0r.pdb B 1i2p.pdb B;73 1i52.pdb B 1i6p.pdb B;74 1i85.pdb B 1iam.pdb B;75 1ied.pdb B 1igf.pdb H;76 1ii7.pdb B 1ikn.pdb C;77 1iom.pdb B 1irq.pdb B;78 1ix2.pdb B 1ixs.pdb B;79 1j1b.pdb B 1j2r.pdb B;80 1j7n.pdb B 1j98.pdb B;81 1jay.pdb B 1jck.pdb B;82 1jdp.pdb B 1jfl.pdb B;83 1ji7.pdb B 1jk0.pdb B;84 1jkj B 1jl5.pdb B;85 1joc.pdb B 1jpm.pdb B;86 1jqp.pdb B 1jrr.pdb P;87 1js3.pdb B 1jv1.pdb B;88 1jys.pdb B 1k04.pdb B;89 1k3e.pdb B 1k51.pdb B;90 1k94.pdb B 1ka9.pdb F;91 1kc7.pdb B 1kd8_2.pdb D;92 1kfi.pdb B 1khh.pdb B;93 1kk8.pdb B 1klf_3.pdb F;94 1knc.pdb B 1koa.pdb B;95 1ktb.pdb B 1ktp.pdb B;96 1kut.pdb B 1kyf.pdb P;97 1l0a.pdb B 1l3i_2.pdb B;98 1l7d D 1l9b.pdb M;99 1ldf.pdb B 1lh0.pdb B;100 1lm8.pdb C 1lo7.pdb B;101 1lq9.pdb B 1lsh.pdb B;102 1ltx.pdb B 1lwj.pdb B;103 1m1l.pdb B 1m2o.pdb B;104 1m3w.pdb B 1m56_1.pdb B;105 1m9x C 1mbx_2.pdb D;106 1mg1.pdb B 1mhw_1.pdb C;107 1miw.pdb B 1mk4.pdb B;108 1mo9.pdb B 1mox.pdb B;109 1mpy.pdb B 1mr8.pdb B;110 1mvk J 1mwq.pdb B;111 1my7.pdb B 1mzg.pdb B;112 1n12 D 1n2a.pdb B;113 1n7h.pdb B 1n8z.pdb B;114 1naw.pdb B 1nbw.pdb B;115 1nf3 C 1nfj.pdb B;116 1ni4.pdb B 1nki.pdb B;117 1nkt.pdb B 1nm3.pdb B;118 1nox.pdb B 1nq7.pdb B;119 1nrj.pdb B 1nsw.pdb B;120 1nvm B 1nw1.pdb B;121 1nxj B 1nyr.pdb B;122 1o0s.pdb B 1o26.pdb B;123 1o5h.pdb B 1o69.pdb B;124 1o6z.pdb B 1o7z.pdb B;125 1oa8.pdb B 1oao.pdb B;126 1obx.pdb C 1ocy.pdb B;127 1oe9.pdb B 1ofz.pdb B;128 1oio.pdb B 1ojr.pdb B;129 1oki.pdb B 1ols.pdb B;130 1ope.pdb B 1oqz.pdb B;131 1oru.pdb B 1otg.pdb B;132 1ou0.pdb B 1ovo_2.pdb B;133 1p0x.pdb B 1p22.pdb B;134 1p35 B 1p65.pdb B;135 1p9o.pdb B 1pc3_2.pdb B;136 1pdo.pdb B 1pfb.pdb B;137 1pgr F 1pj5.pdb B;138 1pm3.pdb B 1pnv.pdb B;139 1pqw.pdb B 1ptu.pdb B;140 1pxv C 1pyg.pdb B;141 1q0q.pdb B 1q2h_1.pdb B;142 1q40 D 1q5n.pdb B;143 1q98.pdb B 1qak.pdb B;144 1qc7.pdb B 1qfh.pdb B;145 1qj8.pdb B 1qjs.pdb B;146 1qks.pdb B 1qma.pdb B;147 1qmu.pdb B 1qou.pdb B;148 1qre.pdb B 1qsg_2.pdb F;149 1qup.pdb B 1qwg.pdb B;150 1qzq.pdb B 1r0r.pdb I;151 1r1k.pdb A 1r31.pdb B;152 1r4m F 1r6u.pdb B;153 1r8s.pdb E 1rcb.pdb B;154 1re3 B 1rfy_1.pdb B;155 1rj9.pdb B 1rk8.pdb B;156 1rmd.pdb B 1rq0.pdb B;157 1rqi.pdb B 1rv3.pdb B;158 1rz1 B 1rzo.pdb B;159 1s1q D 1s4c_2.pdb C;160 1s7m E 1s7z.pdb B;161 1sbb.pdb B 1sc6.pdb B;162 1sdd.pdb B 1sff.pdb B;163 1sh5.pdb B 1sj1.pdb B;164 1skz.pdb B 1snd.pdb B;165 1sqs.pdb B 1sr7.pdb B;166 1stz.pdb B 1sv0_2.pdb D;167 1svx.pdb B 1syx_1.pdb B;168 1t11.pdb B 1t1v.pdb B;169 1t3l.pdb B 1t56.pdb B;170 1t70 H 1t83.pdb B;171 1t9i.pdb B 1tbu.pdb B;172 1tco.pdb B 1ted_1.pdb B;173 1tj7.pdb B 1tlf.pdb B;174 1tm0.pdb B 1to9.pdb B;175 1tue L 1tul.pdb B;176 1tvn.pdb B 1twu.pdb B;177 1txg.pdb B 1tyx.pdb B;178 1u19.pdb B 1u5h.pdb B;179 1u6g.pdb B 1u7n.pdb B;180 1uaz.pdb B 1ub9.pdb B;181 1udd.pdb B 1ueh.pdb B;182 1ufo B 1uix.pdb B;183 1um0.pdb B 1un6_2.pdb D;184 1unn.pdb B 1urh.pdb B;185 1ut7.pdb B 1utc.pdb B;186 1uuj.pdb B 1uvq.pdb B;187 1uwk.pdb B 1uyt_1.pdb B;188 1v1p.pdb B 1v37.pdb B;189 1v4v.pdb B 1v6z.pdb B;190 1v8c B 1v8p_2.pdb F;191 1vbk.pdb B 1vco.pdb B;192 1vdw.pdb B 1vf6_1.pdb C;193 1vi0.pdb B 1vim.pdb B;194 1vj2.pdb B 1vjq.pdb B;195 1vl0.pdb B 1vla_2.pdb D;196 1vme.pdb B 1vp6.pdb C;197 1vpz.pdb B 1vr7.pdb B;198 1w07.pdb B 1w25.pdb B;199 1w36 F 1w5r.pdb B;200 1wa5.pdb B 1wc1_2.pdb C;201 1who.pdb B 1wlg.pdb B;202 1wmh.pdb B 1wpn.pdb B;203 1wud.pdb B 1wv8.pdb B;204 1ww1.pdb B 1www.pdb W;205 1x3m.pdb B 1x6v.pdb A;206 1x8z C 1xar.pdb B;207 1xcf.pdb B 1xdp.pdb B;208 1xgs.pdb B 1xi3.pdb B;209 1xk7 B 1xl3_2.pdb D;210 1xpj.pdb B 1xq4.pdb B;211 1xs0 B 1xtt.pdb B;212 1xuv B 1xw8.pdb B;213 1y0z.pdb B 1y1o.pdb B;214 1y4m.pdb B 1y6x.pdb B;215 1yak B 1yb2.pdb B;216 1ycc.pdb B 1yde_2.pdb F;217 1yer.pdb B 1yhc.pdb B;218 1ynr.pdb B 1ypq.pdb B;219 1yrb.pdb B 1ysr.pdb B;220 1yxb F 1z0j.pdb B;221 1z3e.pdb B 1z63.pdb B;222 1z84.pdb B 1zai.pdb B;223 1zed.pdb B 1zii.pdb B;224 1zke E 1zmt.pdb B;225 1zrs.pdb B 1ztc.pdb B;226 1zvp D 2a15.pdb B;227 2a2q.pdb H 2a4x.pdb B;228 2a99.pdb B 2aa4.pdb B;229 2aca.pdb B 2aeb_2.pdb B;230 2aj7.pdb B 2ako_1.pdb B;231 2arh.pdb B 2auw.pdb B;232 2avp.pdb B 2ayl.pdb B;233 2b0l B 2b48.pdb B;234 2b5g.pdb B 2b82.pdb B;235 2bcn.pdb B 2bdt.pdb B;236 2bgw.pdb B 2bj7.pdb B;237 2bky.pdb B 2bo9.pdb B;238 2c12 B 2c2l_1.pdb E;239 2c4n.pdb B 2c7n_4.pdb H;240 2ch5 B 2cjt_2.pdb D;241 2cn4.pdb B 2ctz.pdb B;242 2cz1.pdb B 2d13_2.pdb D;243 2d6y.pdb B 2dex.pdb A;244 2dp9.pdb B 2e2a.pdb B;245 2eul B 2ey4_1.pdb E;246 2f1f.pdb B 2f2e.pdb B;247 2f69.pdb B 2f6q.pdb B;248 2f96.pdb B 2fb1_1.pdb B;249 2fbk.pdb B 2fe1.pdb B;250 2fhq.pdb B 2fit.pdb B;251 2fml.pdb B 2fnu.pdb B;252 2ftw.pdb B 2fue.pdb B;253 2fzt.pdb B 2g38_1.pdb B;254 2g40.pdb B 2g7l.pdb B;255 2gc7 F 2gec.pdb B;256 2gh1.pdb B 2gib.pdb B;257 2gsm B 2gup.pdb B;258 2gyq.pdb B 2h26.pdb B;259 2h7z.pdb B 2hck.pdb B;260 2hoe.pdb B 2hqv.pdb B;261 2hu7.pdb B 2hzb_2.pdb D;262 2idj.pdb B 2ifg.pdb B;263 2ijd A 2iqq.pdb B;264 2ixc.pdb B 2j88.pdb H;265 2nlz.pdb B 2npt_2.pdb C;266 2nul.pdb B 2nyr.pdb B;267 2otx.pdb B 2p2l.pdb B;268 2pg2.pdb B 2pk8.pdb B;269 2pva.pdb B 2q4i.pdb B;270 2tnf.pdb B 2vir.pdb B;271 3cla.pdb B 3fap.pdb B;272 3pfk.pdb B 3pvi.pdb B;273 4ake.pdb B 4hb1.pdb B;274 4sgb.pdb I 6q21.pdb B;275 1a12.pdb B 1a4i.pdb B;276 1a2z.pdb B 1a99_1.pdb B;277 1aa7.pdb B 1ahs.pdb B;278 1adu.pdb B 1amp.pdb B;279 1ais.pdb B 1ar1.pdb B;280 1aro.pdb L 1avq.pdb B;281 1auo.pdb B 1ay7.pdb B;282 1ayf.pdb B 1b34.pdb B;283 1b1z.pdb B 1b65_1.pdb B;284 1b49.pdb C 1b9b.pdb B;285 1b9c.pdb B 1bf3.pdb B;286 1bdb.pdb B 1bht.pdb B;287 1bi7.pdb B 1bo1.pdb B;288 1bml.pdb B 1bsm.pdb B;289 1bqp B 1bvy.pdb F;290 1bw0.pdb B 1c1y.pdb B;291 1bzq N 1c5x.pdb A;292 1c76.pdb B 1cbl.pdb B;293 1c9o.pdb B 1cfr.pdb B;294 1cd9.pdb B 1cka.pdb B;295 1cku.pdb B 1cq3.pdb B;296 1cnz.pdb B 1ctf.pdb B;297 1ctt.pdb B 1cyw.pdb B;298 1cxp.pdb C 1d2f.pdb B;299 1d0q.pdb B 1d5l.pdb C;300 1d6r.pdb I 1dc1.pdb B;301 1d9c.pdb B 1df9.pdb B;302 1dfn.pdb B 1dk8.pdb B;303 1dj7.pdb B 1dnl.pdb B;304 1dl5.pdb B 1dqw_1.pdb B;305 1dqz.pdb B 1dy7.pdb B;306 1dvp.pdb B 1e1h.pdb B;307 1e1z.pdb A 1e65.pdb B;308 1e50 A 1e9p.pdb B;309 1e7n.pdb B 1ecj_1.pdb B;310 1ecm.pdb B 1efa_1.pdb B;311 1eeq.pdb B 1eh9.pdb B;312 1ei5.pdb B 1elw_1.pdb C;313 1eku.pdb B 1epx.pdb B;314 1eo6.pdb B 1ev4.pdb C;315 1ev7.pdb B 1eye.pdb B;316 1ewy C 1f07.pdb B;317 1f0c.pdb B 1f39.pdb B;318 1f2u.pdb B 1f59_1.pdb C;319 1f3u F 1f89.pdb B;320 1f8e.pdb B 1fcg.pdb B;321 1fbt.pdb B 1fi4.pdb B;322 1fiu.pdb B 1fns.pdb H;323 1fm0.pdb E 1fqk_2.pdb D;324 1fp1.pdb A 1fse_2.pdb F;325 1fsg.pdb C 1fyh_2.pdb E;326 1fwx D 1g4y.pdb R;327 1g5g E 1g8s.pdb B;328 1g8k F 1ge8.pdb B;329 1gc1 C 1gk9.pdb B;330 1gka.pdb B 1got.pdb B;331 1gmv.pdb B 1gqp.pdb B;332 1gr0.pdb B 1gud.pdb B;333 1gtw.pdb B 1gwy.pdb B;334 1gvn.pdb B 1gy8.pdb B;335 1gyg B 1h1o.pdb B;336 1gzj.pdb B 1h3y.pdb B;337 1h4p.pdb B 1h7b.pdb B;338 1h6o.pdb B 1h9x.pdb B;339 1h8g.pdb B 1hdm.pdb B;340 1he1 C 1hkq.pdb B;341 1hh2.pdb A 1hoz.pdb B;342 1hqr.pdb B 1hux.pdb B;343 1htr.pdb P 1hxx.pdb B;344 1hw7.pdb B 1i07.pdb B;345 1i0r.pdb B 1i4d.pdb B;346 1i31.pdb P 1i7n.pdb B;347 1i85.pdb B 1icj.pdb B;348 1ibr D 1ihj_1.pdb D;349 1ied.pdb B 1inl.pdb B;350 1iom.pdb B 1iu8.pdb B;351 1isy.pdb B 1izn_1.pdb B;352 1j1b.pdb B 1j4n.pdb B;353 1j3b.pdb B 1jad.pdb B;354 1j7n.pdb B 1jdh.pdb B;355 1jdp.pdb B 1jhf.pdb B;356 1jg8.pdb B 1jke_1.pdb D;357 1jkj B 1jma.pdb B;358 1jlv B 1jqj_1.pdb C;359 1joc.pdb B 1js2.pdb B;360 1js3.pdb B 1jxz.pdb B;361 1jvm.pdb B 1k1f_1.pdb B;362 1k3e.pdb B 1k6y.pdb B;363 1k5n.pdb B 1kaw.pdb B;364 1k94.pdb B 1kf6_2.pdb N;365 1kfi.pdb B 1kiy.pdb B;366 1ki1 D 1kmh.pdb B;367 1knc.pdb B 1kqp.pdb B;368 1kor.pdb B 1ku6.pdb B;369 1ktb.pdb B 1kzy.pdb B;370 1l0a.pdb B 1l5s.pdb B;371 1l3p.pdb B 1lck.pdb B;372 1ldf.pdb B 1ll2.pdb B;373 1lj9.pdb B 1lp1.pdb B;374 1lm8.pdb C 1ltq.pdb B;375 1ltx.pdb B 1m0d_2.pdb D;376 1lxd.pdb B 1m3e_1.pdb B;377 1m3w.pdb B 1m7g_1.pdb B;378 1m6d.pdb B 1mdy_2.pdb D;379 1m9x C 1mi8.pdb B;380 1miw.pdb B 1mld_1.pdb B;381 1mkf.pdb B 1mp9.pdb B;382 1mpy.pdb B 1mun.pdb B;383 1msc.pdb B 1mxf.pdb B;384 1mvk J 1n0w.pdb B;385 1n12 D 1n57.pdb B;386 1n2m E 1na6.pdb B;387 1naw.pdb B 1ne8.pdb B;388 1nco.pdb B 1nh8.pdb B;389 1nf3 C 1nkq_3.pdb F;390 1nkt.pdb B 1nnw.pdb B;391 1nme.pdb B 1nql.pdb B;392 1nrj.pdb B 1nul.pdb B;393 1nt2.pdb B 1nwp.pdb B;394 1nvm B 1o04_2.pdb F;395 1o0s.pdb B 1o57_2.pdb D;396 1o4w.pdb B 1o6r.pdb B;397 1o6z.pdb B 1o94_3.pdb F;398 1o89.pdb B 1obb.pdb B;399 1oa8.pdb B 1oe5.pdb B;400 1oe9.pdb B 1oi2.pdb B;401 1og6.pdb B 1oke.pdb B;402 1oki.pdb B 1oo0.pdb B;403 1omw.pdb B 1or7_2.pdb F;404 1ope.pdb B 1otv.pdb B;405 1ou0.pdb B 1oys.pdb B;406 1ox0.pdb B 1p2z.pdb B;407 1p35 B 1p80.pdb B;408 1p74.pdb B 1pcx.pdb B;409 1p9o.pdb B 1pfx.pdb L;410 1pgr F 1pkv.pdb B;411 1pk1 B 1ppv.pdb B;412 1pqw.pdb B 1pvh_1.pdb B;413 1pv1.pdb B 1pzw.pdb B;414 1pxv C 1q3o.pdb B;415 1q40 D 1q7l_2.pdb D;416 1q67.pdb B 1qb4.pdb B;417 1qc7.pdb B 1qhh.pdb B;418 1qh3.pdb B 1qkk.pdb B;419 1qj8.pdb B 1qmh.pdb B;420 1qmu.pdb B 1qq5.pdb B;421 1qoz.pdb B 1qtx.pdb B;422 1qup.pdb B 1qyn.pdb B;423 1qx4.pdb B 1r12.pdb B;424 1qzq.pdb B 1r4c_3.pdb F;425 1r4m F 1r8e.pdb D;426 1r7a.pdb B 1rcw_2.pdb C;427 1re3 B 1rhc.pdb B;428 1rgf.pdb B 1rkt.pdb B;429 1rj9.pdb B 1rqg.pdb B;430 1rqi.pdb B 1rya.pdb B;431 1rw0.pdb B 1s12_1.pdb D;432 1s1q D 1s70.pdb B;433 1s5a.pdb B 1s9j.pdb B;434 1s7m E 1sd4.pdb B;435 1sdd.pdb B 1sg9_1.pdb C;436 1sfp.pdb B 1sjy.pdb B;437 1skz.pdb B 1sq5_2.pdb B;438 1sox.pdb B 1sry.pdb B;439 1sqs.pdb B 1svv.pdb B;440 1svx.pdb B 1t08.pdb B;441 1sz2.pdb B 1t3i.pdb B;442 1t3l.pdb B 1t6g_2.pdb D;443 1t5o D 1t92.pdb B;444 1t70 H 1tc5_1.pdb B;445 1tco.pdb B 1the.pdb B;446 1tf0.pdb B 1tll.pdb B;447 1tm0.pdb B 1tsr.pdb B;448 1tqb.pdb B 1tv8.pdb B;449 1tue L 1tx6_1.pdb B;450 1txg.pdb B 1u07.pdb B;451 1tz9.pdb B 1u60_2.pdb D;452 1u6g.pdb B 1u9y.pdb B;453 1u8s.pdb B 1uc3_5.pdb J;454 1uaz.pdb B 1ufi_1.pdb B;455 1ufo B 1ukw.pdb B;456 1ujq.pdb B 1unk.pdb B;457 1unn.pdb B 1usl_3.pdb E;458 1us7.pdb B 1uty.pdb B;459 1ut7.pdb B 1uwg.pdb L;460 1uwk.pdb B 1v0d.pdb B;461 1uzb.pdb B 1v4e.pdb B;462 1v4v.pdb B 1v7m_1.pdb H;463 1v72.pdb B 1v97.pdb B;464 1v8c B 1vdk.pdb B;465 1vdw.pdb B 1vh6.pdb B;466 1vg0.pdb B 1viv.pdb B;467 1vj2.pdb B 1vkj.pdb B;468 1vka.pdb B 1vlr.pdb B;469 1vl0.pdb B 1vpk.pdb B;470 1vpz.pdb B 1vz0_4.pdb F;471 1vrs E 1w2y.pdb B;472 1w36 F 1w8i.pdb B;473 1w6g.pdb B 1wej.pdb H;474 1wa5.pdb B 1wm3.pdb B;475 1wmh.pdb B 1wtl.pdb B;476 1wq1.pdb G 1wvg.pdb B;477 1ww1.pdb B 1wz3.pdb B;478 1wyi.pdb B 1x7d.pdb B;479 1x3m.pdb B 1xbr.pdb B;480 1xcf.pdb B 1xff.pdb B;481 1xdt.pdb R 1xjl.pdb B;482 1xk7 B 1xnx.pdb B;483 1xm3.pdb B 1xrh_4.pdb H;484 1xpj.pdb B 1xuu.pdb B;485 1xuv B 1y0b_2.pdb D;486 1xwr.pdb B 1y44.pdb B;487 1y4m.pdb B 1y88.pdb B;488 1y7m.pdb B 1ybf.pdb B;489 1yak B 1yem.pdb B;490 1yer.pdb B 1ylq.pdb B;491 1yj5 B 1yqh.pdb B;492 1yrb.pdb B 1yvh.pdb B;493 1yu0.pdb B 1z1b.pdb B;494 1yxb F 1z7a_1.pdb B;495 1z84.pdb B 1zc6.pdb B;496 1zbo.pdb B 1zk8.pdb B;497 1zke E 1zq1.pdb B;498 1zp6.pdb B 1zup.pdb B;499 1zrs.pdb B 2a2j.pdb B;500 2a2q.pdb H 2a6n.pdb B;501 2a5t.pdb B 2ab0.pdb B;502 2aca.pdb B 2ahu.pdb B;503 2af4.pdb D 2apo.pdb B;504 2aj7.pdb B 2avn.pdb B;505 2avp.pdb B 2b06.pdb B;506 2ayo.pdb B 2b5a_2.pdb D;507 2b5g.pdb B 2bb2.pdb B;508 2b9c.pdb B 2bf8.pdb B;509 2bcn.pdb B 2bkw.pdb B;510 2bky.pdb B 2bw4.pdb B;511 2bp3 T 2c42.pdb B;512 2c4n.pdb B 2cb5.pdb B;513 2ca1.pdb B 2cm3.pdb B;514 2ch5 B 2cvd_2.pdb C;515 2cvz.pdb B 2d32.pdb B;516 2cz1.pdb B 2dg5_1.pdb B;517 2dp9.pdb B 2ejn.pdb B;518 2e2d.pdb C 2f02.pdb B;519 2eul B 2f36.pdb B;520 2f3d.pdb B 2f6u.pdb B;521 2f69.pdb B 2fbh.pdb B;522 2fbk.pdb B 2ffg.pdb B;523 2fex B 2fl4.pdb B;524 2fhq.pdb B 2fp1.pdb B;525 2fp4.pdb B 2fun_1.pdb B;526 2ftw.pdb B 2g3a.pdb B;527 2g40.pdb B 2ga1.pdb B;528 2g7s.pdb B 2gf6_1.pdb B;529 2gc7 F 2gj4.pdb B;530 2gj7.pdb F 2gv8.pdb B;531 2gsm B 2h6f.pdb B;532 2h7z.pdb B 2hiq.pdb B;533 2hew.pdb A 2hrc.pdb B;534 2hoe.pdb B 2i3o_2.pdb D;535 2i4j.pdb B 2iid_2.pdb D;536 2idj.pdb B 2iuw.pdb B;537 2ixc.pdb B 2mlt.pdb B;538 2jhf.pdb B 2nrh.pdb B;539 2nlz.pdb B 2nzu.pdb L;540 2o23.pdb B 2p54.pdb B;541 2otx.pdb B 2por.pdb B;542 2pva.pdb B 2scu.pdb B;543 2rln.pdb E 2wea.pdb B;544 2tnf.pdb B 3gpd.pdb G;545 3grs.pdb B 3sic.pdb I;546 3pfk.pdb B 4ovo.pdb B;547 137l.pdb B 1a5t.pdb B;548 1a17.pdb B 1a9n_2.pdb D;549 1a3a D 1af6.pdb B;550 1aap.pdb B 1an7.pdb B;551 1af6.pdb B 1aro.pdb L;552 1aox.pdb B 1avv.pdb B;553 1as4.pdb B 1ay9.pdb B;554 1auv.pdb B 1b25.pdb B;555 1ayo.pdb B 1b6c_3.pdb F;556 1b25.pdb B 1b9c.pdb B;557 1b74.pdb B 1bg1.pdb D;558 1b9e.pdb B 1bi2.pdb B;559 1bdf.pdb B 1bmo.pdb B;560 1bih.pdb B 1bt6.pdb B;561 1bmo.pdb B 1bw0.pdb B;562 1btn.pdb B 1c2b.pdb B;563 1bwn.pdb B 1c6o.pdb B;564 1c02.pdb B 1ca0_1.pdb C;565 1c7c.pdb B 1cg2.pdb B;566 1ca0 C 1cku.pdb B;567 1cho.pdb I 1cqk.pdb B;568 1cl7.pdb H 1ctn.pdb B;569 1coi.pdb B 1cxq.pdb B;570 1cu1.pdb B 1d2g.pdb B;571 1cxq.pdb B 1d6r.pdb I;572 1d2z D 1dce_2.pdb D;573 1d7b.pdb B 1dfm.pdb B;574 1dan.pdb H 1dj8_3.pdb F;575 1dg1.pdb H 1doh.pdb B;576 1dj8 F 1dqz.pdb B;577 1dpg.pdb B 1dy9_1.pdb C;578 1dru.pdb B 1e1o.pdb B;579 1dxg.pdb B 1e58.pdb B;580 1e20.pdb B 1eag.pdb B;581 1e58.pdb B 1ecm.pdb B;582 1ear.pdb B 1efn.pdb B;583 1ecs.pdb B 1ehi.pdb B;584 1eer.pdb B 1el6.pdb B;585 1ei6 D 1eqr.pdb B;586 1el6.pdb B 1ev7.pdb B;587 1erv.pdb B 1eys.pdb L;588 1evj C 1f08.pdb B;589 1ex0.pdb B 1f2v.pdb B;590 1f0k.pdb B 1f5m.pdb B;591 1f2v.pdb B 1f8e.pdb B;592 1f60.pdb B 1fd3_1.pdb C;593 1f8f.pdb B 1fic.pdb B;594 1fbv.pdb C 1fmc.pdb B;595 1fjj.pdb B 1fqt.pdb B;596 1fmc.pdb B 1fsg.pdb C;597 1fr8.pdb B 1fzd_2.pdb F;598 1fsk K 1g5c_2.pdb D;599 1fx2.pdb B 1g8l.pdb B;600 1g5h D 1gfl.pdb B;601 1g8l.pdb B 1gka.pdb B;602 1ggx.pdb B 1gp9.pdb B;603 1gkj.pdb B 1gqy.pdb B;604 1gmw.pdb B 1gu2.pdb B;605 1gr7.pdb B 1gx4.pdb B;606 1gu2.pdb B 1gyg_2.pdb B;607 1gxd C 1h1v.pdb G;608 1gyo.pdb B 1h4g.pdb B;609 1gzs D 1h6p.pdb B;610 1h4r.pdb B 1ha0.pdb B;611 1h6p.pdb B 1he1_1.pdb C;612 1hc7 B 1hl6.pdb B;613 1he7.pdb B 1hpc.pdb B;614 1hia B 1hul.pdb B;615 1hqs.pdb B 1hxy.pdb B;616 1hul.pdb B 1i0r.pdb B;617 1hyn S 1i4j.pdb B;618 1i12 C 1i7w.pdb B;619 1i36.pdb B 1iby_1.pdb B;620 1i86.pdb B 1ihk.pdb B;621 1iby B 1iom.pdb B;622 1iil E 1iv3_1.pdb B;623 1ips.pdb B 1j0h.pdb B;624 1itb.pdb B 1j3k.pdb B;625 1j1d E 1jaf.pdb B;626 1j3k.pdb B 1jdp.pdb B;627 1jb7.pdb B 1jhg.pdb B;628 1jdw.pdb B 1jkg.pdb B;629 1jgs.pdb B 1jlx.pdb B;630 1jkm.pdb B 1jql.pdb B;631 1jlx.pdb B 1js3.pdb B;632 1jr7.pdb B 1jya.pdb B;633 1jsd.pdb B 1k2f.pdb B;634 1jw9.pdb D 1k66.pdb B;635 1k3s.pdb B 1kb5.pdb L;636 1k66.pdb B 1kfi.pdb B;637 1kcf.pdb B 1kj1_2.pdb Q;638 1kfu.pdb S 1kmi.pdb Z;639 1ki9.pdb B 1kpf.pdb B;640 1knq.pdb B 1ku7.pdb D;641 1kpf.pdb B 1l0a.pdb B;642 1kvd D 1l6l_7.pdb N;643 1l0o.pdb B 1ldd.pdb B;644 1l4d.pdb B 1lk3_2.pdb M;645 1ldj.pdb B 1lpb.pdb B;646 1lk3 M 1ltx.pdb B;647 1lql D 1m0l.pdb B;648 1lua.pdb B 1m3k.pdb B;649 1lxe.pdb B 1m6e.pdb A;650 1m3y B 1me8.pdb B;651 1m6e.pdb A 1miw.pdb B;652 1mh9.pdb B 1mmf_2.pdb E;653 1mjf.pdb B 1mpx.pdb B;654 1mkk.pdb B 1msp.pdb B;655 1mq8 B 1mxi.pdb B;656 1msp.pdb B 1n12_2.pdb D;657 1myl B 1n69_1.pdb B;658 1n1b.pdb B 1na8.pdb B;659 1n2s.pdb B 1nd4.pdb B;660 1nb5 J 1nhk.pdb L;661 1nd4.pdb B 1nkt.pdb B;662 1ni5.pdb B 1no4_1.pdb B;663 1nkv.pdb B 1nri.pdb B;664 1nms.pdb B 1nt3.pdb B;665 1nrv.pdb B 1nww.pdb B;666 1nt3.pdb B 1o0s.pdb B;667 1nxm.pdb B 1o58.pdb B;668 1o0v.pdb B 1o6u.pdb C;669 1o4z D 1o8b.pdb B;670 1o75.pdb B 1obf.pdb P;671 1o8b.pdb B 1oe9.pdb B;672 1oc0.pdb B 1oi4.pdb B;673 1oey M 1okh.pdb B;674 1ogs.pdb B 1omz.pdb B;675 1okj D 1orr.pdb B;676 1omz.pdb B 1ou0.pdb B;677 1orv.pdb B 1oyv.pdb B;678 1ou8 B 1p32.pdb B;679 1oxk B 1p7h_1.pdb M;680 1p5b.pdb B 1pdg_2.pdb C;681 1p7h M 1pgr_2.pdb F;682 1pe1.pdb B 1pl4.pdb B;683 1pgu.pdb B 1pqh.pdb B;684 1pk8 B 1pv9.pdb B;685 1pqz.pdb B 1q08.pdb B;686 1pv9.pdb B 1q40_2.pdb D;687 1q14.pdb B 1q8b.pdb B;688 1q43 B 1qbk.pdb C;689 1q6o.pdb B 1qh4_1.pdb B;690 1qd1.pdb B 1qkq.pdb B;691 1qh4 B 1qmu.pdb B;692 1qkz.pdb H 1qqf.pdb B;693 1qnn.pdb B 1qu9.pdb B;694 1qp1.pdb B 1qx8.pdb B;695 1quq.pdb B 1r17_2.pdb D;696 1qx8.pdb B 1r4m_3.pdb F;697 1r1t.pdb B 1r8g.pdb B;698 1r4w D 1rdq.pdb I;699 1r7j.pdb B 1rgx.pdb B;700 1rec.pdb B 1rku.pdb B;701 1rgx.pdb B 1rqi.pdb B;702 1rmr.pdb B 1rye.pdb B;703 1rrm.pdb B 1s1c.pdb B;704 1rw6.pdb B 1s5u_1.pdb B;705 1s28 D 1s9r.pdb B;706 1s5u B 1sdd.pdb B;707 1sbs.pdb L 1sgh.pdb B;708 1sdx.pdb E 1skv_2.pdb D;709 1sfx.pdb B 1spp.pdb A;710 1slm.pdb B 1ss4.pdb B;711 1spp.pdb A 1svx.pdb B;712 1su1.pdb B 1t0b_2.pdb F;713 1sw6.pdb B 1t3j.pdb B;714 1sz6.pdb B 1t62.pdb B;715 1t3m.pdb B 1t98.pdb B;716 1t62.pdb B 1tco.pdb B;717 1ta3.pdb B 1thf.pdb A;718 1td2.pdb B 1tlt.pdb B;719 1tfe.pdb B 1tqy_3.pdb F;720 1tmc.pdb B 1tvd.pdb B;721 1tqy F 1txg.pdb B;722 1tvx.pdb B 1u0e.pdb B;723 1txn.pdb B 1u69.pdb B;724 1tzb.pdb B 1u8v.pdb B;725 1u6l.pdb B 1ucr.pdb B;726 1u8v.pdb B 1ufo_1.pdb B;727 1udr.pdb B 1uld_1.pdb A;728 1ufy.pdb B 1unl_2.pdb E;729 1uk8.pdb B 1usc.pdb B;730 1up7 B 1uu1_2.pdb D;731 1usc.pdb B 1uwk.pdb B;732 1uur.pdb B 1v0e_2.pdb E;733 1uwu.pdb B 1v4g.pdb B;734 1uzm.pdb B 1v74.pdb B;735 1v58 B 1v9y.pdb B;736 1v74.pdb B 1vdw.pdb B;737 1vbo A 1vhq.pdb B;738 1ve1.pdb B 1vj0.pdb B;739 1vgy.pdb B 1vkd_2.pdb D;740 1vjg.pdb B 1vls.pdb B;741 1vkd D 1vpz.pdb B;742 1vmg.pdb B 1vz8_1.pdb B;743 1vq0.pdb B 1w30.pdb B;744 1vrw.pdb B 1w6s.pdb B;745 1w3b.pdb B 1wet.pdb D;746 1w6s.pdb B 1wmh.pdb B;747 1wht.pdb B 1wty.pdb B;748 1wmi.pdb B 1wvl.pdb B;749 1wqw.pdb B 1wyu_1.pdb B;750 1ww9.pdb B 1x7y.pdb B;751 1wyu B 1xcf.pdb B;752 1x92.pdb B 1xfs.pdb B;753 1xcg B 1xk4_3.pdb J;754 1xe7 C 1xma.pdb B;755 1xka.pdb C 1xrk.pdb B;756 1xma.pdb B 1xuv_2.pdb B;757 1xs1 B 1y0g_1.pdb B;758 1xv2 B 1y4j.pdb B;759 1xx6.pdb B 1y7p_1.pdb C;760 1y5h.pdb B 1ybh.pdb B;761 1y7p C 1yer.pdb B;762 1ycg B 1ylx.pdb B;763 1yf5.pdb A 1yr0_2.pdb D;764 1ykh.pdb B 1yuz.pdb B;765 1yre D 1z24.pdb B;766 1yuz.pdb B 1z84.pdb B;767 1z41.pdb B 1zcs.pdb B;768 1z8u D 1zkd.pdb B;769 1zbr.pdb B 1zpd.pdb B;770 1zkp C 1zva.pdb B;771 1zpd.pdb B 2a2q.pdb H;772 1zww.pdb B 2a7k_2.pdb E;773 2a2u.pdb B 2ac0.pdb B;774 2a5z.pdb B 2ag5.pdb B;775 2adf.pdb H 2aq6.pdb B;776 2ag5.pdb B 2avp.pdb B;777 2ark B 2b0a.pdb B;778 2awi D 2b5d.pdb A;779 2ayu.pdb B 2ba2.pdb B;780 2b5i.pdb B 2bf9.pdb B;781 2ba2.pdb B 2bky.pdb B;782 2bgx.pdb B 2bwv.pdb B;783 2bll.pdb B 2c4j_1.pdb B;784 2bpl C 2car.pdb B;785 2c4v.pdb B 2cme_4.pdb G;786 2car.pdb B 2cvz.pdb B;787 2cnz.pdb B 2d3e.pdb B;788 2cwk.pdb B 2dj5.pdb B;789 2cz4.pdb B 2e7s_4.pdb H;790 2dpi.pdb D 2f06.pdb B;791 2e7s H 2f3d.pdb B;792 2f1k B 2f7f.pdb B;793 2f3x.pdb B 2fbi.pdb B;794 2f6k.pdb B 2ff4_1.pdb E;795 2fbl.pdb B 2flu.pdb P;796 2ff4 E 2fp4.pdb B;797 2fmt.pdb B 2fur.pdb B;798 2fpn.pdb B 2g3p.pdb B;799 2ftx.pdb B 2g82_1.pdb P;800 2g50 F 2gfa_2.pdb D;801 2g82 P 2gj7.pdb F;802 2ghp F 2gvh.pdb B;803 2gj8 D 2h6l.pdb B;804 2gtd F 2hft.pdb B;805 2h9d D 2hrr.pdb B;806 2hft.pdb B 2i4j.pdb B;807 2hwn D 2iim.pdb B;808 2i52 B 2iv9.pdb B;809 2ido D 2kin.pdb B;810 2ixp D 2ntk.pdb B;811 2kin.pdb B 2o23.pdb B;812 2nvn.pdb B 2p8i_1.pdb B;813 2o25 D 2prg.pdb B;814 2oza.pdb B 2rmc.pdb C;815 2q3m.pdb B 3bam.pdb B;816 2rmc.pdb C 3grs.pdb B;817 3cro.pdb R 3ssi.pdb B;818 3gtu D 4pro_1.pdb B;819 3pnp.pdb B 7gpb.pdb B;820 1a2z.pdb B 1a7b.pdb D;821 1a73.pdb B 1abb.pdb B;822 1adu.pdb B 1alu.pdb B;823 1ais.pdb B 1apy.pdb B;824 1aoh.pdb B 1aua.pdb B;825 1auo.pdb B 1awp.pdb B;826 1avz.pdb B 1b01.pdb B;827 1b1z.pdb B 1b5e.pdb B;828 1b49.pdb C 1b7y.pdb B;829 1b6u.pdb B 1bbp.pdb B;830 1bdb.pdb B 1bh0.pdb B;831 1bgf.pdb B 1bkf.pdb B;832 1bml.pdb B 1br1_1.pdb B;833 1bqp B 1bun.pdb B;834 1btk.pdb B 1byk.pdb B;835 1bzq N 1c4p.pdb B;836 1c3k.pdb B 1c8b.pdb B;837 1c9o.pdb B 1cei.pdb B;838 1cd9.pdb B 1ci6.pdb A;839 1chm.pdb B 1cmc.pdb B;840 1cnz.pdb B 1csg.pdb B;841 1cs1.pdb B 1cv8.pdb B;842 1cxp.pdb C 1d1m.pdb A;843 1d0q.pdb B 1d3y.pdb B;844 1d2s.pdb B 1d8l.pdb B;845 1d9c.pdb B 1dee_2.pdb D;846 1dd3 B 1dhk.pdb B;847 1dj7.pdb B 1dlj.pdb B;848 1dl5.pdb B 1dqe.pdb B;849 1dov.pdb B 1duv.pdb H;850 1dvp.pdb B 1dzf.pdb B;851 1dyw.pdb B 1e3m.pdb B;852 1e50 A 1e87.pdb B;853 1e7n.pdb B 1ebd.pdb B;854 1eak C 1edz.pdb B;855 1eeq.pdb B 1ega.pdb B;856 1eg4.pdb P 1eje.pdb B;857 1eku.pdb B 1ep5.pdb A;858 1eo6.pdb B 1ete_2.pdb D;859 1ern.pdb B 1ew6.pdb B;860 1ewy C 1ezs.pdb B;861 1ezg.pdb B 1f1x.pdb B;862 1f2u.pdb B 1f44.pdb D;863 1f3u F 1f6y.pdb B;864 1f5v.pdb B 1fa2.pdb B;865 1fbt.pdb B 1ff9.pdb B;866 1fe8 J 1flk_2.pdb B;867 1fm0.pdb E 1fp3.pdb B;868 1fp1.pdb A 1fs0.pdb G;869 1fr2.pdb B 1fui_2.pdb E;870 1fwx D 1g2y_2.pdb D;871 1g1j.pdb B 1g6u.pdb B;872 1g8k F 1gcq.pdb A;873 1gc1 C 1gjo.pdb B;874 1gg6.pdb C 1gl4.pdb B;875 1gmv.pdb B 1gpu.pdb B;876 1gpm.pdb B 1gt6.pdb B;877 1gtw.pdb B 1gw0.pdb B;878 1gvn.pdb B 1gxr.pdb B;879 1gxc B 1gz6.pdb B;880 1gzj.pdb B 1h2s.pdb B;881 1h21.pdb B 1h5q_3.pdb J;882 1h6o.pdb B 1h99.pdb B;883 1h8g.pdb B 1hcn.pdb B;884 1hbx E 1hfe_2.pdb M;885 1hh2.pdb A 1hn4.pdb B;886 1hlo.pdb B 1hsj.pdb B;887 1htr.pdb P 1hx1.pdb B;888 1hw7.pdb B 1hyu.pdb B;889 1hyl.pdb B 1i2m_2.pdb D;890 1i31.pdb P 1i6l.pdb B;891 1i52.pdb B 1iag.pdb B;892 1ibr D 1ig3.pdb B;893 1ied.pdb B 1ik6.pdb B;894 1ii7.pdb B 1iro.pdb B;895 1isy.pdb B 1ixm.pdb B;896 1ix2.pdb B 1j2j.pdb B;897 1j3b.pdb B 1j8u.pdb B;898 1j7n.pdb B 1jch.pdb B;899 1jay.pdb B 1jfi.pdb B;900 1jg8.pdb B 1jjc.pdb B;901 1ji7.pdb B 1jl4.pdb B;902 1jlv B 1jog_2.pdb D;903 1joc.pdb B 1jro_2.pdb F;904 1jqp.pdb B 1juv.pdb B;905 1jvm.pdb B 1jzo.pdb B;906 1jys.pdb B 1k4z_2.pdb B;907 1k5n.pdb B 1ka8_1.pdb B;908 1k94.pdb B 1kcx.pdb B;909 1kc7.pdb B 1khd_2.pdb C;910 1ki1 D 1kl1.pdb B;911 1kk8.pdb B 1ko7_1.pdb B;912 1kor.pdb B 1ktn.pdb B;913 1ktb.pdb B 1kxv_1.pdb C;914 1kut.pdb B 1l3c.pdb B;915 1l3p.pdb B 1l8d.pdb B;916 1l7d D 1lfd_1.pdb B;917 1lj9.pdb B 1lns.pdb B;918 1lm8.pdb C 1lrh_2.pdb C;919 1lq9.pdb B 1lwd.pdb B;920 1lxd.pdb B 1m2d.pdb B;921 1m1l.pdb B 1m4z.pdb B;922 1m6d.pdb B 1mah.pdb F;923 1m9x C 1mhp_1.pdb H;924 1mg1.pdb B 1mk2.pdb B;925 1mkf.pdb B 1mou.pdb B;926 1mo9.pdb B 1mr7_1.pdb B;927 1msc.pdb B 1mw5.pdb B;928 1mvk J 1mze.pdb B;929 1my7.pdb B 1n26.pdb B;930 1n2m E 1n7z_2.pdb D;931 1n7h.pdb B 1nbq.pdb B;932 1nco.pdb B 1nfd_1.pdb B;933 1nf3 C 1nkd.pdb B;934 1ni4.pdb B 1nlt.pdb B;935 1nme.pdb B 1npe.pdb B;936 1nox.pdb B 1nsn.pdb H;937 1nt2.pdb B 1nvv.pdb R;938 1nvm B 1ny5.pdb B;939 1nxj B 1o22.pdb B;940 1o4w.pdb B 1o63.pdb B;941 1o5h.pdb B 1o7n.pdb B;942 1o89.pdb B 1oai.pdb B;943 1oa8.pdb B 1ocv.pdb B;944 1obx.pdb C 1ofu.pdb B;945 1og6.pdb B 1ojh_5.pdb J;946 1oio.pdb B 1olq.pdb B;947 1omw.pdb B 1oq9.pdb B;948 1ope.pdb B 1ot6.pdb B;949 1oru.pdb B 1ovn.pdb B;950 1ox0.pdb B 1p1j.pdb B;951 1p0x.pdb B 1p5z.pdb A;952 1p74.pdb B 1pby.pdb B;953 1p9o.pdb B 1pf5.pdb B;954 1pdo.pdb B 1pix.pdb B;955 1pk1 B 1pn2_2.pdb D;956 1pm3.pdb B 1ptq.pdb B;957 1pv1.pdb B 1py9.pdb B;958 1pxv C 1q18.pdb B;959 1q0q.pdb B 1q4u.pdb B;960 1q67.pdb B 1qah.pdb B;961 1q98.pdb B 1qf8.pdb B;962 1qh3.pdb B 1qjh.pdb B;963 1qj8.pdb B 1qm4.pdb B;964 1qks.pdb B 1qoj.pdb B;965 1qoz.pdb B 1qsd.pdb B;966 1qre.pdb B 1qw2.pdb B;967 1qx4.pdb B 1r0o.pdb B;968 1qzq.pdb B 1r30.pdb B;969 1r1k.pdb A 1r6t.pdb B;970 1r7a.pdb B 1r9f.pdb F;971 1r8s.pdb E 1rew.pdb B;972 1rgf.pdb B 1rk4.pdb B;973 1rj9.pdb B 1rp3_1.pdb B;974 1rmd.pdb B 1rv1.pdb B;975 1rw0.pdb B 1rzn.pdb B;976 1rz1 B 1s3z.pdb B;977 1s5a.pdb B 1s7y.pdb B;978 1s7m E 1sc3.pdb B;979 1sbb.pdb B 1sf8_2.pdb H;980 1sfp.pdb B 1shy.pdb B;981 1sh5.pdb B 1smx.pdb B;982 1sox.pdb B 1sr4.pdb B;983 1sqs.pdb B 1suw.pdb B;984 1stz.pdb B 1sy7.pdb B;985 1sz2.pdb B 1t1r.pdb B;986 1t11.pdb B 1t4h.pdb B;987 1t5o D 1t82_1.pdb B;988 1t70 H 1taw.pdb B;989 1t9i.pdb B 1te5.pdb B;990 1tf0.pdb B 1tk9.pdb B;991 1tj7.pdb B 1to6.pdb B;992 1tqb.pdb B 1tui.pdb B;993 1tue L 1twi_1.pdb B;994 1tvn.pdb B 1tye_3.pdb F;995 1tz9.pdb B 1u2g.pdb B;996 1u19.pdb B 1u7i.pdb B;997 1u8s.pdb B 1ub3.pdb B;998 1uaz.pdb B 1ue1.pdb B;999 1udd.pdb B 1uiu.pdb B;1000 1ujq.pdb B 1umy.pdb B;

Random set 4

1 12as.pdb B 1a0h.pdb B;2 1a2z.pdb B 1a3d.pdb B;3 1a73.pdb B 1a8o.pdb B;4 1aa7.pdb B 1ad3.pdb B;5 1ais.pdb B 1am2.pdb B;6 1aoh.pdb B 1aqt.pdb B;7 1auo.pdb B 1ava_2.pdb D;8 1avz.pdb B 1axi.pdb B;9 1ayf.pdb B 1b0u.pdb B;10 1b49.pdb C 1b5p.pdb B;11 1b6u.pdb B 1b8m.pdb B;12 1bdb.pdb B 1bdy.pdb B;13 1bgf.pdb B 1bhg.pdb B;14 1bi7.pdb B 1blx.pdb B;15 1bqp B 1brw.pdb B;16 1btk.pdb B 1bvn.pdb T;17 1bzq N 1c0m_1.pdb B;18 1c3k.pdb B 1c4z.pdb B;19 1c76.pdb B 1c94.pdb B;20 1cd9.pdb B 1cf7.pdb B;21 1chm.pdb B 1cjt.pdb B;22 1cnz.pdb B 1cp2.pdb B;23 1cs1.pdb B 1csn.pdb B;24 1ctt.pdb B 1cvs.pdb B;25 1d0q.pdb B 1d1z.pdb B;26 1d2s.pdb B 1d4x.pdb G;27 1d9c.pdb B 1dbq.pdb B;28 1dd3 B 1dev_1.pdb B;29 1dfn.pdb B 1div.pdb B;30 1dl5.pdb B 1dmu.pdb C;31 1dov.pdb B 1dqn.pdb B;32 1dvp.pdb B 1dxx.pdb B;33 1dyw.pdb B 1e0b.pdb B;34 1e1z.pdb A 1e4e.pdb B;35 1e7n.pdb B 1e96.pdb B;36 1eak C 1ec8.pdb B;37 1eeq.pdb B 1ef1.pdb C;38 1eg4.pdb P 1egp.pdb B;39 1ei5.pdb B 1eke.pdb B;40 1eo6.pdb B 1epf_2.pdb D;41 1ern.pdb B 1euv.pdb B;42 1ewy C 1ex4.pdb B;43 1ezg.pdb B 1ezx.pdb C;44 1f0c.pdb B 1f2l.pdb B;45 1f3u F 1f46.pdb B;46 1f5v.pdb B 1f7l.pdb B;47 1fbt.pdb B 1fcc.pdb C;48 1fe8 J 1fgj_1.pdb B;49 1fiu.pdb B 1flo.pdb B;50 1fp1.pdb A 1fq1.pdb B;51 1fr2.pdb B 1fs2.pdb B;52 1fwx D 1fxw.pdb F;53 1g1j.pdb B 1g3n_1.pdb B;54 1g5g E 1g73_1.pdb C;55 1gc1 C 1gdt.pdb B;56 1gg6.pdb C 1gk1_2.pdb B;57 1gmv.pdb B 1go8.pdb A;58 1gpm.pdb B 1gqi.pdb B;59 1gr0.pdb B 1gtd.pdb B;60 1gvn.pdb B 1gwi.pdb B;61 1gxc B 1gxy.pdb B;62 1gzj.pdb B 1h0d.pdb B;63 1h21.pdb B 1h3f.pdb B;64 1h4p.pdb B 1h6d_2.pdb F;65 1h8g.pdb B 1h9m_1.pdb B;66 1hbx E 1hcx.pdb B;67 1hh2.pdb A 1hjx.pdb B;68 1hlo.pdb B 1hng.pdb B;69 1hqr.pdb B 1hss_1.pdb B;70 1hw7.pdb B 1hxm_3.pdb F;71 1hyl.pdb B 1hzh.pdb K;72 1i31.pdb P 1i3k.pdb B;73 1i52.pdb B 1i75.pdb B;74 1i85.pdb B 1iar.pdb B;75 1ied.pdb B 1igq_1.pdb C;76 1ii7.pdb B 1ilr.pdb 2;77 1isy.pdb B 1itu.pdb B;78 1ix2.pdb B 1iyb.pdb B;79 1j1b.pdb B 1j30.pdb B;80 1j7n.pdb B 1j9l.pdb B;81 1jay.pdb B 1jd0.pdb B;82 1jg8.pdb B 1jhc.pdb B;83 1ji7.pdb B 1jk4.pdb B;84 1jkj B 1jl9.pdb B;85 1joc.pdb B 1jps.pdb H;86 1jqp.pdb B 1js0.pdb B;87 1jvm.pdb B 1jwy.pdb B;88 1jys.pdb B 1k0d_1.pdb B;89 1k3e.pdb B 1k55_1.pdb C;90 1k94.pdb B 1kae.pdb B;91 1kc7.pdb B 1kdg.pdb B;92 1ki1 D 1kij.pdb B;93 1kk8.pdb B 1klg.pdb B;94 1knc.pdb B 1kob.pdb B;95 1ktb.pdb B 1ktz.pdb B;96 1kut.pdb B 1kyq_2.pdb C;97 1l3p.pdb B 1l5o.pdb B;98 1l7d D 1lb6.pdb B;99 1ldf.pdb B 1li4.pdb B;100 1lm8.pdb C 1log.pdb B;101 1lq9.pdb B 1lss_1.pdb B;102 1lxd.pdb B 1ly1.pdb B;103 1m1l.pdb B 1m2t.pdb B;104 1m3w.pdb B 1m63_1.pdb B;105 1m9x C 1mby.pdb B;106 1mg1.pdb B 1mi1.pdb B;107 1mkf.pdb B 1mkz.pdb B;108 1mo9.pdb B 1moy.pdb B;109 1mpy.pdb B 1mrp.pdb B;110 1mvk J 1mwv.pdb B;111 1my7.pdb B 1mzn_1.pdb B;112 1n2m E 1n4q_2.pdb D;113 1n7h.pdb B 1n93.pdb A;114 1naw.pdb B 1nby.pdb B;115 1nf3 C 1ngm_2.pdb F;116 1ni4.pdb B 1nkn_2.pdb D;117 1nme.pdb B 1nmu_1.pdb B;118 1nox.pdb B 1nqd.pdb B;119 1nrj.pdb B 1nsz.pdb B;120 1nvm B 1nw2_2.pdb F;121 1nxj B 1nyt.pdb B;122 1o4w.pdb B 1o51.pdb B;123 1o5h.pdb B 1o6a.pdb B;124 1o6z.pdb B 1o81.pdb B;125 1oa8.pdb B 1oaz_1.pdb H;126 1obx.pdb C 1od4.pdb B;127 1og6.pdb B 1ohw_2.pdb D;128 1oio.pdb B 1ok3.pdb B;129 1oki.pdb B 1olz.pdb B;130 1ope.pdb B 1or0_2.pdb D;131 1oru.pdb B 1otj_2.pdb C;132 1ox0.pdb B 1oya.pdb B;133 1p0x.pdb B 1p27.pdb B;134 1p35 B 1p6a.pdb B;135 1p9o.pdb B 1pc6.pdb B;136 1pdo.pdb B 1pff.pdb B;137 1pk1 B 1pkp.pdb B;138 1pm3.pdb B 1poc.pdb B;139 1pqw.pdb B 1puc.pdb B;140 1pxv C 1pym.pdb B;141 1q0q.pdb B 1q2w.pdb B;142 1q67.pdb B 1q77.pdb B;143 1q98.pdb B 1qav.pdb B;144 1qc7.pdb B 1qfx.pdb B;145 1qj8.pdb B 1qkd.pdb B;146 1qks.pdb B 1qme.pdb B;147 1qoz.pdb B 1qpa.pdb B;148 1qre.pdb B 1qsm.pdb B;149 1qup.pdb B 1qwh.pdb B;150 1qzq.pdb B 1r0v_2.pdb D;151 1r1k.pdb A 1r3s.pdb B;152 1r7a.pdb B 1r89.pdb B;153 1r8s.pdb E 1rcq.pdb B;154 1re3 B 1rfz.pdb B;155 1rj9.pdb B 1rkd.pdb B;156 1rmd.pdb B 1rq2.pdb B;157 1rw0.pdb B 1rwz.pdb B;158 1rz1 B 1rzr_1.pdb C;159 1s1q D 1s4k.pdb B;160 1s7m E 1s96.pdb B;161 1sbb.pdb B 1scf_1.pdb B;162 1sfp.pdb B 1sg1.pdb B;163 1sh5.pdb B 1sjp.pdb B;164 1skz.pdb B 1sny.pdb B;165 1sqs.pdb B 1sr9.pdb B;166 1stz.pdb B 1svd.pdb M;167 1sz2.pdb B 1szq.pdb B;168 1t11.pdb B 1t2d.pdb B;169 1t3l.pdb B 1t5b.pdb B;170 1t70 H 1t8q_2.pdb C;171 1t9i.pdb B 1tbx.pdb B;172 1tf0.pdb B 1tgs.pdb I;173 1tj7.pdb B 1tlj.pdb B;174 1tm0.pdb B 1toq_3.pdb F;175 1tue L 1tuv.pdb B;176 1tvn.pdb B 1tx3_2.pdb D;177 1tz9.pdb B 1tzp.pdb B;178 1u19.pdb B 1u5k.pdb B;179 1u6g.pdb B 1u7z_1.pdb B;180 1uaz.pdb B 1uby.pdb B;181 1udd.pdb B 1uf5.pdb B;182 1ujq.pdb B 1ukl_2.pdb E;183 1um0.pdb B 1un8.pdb B;184 1unn.pdb B 1uru.pdb B;185 1ut7.pdb B 1utg.pdb B;186 1uuj.pdb B 1uw1.pdb B;187 1uzb.pdb B 1v00_1.pdb C;188 1v1p.pdb B 1v3e.pdb B;189 1v4v.pdb B 1v70.pdb B;190 1v8c B 1v8q.pdb B;191 1vbk.pdb B 1vd6.pdb B;192 1vg0.pdb B 1vh4.pdb B;193 1vi0.pdb B 1vio.pdb B;194 1vj2.pdb B 1vk6.pdb B;195 1vl0.pdb B 1vlf_1.pdb N;196 1vme.pdb B 1vp7_1.pdb B;197 1vrs E 1vsg.pdb B;198 1w07.pdb B 1w2i.pdb B;199 1w36 F 1w63_1.pdb B;200 1wa5.pdb B 1wc9.pdb B;201 1who.pdb B 1wlh.pdb B;202 1wq1.pdb G 1ws8.pdb B;203 1wud.pdb B 1wve.pdb C;204 1ww1.pdb B 1wxx_1.pdb B;205 1x3m.pdb B 1x77.pdb B;206 1x8z C 1xb2.pdb B;207 1xdt.pdb R 1xew.pdb Y;208 1xgs.pdb B 1xi9.pdb B;209 1xk7 B 1xl4.pdb B;210 1xpj.pdb B 1xqa.pdb B;211 1xs0 B 1xu9.pdb B;212 1xwr.pdb B 1xzp.pdb B;213 1y0z.pdb B 1y2o.pdb B;214 1y4m.pdb B 1y71.pdb B;215 1yak B 1yb5.pdb B;216 1ycc.pdb B 1ydg_2.pdb F;217 1yj5 B 1ylf.pdb B;218 1ynr.pdb B 1ypt.pdb B;219 1yrb.pdb B 1yta_1.pdb B;220 1yxb F 1z0p.pdb B;221 1z3e.pdb B 1z6b_1.pdb B;222 1zbo.pdb B 1zbx.pdb B;223 1zed.pdb B 1zjc.pdb B;224 1zke E 1zno.pdb B;225 1zrs.pdb B 1ztd.pdb B;226 1zvp D 2a1j.pdb B;227 2a5t.pdb B 2a6a.pdb B;228 2a99.pdb B 2aaf.pdb B;229 2aca.pdb B 2aee.pdb B;230 2aj7.pdb B 2akz.pdb B;231 2arh.pdb B 2av9_2.pdb H;232 2ayo.pdb B 2az5.pdb B;233 2b0l B 2b4j.pdb B;234 2b5g.pdb B 2b8m.pdb B;235 2bcn.pdb B 2be3.pdb B;236 2bgw.pdb B 2bko.pdb B;237 2bp3 T 2buo.pdb T;238 2c12 B 2c31.pdb B;239 2c4n.pdb B 2c9l.pdb Z;240 2ch5 B 2clo.pdb B;241 2cn4.pdb B 2cu6.pdb B;242 2cz1.pdb B 2d29.pdb B;243 2d6y.pdb B 2dft.pdb B;244 2e2d.pdb C 2eia.pdb B;245 2eul B 2f01.pdb B;246 2f1f.pdb B 2f2l.pdb X;247 2f69.pdb B 2f6s.pdb B;248 2f96.pdb B 2fb5.pdb B;249 2fex B 2ffe.pdb B;250 2fhq.pdb B 2fiu.pdb B;251 2fml.pdb B 2fo7.pdb B;252 2ftw.pdb B 2fuj.pdb B;253 2fzt.pdb B 2g39.pdb B;254 2g7s.pdb B 2g8o.pdb B;255 2gc7 F 2gen.pdb B;256 2gh1.pdb B 2giy.pdb B;257 2gsm B 2gus.pdb B;258 2gyq.pdb B 2h2n.pdb B;259 2hew.pdb A 2hhm.pdb B;260 2hoe.pdb B 2hqx.pdb B;261 2hu7.pdb B 2i02.pdb B;262 2idj.pdb B 2igt.pdb B;263 2ijd A 2it9_1.pdb B;264 2jhf.pdb B 2mad.pdb H;265 2nlz.pdb B 2nrf.pdb B;266 2nul.pdb B 2nz8.pdb B;267 2otx.pdb B 2p3r_1.pdb B;268 2pg2.pdb B 2plh.pdb B;269 2rln.pdb E 2scp.pdb B;270 2tnf.pdb B 2vsg.pdb B;271 3cla.pdb B 3fru_2.pdb D;272 3pfk.pdb B 3sdh.pdb B;273 4ake.pdb B 4mt2.pdb B;274 12as.pdb B 1a22.pdb B;275 1a12.pdb B 1a5t.pdb B;276 1a2z.pdb B 1a9n_2.pdb D;277 1aa7.pdb B 1aie.pdb B;278 1adu.pdb B 1an7.pdb B;279 1aoh.pdb B 1au1.pdb B;280 1aro.pdb L 1avv.pdb B;281 1auo.pdb B 1ay9.pdb B;282 1ayf.pdb B 1b3q.pdb B;283 1b1z.pdb B 1b6c_3.pdb F;284 1b6u.pdb B 1bb1.pdb B;285 1b9c.pdb B 1bg1.pdb D;286 1bdb.pdb B 1bi2.pdb B;287 1bi7.pdb B 1bou.pdb B;288 1bml.pdb B 1bt6.pdb B;289 1btk.pdb B 1byf.pdb B;290 1bw0.pdb B 1c2b.pdb B;291 1bzq N 1c6o.pdb B;292 1c76.pdb B 1cbm.pdb B;293 1c9o.pdb B 1cg2.pdb B;294 1chm.pdb B 1clv.pdb I;295 1cku.pdb B 1cqk.pdb B;296 1cnz.pdb B 1ctn.pdb B;297 1ctt.pdb B 1czj.pdb B;298 1cxp.pdb C 1d2g.pdb B;299 1d2s.pdb B 1d7y.pdb B;300 1d6r.pdb I 1dce_2.pdb D;301 1d9c.pdb B 1dfm.pdb B;302 1dfn.pdb B 1dkf.pdb B;303 1dj7.pdb B 1doh.pdb B;304 1dov.pdb B 1dto.pdb B;305 1dqz.pdb B 1dy9_1.pdb C;306 1dvp.pdb B 1e1o.pdb B;307 1e1z.pdb A 1e6b.pdb B;308 1e50 A 1eag.pdb B;309 1eak C 1edh.pdb B;310 1ecm.pdb B 1efn.pdb B;311 1eeq.pdb B 1ehi.pdb B;312 1ei5.pdb B 1em8_2.pdb D;313 1eku.pdb B 1eqr.pdb B;314 1ern.pdb B 1evy.pdb B;315 1ev7.pdb B 1eys.pdb L;316 1ewy C 1f08.pdb B;317 1f0c.pdb B 1f3d.pdb H;318 1f2u.pdb B 1f5m.pdb B;319 1f5v.pdb B 1f9j.pdb B;320 1f8e.pdb B 1fd3_1.pdb C;321 1fbt.pdb B 1fic.pdb B;322 1fiu.pdb B 1fnu.pdb B;323 1fm0.pdb E 1fqt.pdb B;324 1fr2.pdb B 1ftr.pdb B;325 1fsg.pdb C 1fzd_2.pdb F;326 1fwx D 1g5c_2.pdb D;327 1g5g E 1g8t.pdb B;328 1g8k F 1gfl.pdb B;329 1gg6.pdb C 1gl1_1.pdb I;330 1gka.pdb B 1gp9.pdb B;331 1gmv.pdb B 1gqy.pdb B;332 1gr0.pdb B 1gux.pdb B;333 1gtw.pdb B 1gx4.pdb B;334 1gxc B 1gz0_4.pdb E;335 1gyg B 1h1v.pdb G;336 1gzj.pdb B 1h4g.pdb B;337 1h4p.pdb B 1h7e.pdb B;338 1h6o.pdb B 1ha0.pdb B;339 1hbx E 1hei.pdb B;340 1he1 C 1hl6.pdb B;341 1hh2.pdb A 1hpc.pdb B;342 1hqr.pdb B 1hv8.pdb B;343 1htr.pdb P 1hxy.pdb B;344 1hyl.pdb B 1i24.pdb B;345 1i0r.pdb B 1i4j.pdb B;346 1i31.pdb P 1i7w.pdb B;347 1i85.pdb B 1icr.pdb B;348 1ibr D 1ihk.pdb B;349 1ii7.pdb B 1iqd.pdb B;350 1iom.pdb B 1iv3_1.pdb B;351 1isy.pdb B 1j0h.pdb B;352 1j1b.pdb B 1j5p.pdb B;353 1j3b.pdb B 1jaf.pdb B;354 1jay.pdb B 1jer.pdb B;355 1jdp.pdb B 1jhg.pdb B;356 1jg8.pdb B 1jkg.pdb B;357 1jkj B 1jmj.pdb B;358 1jlv B 1jql.pdb B;359 1jqp.pdb B 1jtv.pdb B;360 1js3.pdb B 1jya.pdb B;361 1jvm.pdb B 1k2f.pdb B;362 1k3e.pdb B 1k7w.pdb B;363 1k5n.pdb B 1kb5.pdb L;364 1kc7.pdb B 1kgn_2.pdb D;365 1kfi.pdb B 1kj1_2.pdb Q;366 1ki1 D 1kmi.pdb Z;367 1knc.pdb B 1krl_2.pdb C;368 1kor.pdb B 1ku7.pdb D;369 1kut.pdb B 1l2w_2.pdb D;370 1l0a.pdb B 1l6l_7.pdb N;371 1l3p.pdb B 1ldd.pdb B;372 1ldf.pdb B 1llf.pdb B;373 1lj9.pdb B 1lpb.pdb B;374 1lq9.pdb B 1lvm_1.pdb E;375 1ltx.pdb B 1m0l.pdb B;376 1lxd.pdb B 1m3k.pdb B;377 1m3w.pdb B 1m7r.pdb B;378 1m6d.pdb B 1me8.pdb B;379 1mg1.pdb B 1mjo.pdb B;380 1miw.pdb B 1mmf_2.pdb E;381 1mkf.pdb B 1mpx.pdb B;382 1mpy.pdb B 1mus.pdb F;383 1msc.pdb B 1mxi.pdb B;384 1my7.pdb B 1n1j.pdb B;385 1n12 D 1n69_1.pdb B;386 1n2m E 1na8.pdb B;387 1naw.pdb B 1nek.pdb B;388 1nco.pdb B 1nhk.pdb L;389 1ni4.pdb B 1nlr.pdb B;390 1nkt.pdb B 1no4_1.pdb B;391 1nme.pdb B 1nri.pdb B;392 1nrj.pdb B 1nuq.pdb B;393 1nt2.pdb B 1nww.pdb B;394 1nxj B 1o1h.pdb B;395 1o0s.pdb B 1o58.pdb B;396 1o4w.pdb B 1o6u.pdb C;397 1o6z.pdb B 1o97.pdb D;398 1o89.pdb B 1obf.pdb P;399 1obx.pdb C 1of8.pdb B;400 1oe9.pdb B 1oi4.pdb B;401 1og6.pdb B 1okh.pdb B;402 1oki.pdb B 1ooe.pdb B;403 1omw.pdb B 1orr.pdb B;404 1oru.pdb B 1ova_2.pdb D;405 1ou0.pdb B 1oyv.pdb B;406 1ox0.pdb B 1p32.pdb B;407 1p35 B 1p8j_2.pdb F;408 1p74.pdb B 1pdg_2.pdb C;409 1pdo.pdb B 1pin.pdb C;410 1pgr F 1pl4.pdb B;411 1pk1 B 1pqh.pdb B;412 1pqw.pdb B 1pvm.pdb B;413 1pv1.pdb B 1q08.pdb B;414 1q0q.pdb B 1q4s.pdb B;415 1q40 D 1q8b.pdb B;416 1q67.pdb B 1qbk.pdb C;417 1qc7.pdb B 1qhx.pdb B;418 1qh3.pdb B 1qkq.pdb B;419 1qks.pdb B 1qo7.pdb B;420 1qmu.pdb B 1qqf.pdb B;421 1qoz.pdb B 1qu9.pdb B;422 1qup.pdb B 1qz1.pdb B;423 1qx4.pdb B 1r17_2.pdb D;424 1r1k.pdb A 1r5p.pdb B;425 1r4m F 1r8g.pdb B;426 1r7a.pdb B 1rdq.pdb I;427 1re3 B 1rhg.pdb B;428 1rgf.pdb B 1rku.pdb B;429 1rmd.pdb B 1rtw.pdb B;430 1rqi.pdb B 1rye.pdb B;431 1rw0.pdb B 1s1c.pdb B;432 1s1q D 1s78_1.pdb C;433 1s5a.pdb B 1s9r.pdb B;434 1sbb.pdb B 1sei.pdb B;435 1sdd.pdb B 1sgh.pdb B;436 1sfp.pdb B 1skv_2.pdb D;437 1skz.pdb B 1sqd.pdb B;438 1sox.pdb B 1ss4.pdb B;439 1stz.pdb B 1sxh.pdb D;440 1svx.pdb B 1t0b_2.pdb F;441 1sz2.pdb B 1t3j.pdb B;442 1t3l.pdb B 1t6l.pdb B;443 1t5o D 1t98.pdb B;444 1t9i.pdb B 1te1.pdb B;445 1tco.pdb B 1thf.pdb A;446 1tf0.pdb B 1tlt.pdb B;447 1tm0.pdb B 1tt5_1.pdb B;448 1tqb.pdb B 1tvd.pdb B;449 1tvn.pdb B 1txy.pdb B;450 1txg.pdb B 1u0e.pdb B;451 1tz9.pdb B 1u69.pdb B;452 1u6g.pdb B 1uaa.pdb B;453 1u8s.pdb B 1ucr.pdb B;454 1udd.pdb B 1uhv.pdb B;455 1ufo B 1uld_1.pdb A;456 1ujq.pdb B 1unl_2.pdb E;457 1unn.pdb B 1uso.pdb B;458 1us7.pdb B 1uu1_2.pdb D;459 1uuj.pdb B 1uxa.pdb B;460 1uwk.pdb B 1v0e_2.pdb E;461 1uzb.pdb B 1v4g.pdb B;462 1v4v.pdb B 1v7o.pdb B;463 1v72.pdb B 1v9y.pdb B;464 1vbk.pdb B 1ve9.pdb B;465 1vdw.pdb B 1vhq.pdb B;466 1vg0.pdb B 1vj0.pdb B;467 1vj2.pdb B 1vkm_2.pdb E;468 1vka.pdb B 1vls.pdb B;469 1vme.pdb B 1vqv.pdb B;470 1vpz.pdb B 1vz8_1.pdb B;471 1vrs E 1w30.pdb B;472 1w36 F 1w9c.pdb B;473 1w6g.pdb B 1wet.pdb D;474 1who.pdb B 1wn6.pdb B;475 1wmh.pdb B 1wty.pdb B;476 1wq1.pdb G 1wvl.pdb B;477 1ww1.pdb B 1wzd.pdb B;478 1wyi.pdb B 1x7y.pdb B;479 1x8z C 1xd7.pdb B;480 1xcf.pdb B 1xfs.pdb B;481 1xdt.pdb R 1xk4_3.pdb J;482 1xk7 B 1xo0.pdb B;483 1xm3.pdb B 1xrk.pdb B;484 1xs0 B 1xvh.pdb B;485 1xuv B 1y0g_1.pdb B;486 1xwr.pdb B 1y4j.pdb B;487 1y4m.pdb B 1y97.pdb B;488 1y7m.pdb B 1ybh.pdb B;489 1ycc.pdb B 1yfz.pdb B;490 1yer.pdb B 1ylx.pdb B;491 1yj5 B 1yr0_2.pdb D;492 1yrb.pdb B 1yvk.pdb B;493 1yu0.pdb B 1z24.pdb B;494 1z3e.pdb B 1z9h_1.pdb B;495 1z84.pdb B 1zcs.pdb B;496 1zbo.pdb B 1zkd.pdb B;497 1zke E 1zq7.pdb B;498 1zp6.pdb B 1zva.pdb B;499 1zvp D 2a3q.pdb B;500 2a2q.pdb H 2a7k_2.pdb E;501 2a5t.pdb B 2ac0.pdb B;502 2aca.pdb B 2ai4.pdb B;503 2af4.pdb D 2aq6.pdb B;504 2arh.pdb B 2axo.pdb B;505 2avp.pdb B 2b0a.pdb B;506 2ayo.pdb B 2b5d.pdb A;507 2b5g.pdb B 2bb3_1.pdb B;508 2b9c.pdb B 2bf9.pdb B;509 2bgw.pdb B 2bnm.pdb B;510 2bky.pdb B 2bwv.pdb B;511 2bp3 T 2c4j_1.pdb B;512 2c4n.pdb B 2cc0.pdb B;513 2ca1.pdb B 2cme_4.pdb G;514 2cn4.pdb B 2cxk_2.pdb D;515 2cvz.pdb B 2d3e.pdb B;516 2cz1.pdb B 2dj5.pdb B;517 2dp9.pdb B 2esn.pdb C;518 2e2d.pdb C 2f06.pdb B;519 2f1f.pdb B 2f5g.pdb B;520 2f3d.pdb B 2f7f.pdb B;521 2f69.pdb B 2fbi.pdb B;522 2fbk.pdb B 2ffs.pdb B;523 2fex B 2flu.pdb P;524 2fml.pdb B 2fr5.pdb B;525 2fp4.pdb B 2fur.pdb B;526 2ftw.pdb B 2g3p.pdb B;527 2g40.pdb B 2gan.pdb B;528 2g7s.pdb B 2gfa_2.pdb D;529 2gh1.pdb B 2glz.pdb B;530 2gj7.pdb F 2gvh.pdb B;531 2gsm B 2h6l.pdb B;532 2h7z.pdb B 2hjs.pdb B;533 2hew.pdb A 2hrr.pdb B;534 2hu7.pdb B 2iad.pdb B;535 2i4j.pdb B 2iim.pdb B;536 2idj.pdb B 2iv9.pdb B;537 2ixc.pdb B 2msb.pdb B;538 2jhf.pdb B 2ntk.pdb B;539 2nul.pdb B 2ocy.pdb B;540 2o23.pdb B 2p8i_1.pdb B;541 2otx.pdb B 2prg.pdb B;542 2pva.pdb B 2shk.pdb B;543 2rln.pdb E 3bam.pdb B;544 3cla.pdb B 3ink.pdb D;545 3grs.pdb B 3ssi.pdb B;546 3pfk.pdb B 4pro_1.pdb B;547 137l.pdb B 1a64.pdb B;548 1a17.pdb B 1aa7.pdb B;549 1a78.pdb B 1aih_1.pdb B;550 1aap.pdb B 1aoc.pdb B;551 1af6.pdb B 1as4.pdb B;552 1aox.pdb B 1avw.pdb B;553 1as4.pdb B 1ayf.pdb B;554 1awc.pdb B 1b3t.pdb B;555 1ayo.pdb B 1b6r.pdb B;556 1b25.pdb B 1b9e.pdb B;557 1b74.pdb B 1bg6.pdb B;558 1b9e.pdb B 1bi7.pdb B;559 1bgp.pdb B 1bow.pdb B;560 1bih.pdb B 1btg.pdb B;561 1bmo.pdb B 1bwn.pdb B;562 1btn.pdb B 1c3c.pdb B;563 1bwn.pdb B 1c76.pdb B;564 1c3r.pdb B 1cbq.pdb B;565 1c7c.pdb B 1cg6.pdb B;566 1ca0 C 1cl7.pdb H;567 1cho.pdb I 1cru.pdb B;568 1cl7.pdb H 1ctt.pdb B;569 1cs6.pdb B 1d02.pdb B;570 1cu1.pdb B 1d2o.pdb B;571 1cxq.pdb B 1d7b.pdb B;572 1d2z D 1dcf.pdb B;573 1d7b.pdb B 1dfn.pdb B;574 1ddt.pdb B 1dkg.pdb B;575 1dg1.pdb H 1dos.pdb B;576 1dj8 F 1dru.pdb B;577 1dpg.pdb B 1dyo.pdb B;578 1dru.pdb B 1e1z.pdb A;579 1dza.pdb B 1e6j.pdb L;580 1e20.pdb B 1eaj.pdb B;581 1e58.pdb B 1ecs.pdb B;582 1ear.pdb B 1efu.pdb B;583 1ecs.pdb B 1ei5.pdb B;584 1eg5.pdb B 1ems.pdb B;585 1ei6 D 1eqt.pdb B;586 1el6.pdb B 1evj_1.pdb C;587 1erv.pdb B 1eyv.pdb B;588 1evj C 1f0c.pdb B;589 1ezi.pdb B 1f3g.pdb B;590 1f0k.pdb B 1f5q_2.pdb D;591 1f2v.pdb B 1f8f.pdb B;592 1f60.pdb B 1fe0.pdb B;593 1f8f.pdb B 1fiu.pdb B;594 1few.pdb B 1fo1.pdb B;595 1fjj.pdb B 1fqv_3.pdb F;596 1fmc.pdb B 1fsk_4.pdb K;597 1fr8.pdb B 1g0s.pdb B;598 1fsk K 1g5g_2.pdb E;599 1g29.pdb 2 1g8x.pdb B;600 1g5h D 1gg1.pdb B;601 1g8l.pdb B 1gkj.pdb B;602 1ggx.pdb B 1gpe.pdb B;603 1gkj.pdb B 1gr0.pdb B;604 1gpq.pdb B 1gve.pdb B;605 1gr7.pdb B 1gx5.pdb B;606 1gu2.pdb B 1gyo.pdb B;607 1gxd C 1h1w.pdb B;608 1gyo.pdb B 1h4p.pdb B;609 1h2b.pdb B 1h7z.pdb B;610 1h4r.pdb B 1hav.pdb B;611 1h6p.pdb B 1he7.pdb B;612 1hc7 B 1hle.pdb B;613 1he7.pdb B 1hqr.pdb B;614 1hlq.pdb B 1hvv.pdb B;615 1hqs.pdb B 1hyh.pdb B;616 1hul.pdb B 1i12_1.pdb C;617 1hyn S 1i4m.pdb B;618 1i12 C 1i85.pdb B;619 1i58.pdb B 1id1.pdb B;620 1i86.pdb B 1ii5.pdb B;621 1iby B 1ips.pdb B;622 1iil E 1ivy.pdb B;623 1ips.pdb B 1j1b.pdb B;624 1ix9.pdb B 1j5s.pdb B;625 1j1d E 1jat.pdb B;626 1j3k.pdb B 1jdw.pdb B;627 1jb7.pdb B 1jhl.pdb A;628 1jdw.pdb B 1jkj_2.pdb B;629 1jil.pdb B 1jmk.pdb O;630 1jkm.pdb B 1jqn.pdb B;631 1jlx.pdb B 1jsd.pdb B;632 1jr7.pdb B 1jye.pdb B;633 1jsd.pdb B 1k3e.pdb B;634 1jz7.pdb B 1k8r.pdb B;635 1k3s.pdb B 1kbj.pdb B;636 1k66.pdb B 1kfu.pdb S;637 1kcf.pdb B 1kjn.pdb B;638 1kfu.pdb S 1knc.pdb B;639 1kke.pdb B 1ksh.pdb B;640 1knq.pdb B 1ku9.pdb B;641 1kpf.pdb B 1l0o.pdb B;642 1kvd D 1l6r.pdb B;643 1l0o.pdb B 1ldf.pdb B;644 1l7l.pdb B 1llm.pdb D;645 1ldj.pdb B 1lq1_1.pdb D;646 1lk3 M 1lua.pdb B;647 1lql D 1m0w.pdb B;648 1lua.pdb B 1m3w.pdb B;649 1m1n B 1m7y.pdb B;650 1m3y B 1mf8.pdb B;651 1m6e.pdb A 1mjf.pdb B;652 1mh9.pdb B 1mml.pdb B;653 1mjf.pdb B 1mpy.pdb B;654 1moe.pdb B 1mv5.pdb B;655 1mq8 B 1mxr.pdb B;656 1msp.pdb B 1n1b.pdb B;657 1myl B 1n71_1.pdb B;658 1n1b.pdb B 1naw.pdb B;659 1n7s.pdb B 1neu.pdb B;660 1nb5 J 1nhw.pdb B;661 1nd4.pdb B 1nkv.pdb B;662 1ni5.pdb B 1nog.pdb B;663 1nkv.pdb B 1nrj.pdb B;664 1noy.pdb B 1nv7.pdb B;665 1nrv.pdb B 1nxh.pdb B;666 1nt3.pdb B 1o0v.pdb B;667 1nxm.pdb B 1o5d.pdb H;668 1o0v.pdb B 1o6z.pdb B;669 1o5k.pdb B 1o9l.pdb B;670 1o75.pdb B 1obo.pdb B;671 1o8b.pdb B 1oey_4.pdb M;672 1oc0.pdb B 1oi6.pdb B;673 1oey M 1oki.pdb B;674 1oj4.pdb B 1ooz.pdb B;675 1okj D 1ors.pdb B;676 1omz.pdb B 1ou8_2.pdb B;677 1orv.pdb B 1ozf.pdb B;678 1ou8 B 1p35_2.pdb B;679 1p16 C 1p9e.pdb B;680 1p5b.pdb B 1pdk.pdb B;681 1p7h M 1pgu.pdb B;682 1pe1.pdb B 1pl5.pdb S;683 1pgu.pdb B 1pqw.pdb B;684 1pm4.pdb B 1pwb.pdb B;685 1pqz.pdb B 1q0a.pdb B;686 1pv9.pdb B 1q43_1.pdb B;687 1q14.pdb B 1q8f.pdb B;688 1q43 B 1qc7.pdb B;689 1q9u.pdb B 1qi9.pdb B;690 1qd1.pdb B 1qkr.pdb B;691 1qh4 B 1qnn.pdb B;692 1qkz.pdb H 1qqg.pdb B;693 1qnn.pdb B 1qup.pdb B;694 1qrq.pdb B 1qz8.pdb B;695 1quq.pdb B 1r1d.pdb B;696 1qx8.pdb B 1r4w_2.pdb D;697 1r1t.pdb B 1r8j.pdb B;698 1r4w D 1re3_1.pdb B;699 1r9c.pdb B 1ris.pdb B;700 1rec.pdb B 1rlz.pdb B;701 1rgx.pdb B 1rrm.pdb B;702 1rmr.pdb B 1ryi_2.pdb B;703 1rrm.pdb B 1s1q_2.pdb D;704 1rzh.pdb M 1s7h_2.pdb D;705 1s28 D 1sb7.pdb B;706 1s5u B 1sdx.pdb E;707 1sbs.pdb L 1sgm.pdb B;708 1sdx.pdb E 1skz.pdb B;709 1sh8.pdb B 1sqe.pdb B;710 1slm.pdb B 1stf.pdb I;711 1spp.pdb A 1sw6.pdb B;712 1su1.pdb B 1t0h.pdb B;713 1sw6.pdb B 1t3l.pdb B;714 1t15.pdb B 1t6n.pdb B;715 1t3m.pdb B 1t9b.pdb B;716 1t62.pdb B 1td2.pdb B;717 1ta3.pdb B 1tht.pdb B;718 1td2.pdb B 1tm0.pdb B;719 1tjc.pdb B 1ttw.pdb B;720 1tmc.pdb B 1tvl.pdb B;721 1tqy F 1txn.pdb B;722 1tvx.pdb B 1u0m.pdb B;723 1txn.pdb B 1u6g.pdb B;724 1u1i.pdb B 1uad_2.pdb D;725 1u6l.pdb B 1ud0_1.pdb B;726 1u8v.pdb B 1ufy.pdb B;727 1udr.pdb B 1ulk.pdb B;728 1ufy.pdb B 1unn.pdb B;729 1ump C 1usq_2.pdb B;730 1up7 B 1uuh.pdb B;731 1usc.pdb B 1uwu.pdb B;732 1uur.pdb B 1v13.pdb B;733 1uwu.pdb B 1v4v.pdb B;734 1v25.pdb B 1v7p.pdb B;735 1v58 B 1v9z.pdb B;736 1v74.pdb B 1ve1.pdb B;737 1vbo A 1vhx.pdb B;738 1ve1.pdb B 1vj2.pdb B;739 1vi2.pdb B 1vkn.pdb B;740 1vjg.pdb B 1vlu_2.pdb B;741 1vkd D 1vq0.pdb B;742 1vmg.pdb B 1vzi.pdb B;743 1vq0.pdb B 1w36_2.pdb F;744 1w0m B 1w9e.pdb B;745 1w3b.pdb B 1whi.pdb B;746 1w6s.pdb B 1wmi.pdb B;747 1wht.pdb B 1wu7.pdb B;748 1wmi.pdb B 1ww1.pdb B;749 1wui.pdb L 1x1z.pdb B;750 1ww9.pdb B 1x82.pdb B;751 1wyu B 1xcg_1.pdb B;752 1x92.pdb B 1xg5.pdb B;753 1xcg B 1xk7_1.pdb B;754 1xhk.pdb B 1xoc.pdb B;755 1xka.pdb C 1xrs.pdb B;756 1xma.pdb B 1xv2_1.pdb B;757 1xs1 B 1y0h.pdb B;758 1xv2 B 1y4m.pdb B;759 1y14.pdb B 1y9b.pdb B;760 1y5h.pdb B 1yc5.pdb B;761 1y7p C 1yf5.pdb A;762 1ycg B 1ynb.pdb B;763 1yf5.pdb A 1yrb.pdb B;764 1yoa.pdb B 1yw6.pdb B;765 1yre D 1z2l.pdb B;766 1yuz.pdb B 1z8u_2.pdb D;767 1z41.pdb B 1zd3.pdb B;768 1z8u D 1zke_3.pdb E;769 1zee.pdb B 1zq9.pdb B;770 1zkp C 1zvb.pdb B;771 1zpd.pdb B 2a2u.pdb B;772 1zww.pdb B 2a7w_2.pdb F;773 2a2u.pdb B 2aca.pdb B;774 2a9k.pdb B 2aib.pdb B;775 2adf.pdb H 2arc.pdb B;776 2ag5.pdb B 2awi_2.pdb D;777 2ark B 2b0c.pdb B;778 2awi D 2b5g.pdb B;779 2b1y.pdb B 2bbk.pdb J;780 2b5i.pdb B 2bgc_3.pdb G;781 2ba2.pdb B 2bll.pdb B;782 2bgx.pdb B 2bz1.pdb B;783 2bll.pdb B 2c4n.pdb B;784 2c1n C 2cch_1.pdb B;785 2c4v.pdb B 2cn1.pdb B;786 2car.pdb B 2cwk.pdb B;787 2cnz.pdb B 2d45.pdb B;788 2cwk.pdb B 2dp9.pdb B;789 2d7c D 2esr.pdb B;790 2dpi.pdb D 2f0c.pdb B;791 2e7s H 2f3x.pdb B;792 2f1k B 2f8a.pdb B;793 2f3x.pdb B 2fbk.pdb B;794 2f9h.pdb B 2fge_2.pdb E;795 2fbl.pdb B 2fm8.pdb B;796 2ff4 E 2fpn.pdb B;797 2fmt.pdb B 2fyi_3.pdb D;798 2fpn.pdb B 2g40.pdb B;799 2g0t.pdb B 2gau.pdb B;800 2g50 F 2gfq.pdb B;801 2g82 P 2gj8_2.pdb D;802 2ghp F 2gwg.pdb B;803 2gj8 D 2h7z.pdb B;804 2gys.pdb B 2hlj.pdb B;805 2h9d D 2hth.pdb B;806 2hft.pdb B 2i52_2.pdb B;807 2hwn D 2iiz.pdb B;808 2i52 B 2ixc.pdb B;809 2ilk.pdb B 2mys.pdb B;810 2ixp D 2nuj.pdb B;811 2kin.pdb B 2o25_1.pdb D;812 2nvn.pdb B 2paq.pdb B;813 2o25 D 2pva.pdb B;814 2pgd.pdb B 2spc.pdb B;815 2q3m.pdb B 3cd4.pdb B;816 2rmc.pdb C 3gtu_2.pdb D;817 3cro.pdb R 3vub.pdb B;818 3gtu D 4sgb.pdb I;819 1a12.pdb B 1a3c.pdb B;820 1a2z.pdb B 1a8l.pdb B;821 1a73.pdb B 1acb.pdb I;822 1adu.pdb B 1aly.pdb B;823 1ais.pdb B 1aq0.pdb B;824 1aro.pdb L 1av1.pdb B;825 1auo.pdb B 1ax8.pdb B;826 1avz.pdb B 1b0n.pdb B;827 1b1z.pdb B 1b5f.pdb B;828 1b49.pdb C 1b8a.pdb B;829 1b9c.pdb B 1bdo.pdb B;830 1bdb.pdb B 1bh9.pdb B;831 1bgf.pdb B 1ble.pdb B;832 1bml.pdb B 1brt.pdb B;833 1bqp B 1bv1.pdb B;834 1bw0.pdb B 1c09.pdb B;835 1bzq N 1c4t.pdb B;836 1c3k.pdb B 1c8u.pdb B;837 1c9o.pdb B 1cf2.pdb R;838 1cd9.pdb B 1ci8.pdb B;839 1cku.pdb B 1cos.pdb B;840 1cnz.pdb B 1csh.pdb B;841 1cs1.pdb B 1cvi.pdb B;842 1cxp.pdb C 1d1s.pdb B;843 1d0q.pdb B 1d4t.pdb B;844 1d6r.pdb I 1db3.pdb B;845 1d9c.pdb B 1dek.pdb B;846 1dd3 B 1dir.pdb B;847 1dj7.pdb B 1dmh.pdb B;848 1dl5.pdb B 1dqi.pdb B;849 1dqz.pdb B 1dxs.pdb B;850 1dvp.pdb B 1dzi.pdb B;851 1dyw.pdb B 1e44.pdb B;852 1e50 A 1e8u.pdb B;853 1e7n.pdb B 1ebf.pdb B;854 1ecm.pdb B 1ef0.pdb B;855 1eeq.pdb B 1egj.pdb H;856 1eg4.pdb P 1ek9.pdb B;857 1eku.pdb B 1epa.pdb B;858 1eo6.pdb B 1etx.pdb B;859 1ev7.pdb B 1ex2.pdb B;860 1ewy C 1ezw.pdb B;861 1ezg.pdb B 1f2d_1.pdb B;862 1f2u.pdb B 1f45.pdb B;863 1f3u F 1f74.pdb C;864 1f8e.pdb B 1fc2.pdb D;865 1fbt.pdb B 1fg7.pdb B;866 1fe8 J 1flm.pdb B;867 1fm0.pdb E 1fp5.pdb B;868 1fp1.pdb A 1fs1.pdb B;869 1fsg.pdb C 1fxo_2.pdb F;870 1fwx D 1g2z.pdb B;871 1g1j.pdb B 1g6v.pdb K;872 1g8k F 1gct.pdb C;873 1gc1 C 1gjw.pdb B;874 1gka.pdb B 1go3_1.pdb F;875 1gmv.pdb B 1gqg.pdb B;876 1gpm.pdb B 1gt9.pdb 2;877 1gtw.pdb B 1gwb.pdb B;878 1gvn.pdb B 1gxs.pdb B;879 1gyg B 1h03.pdb Q;880 1gzj.pdb B 1h32.pdb B;881 1h21.pdb B 1h65_2.pdb C;882 1h6o.pdb B 1h9h.pdb I;883 1h8g.pdb B 1hcq_2.pdb F;884 1he1 C 1hjr_2.pdb D;885 1hh2.pdb A 1hne.pdb A;886 1hlo.pdb B 1hsl.pdb B;887 1htr.pdb P 1hx6.pdb B;888 1hw7.pdb B 1hz4.pdb B;889 1i0r.pdb B 1i3d.pdb B;890 1i31.pdb P 1i6p.pdb B;891 1i52.pdb B 1iam.pdb B;892 1ibr D 1igf.pdb H;893 1ied.pdb B 1ikn.pdb C;894 1iom.pdb B 1ith.pdb B;895 1isy.pdb B 1ixs.pdb B;896 1ix2.pdb B 1j2r.pdb B;897 1j3b.pdb B 1j98.pdb B;898 1j7n.pdb B 1jck.pdb B;899 1jdp.pdb B 1jgt.pdb B;900 1jg8.pdb B 1jk0.pdb B;901 1ji7.pdb B 1jl5.pdb B;902 1jlv B 1jpm.pdb B;903 1joc.pdb B 1jrr.pdb P;904 1js3.pdb B 1jwh.pdb B;905 1jvm.pdb B 1k04.pdb B;906 1jys.pdb B 1k51.pdb B;907 1k5n.pdb B 1ka9.pdb F;908 1k94.pdb B 1kd8_2.pdb D;909 1kfi.pdb B 1kig.pdb I;910 1ki1 D 1klf_3.pdb F;911 1kk8.pdb B 1koa.pdb B;912 1kor.pdb B 1ktp.pdb B;913 1ktb.pdb B 1kyf.pdb P;914 1l0a.pdb B 1l4i.pdb B;915 1l3p.pdb B 1l9b.pdb M;916 1l7d D 1lh0.pdb B;917 1lj9.pdb B 1lo7.pdb B;918 1lm8.pdb C 1lsh.pdb B;919 1ltx.pdb B 1lxj.pdb B;920 1lxd.pdb B 1m2o.pdb B;921 1m1l.pdb B 1m56_1.pdb B;922 1m6d.pdb B 1mbx_2.pdb D;923 1m9x C 1mhw_1.pdb C;924 1miw.pdb B 1mkm.pdb B;925 1mkf.pdb B 1mox.pdb B;926 1mo9.pdb B 1mr8.pdb B;927 1msc.pdb B 1mwq.pdb B;928 1mvk J 1mzg.pdb B;929 1n12 D 1n3l.pdb B;930 1n2m E 1n8z.pdb B;931 1n7h.pdb B 1nbw.pdb B;932 1nco.pdb B 1nfj.pdb B;933 1nf3 C 1nki.pdb B;934 1nkt.pdb B 1nmt.pdb B;935 1nme.pdb B 1nq7.pdb B;936 1nox.pdb B 1nsw.pdb B;937 1nt2.pdb B 1nw1.pdb B;938 1nvm B 1nyr.pdb B;939 1o0s.pdb B 1o50.pdb B;940 1o4w.pdb B 1o69.pdb B;941 1o5h.pdb B 1o7z.pdb B;942 1o89.pdb B 1oao.pdb B;943 1oa8.pdb B 1ocy.pdb B;944 1oe9.pdb B 1oh0.pdb B;945 1og6.pdb B 1ojr.pdb B;946 1oio.pdb B 1ols.pdb B;947 1omw.pdb B 1oqz.pdb B;948 1ope.pdb B 1otg.pdb B;949 1ou0.pdb B 1oy9.pdb B;950 1ox0.pdb B 1p22.pdb B;951 1p0x.pdb B 1p65.pdb B;952 1p74.pdb B 1pc3_2.pdb B;953 1p9o.pdb B 1pfb.pdb B;954 1pgr F 1pkh.pdb B;955 1pk1 B 1pnv.pdb B;956 1pm3.pdb B 1ptu.pdb B;957 1pv1.pdb B 1pyg.pdb B;958 1pxv C 1q2h_1.pdb B;959 1q40 D 1q6w_6.pdb L;960 1q67.pdb B 1qak.pdb B;961 1q98.pdb B 1qfh.pdb B;962 1qh3.pdb B 1qjs.pdb B;963 1qj8.pdb B 1qma.pdb B;964 1qmu.pdb B 1qp8.pdb B;965 1qoz.pdb B 1qsg_2.pdb F;966 1qre.pdb B 1qwg.pdb B;967 1qx4.pdb B 1r0r.pdb I;968 1qzq.pdb B 1r31.pdb B;969 1r4m F 1r7l.pdb B;970 1r7a.pdb B 1rcb.pdb B;971 1r8s.pdb E 1rfy_1.pdb B;972 1rgf.pdb B 1rk8.pdb B;973 1rj9.pdb B 1rq0.pdb B;974 1rqi.pdb B 1rwt_2.pdb F;975 1rw0.pdb B 1rzo.pdb B;976 1rz1 B 1s4c_2.pdb C;977 1s5a.pdb B 1s7z.pdb B;978 1s7m E 1sc6.pdb B;979 1sdd.pdb B 1sg0.pdb B;980 1sfp.pdb B 1sj1.pdb B;981 1sh5.pdb B 1snd.pdb B;982 1sox.pdb B 1sr7.pdb B;983 1sqs.pdb B 1sv0_2.pdb D;984 1svx.pdb B 1szo_1.pdb B;985 1sz2.pdb B 1t1v.pdb B;986 1t11.pdb B 1t56.pdb B;987 1t5o D 1t83.pdb B;988 1t70 H 1tbu.pdb B;989 1tco.pdb B 1tgg.pdb B;990 1tf0.pdb B 1tlf.pdb B;991 1tj7.pdb B 1to9.pdb B;992 1tqb.pdb B 1tul.pdb B;993 1tue L 1twu.pdb B;994 1txg.pdb B 1tzj.pdb B;995 1tz9.pdb B 1u5h.pdb B;996 1u19.pdb B 1u7n.pdb B;997 1u8s.pdb B 1ub9.pdb B;998 1uaz.pdb B 1ueh.pdb B;999 1ufo B 1ukk.pdb B;1000 1ujq.pdb B 1un6_2.pdb D;

Random set 5

1 12as.pdb B 1a0j.pdb B;2 1a2z.pdb B 1a3q.pdb B;3 1a73.pdb B 1a8y.pdb B;4 1adu.pdb B 1agq.pdb B;5 1ais.pdb B 1am7.pdb B;6 1aoh.pdb B 1aqu.pdb B;7 1auo.pdb B 1avf_2.pdb J;8 1avz.pdb B 1ay2.pdb B;9 1b1z.pdb B 1b2p.pdb B;10 1b49.pdb C 1b63.pdb B;11 1b6u.pdb B 1b8z.pdb B;12 1bdb.pdb B 1bec.pdb B;13 1bgf.pdb B 1bhh.pdb B;14 1bml.pdb B 1bmt.pdb B;15 1bqp B 1bsl.pdb B;16 1btk.pdb B 1bvp_2.pdb 5;17 1bzq N 1c0p.pdb B;18 1c3k.pdb B 1c5e.pdb B;19 1c9o.pdb B 1cbk.pdb B;20 1cd9.pdb B 1cfm.pdb B;21 1chm.pdb B 1cjx.pdb B;22 1cnz.pdb B 1cpb.pdb B;23 1cs1.pdb B 1ct9_2.pdb C;24 1cxp.pdb C 1cy9.pdb B;25 1d0q.pdb B 1d2e.pdb B;26 1d2s.pdb B 1d5f.pdb B;27 1d9c.pdb B 1dbt_1.pdb B;28 1dd3 B 1df4.pdb B;29 1dj7.pdb B 1djt.pdb B;30 1dl5.pdb B 1dn1.pdb B;31 1dov.pdb B 1dqs.pdb B;32 1dvp.pdb B 1dxy.pdb B;33 1dyw.pdb B 1e19.pdb B;34 1e50 A 1e5r.pdb B;35 1e7n.pdb B 1e9g.pdb B;36 1eak C 1ecf.pdb B;37 1eeq.pdb B 1ef8.pdb B;38 1eg4.pdb P 1egw_2.pdb D;39 1eku.pdb B 1elu.pdb B;40 1eo6.pdb B 1ept.pdb A;41 1ern.pdb B 1euw.pdb B;42 1ewy C 1ext.pdb B;43 1ezg.pdb B 1f02.pdb T;44 1f2u.pdb B 1f38.pdb B;45 1f3u F 1f51_2.pdb D;46 1f5v.pdb B 1f86.pdb B;47 1fbt.pdb B 1fcd_1.pdb C;48 1fe8 J 1fgu.pdb B;49 1fm0.pdb E 1fno.pdb B;50 1fp1.pdb A 1fqj_1.pdb B;51 1fr2.pdb B 1fs8.pdb B;52 1fwx D 1fxz.pdb B;53 1g1j.pdb B 1g4u.pdb R;54 1g8k F 1g8q.pdb B;55 1gc1 C 1ge7.pdb B;56 1gg6.pdb C 1gk6.pdb A;57 1gmv.pdb B 1goi.pdb B;58 1gpm.pdb B 1gqn.pdb B;59 1gtw.pdb B 1gu7.pdb B;60 1gvn.pdb B 1gwn.pdb C;61 1gxc B 1gy6.pdb B;62 1gzj.pdb B 1h16.pdb B;63 1h21.pdb B 1h3o_1.pdb B;64 1h6o.pdb B 1h72.pdb A;65 1h8g.pdb B 1h9r.pdb B;66 1hbx E 1hdh.pdb B;67 1hh2.pdb A 1hk7.pdb B;68 1hlo.pdb B 1hnj.pdb B;69 1htr.pdb P 1huw.pdb B;70 1hw7.pdb B 1hxp.pdb B;71 1hyl.pdb B 1hzy.pdb B;72 1i31.pdb P 1i49.pdb B;73 1i52.pdb B 1i7b.pdb A;74 1ibr D 1icf_2.pdb D;75 1ied.pdb B 1ihb.pdb B;76 1ii7.pdb B 1im3_3.pdb L;77 1isy.pdb B 1itv.pdb B;78 1ix2.pdb B 1izm.pdb B;79 1j3b.pdb B 1j3w.pdb B;80 1j7n.pdb B 1ja3.pdb B;81 1jay.pdb B 1jdf_1.pdb B;82 1jg8.pdb B 1jhd.pdb B;83 1ji7.pdb B 1jk9_2.pdb D;84 1jlv B 1jm6.pdb B;85 1joc.pdb B 1jpy_1.pdb B;86 1jqp.pdb B 1js1.pdb Y;87 1jvm.pdb B 1jxp.pdb B;88 1jys.pdb B 1k1e_3.pdb J;89 1k5n.pdb B 1k6f_2.pdb E;90 1k94.pdb B 1kam.pdb B;91 1kc7.pdb B 1kez_1.pdb B;92 1ki1 D 1kix.pdb C;93 1kk8.pdb B 1klo.pdb B;94 1kor.pdb B 1kpt.pdb B;95 1ktb.pdb B 1ku2.pdb B;96 1kut.pdb B 1kzq.pdb B;97 1l3p.pdb B 1l5r.pdb B;98 1l7d D 1lc5.pdb B;99 1lj9.pdb B 1lkt_1.pdb B;100 1lm8.pdb C 1lom.pdb B;101 1lq9.pdb B 1lt8.pdb B;102 1lxd.pdb B 1lyw_2.pdb D;103 1m1l.pdb B 1m2v.pdb B;104 1m6d.pdb B 1m6p.pdb B;105 1m9x C 1mcw.pdb M;106 1mg1.pdb B 1mi3_2.pdb D;107 1mkf.pdb B 1ml0.pdb D;108 1mo9.pdb B 1mp4.pdb B;109 1msc.pdb B 1mu4.pdb B;110 1mvk J 1mxb.pdb B;111 1my7.pdb B 1n0l_1.pdb B;112 1n2m E 1n55.pdb B;113 1n7h.pdb B 1n9p.pdb B;114 1nco.pdb B 1ne2.pdb B;115 1nf3 C 1nh2.pdb B;116 1ni4.pdb B 1nkp_1.pdb B;117 1nme.pdb B 1nn5.pdb B;118 1nox.pdb B 1nqk.pdb B;119 1nt2.pdb B 1nu9_2.pdb F;120 1nvm B 1nw9.pdb B;121 1nxj B 1nzi.pdb B;122 1o4w.pdb B 1o54.pdb B;123 1o5h.pdb B 1o6e.pdb B;124 1o89.pdb B 1o94_1.pdb B;125 1oa8.pdb B 1ob9.pdb B;126 1obx.pdb C 1oe0.pdb B;127 1og6.pdb B 1oi0.pdb B;128 1oio.pdb B 1ok7.pdb B;129 1omw.pdb B 1on2.pdb B;130 1ope.pdb B 1or4.pdb B;131 1oru.pdb B 1otk.pdb B;132 1ox0.pdb B 1oyn.pdb B;133 1p0x.pdb B 1p2j.pdb I;134 1p74.pdb B 1p7q.pdb D;135 1p9o.pdb B 1pcs.pdb B;136 1pdo.pdb B 1pfo.pdb B;137 1pk1 B 1pkq_1.pdb B;138 1pm3.pdb B 1ppr.pdb N;139 1pv1.pdb B 1pvg.pdb B;140 1pxv C 1pzs.pdb B;141 1q0q.pdb B 1q3g_2.pdb F;142 1q67.pdb B 1q7e.pdb B;143 1q98.pdb B 1qb2.pdb B;144 1qh3.pdb B 1qhd.pdb B;145 1qj8.pdb B 1qki_1.pdb B;146 1qks.pdb B 1qmg_2.pdb D;147 1qoz.pdb B 1qpo_2.pdb D;148 1qre.pdb B 1qtn.pdb B;149 1qx4.pdb B 1qyc.pdb B;150 1qzq.pdb B 1r11.pdb B;151 1r1k.pdb A 1r43.pdb B;152 1r7a.pdb B 1r8d.pdb B;153 1r8s.pdb E 1rcu.pdb B;154 1rgf.pdb B 1rh5.pdb B;155 1rj9.pdb B 1rke.pdb B;156 1rmd.pdb B 1rqb.pdb B;157 1rw0.pdb B 1ry9_1.pdb B;158 1rz1 B 1s0p.pdb B;159 1s5a.pdb B 1s6v_1.pdb B;160 1s7m E 1s98.pdb B;161 1sbb.pdb B 1scj.pdb B;162 1sfp.pdb B 1sg4.pdb B;163 1sh5.pdb B 1sjw.pdb B;164 1sox.pdb B 1sq2.pdb N;165 1sqs.pdb B 1srq_2.pdb D;166 1stz.pdb B 1svp.pdb B;167 1sz2.pdb B 1t06.pdb B;168 1t11.pdb B 1t3c.pdb B;169 1t5o D 1t6f.pdb B;170 1t70 H 1t8t.pdb B;171 1t9i.pdb B 1tc1.pdb B;172 1tf0.pdb B 1th8.pdb B;173 1tj7.pdb B 1tlk.pdb B;174 1tqb.pdb B 1trr_1.pdb B;175 1tue L 1tuw.pdb B;176 1tvn.pdb B 1tx4.pdb B;177 1tz9.pdb B 1u00.pdb P;178 1u19.pdb B 1u5x.pdb B;179 1u8s.pdb B 1u9t.pdb B;180 1uaz.pdb B 1uc2.pdb B;181 1udd.pdb B 1ufh.pdb B;182 1ujq.pdb B 1uku.pdb B;183 1um0.pdb B 1unh.pdb B;184 1us7.pdb B 1usi.pdb C;185 1ut7.pdb B 1uth.pdb B;186 1uuj.pdb B 1uw4_1.pdb B;187 1uzb.pdb B 1v05.pdb B;188 1v1p.pdb B 1v3v.pdb B;189 1v72.pdb B 1v7l.pdb B;190 1v8c B 1v96.pdb B;191 1vbk.pdb B 1vdd.pdb B;192 1vg0.pdb B 1vh5.pdb B;193 1vi0.pdb B 1viu_1.pdb B;194 1vka.pdb B 1vki.pdb B;195 1vl0.pdb B 1vlj.pdb B;196 1vme.pdb B 1vpb.pdb B;197 1vrs E 1vyt_1.pdb E;198 1w07.pdb B 1w2w_2.pdb F;199 1w6g.pdb B 1w7j.pdb B;200 1wa5.pdb B 1wdk.pdb B;201 1who.pdb B 1wlu.pdb B;202 1wq1.pdb G 1wtd.pdb B;203 1wud.pdb B 1wvf.pdb B;204 1wyi.pdb B 1wyz_1.pdb C;205 1x3m.pdb B 1x79.pdb B;206 1x8z C 1xb4_1.pdb B;207 1xdt.pdb R 1xex.pdb B;208 1xgs.pdb B 1xiw_1.pdb B;209 1xm3.pdb B 1xnf.pdb B;210 1xpj.pdb B 1xqb.pdb B;211 1xs0 B 1xub.pdb B;212 1xwr.pdb B 1xzw.pdb B;213 1y0z.pdb B 1y43.pdb B;214 1y7m.pdb B 1y82.pdb B;215 1yak B 1ybe.pdb B;216 1ycc.pdb B 1ydw.pdb B;217 1yj5 B 1yln.pdb B;218 1ynr.pdb B 1yqf_1.pdb B;219 1yu0.pdb B 1yvb.pdb I;220 1yxb F 1z0s.pdb B;221 1z3e.pdb B 1z72.pdb B;222 1zbo.pdb B 1zc3_1.pdb D;223 1zed.pdb B 1zk4.pdb B;224 1zp6.pdb B 1zpv.pdb B;225 1zrs.pdb B 1zun.pdb B;226 1zvp D 2a26_1.pdb B;227 2a5t.pdb B 2a6c_1.pdb B;228 2a99.pdb B 2aal_1.pdb B;229 2af4.pdb D 2aho.pdb B;230 2aj7.pdb B 2amx.pdb B;231 2arh.pdb B 2avd.pdb B;232 2ayo.pdb B 2aze.pdb B;233 2b0l B 2b59.pdb B;234 2b9c.pdb B 2bay_2.pdb C;235 2bcn.pdb B 2bex_2.pdb D;236 2bgw.pdb B 2bkr.pdb B;237 2bp3 T 2bw3.pdb B;238 2c12 B 2c35_4.pdb H;239 2ca1.pdb B 2cay.pdb B;240 2ch5 B 2cm3.pdb B;241 2cvz.pdb B 2cx3.pdb B;242 2cz1.pdb B 2d32.pdb B;243 2d6y.pdb B 2dg5_1.pdb B;244 2e2d.pdb C 2ejn.pdb B;245 2eul B 2f02.pdb B;246 2f3d.pdb B 2f4l_2.pdb D;247 2f69.pdb B 2f6u.pdb B;248 2f96.pdb B 2fbh.pdb B;249 2fex B 2ffg.pdb B;250 2fhq.pdb B 2fl4.pdb B;251 2fp4.pdb B 2fql.pdb B;252 2ftw.pdb B 2fun_1.pdb B;253 2fzt.pdb B 2g3a.pdb B;254 2g7s.pdb B 2ga1.pdb B;255 2gc7 F 2gf6_1.pdb B;256 2gj7.pdb F 2glt.pdb B;257 2gsm B 2gv8.pdb B;258 2gyq.pdb B 2h6f.pdb B;259 2hew.pdb A 2hiq.pdb B;260 2hoe.pdb B 2hrc.pdb B;261 2i4j.pdb B 2i7r.pdb B;262 2idj.pdb B 2iid_2.pdb D;263 2ijd A 2iuw.pdb B;264 2jhf.pdb B 2mlt.pdb B;265 2nlz.pdb B 2nrh.pdb B;266 2o23.pdb B 2oat.pdb B;267 2otx.pdb B 2p54.pdb B;268 2pg2.pdb B 2por.pdb B;269 2rln.pdb E 2scu.pdb B;270 2tnf.pdb B 2wea.pdb B;271 3grs.pdb B 3il8.pdb B;272 3pfk.pdb B 3sic.pdb I;273 4ake.pdb B 4ovo.pdb B;274 12as.pdb B 1a25.pdb B;275 1a12.pdb B 1a64.pdb B;276 1a73.pdb B 1ab8.pdb B;277 1aa7.pdb B 1aih_1.pdb B;278 1adu.pdb B 1aoc.pdb B;279 1aoh.pdb B 1au7.pdb B;280 1aro.pdb L 1avw.pdb B;281 1avz.pdb B 1azw.pdb B;282 1ayf.pdb B 1b3t.pdb B;283 1b1z.pdb B 1b6r.pdb B;284 1b6u.pdb B 1bb9.pdb B;285 1b9c.pdb B 1bg6.pdb B;286 1bgf.pdb B 1bkb.pdb B;287 1bi7.pdb B 1bow.pdb B;288 1bml.pdb B 1btg.pdb B;289 1btk.pdb B 1byi.pdb B;290 1bw0.pdb B 1c3c.pdb B;291 1c3k.pdb B 1c7s.pdb B;292 1c76.pdb B 1cbq.pdb B;293 1c9o.pdb B 1cg6.pdb B;294 1chm.pdb B 1clx_2.pdb D;295 1cku.pdb B 1cru.pdb B;296 1cs1.pdb B 1cun.pdb B;297 1ctt.pdb B 1d02.pdb B;298 1cxp.pdb C 1d2o.pdb B;299 1d2s.pdb B 1d8h_2.pdb C;300 1d6r.pdb I 1dcf.pdb B;301 1dd3 B 1dhf.pdb B;302 1dfn.pdb B 1dkg.pdb B;303 1dj7.pdb B 1dos.pdb B;304 1dov.pdb B 1dun.pdb B;305 1dqz.pdb B 1dyo.pdb B;306 1dyw.pdb B 1e3h.pdb B;307 1e1z.pdb A 1e6j.pdb L;308 1e50 A 1eaj.pdb B;309 1eak C 1edt.pdb B;310 1ecm.pdb B 1efu.pdb B;311 1eg4.pdb P 1eja.pdb B;312 1ei5.pdb B 1ems.pdb B;313 1eku.pdb B 1eqt.pdb B;314 1ern.pdb B 1ew3.pdb B;315 1ev7.pdb B 1eyv.pdb B;316 1ezg.pdb B 1f1m_2.pdb D;317 1f0c.pdb B 1f3g.pdb B;318 1f2u.pdb B 1f5q_2.pdb D;319 1f5v.pdb B 1f9z.pdb B;320 1f8e.pdb B 1fe0.pdb B;321 1fe8 J 1flg.pdb B;322 1fiu.pdb B 1fo1.pdb B;323 1fm0.pdb E 1fqv_3.pdb F;324 1fr2.pdb B 1fua.pdb B;325 1fsg.pdb C 1g0s.pdb B;326 1g1j.pdb B 1g60.pdb B;327 1g5g E 1g8x.pdb B;328 1g8k F 1gg1.pdb B;329 1gg6.pdb C 1gl2.pdb B;330 1gka.pdb B 1gpe.pdb B;331 1gpm.pdb B 1gt3.pdb B;332 1gr0.pdb B 1gve.pdb B;333 1gtw.pdb B 1gx5.pdb B;334 1gxc B 1gz5.pdb B;335 1gyg B 1h1w.pdb B;336 1h21.pdb B 1h5b_2.pdb D;337 1h4p.pdb B 1h7z.pdb B;338 1h6o.pdb B 1hav.pdb B;339 1hbx E 1hf2_1.pdb B;340 1he1 C 1hle.pdb B;341 1hlo.pdb B 1hru.pdb B;342 1hqr.pdb B 1hvv.pdb B;343 1htr.pdb P 1hyh.pdb B;344 1hyl.pdb B 1i2k.pdb B;345 1i0r.pdb B 1i4m.pdb B;346 1i52.pdb B 1ia9.pdb B;347 1i85.pdb B 1id1.pdb B;348 1ibr D 1ii5.pdb B;349 1ii7.pdb B 1ire.pdb B;350 1iom.pdb B 1ivy.pdb B;351 1ix2.pdb B 1j2g.pdb B;352 1j1b.pdb B 1j5s.pdb B;353 1j3b.pdb B 1jat.pdb B;354 1jay.pdb B 1jey.pdb B;355 1jdp.pdb B 1jhl.pdb A;356 1ji7.pdb B 1jl0.pdb B;357 1jkj B 1jmk.pdb O;358 1jlv B 1jqn.pdb B;359 1jqp.pdb B 1jub.pdb B;360 1js3.pdb B 1jye.pdb B;361 1jys.pdb B 1k4m.pdb B;362 1k3e.pdb B 1k8r.pdb B;363 1k5n.pdb B 1kbj.pdb B;364 1kc7.pdb B 1kgy_1.pdb D;365 1kfi.pdb B 1kjn.pdb B;366 1kk8.pdb B 1ko6.pdb C;367 1knc.pdb B 1ksh.pdb B;368 1kor.pdb B 1ku9.pdb B;369 1kut.pdb B 1l3a.pdb B;370 1l0a.pdb B 1l6r.pdb B;371 1l7d D 1lfb.pdb B;372 1ldf.pdb B 1llm.pdb D;373 1lj9.pdb B 1lq1_1.pdb D;374 1lq9.pdb B 1lw7.pdb B;375 1ltx.pdb B 1m0w.pdb B;376 1m1l.pdb B 1m4u.pdb A;377 1m3w.pdb B 1m7y.pdb B;378 1m6d.pdb B 1mf8.pdb B;379 1mg1.pdb B 1mju.pdb H;380 1miw.pdb B 1mml.pdb B;381 1mo9.pdb B 1mr1_2.pdb C;382 1mpy.pdb B 1mv5.pdb B;383 1msc.pdb B 1mxr.pdb B;384 1my7.pdb B 1n1q.pdb B;385 1n12 D 1n71_1.pdb B;386 1n7h.pdb B 1nbf_2.pdb C;387 1naw.pdb B 1neu.pdb B;388 1nco.pdb B 1nhw.pdb B;389 1ni4.pdb B 1nls.pdb B;390 1nkt.pdb B 1nog.pdb B;391 1nox.pdb B 1nsj.pdb B;392 1nrj.pdb B 1nv7.pdb B;393 1nt2.pdb B 1nxh.pdb B;394 1nxj B 1o1x.pdb B;395 1o0s.pdb B 1o5d.pdb H;396 1o5h.pdb B 1o7l.pdb B;397 1o6z.pdb B 1o9l.pdb B;398 1o89.pdb B 1obo.pdb B;399 1obx.pdb C 1oft.pdb B;400 1oe9.pdb B 1oi6.pdb B;401 1oio.pdb B 1olp.pdb B;402 1oki.pdb B 1ooz.pdb B;403 1omw.pdb B 1ors.pdb B;404 1oru.pdb B 1ovm.pdb B;405 1ou0.pdb B 1ozf.pdb B;406 1p0x.pdb B 1p5v.pdb B;407 1p35 B 1p9e.pdb B;408 1p74.pdb B 1pdk.pdb B;409 1pdo.pdb B 1piw.pdb B;410 1pgr F 1pl5.pdb S;411 1pm3.pdb B 1ptm.pdb B;412 1pqw.pdb B 1pwb.pdb B;413 1pv1.pdb B 1q0a.pdb B;414 1q0q.pdb B 1q4t.pdb B;415 1q40 D 1q8f.pdb B;416 1q98.pdb B 1qdn.pdb B;417 1qc7.pdb B 1qi9.pdb B;418 1qh3.pdb B 1qkr.pdb B;419 1qks.pdb B 1qo8.pdb D;420 1qmu.pdb B 1qqg.pdb B;421 1qre.pdb B 1qvz.pdb B;422 1qup.pdb B 1qz8.pdb B;423 1qx4.pdb B 1r1d.pdb B;424 1r1k.pdb A 1r61.pdb B;425 1r4m F 1r8j.pdb B;426 1r8s.pdb E 1rer.pdb B;427 1re3 B 1ris.pdb B;428 1rgf.pdb B 1rlz.pdb B;429 1rmd.pdb B 1ru0.pdb B;430 1rqi.pdb B 1ryi_2.pdb B;431 1rz1 B 1s3o.pdb B;432 1s1q D 1s7h_2.pdb D;433 1s5a.pdb B 1sb7.pdb B;434 1sbb.pdb B 1sek.pdb B;435 1sdd.pdb B 1sgm.pdb B;436 1sh5.pdb B 1smo.pdb B;437 1skz.pdb B 1sqe.pdb B;438 1sox.pdb B 1stf.pdb I;439 1stz.pdb B 1sy6.pdb H;440 1svx.pdb B 1t0h.pdb B;441 1t11.pdb B 1t4b.pdb B;442 1t3l.pdb B 1t6n.pdb B;443 1t5o D 1t9b.pdb B;444 1t9i.pdb B 1te2.pdb B;445 1tco.pdb B 1tht.pdb B;446 1tj7.pdb B 1to3.pdb B;447 1tm0.pdb B 1ttw.pdb B;448 1tqb.pdb B 1tvl.pdb B;449 1tvn.pdb B 1ty0_1.pdb B;450 1txg.pdb B 1u0m.pdb B;451 1u19.pdb B 1u7g.pdb B;452 1u6g.pdb B 1uad_2.pdb D;453 1u8s.pdb B 1ud0_1.pdb B;454 1udd.pdb B 1uis.pdb B;455 1ufo B 1ulk.pdb B;456 1um0.pdb B 1uqt.pdb B;457 1unn.pdb B 1usq_2.pdb B;458 1us7.pdb B 1uuh.pdb B;459 1uuj.pdb B 1uxt.pdb B;460 1uwk.pdb B 1v13.pdb B;461 1v1p.pdb B 1v5v.pdb B;462 1v4v.pdb B 1v7p.pdb B;463 1v72.pdb B 1v9z.pdb B;464 1vbk.pdb B 1vef.pdb B;465 1vdw.pdb B 1vhx.pdb B;466 1vi0.pdb B 1vjo.pdb B;467 1vj2.pdb B 1vkn.pdb B;468 1vka.pdb B 1vlu_2.pdb B;469 1vme.pdb B 1vr0_1.pdb B;470 1vpz.pdb B 1vzi.pdb B;471 1w07.pdb B 1w53.pdb B;472 1w36 F 1w9e.pdb B;473 1w6g.pdb B 1whi.pdb B;474 1who.pdb B 1wok.pdb B;475 1wmh.pdb B 1wu7.pdb B;476 1wud.pdb B 1wwr_2.pdb D;477 1ww1.pdb B 1x1z.pdb B;478 1wyi.pdb B 1x82.pdb B;479 1x8z C 1xdi.pdb B;480 1xcf.pdb B 1xg5.pdb B;481 1xgs.pdb B 1xku.pdb B;482 1xk7 B 1xoc.pdb B;483 1xm3.pdb B 1xrs.pdb B;484 1xs0 B 1xvi.pdb B;485 1xuv B 1y0h.pdb B;486 1y0z.pdb B 1y6k.pdb R;487 1y4m.pdb B 1y9b.pdb B;488 1y7m.pdb B 1yc5.pdb B;489 1ycc.pdb B 1yg2.pdb B;490 1yer.pdb B 1ynb.pdb B;491 1ynr.pdb B 1ys7.pdb B;492 1yrb.pdb B 1yw6.pdb B;493 1yu0.pdb B 1z2l.pdb B;494 1z3e.pdb B 1z9w.pdb B;495 1z84.pdb B 1zd3.pdb B;496 1zed.pdb B 1zmb_3.pdb F;497 1zke E 1zq9.pdb B;498 1zp6.pdb B 1zvb.pdb B;499 1zvp D 2a42.pdb B;500 2a2q.pdb H 2a7w_2.pdb F;501 2a99.pdb B 2ae6.pdb B;502 2aca.pdb B 2aib.pdb B;503 2af4.pdb D 2arc.pdb B;504 2arh.pdb B 2axp.pdb B;505 2avp.pdb B 2b0c.pdb B;506 2b0l B 2b69.pdb B;507 2b5g.pdb B 2bbk.pdb J;508 2b9c.pdb B 2bgc_3.pdb G;509 2bgw.pdb B 2bnx.pdb B;510 2bky.pdb B 2bz1.pdb B;511 2c12 B 2c5w.pdb A;512 2c4n.pdb B 2cch_1.pdb B;513 2ca1.pdb B 2cn1.pdb B;514 2cn4.pdb B 2cy5.pdb B;515 2cvz.pdb B 2d45.pdb B;516 2d6y.pdb B 2dtr.pdb B;517 2dp9.pdb B 2esr.pdb B;518 2e2d.pdb C 2f0c.pdb B;519 2f1f.pdb B 2f5k_2.pdb D;520 2f3d.pdb B 2f8a.pdb B;521 2f96.pdb B 2fcw.pdb B;522 2fbk.pdb B 2fge_2.pdb E;523 2fex B 2fm8.pdb B;524 2fml.pdb B 2frh.pdb B;525 2fp4.pdb B 2fyi_3.pdb D;526 2fzt.pdb B 2g6t.pdb B;527 2g40.pdb B 2gau.pdb B;528 2g7s.pdb B 2gfq.pdb B;529 2gh1.pdb B 2gm4.pdb B;530 2gj7.pdb F 2gwg.pdb B;531 2gyq.pdb B 2hbo.pdb B;532 2h7z.pdb B 2hlj.pdb B;533 2hew.pdb A 2hth.pdb B;534 2hu7.pdb B 2iba.pdb B;535 2i4j.pdb B 2iiz.pdb B;536 2ijd A 2j0w.pdb B;537 2ixc.pdb B 2mys.pdb B;538 2jhf.pdb B 2nuj.pdb B;539 2nul.pdb B 2odk_1.pdb B;540 2o23.pdb B 2paq.pdb B;541 2pg2.pdb B 2q47.pdb B;542 2pva.pdb B 2spc.pdb B;543 2rln.pdb E 3cd4.pdb B;544 3cla.pdb B 3kin.pdb B;545 3grs.pdb B 3vub.pdb B;546 4ake.pdb B 5p21.pdb B;547 137l.pdb B 1a6j.pdb B;548 1a17.pdb B 1aap.pdb B;549 1a78.pdb B 1ail.pdb B;550 1aap.pdb B 1aoh.pdb B;551 1ajs.pdb B 1aua.pdb B;552 1aox.pdb B 1avy_3.pdb B;553 1as4.pdb B 1ayo.pdb B;554 1awc.pdb B 1b43.pdb B;555 1ayo.pdb B 1b6u.pdb B;556 1b4w.pdb B 1bbp.pdb B;557 1b74.pdb B 1bg9.pdb B;558 1b9e.pdb B 1bih.pdb B;559 1bgp.pdb B 1bpl.pdb B;560 1bih.pdb B 1btk.pdb B;561 1bqu.pdb B 1byk.pdb B;562 1btn.pdb B 1c3g.pdb B;563 1bwn.pdb B 1c7c.pdb B;564 1c3r.pdb B 1cby.pdb B;565 1c7c.pdb B 1chm.pdb B;566 1ce9.pdb B 1cmc.pdb B;567 1cho.pdb I 1crx.pdb B;568 1cl7.pdb H 1cu1.pdb B;569 1cs6.pdb B 1d0c.pdb B;570 1cu1.pdb B 1d2s.pdb B;571 1d1j.pdb B 1d8l.pdb B;572 1d2z D 1dcs.pdb B;573 1d7b.pdb B 1dg1.pdb H;574 1ddt.pdb B 1dku.pdb B;575 1dg1.pdb H 1dov.pdb B;576 1dle.pdb B 1duv.pdb H;577 1dpg.pdb B 1dys.pdb B;578 1dru.pdb B 1e20.pdb B;579 1dza.pdb B 1e6u.pdb B;580 1e20.pdb B 1eak_1.pdb C;581 1e85.pdb B 1edz.pdb B;582 1ear.pdb B 1eg1.pdb C;583 1ecs.pdb B 1ei6_1.pdb D;584 1eg5.pdb B 1emu.pdb B;585 1ei6 D 1ern.pdb B;586 1ep3.pdb B 1ew6.pdb B;587 1erv.pdb B 1ez0_1.pdb D;588 1evj C 1f0k.pdb B;589 1ezi.pdb B 1f3h.pdb B;590 1f0k.pdb B 1f5v.pdb B;591 1f3v.pdb B 1fa2.pdb B;592 1f60.pdb B 1fe6.pdb B;593 1f8f.pdb B 1fjj.pdb B;594 1few.pdb B 1foc.pdb B;595 1fjj.pdb B 1fr2.pdb B;596 1fp2.pdb B 1fui_2.pdb E;597 1fr8.pdb B 1g0y.pdb I;598 1fsk K 1g5h_2.pdb D;599 1g29.pdb 2 1g9m.pdb C;600 1g5h D 1gg6.pdb C;601 1gcj.pdb B 1gl4.pdb B;602 1ggx.pdb B 1gpj.pdb B;603 1gkj.pdb B 1gr7.pdb B;604 1gpq.pdb B 1gvf.pdb B;605 1gr7.pdb B 1gxc_1.pdb B;606 1gvp.pdb B 1gz6.pdb B;607 1gxd C 1h1y.pdb B;608 1gyo.pdb B 1h4r.pdb B;609 1h2b.pdb B 1h80.pdb B;610 1h4r.pdb B 1hbx_2.pdb E;611 1h8p.pdb B 1hfe_2.pdb M;612 1hc7 B 1hlg.pdb B;613 1he7.pdb B 1hqs.pdb B;614 1hlq.pdb B 1hw1.pdb B;615 1hqs.pdb B 1hyl.pdb B;616 1hwg.pdb B 1i2m_2.pdb D;617 1hyn S 1i4u.pdb B;618 1i12 C 1i86.pdb B;619 1i58.pdb B 1idp.pdb B;620 1i86.pdb B 1ii7.pdb B;621 1ig0.pdb B 1iro.pdb B;622 1iil E 1ix1.pdb B;623 1ips.pdb B 1j1d_2.pdb E;624 1ix9.pdb B 1j5w.pdb B;625 1j1d E 1jay.pdb B;626 1j8b.pdb B 1jfi.pdb B;627 1jb7.pdb B 1ji3.pdb B;628 1jdw.pdb B 1jkm.pdb B;629 1jil.pdb B 1jmv_2.pdb D;630 1jkm.pdb B 1jqp.pdb B;631 1jof B 1juv.pdb B;632 1jr7.pdb B 1jyl.pdb B;633 1jsd.pdb B 1k3s.pdb B;634 1jz7.pdb B 1k92.pdb B;635 1k3s.pdb B 1kc7.pdb B;636 1ka2.pdb B 1khd_2.pdb C;637 1kcf.pdb B 1kjq.pdb B;638 1kfu.pdb S 1knq.pdb B;639 1kke.pdb B 1ksi.pdb B;640 1knq.pdb B 1kut.pdb B;641 1ktj.pdb B 1l3c.pdb B;642 1kvd D 1l6x.pdb B;643 1l0o.pdb B 1ldj.pdb B;644 1l7l.pdb B 1llu_1.pdb B;645 1ldj.pdb B 1lq9.pdb B;646 1lmb.pdb 4 1lwd.pdb B;647 1lql D 1m1f.pdb B;648 1lua.pdb B 1m3y_1.pdb B;649 1m1n B 1m93.pdb B;650 1m3y B 1mg1.pdb B;651 1ma9.pdb B 1mk2.pdb B;652 1mh9.pdb B 1mnm.pdb B;653 1mjf.pdb B 1mq8_1.pdb B;654 1moe.pdb B 1mv8_2.pdb D;655 1mq8 B 1my7.pdb B;656 1mvo.pdb B 1n26.pdb B;657 1myl B 1n7f.pdb B;658 1n1b.pdb B 1nb5_2.pdb J;659 1n7s.pdb B 1nex_2.pdb D;660 1nb5 J 1ni4.pdb B;661 1nf9.pdb B 1nlt.pdb B;662 1ni5.pdb B 1now_2.pdb B;663 1nkv.pdb B 1nrv.pdb B;664 1noy.pdb B 1nvi.pdb E;665 1nrv.pdb B 1nxj_2.pdb B;666 1nvp.pdb B 1o22.pdb B;667 1nxm.pdb B 1o5e.pdb H;668 1o0v.pdb B 1o75.pdb B;669 1o5k.pdb B 1o9p.pdb B;670 1o75.pdb B 1obx.pdb C;671 1oaa.pdb B 1ofu.pdb B;672 1oc0.pdb B 1oih.pdb B;673 1oey M 1okj_2.pdb D;674 1oj4.pdb B 1op3.pdb H;675 1okj D 1oru.pdb B;676 1oph.pdb B 1ovn.pdb B;677 1orv.pdb B 1ozh.pdb B;678 1ou8 B 1p5b.pdb B;679 1p16 C 1p9h.pdb B;680 1p5b.pdb B 1pdo.pdb B;681 1pbi.pdb B 1pix.pdb B;682 1pe1.pdb B 1pl8_2.pdb B;683 1pgu.pdb B 1pqz.pdb B;684 1pm4.pdb B 1px8.pdb B;685 1pqz.pdb B 1q0q.pdb B;686 1py1 B 1q4u.pdb B;687 1q14.pdb B 1q8r.pdb B;688 1q43 B 1qd1.pdb B;689 1q9u.pdb B 1qiu_2.pdb E;690 1qd1.pdb B 1qks.pdb B;691 1qja.pdb B 1qoj.pdb B;692 1qkz.pdb H 1qqs.pdb B;693 1qnn.pdb B 1quq.pdb B;694 1qrq.pdb B 1qz9.pdb B;695 1quq.pdb B 1r1k.pdb A;696 1r0m D 1r6t.pdb B;697 1r1t.pdb B 1r8o.pdb B;698 1r4w D 1rec.pdb B;699 1r9c.pdb B 1riw.pdb B;700 1rec.pdb B 1rmd.pdb B;701 1rjl.pdb C 1rv1.pdb B;702 1rmr.pdb B 1ryw_2.pdb F;703 1rrm.pdb B 1s28_2.pdb D;704 1rzh.pdb M 1s7i.pdb B;705 1s28 D 1sbb.pdb B;706 1s7o.pdb B 1sf8_2.pdb H;707 1sbs.pdb L 1sgp.pdb I;708 1sdx.pdb E 1slm.pdb B;709 1sh8.pdb B 1sqj.pdb B;710 1slm.pdb B 1stz.pdb B;711 1squ.pdb B 1sy7.pdb B;712 1su1.pdb B 1t0i.pdb B;713 1sw6.pdb B 1t3m.pdb B;714 1t15.pdb B 1t6s.pdb B;715 1t3m.pdb B 1t9i.pdb B;716 1t7r.pdb B 1te5.pdb B;717 1ta3.pdb B 1thw.pdb B;718 1td2.pdb B 1tmc.pdb B;719 1tjc.pdb B 1tu1.pdb B;720 1tmc.pdb B 1tvn.pdb B;721 1tuh.pdb B 1tye_3.pdb F;722 1tvx.pdb B 1u0s.pdb A;723 1txn.pdb B 1u6l.pdb B;724 1u1i.pdb B 1uae.pdb B;725 1u6l.pdb B 1udd.pdb B;726 1ub0.pdb B 1uiu.pdb B;727 1udr.pdb B 1uly.pdb B;728 1ufy.pdb B 1up7_1.pdb B;729 1ump C 1usu.pdb B;730 1up7 B 1uuj.pdb B;731 1ut8.pdb B 1uyj_2.pdb B;732 1uur.pdb B 1v1i.pdb B;733 1uwu.pdb B 1v58_2.pdb B;734 1v25.pdb B 1v7w.pdb B;735 1v58 B 1vbk.pdb B;736 1v8d.pdb B 1vet.pdb B;737 1vbo A 1vhz.pdb B;738 1ve1.pdb B 1vjg.pdb B;739 1vi2.pdb B 1vkp.pdb B;740 1vjg.pdb B 1vme.pdb B;741 1vl4.pdb B 1vr6.pdb B;742 1vmg.pdb B 1vzy.pdb B;743 1vq0.pdb B 1w3b.pdb B;744 1w0m B 1w9q_2.pdb S;745 1w3b.pdb B 1who.pdb B;746 1wbh.pdb B 1wp1_1.pdb B;747 1wht.pdb B 1wu9.pdb B;748 1wmi.pdb B 1ww9.pdb B;749 1wui.pdb L 1x2i.pdb B;750 1ww9.pdb B 1x8z_2.pdb C;751 1x3z.pdb B 1xdl_4.pdb Z;752 1x92.pdb B 1xg7.pdb B;753 1xcg B 1xka.pdb C;754 1xhk.pdb B 1xou.pdb B;755 1xka.pdb C 1xs0_1.pdb B;756 1xpm B 1xvs.pdb B;757 1xs1 B 1y0u.pdb B;758 1xv2 B 1y5h.pdb B;759 1y14.pdb B 1y9i.pdb B;760 1y5h.pdb B 1ycc.pdb B;761 1yav.pdb B 1ygy.pdb B;762 1ycg B 1ynf_2.pdb D;763 1yf5.pdb A 1yre_2.pdb D;764 1yoa.pdb B 1ywq.pdb B;765 1yre D 1z3e.pdb B;766 1yz3.pdb B 1za3_2.pdb L;767 1z41.pdb B 1ze3.pdb D;768 1z8u D 1zkp_1.pdb C;769 1zee.pdb B 1zr4.pdb B;770 1zkp C 1zvp_1.pdb D;771 1zru.pdb B 2a4k.pdb B;772 1zww.pdb B 2a84.pdb B;773 2a2u.pdb B 2adf.pdb H;774 2a9k.pdb B 2aio.pdb B;775 2adf.pdb H 2arh.pdb B;776 2ajf E 2axy_1.pdb B;777 2ark B 2b0j.pdb B;778 2awi D 2b5i.pdb B;779 2b1y.pdb B 2bcg.pdb Y;780 2b5i.pdb B 2bgw.pdb B;781 2bde.pdb B 2bo4_1.pdb B;782 2bgx.pdb B 2bz6.pdb L;783 2bll.pdb B 2c4v.pdb B;784 2c1n C 2cg6.pdb B;785 2c4v.pdb B 2cn4.pdb B;786 2ch7.pdb B 2cy9.pdb B;787 2cnz.pdb B 2d4z.pdb B;788 2cwk.pdb B 2dpi.pdb D;789 2d7c D 2ess.pdb B;790 2dpi.pdb D 2f1f.pdb B;791 2evr.pdb B 2f5v.pdb B;792 2f1k B 2f8f.pdb B;793 2f3x.pdb B 2fbl.pdb B;794 2f9h.pdb B 2fgq.pdb A;795 2fbl.pdb B 2fml.pdb B;796 2fhz.pdb B 2fs2.pdb B;797 2fmt.pdb B 2fzf.pdb B;798 2fpn.pdb B 2g50_2.pdb F;799 2g0t.pdb B 2gb3_1.pdb B;800 2g50 F 2gh1.pdb B;801 2gci B 2gol_1.pdb B;802 2ghp F 2gx9.pdb B;803 2gj8 D 2h9d_2.pdb D;804 2gys.pdb B 2hmf.pdb B;805 2h9d D 2hu7.pdb B;806 2hox B 2ibg_1.pdb F;807 2hwn D 2ij9.pdb B;808 2i52 B 2ixp_2.pdb D;809 2ilk.pdb B 2nac.pdb B;810 2ixp D 2nul.pdb B;811 2nml.pdb B 2omy.pdb B;812 2nvn.pdb B 2pcd_3.pdb O;813 2o25 D 2q3m.pdb B;814 2pgd.pdb B 2tdt.pdb B;815 2q3m.pdb B 3cla.pdb B;816 2toh.pdb B 3lyn.pdb B;817 3cro.pdb R 3ygs.pdb P;818 3gtu D 4ubp.pdb B;819 1a12.pdb B 1a3d.pdb B;820 1a2z.pdb B 1a8o.pdb B;821 1aa7.pdb B 1afw.pdb B;822 1adu.pdb B 1am2.pdb B;823 1ais.pdb B 1aqt.pdb B;824 1aro.pdb L 1ava_2.pdb D;825 1auo.pdb B 1axi.pdb B;826 1ayf.pdb B 1b2k.pdb B;827 1b1z.pdb B 1b5p.pdb B;828 1b49.pdb C 1b8m.pdb B;829 1b9c.pdb B 1bdy.pdb B;830 1bdb.pdb B 1bhg.pdb B;831 1bi7.pdb B 1bmq.pdb B;832 1bml.pdb B 1brw.pdb B;833 1bqp B 1bvn.pdb T;834 1bw0.pdb B 1c0m_1.pdb B;835 1bzq N 1c4z.pdb B;836 1c76.pdb B 1cbf.pdb B;837 1c9o.pdb B 1cf7.pdb B;838 1cd9.pdb B 1cjt.pdb B;839 1cku.pdb B 1cp2.pdb B;840 1cnz.pdb B 1csn.pdb B;841 1ctt.pdb B 1cxz.pdb B;842 1cxp.pdb C 1d1z.pdb B;843 1d0q.pdb B 1d4x.pdb G;844 1d6r.pdb I 1dbq.pdb B;845 1d9c.pdb B 1dev_1.pdb B;846 1dfn.pdb B 1djs.pdb B;847 1dj7.pdb B 1dmu.pdb C;848 1dl5.pdb B 1dqn.pdb B;849 1dqz.pdb B 1dxx.pdb B;850 1dvp.pdb B 1e0b.pdb B;851 1e1z.pdb A 1e5d.pdb B;852 1e50 A 1e96.pdb B;853 1e7n.pdb B 1ec8.pdb B;854 1ecm.pdb B 1ef1.pdb C;855 1eeq.pdb B 1egp.pdb B;856 1ei5.pdb B 1elr.pdb B;857 1eku.pdb B 1epf_2.pdb D;858 1eo6.pdb B 1euv.pdb B;859 1ev7.pdb B 1ex4.pdb B;860 1ewy C 1ezx.pdb C;861 1f0c.pdb B 1f34.pdb B;862 1f2u.pdb B 1f46.pdb B;863 1f3u F 1f7l.pdb B;864 1f8e.pdb B 1fcc.pdb C;865 1fbt.pdb B 1fgj_1.pdb B;866 1fiu.pdb B 1fn9.pdb B;867 1fm0.pdb E 1fq1.pdb B;868 1fp1.pdb A 1fs2.pdb B;869 1fsg.pdb C 1fxw.pdb F;870 1fwx D 1g3n_1.pdb B;871 1g5g E 1g8m.pdb B;872 1g8k F 1gdt.pdb B;873 1gc1 C 1gk1_2.pdb B;874 1gka.pdb B 1go8.pdb A;875 1gmv.pdb B 1gqi.pdb B;876 1gr0.pdb B 1gu3.pdb B;877 1gtw.pdb B 1gwi.pdb B;878 1gvn.pdb B 1gxy.pdb B;879 1gyg B 1h0d.pdb B;880 1gzj.pdb B 1h3f.pdb B;881 1h4p.pdb B 1h6u.pdb B;882 1h6o.pdb B 1h9m_1.pdb B;883 1h8g.pdb B 1hcx.pdb B;884 1he1 C 1hjx.pdb B;885 1hh2.pdb A 1hng.pdb B;886 1hqr.pdb B 1hur.pdb B;887 1htr.pdb P 1hxm_3.pdb F;888 1hw7.pdb B 1hzh.pdb K;889 1i0r.pdb B 1i3k.pdb B;890 1i31.pdb P 1i75.pdb B;891 1i85.pdb B 1ic2_1.pdb B;892 1ibr D 1igq_1.pdb C;893 1ied.pdb B 1ilr.pdb 2;894 1iom.pdb B 1itu.pdb B;895 1isy.pdb B 1iyb.pdb B;896 1j1b.pdb B 1j3m.pdb B;897 1j3b.pdb B 1j9l.pdb B;898 1j7n.pdb B 1jd0.pdb B;899 1jdp.pdb B 1jhc.pdb B;900 1jg8.pdb B 1jk4.pdb B;901 1jkj B 1jm0_1.pdb B;902 1jlv B 1jps.pdb H;903 1joc.pdb B 1js0.pdb B;904 1js3.pdb B 1jwy.pdb B;905 1jvm.pdb B 1k0d_1.pdb B;906 1k3e.pdb B 1k6d.pdb B;907 1k5n.pdb B 1kae.pdb B;908 1k94.pdb B 1kdg.pdb B;909 1kfi.pdb B 1kij.pdb B;910 1ki1 D 1klg.pdb B;911 1knc.pdb B 1kps_2.pdb D;912 1kor.pdb B 1ktz.pdb B;913 1ktb.pdb B 1kyq_2.pdb C;914 1l0a.pdb B 1l5o.pdb B;915 1l3p.pdb B 1lb6.pdb B;916 1ldf.pdb B 1lk5.pdb B;917 1lj9.pdb B 1log.pdb B;918 1lm8.pdb C 1lss_1.pdb B;919 1ltx.pdb B 1ly1.pdb B;920 1lxd.pdb B 1m2t.pdb B;921 1m3w.pdb B 1m6k.pdb B;922 1m6d.pdb B 1mby.pdb B;923 1m9x C 1mi1.pdb B;924 1miw.pdb B 1mkz.pdb B;925 1mkf.pdb B 1moy.pdb B;926 1mpy.pdb B 1mtp.pdb B;927 1msc.pdb B 1mwv.pdb B;928 1mvk J 1mzn_1.pdb B;929 1n12 D 1n4q_2.pdb D;930 1n2m E 1n93.pdb A;931 1naw.pdb B 1nd6_2.pdb D;932 1nco.pdb B 1ngm_2.pdb F;933 1nf3 C 1nkn_2.pdb D;934 1nkt.pdb B 1nmu_1.pdb B;935 1nme.pdb B 1nqd.pdb B;936 1nrj.pdb B 1ntv.pdb B;937 1nt2.pdb B 1nw2_2.pdb F;938 1nvm B 1nyt.pdb B;939 1o0s.pdb B 1o51.pdb B;940 1o4w.pdb B 1o6a.pdb B;941 1o6z.pdb B 1o91.pdb B;942 1o89.pdb B 1oaz_1.pdb H;943 1oa8.pdb B 1od4.pdb B;944 1oe9.pdb B 1ohw_2.pdb D;945 1og6.pdb B 1ok3.pdb B;946 1oki.pdb B 1on0.pdb B;947 1omw.pdb B 1or0_2.pdb D;948 1ope.pdb B 1otj_2.pdb C;949 1ou0.pdb B 1oya.pdb B;950 1ox0.pdb B 1p27.pdb B;951 1p35 B 1p7n.pdb B;952 1p74.pdb B 1pc6.pdb B;953 1p9o.pdb B 1pff.pdb B;954 1pgr F 1pkp.pdb B;955 1pk1 B 1poc.pdb B;956 1pqw.pdb B 1pvd.pdb B;957 1pv1.pdb B 1pym.pdb B;958 1pxv C 1q2w.pdb B;959 1q40 D 1q77.pdb B;960 1q67.pdb B 1qav.pdb B;961 1qc7.pdb B 1qha.pdb B;962 1qh3.pdb B 1qkd.pdb B;963 1qj8.pdb B 1qme.pdb B;964 1qmu.pdb B 1qpa.pdb B;965 1qoz.pdb B 1qsm.pdb B;966 1qup.pdb B 1qxm.pdb B;967 1qx4.pdb B 1r0v_2.pdb D;968 1qzq.pdb B 1r3s.pdb B;969 1r4m F 1r89.pdb B;970 1r7a.pdb B 1rcq.pdb B;971 1re3 B 1rh4.pdb B;972 1rgf.pdb B 1rkd.pdb B;973 1rj9.pdb B 1rq2.pdb B;974 1rqi.pdb B 1rwz.pdb B;975 1rw0.pdb B 1rzr_1.pdb C;976 1s1q D 1s6c.pdb B;977 1s5a.pdb B 1s96.pdb B;978 1s7m E 1scf_1.pdb B;979 1sdd.pdb B 1sg1.pdb B;980 1sfp.pdb B 1sjp.pdb B;981 1skz.pdb B 1sq0.pdb B;982 1sox.pdb B 1sr9.pdb B;983 1sqs.pdb B 1svd.pdb M;984 1svx.pdb B 1szq.pdb B;985 1sz2.pdb B 1t2d.pdb B;986 1t3l.pdb B 1t6b.pdb Y;987 1t5o D 1t8q_2.pdb C;988 1t70 H 1tbx.pdb B;989 1tco.pdb B 1tgs.pdb I;990 1tf0.pdb B 1tlj.pdb B;991 1tm0.pdb B 1trb.pdb B;992 1tqb.pdb B 1tuv.pdb B;993 1tue L 1tx3_2.pdb D;994 1txg.pdb B 1tzp.pdb B;995 1tz9.pdb B 1u5k.pdb B;996 1u6g.pdb B 1u9d.pdb B;997 1u8s.pdb B 1uby.pdb B;998 1uaz.pdb B 1uf5.pdb B;999 1ufo B 1ukl_2.pdb E;1000 1ujq.pdb B 1un8.pdb B;

Random set 6

1 1a12.pdb B 1a1x.pdb B;2 1a2z.pdb B 1a4i.pdb B;3 1a73.pdb B 1a99_1.pdb B;4 1adu.pdb B 1ahs.pdb B;5 1ais.pdb B 1amp.pdb B;6 1aro.pdb L 1at3.pdb B;7 1auo.pdb B 1avq.pdb B;8 1avz.pdb B 1ay7.pdb B;9 1b1z.pdb B 1b34.pdb B;10 1b49.pdb C 1b65_1.pdb B;11 1b9c.pdb B 1b9m.pdb B;12 1bdb.pdb B 1bf3.pdb B;13 1bgf.pdb B 1bht.pdb B;14 1bml.pdb B 1bo1.pdb B;15 1bqp B 1bsm.pdb B;16 1bw0.pdb B 1bxt.pdb B;17 1bzq N 1c1y.pdb B;18 1c3k.pdb B 1c5x.pdb A;19 1c9o.pdb B 1cbl.pdb B;20 1cd9.pdb B 1cfr.pdb B;21 1cku.pdb B 1cli_2.pdb D;22 1cnz.pdb B 1cq3.pdb B;23 1cs1.pdb B 1ctf.pdb B;24 1cxp.pdb C 1cyw.pdb B;25 1d0q.pdb B 1d2f.pdb B;26 1d6r.pdb I 1d7x.pdb B;27 1d9c.pdb B 1dc1.pdb B;28 1dd3 B 1df9.pdb B;29 1dj7.pdb B 1dk8.pdb B;30 1dl5.pdb B 1dnl.pdb B;31 1dqz.pdb B 1dtd.pdb B;32 1dvp.pdb B 1dy7.pdb B;33 1dyw.pdb B 1e1h.pdb B;34 1e50 A 1e65.pdb B;35 1e7n.pdb B 1e9p.pdb B;36 1ecm.pdb B 1ed1.pdb B;37 1eeq.pdb B 1efa_1.pdb B;38 1eg4.pdb P 1eh9.pdb B;39 1eku.pdb B 1elw_1.pdb C;40 1eo6.pdb B 1epx.pdb B;41 1ev7.pdb B 1evt.pdb B;42 1ewy C 1eye.pdb B;43 1ezg.pdb B 1f07.pdb B;44 1f2u.pdb B 1f39.pdb B;45 1f3u F 1f59_1.pdb C;46 1f8e.pdb B 1f93_2.pdb D;47 1fbt.pdb B 1fcg.pdb B;48 1fe8 J 1fi4.pdb B;49 1fm0.pdb E 1fns.pdb H;50 1fp1.pdb A 1fqk_2.pdb D;51 1fsg.pdb C 1ft9.pdb B;52 1fwx D 1fyh_2.pdb E;53 1g1j.pdb B 1g4y.pdb R;54 1g8k F 1g8s.pdb B;55 1gc1 C 1ge8.pdb B;56 1gka.pdb B 1gkr.pdb B;57 1gmv.pdb B 1got.pdb B;58 1gpm.pdb B 1gqp.pdb B;59 1gtw.pdb B 1gud.pdb B;60 1gvn.pdb B 1gwy.pdb B;61 1gyg B 1gz0_1.pdb F;62 1gzj.pdb B 1h1o.pdb B;63 1h21.pdb B 1h3y.pdb B;64 1h6o.pdb B 1h7b.pdb B;65 1h8g.pdb B 1h9x.pdb B;66 1he1 C 1he9.pdb B;67 1hh2.pdb A 1hkq.pdb B;68 1hlo.pdb B 1hoz.pdb B;69 1htr.pdb P 1hux.pdb B;70 1hw7.pdb B 1hxx.pdb B;71 1i0r.pdb B 1i1r.pdb B;72 1i31.pdb P 1i4d.pdb B;73 1i52.pdb B 1i7n.pdb B;74 1ibr D 1icj.pdb B;75 1ied.pdb B 1ihj_1.pdb D;76 1iom.pdb B 1iqc_2.pdb D;77 1isy.pdb B 1iu8.pdb B;78 1ix2.pdb B 1izn_1.pdb B;79 1j3b.pdb B 1j4n.pdb B;80 1j7n.pdb B 1jad.pdb B;81 1jdp.pdb B 1jeq.pdb B;82 1jg8.pdb B 1jhf.pdb B;83 1ji7.pdb B 1jke_1.pdb D;84 1jlv B 1jma.pdb B;85 1joc.pdb B 1jqj_1.pdb C;86 1js3.pdb B 1jtd.pdb B;87 1jvm.pdb B 1jxz.pdb B;88 1jys.pdb B 1k1f_1.pdb B;89 1k5n.pdb B 1k6y.pdb B;90 1k94.pdb B 1kaw.pdb B;91 1kfi.pdb B 1kgc.pdb E;92 1ki1 D 1kiy.pdb B;93 1kk8.pdb B 1kmh.pdb B;94 1kor.pdb B 1kqp.pdb B;95 1ktb.pdb B 1ku6.pdb B;96 1l0a.pdb B 1l1s.pdb B;97 1l3p.pdb B 1l5s.pdb B;98 1l7d D 1lck.pdb B;99 1lj9.pdb B 1ll2.pdb B;100 1lm8.pdb C 1lp1.pdb B;101 1ltx.pdb B 1lvb_1.pdb C;102 1lxd.pdb B 1m0d_2.pdb D;103 1m1l.pdb B 1m3e_1.pdb B;104 1m6d.pdb B 1m7g_1.pdb B;105 1m9x C 1mdy_2.pdb D;106 1miw.pdb B 1mji.pdb B;107 1mkf.pdb B 1mld_1.pdb B;108 1mo9.pdb B 1mp9.pdb B;109 1msc.pdb B 1mun.pdb B;110 1mvk J 1mxf.pdb B;111 1n12 D 1n1d_2.pdb D;112 1n2m E 1n57.pdb B;113 1n7h.pdb B 1na6.pdb B;114 1nco.pdb B 1ne8.pdb B;115 1nf3 C 1nh8.pdb B;116 1nkt.pdb B 1nln.pdb B;117 1nme.pdb B 1nnw.pdb B;118 1nox.pdb B 1nql.pdb B;119 1nt2.pdb B 1nul.pdb B;120 1nvm B 1nwp.pdb B;121 1o0s.pdb B 1o12.pdb B;122 1o4w.pdb B 1o57_2.pdb D;123 1o5h.pdb B 1o6r.pdb B;124 1o89.pdb B 1o94_3.pdb F;125 1oa8.pdb B 1obb.pdb B;126 1oe9.pdb B 1of3.pdb B;127 1og6.pdb B 1oi2.pdb B;128 1oio.pdb B 1oke.pdb B;129 1omw.pdb B 1oo0.pdb B;130 1ope.pdb B 1or7_2.pdb F;131 1ou0.pdb B 1ov9.pdb B;132 1ox0.pdb B 1oys.pdb B;133 1p0x.pdb B 1p2z.pdb B;134 1p74.pdb B 1p80.pdb B;135 1p9o.pdb B 1pcx.pdb B;136 1pgr F 1pid_1.pdb B;137 1pk1 B 1pkv.pdb B;138 1pm3.pdb B 1ppv.pdb B;139 1pv1.pdb B 1pvh_1.pdb B;140 1pxv C 1pzw.pdb B;141 1q40 D 1q4r.pdb B;142 1q67.pdb B 1q7l_2.pdb D;143 1q98.pdb B 1qb4.pdb B;144 1qh3.pdb B 1qhh.pdb B;145 1qj8.pdb B 1qkk.pdb B;146 1qmu.pdb B 1qo2.pdb B;147 1qoz.pdb B 1qq5.pdb B;148 1qre.pdb B 1qtx.pdb B;149 1qx4.pdb B 1qyn.pdb B;150 1qzq.pdb B 1r12.pdb B;151 1r4m F 1r5i_1.pdb B;152 1r7a.pdb B 1r8e.pdb D;153 1r8s.pdb E 1rcw_2.pdb C;154 1rgf.pdb B 1rhc.pdb B;155 1rj9.pdb B 1rkt.pdb B;156 1rqi.pdb B 1rth.pdb B;157 1rw0.pdb B 1rya.pdb B;158 1rz1 B 1s12_1.pdb D;159 1s5a.pdb B 1s70.pdb B;160 1s7m E 1s9j.pdb B;161 1sdd.pdb B 1sed.pdb B;162 1sfp.pdb B 1sg9_1.pdb C;163 1sh5.pdb B 1sjy.pdb B;164 1sox.pdb B 1sq5_2.pdb B;165 1sqs.pdb B 1sry.pdb B;166 1svx.pdb B 1sx5.pdb B;167 1sz2.pdb B 1t08.pdb B;168 1t11.pdb B 1t3i.pdb B;169 1t5o D 1t6g_2.pdb D;170 1t70 H 1t92.pdb B;171 1tco.pdb B 1tdq.pdb A;172 1tf0.pdb B 1the.pdb B;173 1tj7.pdb B 1tll.pdb B;174 1tqb.pdb B 1tsr.pdb B;175 1tue L 1tv8.pdb B;176 1txg.pdb B 1txv.pdb B;177 1tz9.pdb B 1u07.pdb B;178 1u19.pdb B 1u60_2.pdb D;179 1u8s.pdb B 1u9y.pdb B;180 1uaz.pdb B 1uc3_5.pdb J;181 1ufo B 1uh5.pdb B;182 1ujq.pdb B 1ukw.pdb B;183 1um0.pdb B 1unk.pdb B;184 1us7.pdb B 1usl_3.pdb E;185 1ut7.pdb B 1uty.pdb B;186 1uwk.pdb B 1ux4.pdb B;187 1uzb.pdb B 1v0d.pdb B;188 1v1p.pdb B 1v4e.pdb B;189 1v72.pdb B 1v7m_1.pdb H;190 1v8c B 1v97.pdb B;191 1vdw.pdb B 1ve3_1.pdb B;192 1vg0.pdb B 1vh6.pdb B;193 1vi0.pdb B 1viv.pdb B;194 1vka.pdb B 1vkj.pdb B;195 1vl0.pdb B 1vlr.pdb B;196 1vpz.pdb B 1vqt.pdb B;197 1vrs E 1vz0_4.pdb F;198 1w07.pdb B 1w2y.pdb B;199 1w6g.pdb B 1w8i.pdb B;200 1wa5.pdb B 1wej.pdb H;201 1wmh.pdb B 1wmz_2.pdb D;202 1wq1.pdb G 1wtl.pdb B;203 1wud.pdb B 1wvg.pdb B;204 1wyi.pdb B 1wz3.pdb B;205 1x3m.pdb B 1x7d.pdb B;206 1xcf.pdb B 1xd3_1.pdb B;207 1xdt.pdb R 1xff.pdb B;208 1xgs.pdb B 1xjl.pdb B;209 1xm3.pdb B 1xnx.pdb B;210 1xpj.pdb B 1xrh_4.pdb H;211 1xuv B 1xva.pdb B;212 1xwr.pdb B 1y0b_2.pdb D;213 1y0z.pdb B 1y44.pdb B;214 1y7m.pdb B 1y88.pdb B;215 1yak B 1ybf.pdb B;216 1yer.pdb B 1yfu.pdb B;217 1yj5 B 1ylq.pdb B;218 1ynr.pdb B 1yqh.pdb B;219 1yu0.pdb B 1yvh.pdb B;220 1yxb F 1z1b.pdb B;221 1z84.pdb B 1z94_2.pdb B;222 1zbo.pdb B 1zc6.pdb B;223 1zed.pdb B 1zk8.pdb B;224 1zp6.pdb B 1zq1.pdb B;225 1zrs.pdb B 1zup.pdb B;226 2a2q.pdb H 2a35.pdb B;227 2a5t.pdb B 2a6n.pdb B;228 2a99.pdb B 2ab0.pdb B;229 2af4.pdb D 2ahu.pdb B;230 2aj7.pdb B 2apo.pdb B;231 2avp.pdb B 2axi.pdb B;232 2ayo.pdb B 2b06.pdb B;233 2b0l B 2b5a_2.pdb D;234 2b9c.pdb B 2bb2.pdb B;235 2bcn.pdb B 2bf8.pdb B;236 2bky.pdb B 2bnk.pdb B;237 2bp3 T 2bw4.pdb B;238 2c12 B 2c42.pdb B;239 2ca1.pdb B 2cb5.pdb B;240 2ch5 B 2cme_4.pdb G;241 2cvz.pdb B 2cxk_2.pdb D;242 2cz1.pdb B 2d3e.pdb B;243 2dp9.pdb B 2dt5.pdb B;244 2e2d.pdb C 2esn.pdb C;245 2eul B 2f06.pdb B;246 2f3d.pdb B 2f5g.pdb B;247 2f69.pdb B 2f7f.pdb B;248 2fbk.pdb B 2fca.pdb B;249 2fex B 2ffs.pdb B;250 2fhq.pdb B 2flu.pdb P;251 2fp4.pdb B 2fr5.pdb B;252 2ftw.pdb B 2fur.pdb B;253 2g40.pdb B 2g63_1.pdb B;254 2g7s.pdb B 2gan.pdb B;255 2gc7 F 2gfa_2.pdb D;256 2gj7.pdb F 2glz.pdb B;257 2gsm B 2gvh.pdb B;258 2h7z.pdb B 2ha2.pdb B;259 2hew.pdb A 2hjs.pdb B;260 2hoe.pdb B 2hrr.pdb B;261 2i4j.pdb B 2iad.pdb B;262 2idj.pdb B 2iim.pdb B;263 2ixc.pdb B 2j0n.pdb B;264 2jhf.pdb B 2msb.pdb B;265 2nlz.pdb B 2ntk.pdb B;266 2o23.pdb B 2ocy.pdb B;267 2otx.pdb B 2p8i_1.pdb B;268 2pva.pdb B 2q41.pdb B;269 2rln.pdb E 2shk.pdb B;270 2tnf.pdb B 3bam.pdb B;271 3grs.pdb B 3ink.pdb D;272 3pfk.pdb B 3ssi.pdb B;273 4sgb.pdb I 5hpg.pdb B;274 12as.pdb B 1a2k_1.pdb B;275 1a12.pdb B 1a6j.pdb B;276 1a73.pdb B 1abb.pdb B;277 1aa7.pdb B 1ail.pdb B;278 1ais.pdb B 1apy.pdb B;279 1aoh.pdb B 1aua.pdb B;280 1aro.pdb L 1avy_3.pdb B;281 1avz.pdb B 1b01.pdb B;282 1ayf.pdb B 1b43.pdb B;283 1b49.pdb C 1b7y.pdb B;284 1b6u.pdb B 1bbp.pdb B;285 1b9c.pdb B 1bg9.pdb B;286 1bgf.pdb B 1bkf.pdb B;287 1bi7.pdb B 1bpl.pdb B;288 1bqp B 1bun.pdb B;289 1btk.pdb B 1byk.pdb B;290 1bw0.pdb B 1c3g.pdb B;291 1c3k.pdb B 1c8b.pdb B;292 1c76.pdb B 1cby.pdb B;293 1cd9.pdb B 1ci6.pdb A;294 1chm.pdb B 1cmc.pdb B;295 1cku.pdb B 1crx.pdb B;296 1cs1.pdb B 1cv8.pdb B;297 1ctt.pdb B 1d0c.pdb B;298 1d0q.pdb B 1d3y.pdb B;299 1d2s.pdb B 1d8l.pdb B;300 1d6r.pdb I 1dcs.pdb B;301 1dd3 B 1dhk.pdb B;302 1dfn.pdb B 1dku.pdb B;303 1dl5.pdb B 1dqe.pdb B;304 1dov.pdb B 1duv.pdb H;305 1dqz.pdb B 1dys.pdb B;306 1dyw.pdb B 1e3m.pdb B;307 1e1z.pdb A 1e6u.pdb B;308 1e7n.pdb B 1ebd.pdb B;309 1eak C 1edz.pdb B;310 1ecm.pdb B 1eg1.pdb C;311 1eg4.pdb P 1eje.pdb B;312 1ei5.pdb B 1emu.pdb B;313 1eo6.pdb B 1ete_2.pdb D;314 1ern.pdb B 1ew6.pdb B;315 1ev7.pdb B 1ez0_1.pdb D;316 1ezg.pdb B 1f1x.pdb B;317 1f0c.pdb B 1f3h.pdb B;318 1f3u F 1f6y.pdb B;319 1f5v.pdb B 1fa2.pdb B;320 1f8e.pdb B 1fe6.pdb B;321 1fe8 J 1flk_2.pdb B;322 1fiu.pdb B 1foc.pdb B;323 1fp1.pdb A 1fs0.pdb G;324 1fr2.pdb B 1fui_2.pdb E;325 1fsg.pdb C 1g0y.pdb I;326 1g1j.pdb B 1g6u.pdb B;327 1g5g E 1g9m.pdb C;328 1gc1 C 1gjo.pdb B;329 1gg6.pdb C 1gl4.pdb B;330 1gka.pdb B 1gpj.pdb B;331 1gpm.pdb B 1gt6.pdb B;332 1gr0.pdb B 1gvf.pdb B;333 1gvn.pdb B 1gxr.pdb B;334 1gxc B 1gz6.pdb B;335 1gyg B 1h1y.pdb B;336 1h21.pdb B 1h5q_3.pdb J;337 1h4p.pdb B 1h80.pdb B;338 1h8g.pdb B 1hcn.pdb B;339 1hbx E 1hfe_2.pdb M;340 1he1 C 1hlg.pdb B;341 1hlo.pdb B 1hsj.pdb B;342 1hqr.pdb B 1hw1.pdb B;343 1hw7.pdb B 1hyu.pdb B;344 1hyl.pdb B 1i2m_2.pdb D;345 1i0r.pdb B 1i4u.pdb B;346 1i52.pdb B 1iag.pdb B;347 1i85.pdb B 1idp.pdb B;348 1ied.pdb B 1ik6.pdb B;349 1ii7.pdb B 1iro.pdb B;350 1iom.pdb B 1ix1.pdb B;351 1ix2.pdb B 1j2j.pdb B;352 1j1b.pdb B 1j5w.pdb B;353 1j7n.pdb B 1jch.pdb B;354 1jay.pdb B 1jfi.pdb B;355 1jdp.pdb B 1ji3.pdb B;356 1ji7.pdb B 1jl4.pdb B;357 1jkj B 1jmv_2.pdb D;358 1joc.pdb B 1jro_2.pdb F;359 1jqp.pdb B 1juv.pdb B;360 1js3.pdb B 1jyl.pdb B;361 1jys.pdb B 1k4z_2.pdb B;362 1k3e.pdb B 1k92.pdb B;363 1k94.pdb B 1kcx.pdb B;364 1kc7.pdb B 1khd_2.pdb C;365 1kfi.pdb B 1kjq.pdb B;366 1kk8.pdb B 1ko7_1.pdb B;367 1knc.pdb B 1ksi.pdb B;368 1ktb.pdb B 1kxv_1.pdb C;369 1kut.pdb B 1l3c.pdb B;370 1l0a.pdb B 1l6x.pdb B;371 1l7d D 1lfd_1.pdb B;372 1ldf.pdb B 1llu_1.pdb B;373 1lm8.pdb C 1lrh_2.pdb C;374 1lq9.pdb B 1lwd.pdb B;375 1ltx.pdb B 1m1f.pdb B;376 1m1l.pdb B 1m4z.pdb B;377 1m3w.pdb B 1m93.pdb B;378 1m9x C 1mhp_1.pdb H;379 1mg1.pdb B 1mk2.pdb B;380 1miw.pdb B 1mnm.pdb B;381 1mo9.pdb B 1mr7_1.pdb B;382 1mpy.pdb B 1mv8_2.pdb D;383 1mvk J 1mze.pdb B;384 1my7.pdb B 1n26.pdb B;385 1n12 D 1n7f.pdb B;386 1n7h.pdb B 1nbq.pdb B;387 1naw.pdb B 1nex_2.pdb D;388 1nf3 C 1nkd.pdb B;389 1ni4.pdb B 1nlt.pdb B;390 1nkt.pdb B 1now_2.pdb B;391 1nox.pdb B 1nsn.pdb H;392 1nrj.pdb B 1nvi.pdb E;393 1nvm B 1ny5.pdb B;394 1nxj B 1o22.pdb B;395 1o0s.pdb B 1o5e.pdb H;396 1o5h.pdb B 1o7n.pdb B;397 1o6z.pdb B 1o9p.pdb B;398 1oa8.pdb B 1ocv.pdb B;399 1obx.pdb C 1ofu.pdb B;400 1oe9.pdb B 1oih.pdb B;401 1oio.pdb B 1olq.pdb B;402 1oki.pdb B 1op3.pdb H;403 1ope.pdb B 1ot6.pdb B;404 1oru.pdb B 1ovn.pdb B;405 1ou0.pdb B 1ozh.pdb B;406 1p0x.pdb B 1p5z.pdb A;407 1p35 B 1p9h.pdb B;408 1p9o.pdb B 1pf5.pdb B;409 1pdo.pdb B 1pix.pdb B;410 1pgr F 1pl8_2.pdb B;411 1pm3.pdb B 1ptq.pdb B;412 1pqw.pdb B 1px8.pdb B;413 1pxv C 1q18.pdb B;414 1q0q.pdb B 1q4u.pdb B;415 1q40 D 1q8r.pdb B;416 1q98.pdb B 1qf8.pdb B;417 1qc7.pdb B 1qiu_2.pdb E;418 1qj8.pdb B 1qm4.pdb B;419 1qks.pdb B 1qoj.pdb B;420 1qmu.pdb B 1qqs.pdb B;421 1qre.pdb B 1qw2.pdb B;422 1qup.pdb B 1qz9.pdb B;423 1qzq.pdb B 1r30.pdb B;424 1r1k.pdb A 1r6t.pdb B;425 1r4m F 1r8o.pdb B;426 1r8s.pdb E 1rew.pdb B;427 1re3 B 1riw.pdb B;428 1rj9.pdb B 1rp3_1.pdb B;429 1rmd.pdb B 1rv1.pdb B;430 1rqi.pdb B 1ryw_2.pdb F;431 1rz1 B 1s3z.pdb B;432 1s1q D 1s7i.pdb B;433 1s7m E 1sc3.pdb B;434 1sbb.pdb B 1sf8_2.pdb H;435 1sdd.pdb B 1sgp.pdb I;436 1sh5.pdb B 1smx.pdb B;437 1skz.pdb B 1sqj.pdb B;438 1sqs.pdb B 1suw.pdb B;439 1stz.pdb B 1sy7.pdb B;440 1svx.pdb B 1t0i.pdb B;441 1t11.pdb B 1t4h.pdb B;442 1t3l.pdb B 1t6s.pdb B;443 1t70 H 1taw.pdb B;444 1t9i.pdb B 1te5.pdb B;445 1tco.pdb B 1thw.pdb B;446 1tj7.pdb B 1to6.pdb B;447 1tm0.pdb B 1tu1.pdb B;448 1tue L 1twi_1.pdb B;449 1tvn.pdb B 1tye_3.pdb F;450 1txg.pdb B 1u0s.pdb A;451 1u19.pdb B 1u7i.pdb B;452 1u6g.pdb B 1uae.pdb B;453 1uaz.pdb B 1ue1.pdb B;454 1udd.pdb B 1uiu.pdb B;455 1ufo B 1uly.pdb B;456 1um0.pdb B 1ur5.pdb C;457 1unn.pdb B 1usu.pdb B;458 1ut7.pdb B 1uvj.pdb B;459 1uuj.pdb B 1uyj_2.pdb B;460 1uwk.pdb B 1v1i.pdb B;461 1v1p.pdb B 1v6t.pdb B;462 1v4v.pdb B 1v7w.pdb B;463 1v8c B 1vcn.pdb B;464 1vbk.pdb B 1vet.pdb B;465 1vdw.pdb B 1vhz.pdb B;466 1vi0.pdb B 1vjp.pdb B;467 1vj2.pdb B 1vkp.pdb B;468 1vl0.pdb B 1vp4.pdb B;469 1vme.pdb B 1vr6.pdb B;470 1vpz.pdb B 1vzy.pdb B;471 1w07.pdb B 1w58.pdb A;472 1w36 F 1w9q_2.pdb S;473 1wa5.pdb B 1wjg.pdb B;474 1who.pdb B 1wp1_1.pdb B;475 1wmh.pdb B 1wu9.pdb B;476 1wud.pdb B 1wws_4.pdb H;477 1ww1.pdb B 1x2i.pdb B;478 1x3m.pdb B 1x9z.pdb B;479 1x8z C 1xdl_4.pdb Z;480 1xcf.pdb B 1xg7.pdb B;481 1xgs.pdb B 1xkz_2.pdb D;482 1xk7 B 1xou.pdb B;483 1xpj.pdb B 1xto.pdb B;484 1xs0 B 1xvs.pdb B;485 1xuv B 1y0u.pdb B;486 1y0z.pdb B 1y6v.pdb B;487 1y4m.pdb B 1y9i.pdb B;488 1yak B 1ycy.pdb B;489 1ycc.pdb B 1ygy.pdb B;490 1yer.pdb B 1ynf_2.pdb D;491 1ynr.pdb B 1ysj.pdb B;492 1yrb.pdb B 1ywq.pdb B;493 1yxb F 1z5y.pdb E;494 1z3e.pdb B 1za3_2.pdb L;495 1z84.pdb B 1ze3.pdb D;496 1zed.pdb B 1zme.pdb D;497 1zke E 1zr4.pdb B;498 1zrs.pdb B 256b.pdb B;499 1zvp D 2a4k.pdb B;500 2a2q.pdb H 2a84.pdb B;501 2a99.pdb B 2ae8_1.pdb B;502 2aca.pdb B 2aio.pdb B;503 2aj7.pdb B 2aua.pdb B;504 2arh.pdb B 2axy_1.pdb B;505 2avp.pdb B 2b0j.pdb B;506 2b0l B 2b7o.pdb B;507 2b5g.pdb B 2bcg.pdb Y;508 2bcn.pdb B 2bif.pdb B;509 2bgw.pdb B 2bo4_1.pdb B;510 2bky.pdb B 2bz6.pdb L;511 2c12 B 2c62.pdb B;512 2c4n.pdb B 2cg6.pdb B;513 2ch5 B 2cro.pdb B;514 2cn4.pdb B 2cy9.pdb B;515 2cvz.pdb B 2d4z.pdb B;516 2d6y.pdb B 2dvt.pdb B;517 2dp9.pdb B 2ess.pdb B;518 2eul B 2f2a.pdb B;519 2f1f.pdb B 2f5v.pdb B;520 2f3d.pdb B 2f8f.pdb B;521 2f96.pdb B 2fd5.pdb B;522 2fbk.pdb B 2fgq.pdb A;523 2fhq.pdb B 2fnj.pdb B;524 2fml.pdb B 2fs2.pdb B;525 2fp4.pdb B 2fzf.pdb B;526 2fzt.pdb B 2g6w.pdb B;527 2g40.pdb B 2gb3_1.pdb B;528 2gc7 F 2gi3.pdb B;529 2gh1.pdb B 2gol_1.pdb B;530 2gj7.pdb F 2gx9.pdb B;531 2gyq.pdb B 2hbv.pdb B;532 2h7z.pdb B 2hmf.pdb B;533 2hoe.pdb B 2hxv.pdb B;534 2hu7.pdb B 2ibg_1.pdb F;535 2i4j.pdb B 2ij9.pdb B;536 2ijd A 2j6b.pdb B;537 2ixc.pdb B 2nac.pdb B;538 2nlz.pdb B 2nyc.pdb B;539 2nul.pdb B 2omy.pdb B;540 2o23.pdb B 2pcd_3.pdb O;541 2pg2.pdb B 2q48.pdb B;542 2pva.pdb B 2tdt.pdb B;543 2tnf.pdb B 3dni.pdb B;544 3cla.pdb B 3lyn.pdb B;545 3grs.pdb B 3ygs.pdb P;546 4ake.pdb B 5rub.pdb B;547 137l.pdb B 1a73.pdb B;548 1a3a D 1acb.pdb I;549 1a78.pdb B 1aip_1.pdb B;550 1aap.pdb B 1aox.pdb B;551 1ajs.pdb B 1aug.pdb B;552 1aox.pdb B 1avz.pdb B;553 1auv.pdb B 1b0n.pdb B;554 1awc.pdb B 1b47.pdb B;555 1ayo.pdb B 1b74.pdb B;556 1b4w.pdb B 1bc2.pdb B;557 1b74.pdb B 1bgf.pdb B;558 1bdf.pdb B 1ble.pdb B;559 1bgp.pdb B 1bpo.pdb B;560 1bih.pdb B 1btn.pdb B;561 1bqu.pdb B 1bys.pdb B;562 1btn.pdb B 1c3k.pdb B;563 1c02.pdb B 1c8u.pdb B;564 1c3r.pdb B 1ccw.pdb B;565 1c7c.pdb B 1cho.pdb I;566 1ce9.pdb B 1cmi_2.pdb D;567 1cho.pdb I 1cs1.pdb B;568 1coi.pdb B 1cvi.pdb B;569 1cs6.pdb B 1d0n.pdb B;570 1cu1.pdb B 1d2z_2.pdb D;571 1d1j.pdb B 1d8u.pdb B;572 1d2z D 1dd3_1.pdb B;573 1dan.pdb H 1dir.pdb B;574 1ddt.pdb B 1dkx.pdb B;575 1dg1.pdb H 1dpg.pdb B;576 1dle.pdb B 1dv1.pdb B;577 1dpg.pdb B 1dyw.pdb B;578 1dxg.pdb B 1e44.pdb B;579 1dza.pdb B 1e7l.pdb B;580 1e20.pdb B 1ear.pdb B;581 1e85.pdb B 1ee8.pdb B;582 1ear.pdb B 1eg4.pdb P;583 1eer.pdb B 1ek9.pdb B;584 1eg5.pdb B 1eny.pdb B;585 1ei6 D 1erv.pdb B;586 1ep3.pdb B 1ewk.pdb B;587 1erv.pdb B 1ezg.pdb B;588 1ex0.pdb B 1f2d_1.pdb B;589 1ezi.pdb B 1f3m.pdb C;590 1f0k.pdb B 1f60.pdb B;591 1f3v.pdb B 1fak.pdb H;592 1f60.pdb B 1fe8_3.pdb J;593 1fbv.pdb C 1flm.pdb B;594 1few.pdb B 1foe_2.pdb D;595 1fjj.pdb B 1fr8.pdb B;596 1fp2.pdb B 1fuj.pdb B;597 1fr8.pdb B 1g1j.pdb B;598 1fx2.pdb B 1g6v.pdb K;599 1g29.pdb 2 1gbn.pdb B;600 1g5h D 1ggx.pdb B;601 1gcj.pdb B 1gmj_1.pdb B;602 1ggx.pdb B 1gpm.pdb B;603 1gmw.pdb B 1gt9.pdb 2;604 1gpq.pdb B 1gvj.pdb B;605 1gr7.pdb B 1gxd_1.pdb C;606 1gvp.pdb B 1gz7.pdb B;607 1gxd C 1h21.pdb B;608 1gzs D 1h65_2.pdb C;609 1h2b.pdb B 1h82_2.pdb C;610 1h4r.pdb B 1hc7_1.pdb B;611 1h8p.pdb B 1hg4_2.pdb E;612 1hc7 B 1hlo.pdb B;613 1hia B 1hsl.pdb B;614 1hlq.pdb B 1hw5.pdb B;615 1hqs.pdb B 1hyn_2.pdb S;616 1hwg.pdb B 1i2p.pdb B;617 1hyn S 1i52.pdb B;618 1i36.pdb B 1iam.pdb B;619 1i58.pdb B 1idr.pdb B;620 1i86.pdb B 1iil_1.pdb E;621 1ig0.pdb B 1irq.pdb B;622 1iil E 1ix2.pdb B;623 1itb.pdb B 1j2r.pdb B;624 1ix9.pdb B 1j79.pdb B;625 1j1d E 1jb7.pdb B;626 1j8b.pdb B 1jfl.pdb B;627 1jb7.pdb B 1ji7.pdb B;628 1jgs.pdb B 1jl5.pdb B;629 1jil.pdb B 1jnr.pdb B;630 1jkm.pdb B 1jr7.pdb B;631 1jof B 1jv1.pdb B;632 1jr7.pdb B 1jys.pdb B;633 1jw9.pdb D 1k51.pdb B;634 1jz7.pdb B 1k93_2.pdb E;635 1k3s.pdb B 1kcf.pdb B;636 1ka2.pdb B 1khh.pdb B;637 1kcf.pdb B 1kk8.pdb B;638 1ki9.pdb B 1koa.pdb B;639 1kke.pdb B 1kso.pdb B;640 1knq.pdb B 1kvd_2.pdb D;641 1ktj.pdb B 1l3i_2.pdb B;642 1kvd D 1l7d_2.pdb D;643 1l4d.pdb B 1lh0.pdb B;644 1l7l.pdb B 1lm5.pdb B;645 1ldj.pdb B 1lql_2.pdb D;646 1lmb.pdb 4 1lwj.pdb B;647 1lql D 1m1l.pdb B;648 1lxe.pdb B 1m56_1.pdb B;649 1m1n B 1m98.pdb B;650 1m3y B 1mh9.pdb B;651 1ma9.pdb B 1mk4.pdb B;652 1mh9.pdb B 1mo9.pdb B;653 1mkk.pdb B 1mr8.pdb B;654 1moe.pdb B 1mvf_1.pdb E;655 1mq8 B 1myl_1.pdb B;656 1mvo.pdb B 1n2a.pdb B;657 1myl B 1n7h.pdb B;658 1n2s.pdb B 1nbw.pdb B;659 1n7s.pdb B 1nez_2.pdb H;660 1nb5 J 1ni5.pdb B;661 1nf9.pdb B 1nm3.pdb B;662 1ni5.pdb B 1nox.pdb B;663 1nms.pdb B 1nsw.pdb B;664 1noy.pdb B 1nvj_3.pdb F;665 1nrv.pdb B 1nxm.pdb B;666 1nvp.pdb B 1o26.pdb B;667 1nxm.pdb B 1o5h.pdb B;668 1o4z D 1o7z.pdb B;669 1o5k.pdb B 1o9y.pdb B;670 1o75.pdb B 1oc0.pdb B;671 1oaa.pdb B 1ofz.pdb B;672 1oc0.pdb B 1oio.pdb B;673 1ogs.pdb B 1ols.pdb B;674 1oj4.pdb B 1op9.pdb B;675 1okj D 1orv.pdb B;676 1oph.pdb B 1ovo_2.pdb B;677 1orv.pdb B 1p0x.pdb B;678 1oxk B 1p65.pdb B;679 1p16 C 1p9i.pdb B;680 1p5b.pdb B 1pe1.pdb B;681 1pbi.pdb B 1pj5.pdb B;682 1pe1.pdb B 1pm3.pdb B;683 1pk8 B 1ptu.pdb B;684 1pm4.pdb B 1pxf.pdb B;685 1pqz.pdb B 1q14.pdb B;686 1py1 B 1q5n.pdb B;687 1q14.pdb B 1q98.pdb B;688 1q6o.pdb B 1qfh.pdb B;689 1q9u.pdb B 1qj4.pdb B;690 1qd1.pdb B 1qkz.pdb H;691 1qja.pdb B 1qou.pdb B;692 1qkz.pdb H 1qre.pdb B;693 1qp1.pdb B 1qwg.pdb B;694 1qrq.pdb B 1qzf_2.pdb D;695 1quq.pdb B 1r1t.pdb B;696 1r0m D 1r6u.pdb B;697 1r1t.pdb B 1r8s.pdb E;698 1r7j.pdb B 1rfy_1.pdb B;699 1r9c.pdb B 1rj8_2.pdb G;700 1rec.pdb B 1rmr.pdb B;701 1rjl.pdb C 1rv3.pdb B;702 1rmr.pdb B 1rz1_1.pdb B;703 1rw6.pdb B 1s4c_2.pdb C;704 1rzh.pdb M 1s7k.pdb B;705 1s28 D 1sbs.pdb L;706 1s7o.pdb B 1sff.pdb B;707 1sbs.pdb L 1sh5.pdb B;708 1sfx.pdb B 1snd.pdb B;709 1sh8.pdb B 1sqk.pdb B;710 1slm.pdb B 1su1.pdb B;711 1squ.pdb B 1syx_1.pdb B;712 1su1.pdb B 1t11.pdb B;713 1sz6.pdb B 1t56.pdb B;714 1t15.pdb B 1t6t.pdb 2;715 1t3m.pdb B 1ta3.pdb B;716 1t7r.pdb B 1ted_1.pdb B;717 1ta3.pdb B 1tj7.pdb B;718 1tfe.pdb B 1to9.pdb B;719 1tjc.pdb B 1tu3_1.pdb B;720 1tmc.pdb B 1tvx.pdb B;721 1tuh.pdb B 1tyx.pdb B;722 1tvx.pdb B 1u19.pdb B;723 1tzb.pdb B 1u7n.pdb B;724 1u1i.pdb B 1ual.pdb B;725 1u6l.pdb B 1udr.pdb B;726 1ub0.pdb B 1uix.pdb B;727 1udr.pdb B 1um0.pdb B;728 1uk8.pdb B 1urh.pdb B;729 1ump C 1usx.pdb B;730 1up7 B 1uur.pdb B;731 1ut8.pdb B 1uyt_1.pdb B;732 1uur.pdb B 1v1p.pdb B;733 1uzm.pdb B 1v6z.pdb B;734 1v25.pdb B 1v84.pdb B;735 1v58 B 1vbo_1.pdb A;736 1v8d.pdb B 1vf6_1.pdb C;737 1vbo A 1vi0.pdb B;738 1vgy.pdb B 1vjq.pdb B;739 1vi2.pdb B 1vky.pdb B;740 1vjg.pdb B 1vmg.pdb B;741 1vl4.pdb B 1vr7.pdb B;742 1vmg.pdb B 1w07.pdb B;743 1vrw.pdb B 1w5r.pdb B;744 1w0m B 1w9y.pdb B;745 1w3b.pdb B 1wht.pdb B;746 1wbh.pdb B 1wpn.pdb B;747 1wht.pdb B 1wud.pdb B;748 1wqw.pdb B 1www.pdb W;749 1wui.pdb L 1x2t_1.pdb B;750 1ww9.pdb B 1x92.pdb B;751 1x3z.pdb B 1xdp.pdb B;752 1x92.pdb B 1xgs.pdb B;753 1xe7 C 1xl3_2.pdb D;754 1xhk.pdb B 1xpc.pdb B;755 1xka.pdb C 1xs1_1.pdb B;756 1xpm B 1xw8.pdb B;757 1xs1 B 1y0z.pdb B;758 1xx6.pdb B 1y6x.pdb B;759 1y14.pdb B 1y9w.pdb B;760 1y5h.pdb B 1ycg_1.pdb B;761 1yav.pdb B 1yhc.pdb B;762 1ycg B 1ynr.pdb B;763 1ykh.pdb B 1ysr.pdb B;764 1yoa.pdb B 1yx1.pdb B;765 1yre D 1z41.pdb B;766 1yz3.pdb B 1zai.pdb B;767 1z41.pdb B 1zed.pdb B;768 1zbr.pdb B 1zmt.pdb B;769 1zee.pdb B 1zro.pdb B;770 1zkp C 1zww.pdb B;771 1zru.pdb B 2a4x.pdb B;772 1zww.pdb B 2a99.pdb B;773 2a5z.pdb B 2aeb_2.pdb B;774 2a9k.pdb B 2air.pdb B;775 2adf.pdb H 2ark_2.pdb B;776 2ajf E 2ayl.pdb B;777 2ark B 2b0l_2.pdb B;778 2ayu.pdb B 2b82.pdb B;779 2b1y.pdb B 2bcj.pdb B;780 2b5i.pdb B 2bgx.pdb B;781 2bde.pdb B 2bo9.pdb B;782 2bgx.pdb B 2c12_1.pdb B;783 2bpl C 2c7n_4.pdb H;784 2c1n C 2ch4_1.pdb W;785 2c4v.pdb B 2cnz.pdb B;786 2ch7.pdb B 2cye_2.pdb D;787 2cnz.pdb B 2d6y.pdb B;788 2cz4.pdb B 2dw6_1.pdb B;789 2d7c D 2euc.pdb B;790 2dpi.pdb D 2f1k_1.pdb B;791 2evr.pdb B 2f62.pdb B;792 2f1k B 2f96.pdb B;793 2f6k.pdb B 2fdo.pdb A;794 2f9h.pdb B 2fh5.pdb B;795 2fbl.pdb B 2fmt.pdb B;796 2fhz.pdb B 2fsw.pdb B;797 2fmt.pdb B 2fzt.pdb B;798 2ftx.pdb B 2g7g.pdb B;799 2g0t.pdb B 2gbo.pdb B;800 2g50 F 2ghp_2.pdb F;801 2gci B 2gqv.pdb B;802 2ghp F 2gyq.pdb B;803 2gtd F 2hcb.pdb B;804 2gys.pdb B 2hmp.pdb B;805 2h9d D 2hwn_2.pdb D;806 2hox B 2icy_1.pdb B;807 2hwn D 2ijd_1.pdb A;808 2ido D 2j7p.pdb B;809 2ilk.pdb B 2nlv.pdb B;810 2ixp D 2nvn.pdb B;811 2nml.pdb B 2oq1.pdb B;812 2nvn.pdb B 2pg2.pdb B;813 2oza.pdb B 2q4g.pdb W;814 2pgd.pdb B 2tgi.pdb B;815 2q3m.pdb B 3cro.pdb R;816 2toh.pdb B 3mag.pdb B;817 3cro.pdb R 4ake.pdb B;818 3pnp.pdb B 6ldh.pdb B;819 1a12.pdb B 1a3q.pdb B;820 1a2z.pdb B 1a8y.pdb B;821 1aa7.pdb B 1agq.pdb B;822 1adu.pdb B 1am7.pdb B;823 1aoh.pdb B 1at0.pdb B;824 1aro.pdb L 1avf_2.pdb J;825 1auo.pdb B 1ay2.pdb B;826 1ayf.pdb B 1b2p.pdb B;827 1b1z.pdb B 1b63.pdb B;828 1b6u.pdb B 1b9h.pdb B;829 1b9c.pdb B 1bec.pdb B;830 1bdb.pdb B 1bhh.pdb B;831 1bi7.pdb B 1bmt.pdb B;832 1bml.pdb B 1bsl.pdb B;833 1btk.pdb B 1bxg.pdb B;834 1bw0.pdb B 1c0p.pdb B;835 1bzq N 1c5e.pdb B;836 1c76.pdb B 1cbk.pdb B;837 1c9o.pdb B 1cfm.pdb B;838 1chm.pdb B 1cl8.pdb D;839 1cku.pdb B 1cpb.pdb B;840 1cnz.pdb B 1ct9_2.pdb C;841 1ctt.pdb B 1cy9.pdb B;842 1cxp.pdb C 1d2e.pdb B;843 1d2s.pdb B 1d7u.pdb B;844 1d6r.pdb I 1dbt_1.pdb B;845 1d9c.pdb B 1df4.pdb B;846 1dfn.pdb B 1djt.pdb B;847 1dj7.pdb B 1dn1.pdb B;848 1dov.pdb B 1ds6.pdb B;849 1dqz.pdb B 1dxy.pdb B;850 1dvp.pdb B 1e19.pdb B;851 1e1z.pdb A 1e5r.pdb B;852 1e50 A 1e9g.pdb B;853 1eak C 1ecy.pdb B;854 1ecm.pdb B 1ef8.pdb B;855 1eeq.pdb B 1egw_2.pdb D;856 1ei5.pdb B 1elu.pdb B;857 1eku.pdb B 1ept.pdb A;858 1ern.pdb B 1evl_2.pdb D;859 1ev7.pdb B 1ext.pdb B;860 1ewy C 1f02.pdb T;861 1f0c.pdb B 1f38.pdb B;862 1f2u.pdb B 1f51_2.pdb D;863 1f5v.pdb B 1f8m.pdb B;864 1f8e.pdb B 1fcd_1.pdb C;865 1fbt.pdb B 1fgu.pdb B;866 1fiu.pdb B 1fno.pdb B;867 1fm0.pdb E 1fqj_1.pdb B;868 1fr2.pdb B 1fsy.pdb B;869 1fsg.pdb C 1fxz.pdb B;870 1fwx D 1g4u.pdb R;871 1g5g E 1g8q.pdb B;872 1g8k F 1ge7.pdb B;873 1gg6.pdb C 1gkm.pdb B;874 1gka.pdb B 1goi.pdb B;875 1gmv.pdb B 1gqn.pdb B;876 1gr0.pdb B 1gu7.pdb B;877 1gtw.pdb B 1gwn.pdb C;878 1gxc B 1gyy.pdb B;879 1gyg B 1h16.pdb B;880 1gzj.pdb B 1h3o_1.pdb B;881 1h4p.pdb B 1h72.pdb A;882 1h6o.pdb B 1h9r.pdb B;883 1hbx E 1he8.pdb B;884 1he1 C 1hk7.pdb B;885 1hh2.pdb A 1hnj.pdb B;886 1hqr.pdb B 1huw.pdb B;887 1htr.pdb P 1hxp.pdb B;888 1hyl.pdb B 1i1q.pdb B;889 1i0r.pdb B 1i49.pdb B;890 1i31.pdb P 1i7b.pdb A;891 1i85.pdb B 1icf_2.pdb D;892 1ibr D 1ihb.pdb B;893 1ii7.pdb B 1iq8.pdb B;894 1iom.pdb B 1itv.pdb B;895 1isy.pdb B 1izm.pdb B;896 1j1b.pdb B 1j3w.pdb B;897 1j3b.pdb B 1ja3.pdb B;898 1jay.pdb B 1je5.pdb B;899 1jdp.pdb B 1jhd.pdb B;900 1jg8.pdb B 1jk9_2.pdb D;901 1jkj B 1jm6.pdb B;902 1jlv B 1jpy_1.pdb B;903 1jqp.pdb B 1jsu.pdb B;904 1js3.pdb B 1jxp.pdb B;905 1jvm.pdb B 1k1e_3.pdb J;906 1k3e.pdb B 1k6f_2.pdb E;907 1k5n.pdb B 1kam.pdb B;908 1kc7.pdb B 1kg0.pdb B;909 1kfi.pdb B 1kix.pdb C;910 1ki1 D 1klo.pdb B;911 1knc.pdb B 1kpt.pdb B;912 1kor.pdb B 1ku2.pdb B;913 1kut.pdb B 1l0w.pdb B;914 1l0a.pdb B 1l5r.pdb B;915 1l3p.pdb B 1lc5.pdb B;916 1ldf.pdb B 1lkt_1.pdb B;917 1lj9.pdb B 1lom.pdb B;918 1lq9.pdb B 1luc.pdb B;919 1ltx.pdb B 1lyw_2.pdb D;920 1lxd.pdb B 1m2v.pdb B;921 1m3w.pdb B 1m6p.pdb B;922 1m6d.pdb B 1mcw.pdb M;923 1mg1.pdb B 1mjh.pdb B;924 1miw.pdb B 1ml0.pdb D;925 1mkf.pdb B 1mp4.pdb B;926 1mpy.pdb B 1mu4.pdb B;927 1msc.pdb B 1mxb.pdb B;928 1my7.pdb B 1n1c.pdb B;929 1n12 D 1n55.pdb B;930 1n2m E 1n9p.pdb B;931 1naw.pdb B 1ne2.pdb B;932 1nco.pdb B 1nh2.pdb B;933 1ni4.pdb B 1nl0.pdb H;934 1nkt.pdb B 1nn5.pdb B;935 1nme.pdb B 1nqk.pdb B;936 1nrj.pdb B 1nu9_2.pdb F;937 1nt2.pdb B 1nw9.pdb B;938 1nxj B 1o0w.pdb B;939 1o0s.pdb B 1o54.pdb B;940 1o4w.pdb B 1o6e.pdb B;941 1o6z.pdb B 1o94_1.pdb B;942 1o89.pdb B 1ob9.pdb B;943 1obx.pdb C 1oez.pdb X;944 1oe9.pdb B 1oi0.pdb B;945 1og6.pdb B 1ok7.pdb B;946 1oki.pdb B 1on2.pdb B;947 1omw.pdb B 1or4.pdb B;948 1oru.pdb B 1ov3.pdb A;949 1ou0.pdb B 1oyn.pdb B;950 1ox0.pdb B 1p2j.pdb I;951 1p35 B 1p7q.pdb D;952 1p74.pdb B 1pcs.pdb B;953 1pdo.pdb B 1phz.pdb B;954 1pgr F 1pkq_1.pdb B;955 1pk1 B 1ppr.pdb N;956 1pqw.pdb B 1pvg.pdb B;957 1pv1.pdb B 1pzs.pdb B;958 1q0q.pdb B 1q47.pdb B;959 1q40 D 1q7e.pdb B;960 1q67.pdb B 1qb2.pdb B;961 1qc7.pdb B 1qhd.pdb B;962 1qh3.pdb B 1qki_1.pdb B;963 1qks.pdb B 1qo0.pdb B;964 1qmu.pdb B 1qpo_2.pdb D;965 1qoz.pdb B 1qtn.pdb B;966 1qup.pdb B 1qyc.pdb B;967 1qx4.pdb B 1r11.pdb B;968 1r1k.pdb A 1r59.pdb X;969 1r4m F 1r8d.pdb B;970 1r7a.pdb B 1rcu.pdb B;971 1re3 B 1rh5.pdb B;972 1rgf.pdb B 1rke.pdb B;973 1rmd.pdb B 1rrp_1.pdb B;974 1rqi.pdb B 1ry9_1.pdb B;975 1rw0.pdb B 1s0p.pdb B;976 1s1q D 1s6v_1.pdb B;977 1s5a.pdb B 1s98.pdb B;978 1sbb.pdb B 1se8.pdb B;979 1sdd.pdb B 1sg4.pdb B;980 1sfp.pdb B 1sjw.pdb B;981 1skz.pdb B 1sq2.pdb N;982 1sox.pdb B 1srq_2.pdb D;983 1stz.pdb B 1sww.pdb B;984 1svx.pdb B 1t06.pdb B;985 1sz2.pdb B 1t3c.pdb B;986 1t3l.pdb B 1t6f.pdb B;987 1t5o D 1t8t.pdb B;988 1t9i.pdb B 1tdj.pdb B;989 1tco.pdb B 1th8.pdb B;990 1tf0.pdb B 1tlk.pdb B;991 1tm0.pdb B 1trr_1.pdb B;992 1tqb.pdb B 1tuw.pdb B;993 1tvn.pdb B 1txq.pdb B;994 1txg.pdb B 1u00.pdb P;995 1tz9.pdb B 1u5x.pdb B;996 1u6g.pdb B 1u9t.pdb B;997 1u8s.pdb B 1uc2.pdb B;998 1udd.pdb B 1ugh.pdb I;999 1ufo B 1uku.pdb B;1000 1ujq.pdb B 1unh.pdb B;

List of monomer pairs

3two.ent B 5mdh.ent A;2 3n9s.ent A 5mdh.ent A;3 3n9s.ent A 3two.ent B;4 3exe.ent B 5mdh.ent A;5 3exe.ent B 3two.ent B;6 3exe.ent B 3n9s.ent A;7 2yy7.ent A 5mdh.ent A;8 2yy7.ent A 3two.ent B;9 2yy7.ent A 3n9s.ent A;10 2yy7.ent A 3exe.ent B;11 2qx3.ent A 5mdh.ent A;12 2qx3.ent A 3two.ent B;13 2qx3.ent A 3n9s.ent A;14 2qx3.ent A 3exe.ent B;15 2qx3.ent A 2yy7.ent A;16 2hk2.ent A 5mdh.ent A;17 2hk2.ent A 3two.ent B;18 2hk2.ent A 3n9s.ent A;19 2hk2.ent A 3exe.ent B;20 2hk2.ent A 2yy7.ent A;21 2hk2.ent A 2qx3.ent A;22 2eih.ent A 5mdh.ent A;23 2eih.ent A 3two.ent B;24 2eih.ent A 3n9s.ent A;25 2eih.ent A 3exe.ent B;26 2eih.ent A 2yy7.ent A;27 2eih.ent A 2qx3.ent A;28 2eih.ent A 2hk2.ent A;29 1yg9.ent A 5mdh.ent A;30 1yg9.ent A 3two.ent B;31 1yg9.ent A 3n9s.ent A;32 1yg9.ent A 3exe.ent B;33 1yg9.ent A 2yy7.ent A;34 1yg9.ent A 2qx3.ent A;35 1yg9.ent A 2hk2.ent A;36 1yg9.ent A 2eih.ent A;37 1tca.ent A 5mdh.ent A;38 1tca.ent A 3two.ent B;39 1tca.ent A 3n9s.ent A;40 1tca.ent A 3exe.ent B;41 1tca.ent A 2yy7.ent A;42 1tca.ent A 2qx3.ent A;43 1tca.ent A 2hk2.ent A;44 1tca.ent A 2eih.ent A;45 1tca.ent A 1yg9.ent A;46 1pxz.ent A 5mdh.ent A;47 1pxz.ent A 3two.ent B;48 1pxz.ent A 3n9s.ent A;49 1pxz.ent A 3exe.ent B;50 1pxz.ent A 2yy7.ent A;51 1pxz.ent A 2qx3.ent A;52 1pxz.ent A 2hk2.ent A;53 1pxz.ent A 2eih.ent A;54 1pxz.ent A 1yg9.ent A;55 1pxz.ent A 1tca.ent A;56 1llp.ent A 5mdh.ent A;57 1llp.ent A 3two.ent B;58 1llp.ent A 3n9s.ent A;59 1llp.ent A 3exe.ent B;60 1llp.ent A 2yy7.ent A;61 1llp.ent A 2qx3.ent A;62 1llp.ent A 2hk2.ent A;63 1llp.ent A 2eih.ent A;64 1llp.ent A 1yg9.ent A;65 1llp.ent A 1tca.ent A;66 1llp.ent A 1pxz.ent A;67 1h6u.ent A 5mdh.ent A;68 1h6u.ent A 3two.ent B;69 1h6u.ent A 3n9s.ent A;70 1h6u.ent A 3exe.ent B;71 1h6u.ent A 2yy7.ent A;72 1h6u.ent A 2qx3.ent A;73 1h6u.ent A 2hk2.ent A;74 1h6u.ent A 2eih.ent A;75 1h6u.ent A 1yg9.ent A;76 1h6u.ent A 1tca.ent A;77 1h6u.ent A 1pxz.ent A;78 1h6u.ent A 1llp.ent A;79 1ea7.ent A 5mdh.ent A;80 1ea7.ent A 3two.ent B;81 1ea7.ent A 3n9s.ent A;82 1ea7.ent A 3exe.ent B;83 1ea7.ent A 2yy7.ent A;84 1ea7.ent A 2qx3.ent A;85 1ea7.ent A 2hk2.ent A;86 1ea7.ent A 2eih.ent A;87 1ea7.ent A 1yg9.ent A;88 1ea7.ent A 1tca.ent A;89 1ea7.ent A 1pxz.ent A;90 1ea7.ent A 1llp.ent A;91 1ea7.ent A 1h6u.ent A;92 1a4m.ent A 5mdh.ent A;93 1a4m.ent A 3two.ent B;94 1a4m.ent A 3n9s.ent A;95 1a4m.ent A 3exe.ent B;96 1a4m.ent A 2yy7.ent A;97 1a4m.ent A 2qx3.ent A;98 1a4m.ent A 2hk2.ent A;99 1a4m.ent A 2eih.ent A;100 1a4m.ent A 1yg9.ent A;101 1a4m.ent A 1tca.ent A;102 1a4m.ent A 1pxz.ent A;103 1a4m.ent A 1llp.ent A;104 1a4m.ent A 1h6u.ent A;105 1a4m.ent A 1ea7.ent A;106 4b5w.ent A 5mdh.ent A;107 4b5w.ent A 3two.ent B;108 4b5w.ent A 3n9s.ent A;109 4b5w.ent A 3exe.ent B;110 4b5w.ent A 2yy7.ent A;111 4b5w.ent A 2qx3.ent A;112 4b5w.ent A 2hk2.ent A;113 4b5w.ent A 2eih.ent A;114 4b5w.ent A 1yg9.ent A;115 4b5w.ent A 1tca.ent A;116 4b5w.ent A 1pxz.ent A;117 4b5w.ent A 1llp.ent A;118 4b5w.ent A 1h6u.ent A;119 4b5w.ent A 1ea7.ent A;120 4b5w.ent A 1a4m.ent A;121 3q1n.ent A 5mdh.ent A;122 3q1n.ent A 3two.ent B;123 3q1n.ent A 3n9s.ent A;124 3q1n.ent A 3exe.ent B;125 3q1n.ent A 2yy7.ent A;126 3q1n.ent A 2qx3.ent A;127 3q1n.ent A 2hk2.ent A;128 3q1n.ent A 2eih.ent A;129 3q1n.ent A 1yg9.ent A;130 3q1n.ent A 1tca.ent A;131 3q1n.ent A 1pxz.ent A;132 3q1n.ent A 1llp.ent A;133 3q1n.ent A 1h6u.ent A;134 3q1n.ent A 1ea7.ent A;135 3q1n.ent A 1a4m.ent A;136 3q1n.ent A 4b5w.ent A;137 3m66.ent A 5mdh.ent A;138 3m66.ent A 3two.ent B;139 3m66.ent A 3n9s.ent A;140 3m66.ent A 3exe.ent B;141 3m66.ent A 2yy7.ent A;142 3m66.ent A 2qx3.ent A;143 3m66.ent A 2hk2.ent A;144 3m66.ent A 2eih.ent A;145 3m66.ent A 1yg9.ent A;146 3m66.ent A 1tca.ent A;147 3m66.ent A 1pxz.ent A;148 3m66.ent A 1llp.ent A;149 3m66.ent A 1h6u.ent A;150 3m66.ent A 1ea7.ent A;151 3m66.ent A 1a4m.ent A;152 3m66.ent A 4b5w.ent A;153 3m66.ent A 3q1n.ent A;154 3gae.ent A 5mdh.ent A;155 3gae.ent A 3two.ent B;156 3gae.ent A 3n9s.ent A;157 3gae.ent A 3exe.ent B;158 3gae.ent A 2yy7.ent A;159 3gae.ent A 2qx3.ent A;160 3gae.ent A 2hk2.ent A;161 3gae.ent A 2eih.ent A;162 3gae.ent A 1yg9.ent A;163 3gae.ent A 1tca.ent A;164 3gae.ent A 1pxz.ent A;165 3gae.ent A 1llp.ent A;166 3gae.ent A 1h6u.ent A;167 3gae.ent A 1ea7.ent A;168 3gae.ent A 1a4m.ent A;169 3gae.ent A 4b5w.ent A;170 3gae.ent A 3q1n.ent A;171 3gae.ent A 3m66.ent A;172 3bcz.ent A 5mdh.ent A;173 3bcz.ent A 3two.ent B;174 3bcz.ent A 3n9s.ent A;175 3bcz.ent A 3exe.ent B;176 3bcz.ent A 2yy7.ent A;177 3bcz.ent A 2qx3.ent A;178 3bcz.ent A 2hk2.ent A;179 3bcz.ent A 2eih.ent A;180 3bcz.ent A 1yg9.ent A;181 3bcz.ent A 1tca.ent A;182 3bcz.ent A 1pxz.ent A;183 3bcz.ent A 1llp.ent A;184 3bcz.ent A 1h6u.ent A;185 3bcz.ent A 1ea7.ent A;186 3bcz.ent A 1a4m.ent A;187 3bcz.ent A 4b5w.ent A;188 3bcz.ent A 3q1n.ent A;189 3bcz.ent A 3m66.ent A;190 3bcz.ent A 3gae.ent A;191 2voa.ent A 5mdh.ent A;192 2voa.ent A 3two.ent B;193 2voa.ent A 3n9s.ent A;194 2voa.ent A 3exe.ent B;195 2voa.ent A 2yy7.ent A;196 2voa.ent A 2qx3.ent A;197 2voa.ent A 2hk2.ent A;198 2voa.ent A 2eih.ent A;199 2voa.ent A 1yg9.ent A;200 2voa.ent A 1tca.ent A;201 2voa.ent A 1pxz.ent A;202 2voa.ent A 1llp.ent A;203 2voa.ent A 1h6u.ent A;204 2voa.ent A 1ea7.ent A;205 2voa.ent A 1a4m.ent A;206 2voa.ent A 4b5w.ent A;207 2voa.ent A 3q1n.ent A;208 2voa.ent A 3m66.ent A;209 2voa.ent A 3gae.ent A;210 2voa.ent A 3bcz.ent A;211 2p9h.ent A 5mdh.ent A;212 2p9h.ent A 3two.ent B;213 2p9h.ent A 3n9s.ent A;214 2p9h.ent A 3exe.ent B;215 2p9h.ent A 2yy7.ent A;216 2p9h.ent A 2qx3.ent A;217 2p9h.ent A 2hk2.ent A;218 2p9h.ent A 2eih.ent A;219 2p9h.ent A 1yg9.ent A;220 2p9h.ent A 1tca.ent A;221 2p9h.ent A 1pxz.ent A;222 2p9h.ent A 1llp.ent A;223 2p9h.ent A 1h6u.ent A;224 2p9h.ent A 1ea7.ent A;225 2p9h.ent A 1a4m.ent A;226 2p9h.ent A 4b5w.ent A;227 2p9h.ent A 3q1n.ent A;228 2p9h.ent A 3m66.ent A;229 2p9h.ent A 3gae.ent A;230 2p9h.ent A 3bcz.ent A;231 2p9h.ent A 2voa.ent A;232 2gnu.ent M 5mdh.ent A;233 2gnu.ent M 3two.ent B;234 2gnu.ent M 3n9s.ent A;235 2gnu.ent M 3exe.ent B;236 2gnu.ent M 2yy7.ent A;237 2gnu.ent M 2qx3.ent A;238 2gnu.ent M 2hk2.ent A;239 2gnu.ent M 2eih.ent A;240 2gnu.ent M 1yg9.ent A;241 2gnu.ent M 1tca.ent A;242 2gnu.ent M 1pxz.ent A;243 2gnu.ent M 1llp.ent A;244 2gnu.ent M 1h6u.ent A;245 2gnu.ent M 1ea7.ent A;246 2gnu.ent M 1a4m.ent A;247 2gnu.ent M 4b5w.ent A;248 2gnu.ent M 3q1n.ent A;249 2gnu.ent M 3m66.ent A;250 2gnu.ent M 3gae.ent A;251 2gnu.ent M 3bcz.ent A;252 2gnu.ent M 2voa.ent A;253 2gnu.ent M 2p9h.ent A;254 2ci1.ent A 5mdh.ent A;255 2ci1.ent A 3two.ent B;256 2ci1.ent A 3n9s.ent A;257 2ci1.ent A 3exe.ent B;258 2ci1.ent A 2yy7.ent A;259 2ci1.ent A 2qx3.ent A;260 2ci1.ent A 2hk2.ent A;261 2ci1.ent A 2eih.ent A;262 2ci1.ent A 1yg9.ent A;263 2ci1.ent A 1tca.ent A;264 2ci1.ent A 1pxz.ent A;265 2ci1.ent A 1llp.ent A;266 2ci1.ent A 1h6u.ent A;267 2ci1.ent A 1ea7.ent A;268 2ci1.ent A 1a4m.ent A;269 2ci1.ent A 4b5w.ent A;270 2ci1.ent A 3q1n.ent A;271 2ci1.ent A 3m66.ent A;272 2ci1.ent A 3gae.ent A;273 2ci1.ent A 3bcz.ent A;274 2ci1.ent A 2voa.ent A;275 2ci1.ent A 2p9h.ent A;276 2ci1.ent A 2gnu.ent M;277 1xq6.ent A 5mdh.ent A;278 1xq6.ent A 3two.ent B;279 1xq6.ent A 3n9s.ent A;280 1xq6.ent A 3exe.ent B;281 1xq6.ent A 2yy7.ent A;282 1xq6.ent A 2qx3.ent A;283 1xq6.ent A 2hk2.ent A;284 1xq6.ent A 2eih.ent A;285 1xq6.ent A 1yg9.ent A;286 1xq6.ent A 1tca.ent A;287 1xq6.ent A 1pxz.ent A;288 1xq6.ent A 1llp.ent A;289 1xq6.ent A 1h6u.ent A;290 1xq6.ent A 1ea7.ent A;291 1xq6.ent A 1a4m.ent A;292 1xq6.ent A 4b5w.ent A;293 1xq6.ent A 3q1n.ent A;294 1xq6.ent A 3m66.ent A;295 1xq6.ent A 3gae.ent A;296 1xq6.ent A 3bcz.ent A;297 1xq6.ent A 2voa.ent A;298 1xq6.ent A 2p9h.ent A;299 1xq6.ent A 2gnu.ent M;300 1xq6.ent A 2ci1.ent A;301 1tml.ent A 5mdh.ent A;302 1tml.ent A 3two.ent B;303 1tml.ent A 3n9s.ent A;304 1tml.ent A 3exe.ent B;305 1tml.ent A 2yy7.ent A;306 1tml.ent A 2qx3.ent A;307 1tml.ent A 2hk2.ent A;308 1tml.ent A 2eih.ent A;309 1tml.ent A 1yg9.ent A;310 1tml.ent A 1tca.ent A;311 1tml.ent A 1pxz.ent A;312 1tml.ent A 1llp.ent A;313 1tml.ent A 1h6u.ent A;314 1tml.ent A 1ea7.ent A;315 1tml.ent A 1a4m.ent A;316 1tml.ent A 4b5w.ent A;317 1tml.ent A 3q1n.ent A;318 1tml.ent A 3m66.ent A;319 1tml.ent A 3gae.ent A;320 1tml.ent A 3bcz.ent A;321 1tml.ent A 2voa.ent A;322 1tml.ent A 2p9h.ent A;323 1tml.ent A 2gnu.ent M;324 1tml.ent A 2ci1.ent A;325 1tml.ent A 1xq6.ent A;326 1qgj.ent A 5mdh.ent A;327 1qgj.ent A 3two.ent B;328 1qgj.ent A 3n9s.ent A;329 1qgj.ent A 3exe.ent B;330 1qgj.ent A 2yy7.ent A;331 1qgj.ent A 2qx3.ent A;332 1qgj.ent A 2hk2.ent A;333 1qgj.ent A 2eih.ent A;334 1qgj.ent A 1yg9.ent A;335 1qgj.ent A 1tca.ent A;336 1qgj.ent A 1pxz.ent A;337 1qgj.ent A 1llp.ent A;338 1qgj.ent A 1h6u.ent A;339 1qgj.ent A 1ea7.ent A;340 1qgj.ent A 1a4m.ent A;341 1qgj.ent A 4b5w.ent A;342 1qgj.ent A 3q1n.ent A;343 1qgj.ent A 3m66.ent A;344 1qgj.ent A 3gae.ent A;345 1qgj.ent A 3bcz.ent A;346 1qgj.ent A 2voa.ent A;347 1qgj.ent A 2p9h.ent A;348 1qgj.ent A 2gnu.ent M;349 1qgj.ent A 2ci1.ent A;350 1qgj.ent A 1xq6.ent A;351 1qgj.ent A 1tml.ent A;352 1nas.ent A 5mdh.ent A;353 1nas.ent A 3two.ent B;354 1nas.ent A 3n9s.ent A;355 1nas.ent A 3exe.ent B;356 1nas.ent A 2yy7.ent A;357 1nas.ent A 2qx3.ent A;358 1nas.ent A 2hk2.ent A;359 1nas.ent A 2eih.ent A;360 1nas.ent A 1yg9.ent A;361 1nas.ent A 1tca.ent A;362 1nas.ent A 1pxz.ent A;363 1nas.ent A 1llp.ent A;364 1nas.ent A 1h6u.ent A;365 1nas.ent A 1ea7.ent A;366 1nas.ent A 1a4m.ent A;367 1nas.ent A 4b5w.ent A;368 1nas.ent A 3q1n.ent A;369 1nas.ent A 3m66.ent A;370 1nas.ent A 3gae.ent A;371 1nas.ent A 3bcz.ent A;372 1nas.ent A 2voa.ent A;373 1nas.ent A 2p9h.ent A;374 1nas.ent A 2gnu.ent M;375 1nas.ent A 2ci1.ent A;376 1nas.ent A 1xq6.ent A;377 1nas.ent A 1tml.ent A;378 1nas.ent A 1qgj.ent A;379 1hvq.ent A 5mdh.ent A;380 1hvq.ent A 3two.ent B;381 1hvq.ent A 3n9s.ent A;382 1hvq.ent A 3exe.ent B;383 1hvq.ent A 2yy7.ent A;384 1hvq.ent A 2qx3.ent A;385 1hvq.ent A 2hk2.ent A;386 1hvq.ent A 2eih.ent A;387 1hvq.ent A 1yg9.ent A;388 1hvq.ent A 1tca.ent A;389 1hvq.ent A 1pxz.ent A;390 1hvq.ent A 1llp.ent A;391 1hvq.ent A 1h6u.ent A;392 1hvq.ent A 1ea7.ent A;393 1hvq.ent A 1a4m.ent A;394 1hvq.ent A 4b5w.ent A;395 1hvq.ent A 3q1n.ent A;396 1hvq.ent A 3m66.ent A;397 1hvq.ent A 3gae.ent A;398 1hvq.ent A 3bcz.ent A;399 1hvq.ent A 2voa.ent A;400 1hvq.ent A 2p9h.ent A;401 1hvq.ent A 2gnu.ent M;402 1hvq.ent A 2ci1.ent A;403 1hvq.ent A 1xq6.ent A;404 1hvq.ent A 1tml.ent A;405 1hvq.ent A 1qgj.ent A;406 1hvq.ent A 1nas.ent A;407 1fts.ent A 5mdh.ent A;408 1fts.ent A 3two.ent B;409 1fts.ent A 3n9s.ent A;410 1fts.ent A 3exe.ent B;411 1fts.ent A 2yy7.ent A;412 1fts.ent A 2qx3.ent A;413 1fts.ent A 2hk2.ent A;414 1fts.ent A 2eih.ent A;415 1fts.ent A 1yg9.ent A;416 1fts.ent A 1tca.ent A;417 1fts.ent A 1pxz.ent A;418 1fts.ent A 1llp.ent A;419 1fts.ent A 1h6u.ent A;420 1fts.ent A 1ea7.ent A;421 1fts.ent A 1a4m.ent A;422 1fts.ent A 4b5w.ent A;423 1fts.ent A 3q1n.ent A;424 1fts.ent A 3m66.ent A;425 1fts.ent A 3gae.ent A;426 1fts.ent A 3bcz.ent A;427 1fts.ent A 2voa.ent A;428 1fts.ent A 2p9h.ent A;429 1fts.ent A 2gnu.ent M;430 1fts.ent A 2ci1.ent A;431 1fts.ent A 1xq6.ent A;432 1fts.ent A 1tml.ent A;433 1fts.ent A 1qgj.ent A;434 1fts.ent A 1nas.ent A;435 1fts.ent A 1hvq.ent A;436 1dea.ent A 5mdh.ent A;437 1dea.ent A 3two.ent B;438 1dea.ent A 3n9s.ent A;439 1dea.ent A 3exe.ent B;440 1dea.ent A 2yy7.ent A;441 1dea.ent A 2qx3.ent A;442 1dea.ent A 2hk2.ent A;443 1dea.ent A 2eih.ent A;444 1dea.ent A 1yg9.ent A;445 1dea.ent A 1tca.ent A;446 1dea.ent A 1pxz.ent A;447 1dea.ent A 1llp.ent A;448 1dea.ent A 1h6u.ent A;449 1dea.ent A 1ea7.ent A;450 1dea.ent A 1a4m.ent A;451 1dea.ent A 4b5w.ent A;452 1dea.ent A 3q1n.ent A;453 1dea.ent A 3m66.ent A;454 1dea.ent A 3gae.ent A;455 1dea.ent A 3bcz.ent A;456 1dea.ent A 2voa.ent A;457 1dea.ent A 2p9h.ent A;458 1dea.ent A 2gnu.ent M;459 1dea.ent A 2ci1.ent A;460 1dea.ent A 1xq6.ent A;461 1dea.ent A 1tml.ent A;462 1dea.ent A 1qgj.ent A;463 1dea.ent A 1nas.ent A;464 1dea.ent A 1hvq.ent A;465 1dea.ent A 1fts.ent A;466 1b35.ent B 5mdh.ent A;467 1b35.ent B 3two.ent B;468 1b35.ent B 3n9s.ent A;469 1b35.ent B 3exe.ent B;470 1b35.ent B 2yy7.ent A;471 1b35.ent B 2qx3.ent A;472 1b35.ent B 2hk2.ent A;473 1b35.ent B 2eih.ent A;474 1b35.ent B 1yg9.ent A;475 1b35.ent B 1tca.ent A;476 1b35.ent B 1pxz.ent A;477 1b35.ent B 1llp.ent A;478 1b35.ent B 1h6u.ent A;479 1b35.ent B 1ea7.ent A;480 1b35.ent B 1a4m.ent A;481 1b35.ent B 4b5w.ent A;482 1b35.ent B 3q1n.ent A;483 1b35.ent B 3m66.ent A;484 1b35.ent B 3gae.ent A;485 1b35.ent B 3bcz.ent A;486 1b35.ent B 2voa.ent A;487 1b35.ent B 2p9h.ent A;488 1b35.ent B 2gnu.ent M;489 1b35.ent B 2ci1.ent A;490 1b35.ent B 1xq6.ent A;491 1b35.ent B 1tml.ent A;492 1b35.ent B 1qgj.ent A;493 1b35.ent B 1nas.ent A;494 1b35.ent B 1hvq.ent A;495 1b35.ent B 1fts.ent A;496 1b35.ent B 1dea.ent A;497 1a0g.ent B 5mdh.ent A;498 1a0g.ent B 3two.ent B;499 1a0g.ent B 3n9s.ent A;500 1a0g.ent B 3exe.ent B;501 1a0g.ent B 2yy7.ent A;502 1a0g.ent B 2qx3.ent A;503 1a0g.ent B 2hk2.ent A;504 1a0g.ent B 2eih.ent A;505 1a0g.ent B 1yg9.ent A;506 1a0g.ent B 1tca.ent A;507 1a0g.ent B 1pxz.ent A;508 1a0g.ent B 1llp.ent A;509 1a0g.ent B 1h6u.ent A;510 1a0g.ent B 1ea7.ent A;511 1a0g.ent B 1a4m.ent A;512 1a0g.ent B 4b5w.ent A;513 1a0g.ent B 3q1n.ent A;514 1a0g.ent B 3m66.ent A;515 1a0g.ent B 3gae.ent A;516 1a0g.ent B 3bcz.ent A;517 1a0g.ent B 2voa.ent A;518 1a0g.ent B 2p9h.ent A;519 1a0g.ent B 2gnu.ent M;520 1a0g.ent B 2ci1.ent A;521 1a0g.ent B 1xq6.ent A;522 1a0g.ent B 1tml.ent A;523 1a0g.ent B 1qgj.ent A;524 1a0g.ent B 1nas.ent A;525 1a0g.ent B 1hvq.ent A;526 1a0g.ent B 1fts.ent A;527 1a0g.ent B 1dea.ent A;528 1a0g.ent B 1b35.ent B;529 3tt9.ent A 5mdh.ent A;530 3tt9.ent A 3two.ent B;531 3tt9.ent A 3n9s.ent A;532 3tt9.ent A 3exe.ent B;533 3tt9.ent A 2yy7.ent A;534 3tt9.ent A 2qx3.ent A;535 3tt9.ent A 2hk2.ent A;536 3tt9.ent A 2eih.ent A;537 3tt9.ent A 1yg9.ent A;538 3tt9.ent A 1tca.ent A;539 3tt9.ent A 1pxz.ent A;540 3tt9.ent A 1llp.ent A;541 3tt9.ent A 1h6u.ent A;542 3tt9.ent A 1ea7.ent A;543 3tt9.ent A 1a4m.ent A;544 3tt9.ent A 4b5w.ent A;545 3tt9.ent A 3q1n.ent A;546 3tt9.ent A 3m66.ent A;547 3tt9.ent A 3gae.ent A;548 3tt9.ent A 3bcz.ent A;549 3tt9.ent A 2voa.ent A;550 3tt9.ent A 2p9h.ent A;551 3tt9.ent A 2gnu.ent M;552 3tt9.ent A 2ci1.ent A;553 3tt9.ent A 1xq6.ent A;554 3tt9.ent A 1tml.ent A;555 3tt9.ent A 1qgj.ent A;556 3tt9.ent A 1nas.ent A;557 3tt9.ent A 1hvq.ent A;558 3tt9.ent A 1fts.ent A;559 3tt9.ent A 1dea.ent A;560 3tt9.ent A 1b35.ent B;561 3tt9.ent A 1a0g.ent B;562 3pnl.ent B 5mdh.ent A;563 3pnl.ent B 3two.ent B;564 3pnl.ent B 3n9s.ent A;565 3pnl.ent B 3exe.ent B;566 3pnl.ent B 2yy7.ent A;567 3pnl.ent B 2qx3.ent A;568 3pnl.ent B 2hk2.ent A;569 3pnl.ent B 2eih.ent A;570 3pnl.ent B 1yg9.ent A;571 3pnl.ent B 1tca.ent A;572 3pnl.ent B 1pxz.ent A;573 3pnl.ent B 1llp.ent A;574 3pnl.ent B 1h6u.ent A;575 3pnl.ent B 1ea7.ent A;576 3pnl.ent B 1a4m.ent A;577 3pnl.ent B 4b5w.ent A;578 3pnl.ent B 3q1n.ent A;579 3pnl.ent B 3m66.ent A;580 3pnl.ent B 3gae.ent A;581 3pnl.ent B 3bcz.ent A;582 3pnl.ent B 2voa.ent A;583 3pnl.ent B 2p9h.ent A;584 3pnl.ent B 2gnu.ent M;585 3pnl.ent B 2ci1.ent A;586 3pnl.ent B 1xq6.ent A;587 3pnl.ent B 1tml.ent A;588 3pnl.ent B 1qgj.ent A;589 3pnl.ent B 1nas.ent A;590 3pnl.ent B 1hvq.ent A;591 3pnl.ent B 1fts.ent A;592 3pnl.ent B 1dea.ent A;593 3pnl.ent B 1b35.ent B;594 3pnl.ent B 1a0g.ent B;595 3pnl.ent B 3tt9.ent A;596 3kvd.ent D 5mdh.ent A;597 3kvd.ent D 3two.ent B;598 3kvd.ent D 3n9s.ent A;599 3kvd.ent D 3exe.ent B;600 3kvd.ent D 2yy7.ent A;601 3kvd.ent D 2qx3.ent A;602 3kvd.ent D 2hk2.ent A;603 3kvd.ent D 2eih.ent A;604 3kvd.ent D 1yg9.ent A;605 3kvd.ent D 1tca.ent A;606 3kvd.ent D 1pxz.ent A;607 3kvd.ent D 1llp.ent A;608 3kvd.ent D 1h6u.ent A;609 3kvd.ent D 1ea7.ent A;610 3kvd.ent D 1a4m.ent A;611 3kvd.ent D 4b5w.ent A;612 3kvd.ent D 3q1n.ent A;613 3kvd.ent D 3m66.ent A;614 3kvd.ent D 3gae.ent A;615 3kvd.ent D 3bcz.ent A;616 3kvd.ent D 2voa.ent A;617 3kvd.ent D 2p9h.ent A;618 3kvd.ent D 2gnu.ent M;619 3kvd.ent D 2ci1.ent A;620 3kvd.ent D 1xq6.ent A;621 3kvd.ent D 1tml.ent A;622 3kvd.ent D 1qgj.ent A;623 3kvd.ent D 1nas.ent A;624 3kvd.ent D 1hvq.ent A;625 3kvd.ent D 1fts.ent A;626 3kvd.ent D 1dea.ent A;627 3kvd.ent D 1b35.ent B;628 3kvd.ent D 1a0g.ent B;629 3kvd.ent D 3tt9.ent A;630 3kvd.ent D 3pnl.ent B;631 3fj7.ent A 5mdh.ent A;632 3fj7.ent A 3two.ent B;633 3fj7.ent A 3n9s.ent A;634 3fj7.ent A 3exe.ent B;635 3fj7.ent A 2yy7.ent A;636 3fj7.ent A 2qx3.ent A;637 3fj7.ent A 2hk2.ent A;638 3fj7.ent A 2eih.ent A;639 3fj7.ent A 1yg9.ent A;640 3fj7.ent A 1tca.ent A;641 3fj7.ent A 1pxz.ent A;642 3fj7.ent A 1llp.ent A;643 3fj7.ent A 1h6u.ent A;644 3fj7.ent A 1ea7.ent A;645 3fj7.ent A 1a4m.ent A;646 3fj7.ent A 4b5w.ent A;647 3fj7.ent A 3q1n.ent A;648 3fj7.ent A 3m66.ent A;649 3fj7.ent A 3gae.ent A;650 3fj7.ent A 3bcz.ent A;651 3fj7.ent A 2voa.ent A;652 3fj7.ent A 2p9h.ent A;653 3fj7.ent A 2gnu.ent M;654 3fj7.ent A 2ci1.ent A;655 3fj7.ent A 1xq6.ent A;656 3fj7.ent A 1tml.ent A;657 3fj7.ent A 1qgj.ent A;658 3fj7.ent A 1nas.ent A;659 3fj7.ent A 1hvq.ent A;660 3fj7.ent A 1fts.ent A;661 3fj7.ent A 1dea.ent A;662 3fj7.ent A 1b35.ent B;663 3fj7.ent A 1a0g.ent B;664 3fj7.ent A 3tt9.ent A;665 3fj7.ent A 3pnl.ent B;666 3fj7.ent A 3kvd.ent D;667 3cjj.ent A 5mdh.ent A;668 3cjj.ent A 3two.ent B;669 3cjj.ent A 3n9s.ent A;670 3cjj.ent A 3exe.ent B;671 3cjj.ent A 2yy7.ent A;672 3cjj.ent A 2qx3.ent A;673 3cjj.ent A 2hk2.ent A;674 3cjj.ent A 2eih.ent A;675 3cjj.ent A 1yg9.ent A;676 3cjj.ent A 1tca.ent A;677 3cjj.ent A 1pxz.ent A;678 3cjj.ent A 1llp.ent A;679 3cjj.ent A 1h6u.ent A;680 3cjj.ent A 1ea7.ent A;681 3cjj.ent A 1a4m.ent A;682 3cjj.ent A 4b5w.ent A;683 3cjj.ent A 3q1n.ent A;684 3cjj.ent A 3m66.ent A;685 3cjj.ent A 3gae.ent A;686 3cjj.ent A 3bcz.ent A;687 3cjj.ent A 2voa.ent A;688 3cjj.ent A 2p9h.ent A;689 3cjj.ent A 2gnu.ent M;690 3cjj.ent A 2ci1.ent A;691 3cjj.ent A 1xq6.ent A;692 3cjj.ent A 1tml.ent A;693 3cjj.ent A 1qgj.ent A;694 3cjj.ent A 1nas.ent A;695 3cjj.ent A 1hvq.ent A;696 3cjj.ent A 1fts.ent A;697 3cjj.ent A 1dea.ent A;698 3cjj.ent A 1b35.ent B;699 3cjj.ent A 1a0g.ent B;700 3cjj.ent A 3tt9.ent A;701 3cjj.ent A 3pnl.ent B;702 3cjj.ent A 3kvd.ent D;703 3cjj.ent A 3fj7.ent A;704 3ai9.ent X 5mdh.ent A;705 3ai9.ent X 3two.ent B;706 3ai9.ent X 3n9s.ent A;707 3ai9.ent X 3exe.ent B;708 3ai9.ent X 2yy7.ent A;709 3ai9.ent X 2qx3.ent A;710 3ai9.ent X 2hk2.ent A;711 3ai9.ent X 2eih.ent A;712 3ai9.ent X 1yg9.ent A;713 3ai9.ent X 1tca.ent A;714 3ai9.ent X 1pxz.ent A;715 3ai9.ent X 1llp.ent A;716 3ai9.ent X 1h6u.ent A;717 3ai9.ent X 1ea7.ent A;718 3ai9.ent X 1a4m.ent A;719 3ai9.ent X 4b5w.ent A;720 3ai9.ent X 3q1n.ent A;721 3ai9.ent X 3m66.ent A;722 3ai9.ent X 3gae.ent A;723 3ai9.ent X 3bcz.ent A;724 3ai9.ent X 2voa.ent A;725 3ai9.ent X 2p9h.ent A;726 3ai9.ent X 2gnu.ent M;727 3ai9.ent X 2ci1.ent A;728 3ai9.ent X 1xq6.ent A;729 3ai9.ent X 1tml.ent A;730 3ai9.ent X 1qgj.ent A;731 3ai9.ent X 1nas.ent A;732 3ai9.ent X 1hvq.ent A;733 3ai9.ent X 1fts.ent A;734 3ai9.ent X 1dea.ent A;735 3ai9.ent X 1b35.ent B;736 3ai9.ent X 1a0g.ent B;737 3ai9.ent X 3tt9.ent A;738 3ai9.ent X 3pnl.ent B;739 3ai9.ent X 3kvd.ent D;740 3ai9.ent X 3fj7.ent A;741 3ai9.ent X 3cjj.ent A;742 2vg9.ent A 5mdh.ent A;743 2vg9.ent A 3two.ent B;744 2vg9.ent A 3n9s.ent A;745 2vg9.ent A 3exe.ent B;746 2vg9.ent A 2yy7.ent A;747 2vg9.ent A 2qx3.ent A;748 2vg9.ent A 2hk2.ent A;749 2vg9.ent A 2eih.ent A;750 2vg9.ent A 1yg9.ent A;751 2vg9.ent A 1tca.ent A;752 2vg9.ent A 1pxz.ent A;753 2vg9.ent A 1llp.ent A;754 2vg9.ent A 1h6u.ent A;755 2vg9.ent A 1ea7.ent A;756 2vg9.ent A 1a4m.ent A;757 2vg9.ent A 4b5w.ent A;758 2vg9.ent A 3q1n.ent A;759 2vg9.ent A 3m66.ent A;760 2vg9.ent A 3gae.ent A;761 2vg9.ent A 3bcz.ent A;762 2vg9.ent A 2voa.ent A;763 2vg9.ent A 2p9h.ent A;764 2vg9.ent A 2gnu.ent M;765 2vg9.ent A 2ci1.ent A;766 2vg9.ent A 1xq6.ent A;767 2vg9.ent A 1tml.ent A;768 2vg9.ent A 1qgj.ent A;769 2vg9.ent A 1nas.ent A;770 2vg9.ent A 1hvq.ent A;771 2vg9.ent A 1fts.ent A;772 2vg9.ent A 1dea.ent A;773 2vg9.ent A 1b35.ent B;774 2vg9.ent A 1a0g.ent B;775 2vg9.ent A 3tt9.ent A;776 2vg9.ent A 3pnl.ent B;777 2vg9.ent A 3kvd.ent D;778 2vg9.ent A 3fj7.ent A;779 2vg9.ent A 3cjj.ent A;780 2vg9.ent A 3ai9.ent X;781 2p0j.ent A 5mdh.ent A;782 2p0j.ent A 3two.ent B;783 2p0j.ent A 3n9s.ent A;784 2p0j.ent A 3exe.ent B;785 2p0j.ent A 2yy7.ent A;786 2p0j.ent A 2qx3.ent A;787 2p0j.ent A 2hk2.ent A;788 2p0j.ent A 2eih.ent A;789 2p0j.ent A 1yg9.ent A;790 2p0j.ent A 1tca.ent A;791 2p0j.ent A 1pxz.ent A;792 2p0j.ent A 1llp.ent A;793 2p0j.ent A 1h6u.ent A;794 2p0j.ent A 1ea7.ent A;795 2p0j.ent A 1a4m.ent A;796 2p0j.ent A 4b5w.ent A;797 2p0j.ent A 3q1n.ent A;798 2p0j.ent A 3m66.ent A;799 2p0j.ent A 3gae.ent A;800 2p0j.ent A 3bcz.ent A;801 2p0j.ent A 2voa.ent A;802 2p0j.ent A 2p9h.ent A;803 2p0j.ent A 2gnu.ent M;804 2p0j.ent A 2ci1.ent A;805 2p0j.ent A 1xq6.ent A;806 2p0j.ent A 1tml.ent A;807 2p0j.ent A 1qgj.ent A;808 2p0j.ent A 1nas.ent A;809 2p0j.ent A 1hvq.ent A;810 2p0j.ent A 1fts.ent A;811 2p0j.ent A 1dea.ent A;812 2p0j.ent A 1b35.ent B;813 2p0j.ent A 1a0g.ent B;814 2p0j.ent A 3tt9.ent A;815 2p0j.ent A 3pnl.ent B;816 2p0j.ent A 3kvd.ent D;817 2p0j.ent A 3fj7.ent A;818 2p0j.ent A 3cjj.ent A;819 2p0j.ent A 3ai9.ent X;820 2p0j.ent A 2vg9.ent A;821 2is9.ent A 5mdh.ent A;822 2is9.ent A 3two.ent B;823 2is9.ent A 3n9s.ent A;824 2is9.ent A 3exe.ent B;825 2is9.ent A 2yy7.ent A;826 2is9.ent A 2qx3.ent A;827 2is9.ent A 2hk2.ent A;828 2is9.ent A 2eih.ent A;829 2is9.ent A 1yg9.ent A;830 2is9.ent A 1tca.ent A;831 2is9.ent A 1pxz.ent A;832 2is9.ent A 1llp.ent A;833 2is9.ent A 1h6u.ent A;834 2is9.ent A 1ea7.ent A;835 2is9.ent A 1a4m.ent A;836 2is9.ent A 4b5w.ent A;837 2is9.ent A 3q1n.ent A;838 2is9.ent A 3m66.ent A;839 2is9.ent A 3gae.ent A;840 2is9.ent A 3bcz.ent A;841 2is9.ent A 2voa.ent A;842 2is9.ent A 2p9h.ent A;843 2is9.ent A 2gnu.ent M;844 2is9.ent A 2ci1.ent A;845 2is9.ent A 1xq6.ent A;846 2is9.ent A 1tml.ent A;847 2is9.ent A 1qgj.ent A;848 2is9.ent A 1nas.ent A;849 2is9.ent A 1hvq.ent A;850 2is9.ent A 1fts.ent A;851 2is9.ent A 1dea.ent A;852 2is9.ent A 1b35.ent B;853 2is9.ent A 1a0g.ent B;854 2is9.ent A 3tt9.ent A;855 2is9.ent A 3pnl.ent B;856 2is9.ent A 3kvd.ent D;857 2is9.ent A 3fj7.ent A;858 2is9.ent A 3cjj.ent A;859 2is9.ent A 3ai9.ent X;860 2is9.ent A 2vg9.ent A;861 2is9.ent A 2p0j.ent A;862 2eyi.ent A 5mdh.ent A;863 2eyi.ent A 3two.ent B;864 2eyi.ent A 3n9s.ent A;865 2eyi.ent A 3exe.ent B;866 2eyi.ent A 2yy7.ent A;867 2eyi.ent A 2qx3.ent A;868 2eyi.ent A 2hk2.ent A;869 2eyi.ent A 2eih.ent A;870 2eyi.ent A 1yg9.ent A;871 2eyi.ent A 1tca.ent A;872 2eyi.ent A 1pxz.ent A;873 2eyi.ent A 1llp.ent A;874 2eyi.ent A 1h6u.ent A;875 2eyi.ent A 1ea7.ent A;876 2eyi.ent A 1a4m.ent A;877 2eyi.ent A 4b5w.ent A;878 2eyi.ent A 3q1n.ent A;879 2eyi.ent A 3m66.ent A;880 2eyi.ent A 3gae.ent A;881 2eyi.ent A 3bcz.ent A;882 2eyi.ent A 2voa.ent A;883 2eyi.ent A 2p9h.ent A;884 2eyi.ent A 2gnu.ent M;885 2eyi.ent A 2ci1.ent A;886 2eyi.ent A 1xq6.ent A;887 2eyi.ent A 1tml.ent A;888 2eyi.ent A 1qgj.ent A;889 2eyi.ent A 1nas.ent A;890 2eyi.ent A 1hvq.ent A;891 2eyi.ent A 1fts.ent A;892 2eyi.ent A 1dea.ent A;893 2eyi.ent A 1b35.ent B;894 2eyi.ent A 1a0g.ent B;895 2eyi.ent A 3tt9.ent A;896 2eyi.ent A 3pnl.ent B;897 2eyi.ent A 3kvd.ent D;898 2eyi.ent A 3fj7.ent A;899 2eyi.ent A 3cjj.ent A;900 2eyi.ent A 3ai9.ent X;901 2eyi.ent A 2vg9.ent A;902 2eyi.ent A 2p0j.ent A;903 2eyi.ent A 2is9.ent A;904 2cga.ent A 5mdh.ent A;905 2cga.ent A 3two.ent B;906 2cga.ent A 3n9s.ent A;907 2cga.ent A 3exe.ent B;908 2cga.ent A 2yy7.ent A;909 2cga.ent A 2qx3.ent A;910 2cga.ent A 2hk2.ent A;911 2cga.ent A 2eih.ent A;912 2cga.ent A 1yg9.ent A;913 2cga.ent A 1tca.ent A;914 2cga.ent A 1pxz.ent A;915 2cga.ent A 1llp.ent A;916 2cga.ent A 1h6u.ent A;917 2cga.ent A 1ea7.ent A;918 2cga.ent A 1a4m.ent A;919 2cga.ent A 4b5w.ent A;920 2cga.ent A 3q1n.ent A;921 2cga.ent A 3m66.ent A;922 2cga.ent A 3gae.ent A;923 2cga.ent A 3bcz.ent A;924 2cga.ent A 2voa.ent A;925 2cga.ent A 2p9h.ent A;926 2cga.ent A 2gnu.ent M;927 2cga.ent A 2ci1.ent A;928 2cga.ent A 1xq6.ent A;929 2cga.ent A 1tml.ent A;930 2cga.ent A 1qgj.ent A;931 2cga.ent A 1nas.ent A;932 2cga.ent A 1hvq.ent A;933 2cga.ent A 1fts.ent A;934 2cga.ent A 1dea.ent A;935 2cga.ent A 1b35.ent B;936 2cga.ent A 1a0g.ent B;937 2cga.ent A 3tt9.ent A;938 2cga.ent A 3pnl.ent B;939 2cga.ent A 3kvd.ent D;940 2cga.ent A 3fj7.ent A;941 2cga.ent A 3cjj.ent A;942 2cga.ent A 3ai9.ent X;943 2cga.ent A 2vg9.ent A;944 2cga.ent A 2p0j.ent A;945 2cga.ent A 2is9.ent A;946 2cga.ent A 2eyi.ent A;947 1ym0.ent A 5mdh.ent A;948 1ym0.ent A 3two.ent B;949 1ym0.ent A 3n9s.ent A;950 1ym0.ent A 3exe.ent B;951 1ym0.ent A 2yy7.ent A;952 1ym0.ent A 2qx3.ent A;953 1ym0.ent A 2hk2.ent A;954 1ym0.ent A 2eih.ent A;955 1ym0.ent A 1yg9.ent A;956 1ym0.ent A 1tca.ent A;957 1ym0.ent A 1pxz.ent A;958 1ym0.ent A 1llp.ent A;959 1ym0.ent A 1h6u.ent A;960 1ym0.ent A 1ea7.ent A;961 1ym0.ent A 1a4m.ent A;962 1ym0.ent A 4b5w.ent A;963 1ym0.ent A 3q1n.ent A;964 1ym0.ent A 3m66.ent A;965 1ym0.ent A 3gae.ent A;966 1ym0.ent A 3bcz.ent A;967 1ym0.ent A 2voa.ent A;968 1ym0.ent A 2p9h.ent A;969 1ym0.ent A 2gnu.ent M;970 1ym0.ent A 2ci1.ent A;971 1ym0.ent A 1xq6.ent A;972 1ym0.ent A 1tml.ent A;973 1ym0.ent A 1qgj.ent A;974 1ym0.ent A 1nas.ent A;975 1ym0.ent A 1hvq.ent A;976 1ym0.ent A 1fts.ent A;977 1ym0.ent A 1dea.ent A;978 1ym0.ent A 1b35.ent B;979 1ym0.ent A 1a0g.ent B;980 1ym0.ent A 3tt9.ent A;981 1ym0.ent A 3pnl.ent B;982 1ym0.ent A 3kvd.ent D;983 1ym0.ent A 3fj7.ent A;984 1ym0.ent A 3cjj.ent A;985 1ym0.ent A 3ai9.ent X;986 1ym0.ent A 2vg9.ent A;987 1ym0.ent A 2p0j.ent A;988 1ym0.ent A 2is9.ent A;989 1ym0.ent A 2eyi.ent A;990 1ym0.ent A 2cga.ent A;991 1twf.ent E 5mdh.ent A;992 1twf.ent E 3two.ent B;993 1twf.ent E 3n9s.ent A;994 1twf.ent E 3exe.ent B;995 1twf.ent E 2yy7.ent A;996 1twf.ent E 2qx3.ent A;997 1twf.ent E 2hk2.ent A;998 1twf.ent E 2eih.ent A;999 1twf.ent E 1yg9.ent A;1000 1twf.ent E 1tca.ent A;1001 1twf.ent E 1pxz.ent A;1002 1twf.ent E 1llp.ent A;1003 1twf.ent E 1h6u.ent A;1004 1twf.ent E 1ea7.ent A;1005 1twf.ent E 1a4m.ent A;1006 1twf.ent E 4b5w.ent A;1007 1twf.ent E 3q1n.ent A;1008 1twf.ent E 3m66.ent A;1009 1twf.ent E 3gae.ent A;1010 1twf.ent E 3bcz.ent A;1011 1twf.ent E 2voa.ent A;1012 1twf.ent E 2p9h.ent A;1013 1twf.ent E 2gnu.ent M;1014 1twf.ent E 2ci1.ent A;1015 1twf.ent E 1xq6.ent A;1016 1twf.ent E 1tml.ent A;1017 1twf.ent E 1qgj.ent A;1018 1twf.ent E 1nas.ent A;1019 1twf.ent E 1hvq.ent A;1020 1twf.ent E 1fts.ent A;1021 1twf.ent E 1dea.ent A;1022 1twf.ent E 1b35.ent B;1023 1twf.ent E 1a0g.ent B;1024 1twf.ent E 3tt9.ent A;1025 1twf.ent E 3pnl.ent B;1026 1twf.ent E 3kvd.ent D;1027 1twf.ent E 3fj7.ent A;1028 1twf.ent E 3cjj.ent A;1029 1twf.ent E 3ai9.ent X;1030 1twf.ent E 2vg9.ent A;1031 1twf.ent E 2p0j.ent A;1032 1twf.ent E 2is9.ent A;1033 1twf.ent E 2eyi.ent A;1034 1twf.ent E 2cga.ent A;1035 1twf.ent E 1ym0.ent A;1036 1rc9.ent A 5mdh.ent A;1037 1rc9.ent A 3two.ent B;1038 1rc9.ent A 3n9s.ent A;1039 1rc9.ent A 3exe.ent B;1040 1rc9.ent A 2yy7.ent A;1041 1rc9.ent A 2qx3.ent A;1042 1rc9.ent A 2hk2.ent A;1043 1rc9.ent A 2eih.ent A;1044 1rc9.ent A 1yg9.ent A;1045 1rc9.ent A 1tca.ent A;1046 1rc9.ent A 1pxz.ent A;1047 1rc9.ent A 1llp.ent A;1048 1rc9.ent A 1h6u.ent A;1049 1rc9.ent A 1ea7.ent A;1050 1rc9.ent A 1a4m.ent A;1051 1rc9.ent A 4b5w.ent A;1052 1rc9.ent A 3q1n.ent A;1053 1rc9.ent A 3m66.ent A;1054 1rc9.ent A 3gae.ent A;1055 1rc9.ent A 3bcz.ent A;1056 1rc9.ent A 2voa.ent A;1057 1rc9.ent A 2p9h.ent A;1058 1rc9.ent A 2gnu.ent M;1059 1rc9.ent A 2ci1.ent A;1060 1rc9.ent A 1xq6.ent A;1061 1rc9.ent A 1tml.ent A;1062 1rc9.ent A 1qgj.ent A;1063 1rc9.ent A 1nas.ent A;1064 1rc9.ent A 1hvq.ent A;1065 1rc9.ent A 1fts.ent A;1066 1rc9.ent A 1dea.ent A;1067 1rc9.ent A 1b35.ent B;1068 1rc9.ent A 1a0g.ent B;1069 1rc9.ent A 3tt9.ent A;1070 1rc9.ent A 3pnl.ent B;1071 1rc9.ent A 3kvd.ent D;1072 1rc9.ent A 3fj7.ent A;1073 1rc9.ent A 3cjj.ent A;1074 1rc9.ent A 3ai9.ent X;1075 1rc9.ent A 2vg9.ent A;1076 1rc9.ent A 2p0j.ent A;1077 1rc9.ent A 2is9.ent A;1078 1rc9.ent A 2eyi.ent A;1079 1rc9.ent A 2cga.ent A;1080 1rc9.ent A 1ym0.ent A;1081 1rc9.ent A 1twf.ent E;1082 1pn9.ent A 5mdh.ent A;1083 1pn9.ent A 3two.ent B;1084 1pn9.ent A 3n9s.ent A;1085 1pn9.ent A 3exe.ent B;1086 1pn9.ent A 2yy7.ent A;1087 1pn9.ent A 2qx3.ent A;1088 1pn9.ent A 2hk2.ent A;1089 1pn9.ent A 2eih.ent A;1090 1pn9.ent A 1yg9.ent A;1091 1pn9.ent A 1tca.ent A;1092 1pn9.ent A 1pxz.ent A;1093 1pn9.ent A 1llp.ent A;1094 1pn9.ent A 1h6u.ent A;1095 1pn9.ent A 1ea7.ent A;1096 1pn9.ent A 1a4m.ent A;1097 1pn9.ent A 4b5w.ent A;1098 1pn9.ent A 3q1n.ent A;1099 1pn9.ent A 3m66.ent A;1100 1pn9.ent A 3gae.ent A;1101 1pn9.ent A 3bcz.ent A;1102 1pn9.ent A 2voa.ent A;1103 1pn9.ent A 2p9h.ent A;1104 1pn9.ent A 2gnu.ent M;1105 1pn9.ent A 2ci1.ent A;1106 1pn9.ent A 1xq6.ent A;1107 1pn9.ent A 1tml.ent A;1108 1pn9.ent A 1qgj.ent A;1109 1pn9.ent A 1nas.ent A;1110 1pn9.ent A 1hvq.ent A;1111 1pn9.ent A 1fts.ent A;1112 1pn9.ent A 1dea.ent A;1113 1pn9.ent A 1b35.ent B;1114 1pn9.ent A 1a0g.ent B;1115 1pn9.ent A 3tt9.ent A;1116 1pn9.ent A 3pnl.ent B;1117 1pn9.ent A 3kvd.ent D;1118 1pn9.ent A 3fj7.ent A;1119 1pn9.ent A 3cjj.ent A;1120 1pn9.ent A 3ai9.ent X;1121 1pn9.ent A 2vg9.ent A;1122 1pn9.ent A 2p0j.ent A;1123 1pn9.ent A 2is9.ent A;1124 1pn9.ent A 2eyi.ent A;1125 1pn9.ent A 2cga.ent A;1126 1pn9.ent A 1ym0.ent A;1127 1pn9.ent A 1twf.ent E;1128 1pn9.ent A 1rc9.ent A;1129 1n7k.ent A 5mdh.ent A;1130 1n7k.ent A 3two.ent B;1131 1n7k.ent A 3n9s.ent A;1132 1n7k.ent A 3exe.ent B;1133 1n7k.ent A 2yy7.ent A;1134 1n7k.ent A 2qx3.ent A;1135 1n7k.ent A 2hk2.ent A;1136 1n7k.ent A 2eih.ent A;1137 1n7k.ent A 1yg9.ent A;1138 1n7k.ent A 1tca.ent A;1139 1n7k.ent A 1pxz.ent A;1140 1n7k.ent A 1llp.ent A;1141 1n7k.ent A 1h6u.ent A;1142 1n7k.ent A 1ea7.ent A;1143 1n7k.ent A 1a4m.ent A;1144 1n7k.ent A 4b5w.ent A;1145 1n7k.ent A 3q1n.ent A;1146 1n7k.ent A 3m66.ent A;1147 1n7k.ent A 3gae.ent A;1148 1n7k.ent A 3bcz.ent A;1149 1n7k.ent A 2voa.ent A;1150 1n7k.ent A 2p9h.ent A;1151 1n7k.ent A 2gnu.ent M;1152 1n7k.ent A 2ci1.ent A;1153 1n7k.ent A 1xq6.ent A;1154 1n7k.ent A 1tml.ent A;1155 1n7k.ent A 1qgj.ent A;1156 1n7k.ent A 1nas.ent A;1157 1n7k.ent A 1hvq.ent A;1158 1n7k.ent A 1fts.ent A;1159 1n7k.ent A 1dea.ent A;1160 1n7k.ent A 1b35.ent B;1161 1n7k.ent A 1a0g.ent B;1162 1n7k.ent A 3tt9.ent A;1163 1n7k.ent A 3pnl.ent B;1164 1n7k.ent A 3kvd.ent D;1165 1n7k.ent A 3fj7.ent A;1166 1n7k.ent A 3cjj.ent A;1167 1n7k.ent A 3ai9.ent X;1168 1n7k.ent A 2vg9.ent A;1169 1n7k.ent A 2p0j.ent A;1170 1n7k.ent A 2is9.ent A;1171 1n7k.ent A 2eyi.ent A;1172 1n7k.ent A 2cga.ent A;1173 1n7k.ent A 1ym0.ent A;1174 1n7k.ent A 1twf.ent E;1175 1n7k.ent A 1rc9.ent A;1176 1n7k.ent A 1pn9.ent A;1177 1kg5.ent A 5mdh.ent A;1178 1kg5.ent A 3two.ent B;1179 1kg5.ent A 3n9s.ent A;1180 1kg5.ent A 3exe.ent B;1181 1kg5.ent A 2yy7.ent A;1182 1kg5.ent A 2qx3.ent A;1183 1kg5.ent A 2hk2.ent A;1184 1kg5.ent A 2eih.ent A;1185 1kg5.ent A 1yg9.ent A;1186 1kg5.ent A 1tca.ent A;1187 1kg5.ent A 1pxz.ent A;1188 1kg5.ent A 1llp.ent A;1189 1kg5.ent A 1h6u.ent A;1190 1kg5.ent A 1ea7.ent A;1191 1kg5.ent A 1a4m.ent A;1192 1kg5.ent A 4b5w.ent A;1193 1kg5.ent A 3q1n.ent A;1194 1kg5.ent A 3m66.ent A;1195 1kg5.ent A 3gae.ent A;1196 1kg5.ent A 3bcz.ent A;1197 1kg5.ent A 2voa.ent A;1198 1kg5.ent A 2p9h.ent A;1199 1kg5.ent A 2gnu.ent M;1200 1kg5.ent A 2ci1.ent A;1201 1kg5.ent A 1xq6.ent A;1202 1kg5.ent A 1tml.ent A;1203 1kg5.ent A 1qgj.ent A;1204 1kg5.ent A 1nas.ent A;1205 1kg5.ent A 1hvq.ent A;1206 1kg5.ent A 1fts.ent A;1207 1kg5.ent A 1dea.ent A;1208 1kg5.ent A 1b35.ent B;1209 1kg5.ent A 1a0g.ent B;1210 1kg5.ent A 3tt9.ent A;1211 1kg5.ent A 3pnl.ent B;1212 1kg5.ent A 3kvd.ent D;1213 1kg5.ent A 3fj7.ent A;1214 1kg5.ent A 3cjj.ent A;1215 1kg5.ent A 3ai9.ent X;1216 1kg5.ent A 2vg9.ent A;1217 1kg5.ent A 2p0j.ent A;1218 1kg5.ent A 2is9.ent A;1219 1kg5.ent A 2eyi.ent A;1220 1kg5.ent A 2cga.ent A;1221 1kg5.ent A 1ym0.ent A;1222 1kg5.ent A 1twf.ent E;1223 1kg5.ent A 1rc9.ent A;1224 1kg5.ent A 1pn9.ent A;1225 1kg5.ent A 1n7k.ent A;1226 1gis.ent A 5mdh.ent A;1227 1gis.ent A 3two.ent B;1228 1gis.ent A 3n9s.ent A;1229 1gis.ent A 3exe.ent B;1230 1gis.ent A 2yy7.ent A;1231 1gis.ent A 2qx3.ent A;1232 1gis.ent A 2hk2.ent A;1233 1gis.ent A 2eih.ent A;1234 1gis.ent A 1yg9.ent A;1235 1gis.ent A 1tca.ent A;1236 1gis.ent A 1pxz.ent A;1237 1gis.ent A 1llp.ent A;1238 1gis.ent A 1h6u.ent A;1239 1gis.ent A 1ea7.ent A;1240 1gis.ent A 1a4m.ent A;1241 1gis.ent A 4b5w.ent A;1242 1gis.ent A 3q1n.ent A;1243 1gis.ent A 3m66.ent A;1244 1gis.ent A 3gae.ent A;1245 1gis.ent A 3bcz.ent A;1246 1gis.ent A 2voa.ent A;1247 1gis.ent A 2p9h.ent A;1248 1gis.ent A 2gnu.ent M;1249 1gis.ent A 2ci1.ent A;1250 1gis.ent A 1xq6.ent A;1251 1gis.ent A 1tml.ent A;1252 1gis.ent A 1qgj.ent A;1253 1gis.ent A 1nas.ent A;1254 1gis.ent A 1hvq.ent A;1255 1gis.ent A 1fts.ent A;1256 1gis.ent A 1dea.ent A;1257 1gis.ent A 1b35.ent B;1258 1gis.ent A 1a0g.ent B;1259 1gis.ent A 3tt9.ent A;1260 1gis.ent A 3pnl.ent B;1261 1gis.ent A 3kvd.ent D;1262 1gis.ent A 3fj7.ent A;1263 1gis.ent A 3cjj.ent A;1264 1gis.ent A 3ai9.ent X;1265 1gis.ent A 2vg9.ent A;1266 1gis.ent A 2p0j.ent A;1267 1gis.ent A 2is9.ent A;1268 1gis.ent A 2eyi.ent A;1269 1gis.ent A 2cga.ent A;1270 1gis.ent A 1ym0.ent A;1271 1gis.ent A 1twf.ent E;1272 1gis.ent A 1rc9.ent A;1273 1gis.ent A 1pn9.ent A;1274 1gis.ent A 1n7k.ent A;1275 1gis.ent A 1kg5.ent A;1276 1fuj.ent A 5mdh.ent A;1277 1fuj.ent A 3two.ent B;1278 1fuj.ent A 3n9s.ent A;1279 1fuj.ent A 3exe.ent B;1280 1fuj.ent A 2yy7.ent A;1281 1fuj.ent A 2qx3.ent A;1282 1fuj.ent A 2hk2.ent A;1283 1fuj.ent A 2eih.ent A;1284 1fuj.ent A 1yg9.ent A;1285 1fuj.ent A 1tca.ent A;1286 1fuj.ent A 1pxz.ent A;1287 1fuj.ent A 1llp.ent A;1288 1fuj.ent A 1h6u.ent A;1289 1fuj.ent A 1ea7.ent A;1290 1fuj.ent A 1a4m.ent A;1291 1fuj.ent A 4b5w.ent A;1292 1fuj.ent A 3q1n.ent A;1293 1fuj.ent A 3m66.ent A;1294 1fuj.ent A 3gae.ent A;1295 1fuj.ent A 3bcz.ent A;1296 1fuj.ent A 2voa.ent A;1297 1fuj.ent A 2p9h.ent A;1298 1fuj.ent A 2gnu.ent M;1299 1fuj.ent A 2ci1.ent A;1300 1fuj.ent A 1xq6.ent A;1301 1fuj.ent A 1tml.ent A;1302 1fuj.ent A 1qgj.ent A;1303 1fuj.ent A 1nas.ent A;1304 1fuj.ent A 1hvq.ent A;1305 1fuj.ent A 1fts.ent A;1306 1fuj.ent A 1dea.ent A;1307 1fuj.ent A 1b35.ent B;1308 1fuj.ent A 1a0g.ent B;1309 1fuj.ent A 3tt9.ent A;1310 1fuj.ent A 3pnl.ent B;1311 1fuj.ent A 3kvd.ent D;1312 1fuj.ent A 3fj7.ent A;1313 1fuj.ent A 3cjj.ent A;1314 1fuj.ent A 3ai9.ent X;1315 1fuj.ent A 2vg9.ent A;1316 1fuj.ent A 2p0j.ent A;1317 1fuj.ent A 2is9.ent A;1318 1fuj.ent A 2eyi.ent A;1319 1fuj.ent A 2cga.ent A;1320 1fuj.ent A 1ym0.ent A;1321 1fuj.ent A 1twf.ent E;1322 1fuj.ent A 1rc9.ent A;1323 1fuj.ent A 1pn9.ent A;1324 1fuj.ent A 1n7k.ent A;1325 1fuj.ent A 1kg5.ent A;1326 1fuj.ent A 1gis.ent A;1327 1del.ent A 5mdh.ent A;1328 1del.ent A 3two.ent B;1329 1del.ent A 3n9s.ent A;1330 1del.ent A 3exe.ent B;1331 1del.ent A 2yy7.ent A;1332 1del.ent A 2qx3.ent A;1333 1del.ent A 2hk2.ent A;1334 1del.ent A 2eih.ent A;1335 1del.ent A 1yg9.ent A;1336 1del.ent A 1tca.ent A;1337 1del.ent A 1pxz.ent A;1338 1del.ent A 1llp.ent A;1339 1del.ent A 1h6u.ent A;1340 1del.ent A 1ea7.ent A;1341 1del.ent A 1a4m.ent A;1342 1del.ent A 4b5w.ent A;1343 1del.ent A 3q1n.ent A;1344 1del.ent A 3m66.ent A;1345 1del.ent A 3gae.ent A;1346 1del.ent A 3bcz.ent A;1347 1del.ent A 2voa.ent A;1348 1del.ent A 2p9h.ent A;1349 1del.ent A 2gnu.ent M;1350 1del.ent A 2ci1.ent A;1351 1del.ent A 1xq6.ent A;1352 1del.ent A 1tml.ent A;1353 1del.ent A 1qgj.ent A;1354 1del.ent A 1nas.ent A;1355 1del.ent A 1hvq.ent A;1356 1del.ent A 1fts.ent A;1357 1del.ent A 1dea.ent A;1358 1del.ent A 1b35.ent B;1359 1del.ent A 1a0g.ent B;1360 1del.ent A 3tt9.ent A;1361 1del.ent A 3pnl.ent B;1362 1del.ent A 3kvd.ent D;1363 1del.ent A 3fj7.ent A;1364 1del.ent A 3cjj.ent A;1365 1del.ent A 3ai9.ent X;1366 1del.ent A 2vg9.ent A;1367 1del.ent A 2p0j.ent A;1368 1del.ent A 2is9.ent A;1369 1del.ent A 2eyi.ent A;1370 1del.ent A 2cga.ent A;1371 1del.ent A 1ym0.ent A;1372 1del.ent A 1twf.ent E;1373 1del.ent A 1rc9.ent A;1374 1del.ent A 1pn9.ent A;1375 1del.ent A 1n7k.ent A;1376 1del.ent A 1kg5.ent A;1377 1del.ent A 1gis.ent A;1378 1del.ent A 1fuj.ent A;1379 1b77.ent A 5mdh.ent A;1380 1b77.ent A 3two.ent B;1381 1b77.ent A 3n9s.ent A;1382 1b77.ent A 3exe.ent B;1383 1b77.ent A 2yy7.ent A;1384 1b77.ent A 2qx3.ent A;1385 1b77.ent A 2hk2.ent A;1386 1b77.ent A 2eih.ent A;1387 1b77.ent A 1yg9.ent A;1388 1b77.ent A 1tca.ent A;1389 1b77.ent A 1pxz.ent A;1390 1b77.ent A 1llp.ent A;1391 1b77.ent A 1h6u.ent A;1392 1b77.ent A 1ea7.ent A;1393 1b77.ent A 1a4m.ent A;1394 1b77.ent A 4b5w.ent A;1395 1b77.ent A 3q1n.ent A;1396 1b77.ent A 3m66.ent A;1397 1b77.ent A 3gae.ent A;1398 1b77.ent A 3bcz.ent A;1399 1b77.ent A 2voa.ent A;1400 1b77.ent A 2p9h.ent A;1401 1b77.ent A 2gnu.ent M;1402 1b77.ent A 2ci1.ent A;1403 1b77.ent A 1xq6.ent A;1404 1b77.ent A 1tml.ent A;1405 1b77.ent A 1qgj.ent A;1406 1b77.ent A 1nas.ent A;1407 1b77.ent A 1hvq.ent A;1408 1b77.ent A 1fts.ent A;1409 1b77.ent A 1dea.ent A;1410 1b77.ent A 1b35.ent B;1411 1b77.ent A 1a0g.ent B;1412 1b77.ent A 3tt9.ent A;1413 1b77.ent A 3pnl.ent B;1414 1b77.ent A 3kvd.ent D;1415 1b77.ent A 3fj7.ent A;1416 1b77.ent A 3cjj.ent A;1417 1b77.ent A 3ai9.ent X;1418 1b77.ent A 2vg9.ent A;1419 1b77.ent A 2p0j.ent A;1420 1b77.ent A 2is9.ent A;1421 1b77.ent A 2eyi.ent A;1422 1b77.ent A 2cga.ent A;1423 1b77.ent A 1ym0.ent A;1424 1b77.ent A 1twf.ent E;1425 1b77.ent A 1rc9.ent A;1426 1b77.ent A 1pn9.ent A;1427 1b77.ent A 1n7k.ent A;1428 1b77.ent A 1kg5.ent A;1429 1b77.ent A 1gis.ent A;1430 1b77.ent A 1fuj.ent A;1431 1b77.ent A 1del.ent A;1432 4h6a.ent A 5mdh.ent A;1433 4h6a.ent A 3two.ent B;1434 4h6a.ent A 3n9s.ent A;1435 4h6a.ent A 3exe.ent B;1436 4h6a.ent A 2yy7.ent A;1437 4h6a.ent A 2qx3.ent A;1438 4h6a.ent A 2hk2.ent A;1439 4h6a.ent A 2eih.ent A;1440 4h6a.ent A 1yg9.ent A;1441 4h6a.ent A 1tca.ent A;1442 4h6a.ent A 1pxz.ent A;1443 4h6a.ent A 1llp.ent A;1444 4h6a.ent A 1h6u.ent A;1445 4h6a.ent A 1ea7.ent A;1446 4h6a.ent A 1a4m.ent A;1447 4h6a.ent A 4b5w.ent A;1448 4h6a.ent A 3q1n.ent A;1449 4h6a.ent A 3m66.ent A;1450 4h6a.ent A 3gae.ent A;1451 4h6a.ent A 3bcz.ent A;1452 4h6a.ent A 2voa.ent A;1453 4h6a.ent A 2p9h.ent A;1454 4h6a.ent A 2gnu.ent M;1455 4h6a.ent A 2ci1.ent A;1456 4h6a.ent A 1xq6.ent A;1457 4h6a.ent A 1tml.ent A;1458 4h6a.ent A 1qgj.ent A;1459 4h6a.ent A 1nas.ent A;1460 4h6a.ent A 1hvq.ent A;1461 4h6a.ent A 1fts.ent A;1462 4h6a.ent A 1dea.ent A;1463 4h6a.ent A 1b35.ent B;1464 4h6a.ent A 1a0g.ent B;1465 4h6a.ent A 3tt9.ent A;1466 4h6a.ent A 3pnl.ent B;1467 4h6a.ent A 3kvd.ent D;1468 4h6a.ent A 3fj7.ent A;1469 4h6a.ent A 3cjj.ent A;1470 4h6a.ent A 3ai9.ent X;1471 4h6a.ent A 2vg9.ent A;1472 4h6a.ent A 2p0j.ent A;1473 4h6a.ent A 2is9.ent A;1474 4h6a.ent A 2eyi.ent A;1475 4h6a.ent A 2cga.ent A;1476 4h6a.ent A 1ym0.ent A;1477 4h6a.ent A 1twf.ent E;1478 4h6a.ent A 1rc9.ent A;1479 4h6a.ent A 1pn9.ent A;1480 4h6a.ent A 1n7k.ent A;1481 4h6a.ent A 1kg5.ent A;1482 4h6a.ent A 1gis.ent A;1483 4h6a.ent A 1fuj.ent A;1484 4h6a.ent A 1del.ent A;1485 4h6a.ent A 1b77.ent A;1486 4a25.ent B 5mdh.ent A;1487 4a25.ent B 3two.ent B;1488 4a25.ent B 3n9s.ent A;1489 4a25.ent B 3exe.ent B;1490 4a25.ent B 2yy7.ent A;1491 4a25.ent B 2qx3.ent A;1492 4a25.ent B 2hk2.ent A;1493 4a25.ent B 2eih.ent A;1494 4a25.ent B 1yg9.ent A;1495 4a25.ent B 1tca.ent A;1496 4a25.ent B 1pxz.ent A;1497 4a25.ent B 1llp.ent A;1498 4a25.ent B 1h6u.ent A;1499 4a25.ent B 1ea7.ent A;1500 4a25.ent B 1a4m.ent A;1501 4a25.ent B 4b5w.ent A;1502 4a25.ent B 3q1n.ent A;1503 4a25.ent B 3m66.ent A;1504 4a25.ent B 3gae.ent A;1505 4a25.ent B 3bcz.ent A;1506 4a25.ent B 2voa.ent A;1507 4a25.ent B 2p9h.ent A;1508 4a25.ent B 2gnu.ent M;1509 4a25.ent B 2ci1.ent A;1510 4a25.ent B 1xq6.ent A;1511 4a25.ent B 1tml.ent A;1512 4a25.ent B 1qgj.ent A;1513 4a25.ent B 1nas.ent A;1514 4a25.ent B 1hvq.ent A;1515 4a25.ent B 1fts.ent A;1516 4a25.ent B 1dea.ent A;1517 4a25.ent B 1b35.ent B;1518 4a25.ent B 1a0g.ent B;1519 4a25.ent B 3tt9.ent A;1520 4a25.ent B 3pnl.ent B;1521 4a25.ent B 3kvd.ent D;1522 4a25.ent B 3fj7.ent A;1523 4a25.ent B 3cjj.ent A;1524 4a25.ent B 3ai9.ent X;1525 4a25.ent B 2vg9.ent A;1526 4a25.ent B 2p0j.ent A;1527 4a25.ent B 2is9.ent A;1528 4a25.ent B 2eyi.ent A;1529 4a25.ent B 2cga.ent A;1530 4a25.ent B 1ym0.ent A;1531 4a25.ent B 1twf.ent E;1532 4a25.ent B 1rc9.ent A;1533 4a25.ent B 1pn9.ent A;1534 4a25.ent B 1n7k.ent A;1535 4a25.ent B 1kg5.ent A;1536 4a25.ent B 1gis.ent A;1537 4a25.ent B 1fuj.ent A;1538 4a25.ent B 1del.ent A;1539 4a25.ent B 1b77.ent A;1540 4a25.ent B 4h6a.ent A;1541 3q5v.ent A 5mdh.ent A;1542 3q5v.ent A 3two.ent B;1543 3q5v.ent A 3n9s.ent A;1544 3q5v.ent A 3exe.ent B;1545 3q5v.ent A 2yy7.ent A;1546 3q5v.ent A 2qx3.ent A;1547 3q5v.ent A 2hk2.ent A;1548 3q5v.ent A 2eih.ent A;1549 3q5v.ent A 1yg9.ent A;1550 3q5v.ent A 1tca.ent A;1551 3q5v.ent A 1pxz.ent A;1552 3q5v.ent A 1llp.ent A;1553 3q5v.ent A 1h6u.ent A;1554 3q5v.ent A 1ea7.ent A;1555 3q5v.ent A 1a4m.ent A;1556 3q5v.ent A 4b5w.ent A;1557 3q5v.ent A 3q1n.ent A;1558 3q5v.ent A 3m66.ent A;1559 3q5v.ent A 3gae.ent A;1560 3q5v.ent A 3bcz.ent A;1561 3q5v.ent A 2voa.ent A;1562 3q5v.ent A 2p9h.ent A;1563 3q5v.ent A 2gnu.ent M;1564 3q5v.ent A 2ci1.ent A;1565 3q5v.ent A 1xq6.ent A;1566 3q5v.ent A 1tml.ent A;1567 3q5v.ent A 1qgj.ent A;1568 3q5v.ent A 1nas.ent A;1569 3q5v.ent A 1hvq.ent A;1570 3q5v.ent A 1fts.ent A;1571 3q5v.ent A 1dea.ent A;1572 3q5v.ent A 1b35.ent B;1573 3q5v.ent A 1a0g.ent B;1574 3q5v.ent A 3tt9.ent A;1575 3q5v.ent A 3pnl.ent B;1576 3q5v.ent A 3kvd.ent D;1577 3q5v.ent A 3fj7.ent A;1578 3q5v.ent A 3cjj.ent A;1579 3q5v.ent A 3ai9.ent X;1580 3q5v.ent A 2vg9.ent A;1581 3q5v.ent A 2p0j.ent A;1582 3q5v.ent A 2is9.ent A;1583 3q5v.ent A 2eyi.ent A;1584 3q5v.ent A 2cga.ent A;1585 3q5v.ent A 1ym0.ent A;1586 3q5v.ent A 1twf.ent E;1587 3q5v.ent A 1rc9.ent A;1588 3q5v.ent A 1pn9.ent A;1589 3q5v.ent A 1n7k.ent A;1590 3q5v.ent A 1kg5.ent A;1591 3q5v.ent A 1gis.ent A;1592 3q5v.ent A 1fuj.ent A;1593 3q5v.ent A 1del.ent A;1594 3q5v.ent A 1b77.ent A;1595 3q5v.ent A 4h6a.ent A;1596 3q5v.ent A 4a25.ent B;1597 3nmr.ent A 5mdh.ent A;1598 3nmr.ent A 3two.ent B;1599 3nmr.ent A 3n9s.ent A;1600 3nmr.ent A 3exe.ent B;1601 3nmr.ent A 2yy7.ent A;1602 3nmr.ent A 2qx3.ent A;1603 3nmr.ent A 2hk2.ent A;1604 3nmr.ent A 2eih.ent A;1605 3nmr.ent A 1yg9.ent A;1606 3nmr.ent A 1tca.ent A;1607 3nmr.ent A 1pxz.ent A;1608 3nmr.ent A 1llp.ent A;1609 3nmr.ent A 1h6u.ent A;1610 3nmr.ent A 1ea7.ent A;1611 3nmr.ent A 1a4m.ent A;1612 3nmr.ent A 4b5w.ent A;1613 3nmr.ent A 3q1n.ent A;1614 3nmr.ent A 3m66.ent A;1615 3nmr.ent A 3gae.ent A;1616 3nmr.ent A 3bcz.ent A;1617 3nmr.ent A 2voa.ent A;1618 3nmr.ent A 2p9h.ent A;1619 3nmr.ent A 2gnu.ent M;1620 3nmr.ent A 2ci1.ent A;1621 3nmr.ent A 1xq6.ent A;1622 3nmr.ent A 1tml.ent A;1623 3nmr.ent A 1qgj.ent A;1624 3nmr.ent A 1nas.ent A;1625 3nmr.ent A 1hvq.ent A;1626 3nmr.ent A 1fts.ent A;1627 3nmr.ent A 1dea.ent A;1628 3nmr.ent A 1b35.ent B;1629 3nmr.ent A 1a0g.ent B;1630 3nmr.ent A 3tt9.ent A;1631 3nmr.ent A 3pnl.ent B;1632 3nmr.ent A 3kvd.ent D;1633 3nmr.ent A 3fj7.ent A;1634 3nmr.ent A 3cjj.ent A;1635 3nmr.ent A 3ai9.ent X;1636 3nmr.ent A 2vg9.ent A;1637 3nmr.ent A 2p0j.ent A;1638 3nmr.ent A 2is9.ent A;1639 3nmr.ent A 2eyi.ent A;1640 3nmr.ent A 2cga.ent A;1641 3nmr.ent A 1ym0.ent A;1642 3nmr.ent A 1twf.ent E;1643 3nmr.ent A 1rc9.ent A;1644 3nmr.ent A 1pn9.ent A;1645 3nmr.ent A 1n7k.ent A;1646 3nmr.ent A 1kg5.ent A;1647 3nmr.ent A 1gis.ent A;1648 3nmr.ent A 1fuj.ent A;1649 3nmr.ent A 1del.ent A;1650 3nmr.ent A 1b77.ent A;1651 3nmr.ent A 4h6a.ent A;1652 3nmr.ent A 4a25.ent B;1653 3nmr.ent A 3q5v.ent A;1654 3kjz.ent A 5mdh.ent A;1655 3kjz.ent A 3two.ent B;1656 3kjz.ent A 3n9s.ent A;1657 3kjz.ent A 3exe.ent B;1658 3kjz.ent A 2yy7.ent A;1659 3kjz.ent A 2qx3.ent A;1660 3kjz.ent A 2hk2.ent A;1661 3kjz.ent A 2eih.ent A;1662 3kjz.ent A 1yg9.ent A;1663 3kjz.ent A 1tca.ent A;1664 3kjz.ent A 1pxz.ent A;1665 3kjz.ent A 1llp.ent A;1666 3kjz.ent A 1h6u.ent A;1667 3kjz.ent A 1ea7.ent A;1668 3kjz.ent A 1a4m.ent A;1669 3kjz.ent A 4b5w.ent A;1670 3kjz.ent A 3q1n.ent A;1671 3kjz.ent A 3m66.ent A;1672 3kjz.ent A 3gae.ent A;1673 3kjz.ent A 3bcz.ent A;1674 3kjz.ent A 2voa.ent A;1675 3kjz.ent A 2p9h.ent A;1676 3kjz.ent A 2gnu.ent M;1677 3kjz.ent A 2ci1.ent A;1678 3kjz.ent A 1xq6.ent A;1679 3kjz.ent A 1tml.ent A;1680 3kjz.ent A 1qgj.ent A;1681 3kjz.ent A 1nas.ent A;1682 3kjz.ent A 1hvq.ent A;1683 3kjz.ent A 1fts.ent A;1684 3kjz.ent A 1dea.ent A;1685 3kjz.ent A 1b35.ent B;1686 3kjz.ent A 1a0g.ent B;1687 3kjz.ent A 3tt9.ent A;1688 3kjz.ent A 3pnl.ent B;1689 3kjz.ent A 3kvd.ent D;1690 3kjz.ent A 3fj7.ent A;1691 3kjz.ent A 3cjj.ent A;1692 3kjz.ent A 3ai9.ent X;1693 3kjz.ent A 2vg9.ent A;1694 3kjz.ent A 2p0j.ent A;1695 3kjz.ent A 2is9.ent A;1696 3kjz.ent A 2eyi.ent A;1697 3kjz.ent A 2cga.ent A;1698 3kjz.ent A 1ym0.ent A;1699 3kjz.ent A 1twf.ent E;1700 3kjz.ent A 1rc9.ent A;1701 3kjz.ent A 1pn9.ent A;1702 3kjz.ent A 1n7k.ent A;1703 3kjz.ent A 1kg5.ent A;1704 3kjz.ent A 1gis.ent A;1705 3kjz.ent A 1fuj.ent A;1706 3kjz.ent A 1del.ent A;1707 3kjz.ent A 1b77.ent A;1708 3kjz.ent A 4h6a.ent A;1709 3kjz.ent A 4a25.ent B;1710 3kjz.ent A 3q5v.ent A;1711 3kjz.ent A 3nmr.ent A;1712 3g5k.ent A 5mdh.ent A;1713 3g5k.ent A 3two.ent B;1714 3g5k.ent A 3n9s.ent A;1715 3g5k.ent A 3exe.ent B;1716 3g5k.ent A 2yy7.ent A;1717 3g5k.ent A 2qx3.ent A;1718 3g5k.ent A 2hk2.ent A;1719 3g5k.ent A 2eih.ent A;1720 3g5k.ent A 1yg9.ent A;1721 3g5k.ent A 1tca.ent A;1722 3g5k.ent A 1pxz.ent A;1723 3g5k.ent A 1llp.ent A;1724 3g5k.ent A 1h6u.ent A;1725 3g5k.ent A 1ea7.ent A;1726 3g5k.ent A 1a4m.ent A;1727 3g5k.ent A 4b5w.ent A;1728 3g5k.ent A 3q1n.ent A;1729 3g5k.ent A 3m66.ent A;1730 3g5k.ent A 3gae.ent A;1731 3g5k.ent A 3bcz.ent A;1732 3g5k.ent A 2voa.ent A;1733 3g5k.ent A 2p9h.ent A;1734 3g5k.ent A 2gnu.ent M;1735 3g5k.ent A 2ci1.ent A;1736 3g5k.ent A 1xq6.ent A;1737 3g5k.ent A 1tml.ent A;1738 3g5k.ent A 1qgj.ent A;1739 3g5k.ent A 1nas.ent A;1740 3g5k.ent A 1hvq.ent A;1741 3g5k.ent A 1fts.ent A;1742 3g5k.ent A 1dea.ent A;1743 3g5k.ent A 1b35.ent B;1744 3g5k.ent A 1a0g.ent B;1745 3g5k.ent A 3tt9.ent A;1746 3g5k.ent A 3pnl.ent B;1747 3g5k.ent A 3kvd.ent D;1748 3g5k.ent A 3fj7.ent A;1749 3g5k.ent A 3cjj.ent A;1750 3g5k.ent A 3ai9.ent X;1751 3g5k.ent A 2vg9.ent A;1752 3g5k.ent A 2p0j.ent A;1753 3g5k.ent A 2is9.ent A;1754 3g5k.ent A 2eyi.ent A;1755 3g5k.ent A 2cga.ent A;1756 3g5k.ent A 1ym0.ent A;1757 3g5k.ent A 1twf.ent E;1758 3g5k.ent A 1rc9.ent A;1759 3g5k.ent A 1pn9.ent A;1760 3g5k.ent A 1n7k.ent A;1761 3g5k.ent A 1kg5.ent A;1762 3g5k.ent A 1gis.ent A;1763 3g5k.ent A 1fuj.ent A;1764 3g5k.ent A 1del.ent A;1765 3g5k.ent A 1b77.ent A;1766 3g5k.ent A 4h6a.ent A;1767 3g5k.ent A 4a25.ent B;1768 3g5k.ent A 3q5v.ent A;1769 3g5k.ent A 3nmr.ent A;1770 3g5k.ent A 3kjz.ent A;1771 3csr.ent A 5mdh.ent A;1772 3csr.ent A 3two.ent B;1773 3csr.ent A 3n9s.ent A;1774 3csr.ent A 3exe.ent B;1775 3csr.ent A 2yy7.ent A;1776 3csr.ent A 2qx3.ent A;1777 3csr.ent A 2hk2.ent A;1778 3csr.ent A 2eih.ent A;1779 3csr.ent A 1yg9.ent A;1780 3csr.ent A 1tca.ent A;1781 3csr.ent A 1pxz.ent A;1782 3csr.ent A 1llp.ent A;1783 3csr.ent A 1h6u.ent A;1784 3csr.ent A 1ea7.ent A;1785 3csr.ent A 1a4m.ent A;1786 3csr.ent A 4b5w.ent A;1787 3csr.ent A 3q1n.ent A;1788 3csr.ent A 3m66.ent A;1789 3csr.ent A 3gae.ent A;1790 3csr.ent A 3bcz.ent A;1791 3csr.ent A 2voa.ent A;1792 3csr.ent A 2p9h.ent A;1793 3csr.ent A 2gnu.ent M;1794 3csr.ent A 2ci1.ent A;1795 3csr.ent A 1xq6.ent A;1796 3csr.ent A 1tml.ent A;1797 3csr.ent A 1qgj.ent A;1798 3csr.ent A 1nas.ent A;1799 3csr.ent A 1hvq.ent A;1800 3csr.ent A 1fts.ent A;1801 3csr.ent A 1dea.ent A;1802 3csr.ent A 1b35.ent B;1803 3csr.ent A 1a0g.ent B;1804 3csr.ent A 3tt9.ent A;1805 3csr.ent A 3pnl.ent B;1806 3csr.ent A 3kvd.ent D;1807 3csr.ent A 3fj7.ent A;1808 3csr.ent A 3cjj.ent A;1809 3csr.ent A 3ai9.ent X;1810 3csr.ent A 2vg9.ent A;1811 3csr.ent A 2p0j.ent A;1812 3csr.ent A 2is9.ent A;1813 3csr.ent A 2eyi.ent A;1814 3csr.ent A 2cga.ent A;1815 3csr.ent A 1ym0.ent A;1816 3csr.ent A 1twf.ent E;1817 3csr.ent A 1rc9.ent A;1818 3csr.ent A 1pn9.ent A;1819 3csr.ent A 1n7k.ent A;1820 3csr.ent A 1kg5.ent A;1821 3csr.ent A 1gis.ent A;1822 3csr.ent A 1fuj.ent A;1823 3csr.ent A 1del.ent A;1824 3csr.ent A 1b77.ent A;1825 3csr.ent A 4h6a.ent A;1826 3csr.ent A 4a25.ent B;1827 3csr.ent A 3q5v.ent A;1828 3csr.ent A 3nmr.ent A;1829 3csr.ent A 3kjz.ent A;1830 3csr.ent A 3g5k.ent A;1831 2zk9.ent X 5mdh.ent A;1832 2zk9.ent X 3two.ent B;1833 2zk9.ent X 3n9s.ent A;1834 2zk9.ent X 3exe.ent B;1835 2zk9.ent X 2yy7.ent A;1836 2zk9.ent X 2qx3.ent A;1837 2zk9.ent X 2hk2.ent A;1838 2zk9.ent X 2eih.ent A;1839 2zk9.ent X 1yg9.ent A;1840 2zk9.ent X 1tca.ent A;1841 2zk9.ent X 1pxz.ent A;1842 2zk9.ent X 1llp.ent A;1843 2zk9.ent X 1h6u.ent A;1844 2zk9.ent X 1ea7.ent A;1845 2zk9.ent X 1a4m.ent A;1846 2zk9.ent X 4b5w.ent A;1847 2zk9.ent X 3q1n.ent A;1848 2zk9.ent X 3m66.ent A;1849 2zk9.ent X 3gae.ent A;1850 2zk9.ent X 3bcz.ent A;1851 2zk9.ent X 2voa.ent A;1852 2zk9.ent X 2p9h.ent A;1853 2zk9.ent X 2gnu.ent M;1854 2zk9.ent X 2ci1.ent A;1855 2zk9.ent X 1xq6.ent A;1856 2zk9.ent X 1tml.ent A;1857 2zk9.ent X 1qgj.ent A;1858 2zk9.ent X 1nas.ent A;1859 2zk9.ent X 1hvq.ent A;1860 2zk9.ent X 1fts.ent A;1861 2zk9.ent X 1dea.ent A;1862 2zk9.ent X 1b35.ent B;1863 2zk9.ent X 1a0g.ent B;1864 2zk9.ent X 3tt9.ent A;1865 2zk9.ent X 3pnl.ent B;1866 2zk9.ent X 3kvd.ent D;1867 2zk9.ent X 3fj7.ent A;1868 2zk9.ent X 3cjj.ent A;1869 2zk9.ent X 3ai9.ent X;1870 2zk9.ent X 2vg9.ent A;1871 2zk9.ent X 2p0j.ent A;1872 2zk9.ent X 2is9.ent A;1873 2zk9.ent X 2eyi.ent A;1874 2zk9.ent X 2cga.ent A;1875 2zk9.ent X 1ym0.ent A;1876 2zk9.ent X 1twf.ent E;1877 2zk9.ent X 1rc9.ent A;1878 2zk9.ent X 1pn9.ent A;1879 2zk9.ent X 1n7k.ent A;1880 2zk9.ent X 1kg5.ent A;1881 2zk9.ent X 1gis.ent A;1882 2zk9.ent X 1fuj.ent A;1883 2zk9.ent X 1del.ent A;1884 2zk9.ent X 1b77.ent A;1885 2zk9.ent X 4h6a.ent A;1886 2zk9.ent X 4a25.ent B;1887 2zk9.ent X 3q5v.ent A;1888 2zk9.ent X 3nmr.ent A;1889 2zk9.ent X 3kjz.ent A;1890 2zk9.ent X 3g5k.ent A;1891 2zk9.ent X 3csr.ent A;1892 2wmm.ent A 5mdh.ent A;1893 2wmm.ent A 3two.ent B;1894 2wmm.ent A 3n9s.ent A;1895 2wmm.ent A 3exe.ent B;1896 2wmm.ent A 2yy7.ent A;1897 2wmm.ent A 2qx3.ent A;1898 2wmm.ent A 2hk2.ent A;1899 2wmm.ent A 2eih.ent A;1900 2wmm.ent A 1yg9.ent A;1901 2wmm.ent A 1tca.ent A;1902 2wmm.ent A 1pxz.ent A;1903 2wmm.ent A 1llp.ent A;1904 2wmm.ent A 1h6u.ent A;1905 2wmm.ent A 1ea7.ent A;1906 2wmm.ent A 1a4m.ent A;1907 2wmm.ent A 4b5w.ent A;1908 2wmm.ent A 3q1n.ent A;1909 2wmm.ent A 3m66.ent A;1910 2wmm.ent A 3gae.ent A;1911 2wmm.ent A 3bcz.ent A;1912 2wmm.ent A 2voa.ent A;1913 2wmm.ent A 2p9h.ent A;1914 2wmm.ent A 2gnu.ent M;1915 2wmm.ent A 2ci1.ent A;1916 2wmm.ent A 1xq6.ent A;1917 2wmm.ent A 1tml.ent A;1918 2wmm.ent A 1qgj.ent A;1919 2wmm.ent A 1nas.ent A;1920 2wmm.ent A 1hvq.ent A;1921 2wmm.ent A 1fts.ent A;1922 2wmm.ent A 1dea.ent A;1923 2wmm.ent A 1b35.ent B;1924 2wmm.ent A 1a0g.ent B;1925 2wmm.ent A 3tt9.ent A;1926 2wmm.ent A 3pnl.ent B;1927 2wmm.ent A 3kvd.ent D;1928 2wmm.ent A 3fj7.ent A;1929 2wmm.ent A 3cjj.ent A;1930 2wmm.ent A 3ai9.ent X;1931 2wmm.ent A 2vg9.ent A;1932 2wmm.ent A 2p0j.ent A;1933 2wmm.ent A 2is9.ent A;1934 2wmm.ent A 2eyi.ent A;1935 2wmm.ent A 2cga.ent A;1936 2wmm.ent A 1ym0.ent A;1937 2wmm.ent A 1twf.ent E;1938 2wmm.ent A 1rc9.ent A;1939 2wmm.ent A 1pn9.ent A;1940 2wmm.ent A 1n7k.ent A;1941 2wmm.ent A 1kg5.ent A;1942 2wmm.ent A 1gis.ent A;1943 2wmm.ent A 1fuj.ent A;1944 2wmm.ent A 1del.ent A;1945 2wmm.ent A 1b77.ent A;1946 2wmm.ent A 4h6a.ent A;1947 2wmm.ent A 4a25.ent B;1948 2wmm.ent A 3q5v.ent A;1949 2wmm.ent A 3nmr.ent A;1950 2wmm.ent A 3kjz.ent A;1951 2wmm.ent A 3g5k.ent A;1952 2wmm.ent A 3csr.ent A;1953 2wmm.ent A 2zk9.ent X;1954 2q7a.ent A 5mdh.ent A;1955 2q7a.ent A 3two.ent B;1956 2q7a.ent A 3n9s.ent A;1957 2q7a.ent A 3exe.ent B;1958 2q7a.ent A 2yy7.ent A;1959 2q7a.ent A 2qx3.ent A;1960 2q7a.ent A 2hk2.ent A;1961 2q7a.ent A 2eih.ent A;1962 2q7a.ent A 1yg9.ent A;1963 2q7a.ent A 1tca.ent A;1964 2q7a.ent A 1pxz.ent A;1965 2q7a.ent A 1llp.ent A;1966 2q7a.ent A 1h6u.ent A;1967 2q7a.ent A 1ea7.ent A;1968 2q7a.ent A 1a4m.ent A;1969 2q7a.ent A 4b5w.ent A;1970 2q7a.ent A 3q1n.ent A;1971 2q7a.ent A 3m66.ent A;1972 2q7a.ent A 3gae.ent A;1973 2q7a.ent A 3bcz.ent A;1974 2q7a.ent A 2voa.ent A;1975 2q7a.ent A 2p9h.ent A;1976 2q7a.ent A 2gnu.ent M;1977 2q7a.ent A 2ci1.ent A;1978 2q7a.ent A 1xq6.ent A;1979 2q7a.ent A 1tml.ent A;1980 2q7a.ent A 1qgj.ent A;1981 2q7a.ent A 1nas.ent A;1982 2q7a.ent A 1hvq.ent A;1983 2q7a.ent A 1fts.ent A;1984 2q7a.ent A 1dea.ent A;1985 2q7a.ent A 1b35.ent B;1986 2q7a.ent A 1a0g.ent B;1987 2q7a.ent A 3tt9.ent A;1988 2q7a.ent A 3pnl.ent B;1989 2q7a.ent A 3kvd.ent D;1990 2q7a.ent A 3fj7.ent A;1991 2q7a.ent A 3cjj.ent A;1992 2q7a.ent A 3ai9.ent X;1993 2q7a.ent A 2vg9.ent A;1994 2q7a.ent A 2p0j.ent A;1995 2q7a.ent A 2is9.ent A;1996 2q7a.ent A 2eyi.ent A;1997 2q7a.ent A 2cga.ent A;1998 2q7a.ent A 1ym0.ent A;1999 2q7a.ent A 1twf.ent E;2000 2q7a.ent A 1rc9.ent A;2001 2q7a.ent A 1pn9.ent A;2002 2q7a.ent A 1n7k.ent A;2003 2q7a.ent A 1kg5.ent A;2004 2q7a.ent A 1gis.ent A;2005 2q7a.ent A 1fuj.ent A;2006 2q7a.ent A 1del.ent A;2007 2q7a.ent A 1b77.ent A;2008 2q7a.ent A 4h6a.ent A;2009 2q7a.ent A 4a25.ent B;2010 2q7a.ent A 3q5v.ent A;2011 2q7a.ent A 3nmr.ent A;2012 2q7a.ent A 3kjz.ent A;2013 2q7a.ent A 3g5k.ent A;2014 2q7a.ent A 3csr.ent A;2015 2q7a.ent A 2zk9.ent X;2016 2q7a.ent A 2wmm.ent A;2017 2ill.ent A 5mdh.ent A;2018 2ill.ent A 3two.ent B;2019 2ill.ent A 3n9s.ent A;2020 2ill.ent A 3exe.ent B;2021 2ill.ent A 2yy7.ent A;2022 2ill.ent A 2qx3.ent A;2023 2ill.ent A 2hk2.ent A;2024 2ill.ent A 2eih.ent A;2025 2ill.ent A 1yg9.ent A;2026 2ill.ent A 1tca.ent A;2027 2ill.ent A 1pxz.ent A;2028 2ill.ent A 1llp.ent A;2029 2ill.ent A 1h6u.ent A;2030 2ill.ent A 1ea7.ent A;2031 2ill.ent A 1a4m.ent A;2032 2ill.ent A 4b5w.ent A;2033 2ill.ent A 3q1n.ent A;2034 2ill.ent A 3m66.ent A;2035 2ill.ent A 3gae.ent A;2036 2ill.ent A 3bcz.ent A;2037 2ill.ent A 2voa.ent A;2038 2ill.ent A 2p9h.ent A;2039 2ill.ent A 2gnu.ent M;2040 2ill.ent A 2ci1.ent A;2041 2ill.ent A 1xq6.ent A;2042 2ill.ent A 1tml.ent A;2043 2ill.ent A 1qgj.ent A;2044 2ill.ent A 1nas.ent A;2045 2ill.ent A 1hvq.ent A;2046 2ill.ent A 1fts.ent A;2047 2ill.ent A 1dea.ent A;2048 2ill.ent A 1b35.ent B;2049 2ill.ent A 1a0g.ent B;2050 2ill.ent A 3tt9.ent A;2051 2ill.ent A 3pnl.ent B;2052 2ill.ent A 3kvd.ent D;2053 2ill.ent A 3fj7.ent A;2054 2ill.ent A 3cjj.ent A;2055 2ill.ent A 3ai9.ent X;2056 2ill.ent A 2vg9.ent A;2057 2ill.ent A 2p0j.ent A;2058 2ill.ent A 2is9.ent A;2059 2ill.ent A 2eyi.ent A;2060 2ill.ent A 2cga.ent A;2061 2ill.ent A 1ym0.ent A;2062 2ill.ent A 1twf.ent E;2063 2ill.ent A 1rc9.ent A;2064 2ill.ent A 1pn9.ent A;2065 2ill.ent A 1n7k.ent A;2066 2ill.ent A 1kg5.ent A;2067 2ill.ent A 1gis.ent A;2068 2ill.ent A 1fuj.ent A;2069 2ill.ent A 1del.ent A;2070 2ill.ent A 1b77.ent A;2071 2ill.ent A 4h6a.ent A;2072 2ill.ent A 4a25.ent B;2073 2ill.ent A 3q5v.ent A;2074 2ill.ent A 3nmr.ent A;2075 2ill.ent A 3kjz.ent A;2076 2ill.ent A 3g5k.ent A;2077 2ill.ent A 3csr.ent A;2078 2ill.ent A 2zk9.ent X;2079 2ill.ent A 2wmm.ent A;2080 2ill.ent A 2q7a.ent A;2081 2fko.ent A 5mdh.ent A;2082 2fko.ent A 3two.ent B;2083 2fko.ent A 3n9s.ent A;2084 2fko.ent A 3exe.ent B;2085 2fko.ent A 2yy7.ent A;2086 2fko.ent A 2qx3.ent A;2087 2fko.ent A 2hk2.ent A;2088 2fko.ent A 2eih.ent A;2089 2fko.ent A 1yg9.ent A;2090 2fko.ent A 1tca.ent A;2091 2fko.ent A 1pxz.ent A;2092 2fko.ent A 1llp.ent A;2093 2fko.ent A 1h6u.ent A;2094 2fko.ent A 1ea7.ent A;2095 2fko.ent A 1a4m.ent A;2096 2fko.ent A 4b5w.ent A;2097 2fko.ent A 3q1n.ent A;2098 2fko.ent A 3m66.ent A;2099 2fko.ent A 3gae.ent A;2100 2fko.ent A 3bcz.ent A;2101 2fko.ent A 2voa.ent A;2102 2fko.ent A 2p9h.ent A;2103 2fko.ent A 2gnu.ent M;2104 2fko.ent A 2ci1.ent A;2105 2fko.ent A 1xq6.ent A;2106 2fko.ent A 1tml.ent A;2107 2fko.ent A 1qgj.ent A;2108 2fko.ent A 1nas.ent A;2109 2fko.ent A 1hvq.ent A;2110 2fko.ent A 1fts.ent A;2111 2fko.ent A 1dea.ent A;2112 2fko.ent A 1b35.ent B;2113 2fko.ent A 1a0g.ent B;2114 2fko.ent A 3tt9.ent A;2115 2fko.ent A 3pnl.ent B;2116 2fko.ent A 3kvd.ent D;2117 2fko.ent A 3fj7.ent A;2118 2fko.ent A 3cjj.ent A;2119 2fko.ent A 3ai9.ent X;2120 2fko.ent A 2vg9.ent A;2121 2fko.ent A 2p0j.ent A;2122 2fko.ent A 2is9.ent A;2123 2fko.ent A 2eyi.ent A;2124 2fko.ent A 2cga.ent A;2125 2fko.ent A 1ym0.ent A;2126 2fko.ent A 1twf.ent E;2127 2fko.ent A 1rc9.ent A;2128 2fko.ent A 1pn9.ent A;2129 2fko.ent A 1n7k.ent A;2130 2fko.ent A 1kg5.ent A;2131 2fko.ent A 1gis.ent A;2132 2fko.ent A 1fuj.ent A;2133 2fko.ent A 1del.ent A;2134 2fko.ent A 1b77.ent A;2135 2fko.ent A 4h6a.ent A;2136 2fko.ent A 4a25.ent B;2137 2fko.ent A 3q5v.ent A;2138 2fko.ent A 3nmr.ent A;2139 2fko.ent A 3kjz.ent A;2140 2fko.ent A 3g5k.ent A;2141 2fko.ent A 3csr.ent A;2142 2fko.ent A 2zk9.ent X;2143 2fko.ent A 2wmm.ent A;2144 2fko.ent A 2q7a.ent A;2145 2fko.ent A 2ill.ent A;2146 2cut.ent A 5mdh.ent A;2147 2cut.ent A 3two.ent B;2148 2cut.ent A 3n9s.ent A;2149 2cut.ent A 3exe.ent B;2150 2cut.ent A 2yy7.ent A;2151 2cut.ent A 2qx3.ent A;2152 2cut.ent A 2hk2.ent A;2153 2cut.ent A 2eih.ent A;2154 2cut.ent A 1yg9.ent A;2155 2cut.ent A 1tca.ent A;2156 2cut.ent A 1pxz.ent A;2157 2cut.ent A 1llp.ent A;2158 2cut.ent A 1h6u.ent A;2159 2cut.ent A 1ea7.ent A;2160 2cut.ent A 1a4m.ent A;2161 2cut.ent A 4b5w.ent A;2162 2cut.ent A 3q1n.ent A;2163 2cut.ent A 3m66.ent A;2164 2cut.ent A 3gae.ent A;2165 2cut.ent A 3bcz.ent A;2166 2cut.ent A 2voa.ent A;2167 2cut.ent A 2p9h.ent A;2168 2cut.ent A 2gnu.ent M;2169 2cut.ent A 2ci1.ent A;2170 2cut.ent A 1xq6.ent A;2171 2cut.ent A 1tml.ent A;2172 2cut.ent A 1qgj.ent A;2173 2cut.ent A 1nas.ent A;2174 2cut.ent A 1hvq.ent A;2175 2cut.ent A 1fts.ent A;2176 2cut.ent A 1dea.ent A;2177 2cut.ent A 1b35.ent B;2178 2cut.ent A 1a0g.ent B;2179 2cut.ent A 3tt9.ent A;2180 2cut.ent A 3pnl.ent B;2181 2cut.ent A 3kvd.ent D;2182 2cut.ent A 3fj7.ent A;2183 2cut.ent A 3cjj.ent A;2184 2cut.ent A 3ai9.ent X;2185 2cut.ent A 2vg9.ent A;2186 2cut.ent A 2p0j.ent A;2187 2cut.ent A 2is9.ent A;2188 2cut.ent A 2eyi.ent A;2189 2cut.ent A 2cga.ent A;2190 2cut.ent A 1ym0.ent A;2191 2cut.ent A 1twf.ent E;2192 2cut.ent A 1rc9.ent A;2193 2cut.ent A 1pn9.ent A;2194 2cut.ent A 1n7k.ent A;2195 2cut.ent A 1kg5.ent A;2196 2cut.ent A 1gis.ent A;2197 2cut.ent A 1fuj.ent A;2198 2cut.ent A 1del.ent A;2199 2cut.ent A 1b77.ent A;2200 2cut.ent A 4h6a.ent A;2201 2cut.ent A 4a25.ent B;2202 2cut.ent A 3q5v.ent A;2203 2cut.ent A 3nmr.ent A;2204 2cut.ent A 3kjz.ent A;2205 2cut.ent A 3g5k.ent A;2206 2cut.ent A 3csr.ent A;2207 2cut.ent A 2zk9.ent X;2208 2cut.ent A 2wmm.ent A;2209 2cut.ent A 2q7a.ent A;2210 2cut.ent A 2ill.ent A;2211 2cut.ent A 2fko.ent A;2212 1zv9.ent A 5mdh.ent A;2213 1zv9.ent A 3two.ent B;2214 1zv9.ent A 3n9s.ent A;2215 1zv9.ent A 3exe.ent B;2216 1zv9.ent A 2yy7.ent A;2217 1zv9.ent A 2qx3.ent A;2218 1zv9.ent A 2hk2.ent A;2219 1zv9.ent A 2eih.ent A;2220 1zv9.ent A 1yg9.ent A;2221 1zv9.ent A 1tca.ent A;2222 1zv9.ent A 1pxz.ent A;2223 1zv9.ent A 1llp.ent A;2224 1zv9.ent A 1h6u.ent A;2225 1zv9.ent A 1ea7.ent A;2226 1zv9.ent A 1a4m.ent A;2227 1zv9.ent A 4b5w.ent A;2228 1zv9.ent A 3q1n.ent A;2229 1zv9.ent A 3m66.ent A;2230 1zv9.ent A 3gae.ent A;2231 1zv9.ent A 3bcz.ent A;2232 1zv9.ent A 2voa.ent A;2233 1zv9.ent A 2p9h.ent A;2234 1zv9.ent A 2gnu.ent M;2235 1zv9.ent A 2ci1.ent A;2236 1zv9.ent A 1xq6.ent A;2237 1zv9.ent A 1tml.ent A;2238 1zv9.ent A 1qgj.ent A;2239 1zv9.ent A 1nas.ent A;2240 1zv9.ent A 1hvq.ent A;2241 1zv9.ent A 1fts.ent A;2242 1zv9.ent A 1dea.ent A;2243 1zv9.ent A 1b35.ent B;2244 1zv9.ent A 1a0g.ent B;2245 1zv9.ent A 3tt9.ent A;2246 1zv9.ent A 3pnl.ent B;2247 1zv9.ent A 3kvd.ent D;2248 1zv9.ent A 3fj7.ent A;2249 1zv9.ent A 3cjj.ent A;2250 1zv9.ent A 3ai9.ent X;2251 1zv9.ent A 2vg9.ent A;2252 1zv9.ent A 2p0j.ent A;2253 1zv9.ent A 2is9.ent A;2254 1zv9.ent A 2eyi.ent A;2255 1zv9.ent A 2cga.ent A;2256 1zv9.ent A 1ym0.ent A;2257 1zv9.ent A 1twf.ent E;2258 1zv9.ent A 1rc9.ent A;2259 1zv9.ent A 1pn9.ent A;2260 1zv9.ent A 1n7k.ent A;2261 1zv9.ent A 1kg5.ent A;2262 1zv9.ent A 1gis.ent A;2263 1zv9.ent A 1fuj.ent A;2264 1zv9.ent A 1del.ent A;2265 1zv9.ent A 1b77.ent A;2266 1zv9.ent A 4h6a.ent A;2267 1zv9.ent A 4a25.ent B;2268 1zv9.ent A 3q5v.ent A;2269 1zv9.ent A 3nmr.ent A;2270 1zv9.ent A 3kjz.ent A;2271 1zv9.ent A 3g5k.ent A;2272 1zv9.ent A 3csr.ent A;2273 1zv9.ent A 2zk9.ent X;2274 1zv9.ent A 2wmm.ent A;2275 1zv9.ent A 2q7a.ent A;2276 1zv9.ent A 2ill.ent A;2277 1zv9.ent A 2fko.ent A;2278 1zv9.ent A 2cut.ent A;2279 1xt0.ent B 5mdh.ent A;2280 1xt0.ent B 3two.ent B;2281 1xt0.ent B 3n9s.ent A;2282 1xt0.ent B 3exe.ent B;2283 1xt0.ent B 2yy7.ent A;2284 1xt0.ent B 2qx3.ent A;2285 1xt0.ent B 2hk2.ent A;2286 1xt0.ent B 2eih.ent A;2287 1xt0.ent B 1yg9.ent A;2288 1xt0.ent B 1tca.ent A;2289 1xt0.ent B 1pxz.ent A;2290 1xt0.ent B 1llp.ent A;2291 1xt0.ent B 1h6u.ent A;2292 1xt0.ent B 1ea7.ent A;2293 1xt0.ent B 1a4m.ent A;2294 1xt0.ent B 4b5w.ent A;2295 1xt0.ent B 3q1n.ent A;2296 1xt0.ent B 3m66.ent A;2297 1xt0.ent B 3gae.ent A;2298 1xt0.ent B 3bcz.ent A;2299 1xt0.ent B 2voa.ent A;2300 1xt0.ent B 2p9h.ent A;2301 1xt0.ent B 2gnu.ent M;2302 1xt0.ent B 2ci1.ent A;2303 1xt0.ent B 1xq6.ent A;2304 1xt0.ent B 1tml.ent A;2305 1xt0.ent B 1qgj.ent A;2306 1xt0.ent B 1nas.ent A;2307 1xt0.ent B 1hvq.ent A;2308 1xt0.ent B 1fts.ent A;2309 1xt0.ent B 1dea.ent A;2310 1xt0.ent B 1b35.ent B;2311 1xt0.ent B 1a0g.ent B;2312 1xt0.ent B 3tt9.ent A;2313 1xt0.ent B 3pnl.ent B;2314 1xt0.ent B 3kvd.ent D;2315 1xt0.ent B 3fj7.ent A;2316 1xt0.ent B 3cjj.ent A;2317 1xt0.ent B 3ai9.ent X;2318 1xt0.ent B 2vg9.ent A;2319 1xt0.ent B 2p0j.ent A;2320 1xt0.ent B 2is9.ent A;2321 1xt0.ent B 2eyi.ent A;2322 1xt0.ent B 2cga.ent A;2323 1xt0.ent B 1ym0.ent A;2324 1xt0.ent B 1twf.ent E;2325 1xt0.ent B 1rc9.ent A;2326 1xt0.ent B 1pn9.ent A;2327 1xt0.ent B 1n7k.ent A;2328 1xt0.ent B 1kg5.ent A;2329 1xt0.ent B 1gis.ent A;2330 1xt0.ent B 1fuj.ent A;2331 1xt0.ent B 1del.ent A;2332 1xt0.ent B 1b77.ent A;2333 1xt0.ent B 4h6a.ent A;2334 1xt0.ent B 4a25.ent B;2335 1xt0.ent B 3q5v.ent A;2336 1xt0.ent B 3nmr.ent A;2337 1xt0.ent B 3kjz.ent A;2338 1xt0.ent B 3g5k.ent A;2339 1xt0.ent B 3csr.ent A;2340 1xt0.ent B 2zk9.ent X;2341 1xt0.ent B 2wmm.ent A;2342 1xt0.ent B 2q7a.ent A;2343 1xt0.ent B 2ill.ent A;2344 1xt0.ent B 2fko.ent A;2345 1xt0.ent B 2cut.ent A;2346 1xt0.ent B 1zv9.ent A;2347 1u7p.ent A 5mdh.ent A;2348 1u7p.ent A 3two.ent B;2349 1u7p.ent A 3n9s.ent A;2350 1u7p.ent A 3exe.ent B;2351 1u7p.ent A 2yy7.ent A;2352 1u7p.ent A 2qx3.ent A;2353 1u7p.ent A 2hk2.ent A;2354 1u7p.ent A 2eih.ent A;2355 1u7p.ent A 1yg9.ent A;2356 1u7p.ent A 1tca.ent A;2357 1u7p.ent A 1pxz.ent A;2358 1u7p.ent A 1llp.ent A;2359 1u7p.ent A 1h6u.ent A;2360 1u7p.ent A 1ea7.ent A;2361 1u7p.ent A 1a4m.ent A;2362 1u7p.ent A 4b5w.ent A;2363 1u7p.ent A 3q1n.ent A;2364 1u7p.ent A 3m66.ent A;2365 1u7p.ent A 3gae.ent A;2366 1u7p.ent A 3bcz.ent A;2367 1u7p.ent A 2voa.ent A;2368 1u7p.ent A 2p9h.ent A;2369 1u7p.ent A 2gnu.ent M;2370 1u7p.ent A 2ci1.ent A;2371 1u7p.ent A 1xq6.ent A;2372 1u7p.ent A 1tml.ent A;2373 1u7p.ent A 1qgj.ent A;2374 1u7p.ent A 1nas.ent A;2375 1u7p.ent A 1hvq.ent A;2376 1u7p.ent A 1fts.ent A;2377 1u7p.ent A 1dea.ent A;2378 1u7p.ent A 1b35.ent B;2379 1u7p.ent A 1a0g.ent B;2380 1u7p.ent A 3tt9.ent A;2381 1u7p.ent A 3pnl.ent B;2382 1u7p.ent A 3kvd.ent D;2383 1u7p.ent A 3fj7.ent A;2384 1u7p.ent A 3cjj.ent A;2385 1u7p.ent A 3ai9.ent X;2386 1u7p.ent A 2vg9.ent A;2387 1u7p.ent A 2p0j.ent A;2388 1u7p.ent A 2is9.ent A;2389 1u7p.ent A 2eyi.ent A;2390 1u7p.ent A 2cga.ent A;2391 1u7p.ent A 1ym0.ent A;2392 1u7p.ent A 1twf.ent E;2393 1u7p.ent A 1rc9.ent A;2394 1u7p.ent A 1pn9.ent A;2395 1u7p.ent A 1n7k.ent A;2396 1u7p.ent A 1kg5.ent A;2397 1u7p.ent A 1gis.ent A;2398 1u7p.ent A 1fuj.ent A;2399 1u7p.ent A 1del.ent A;2400 1u7p.ent A 1b77.ent A;2401 1u7p.ent A 4h6a.ent A;2402 1u7p.ent A 4a25.ent B;2403 1u7p.ent A 3q5v.ent A;2404 1u7p.ent A 3nmr.ent A;2405 1u7p.ent A 3kjz.ent A;2406 1u7p.ent A 3g5k.ent A;2407 1u7p.ent A 3csr.ent A;2408 1u7p.ent A 2zk9.ent X;2409 1u7p.ent A 2wmm.ent A;2410 1u7p.ent A 2q7a.ent A;2411 1u7p.ent A 2ill.ent A;2412 1u7p.ent A 2fko.ent A;2413 1u7p.ent A 2cut.ent A;2414 1u7p.ent A 1zv9.ent A;2415 1u7p.ent A 1xt0.ent B;2416 1r8n.ent A 5mdh.ent A;2417 1r8n.ent A 3two.ent B;2418 1r8n.ent A 3n9s.ent A;2419 1r8n.ent A 3exe.ent B;2420 1r8n.ent A 2yy7.ent A;2421 1r8n.ent A 2qx3.ent A;2422 1r8n.ent A 2hk2.ent A;2423 1r8n.ent A 2eih.ent A;2424 1r8n.ent A 1yg9.ent A;2425 1r8n.ent A 1tca.ent A;2426 1r8n.ent A 1pxz.ent A;2427 1r8n.ent A 1llp.ent A;2428 1r8n.ent A 1h6u.ent A;2429 1r8n.ent A 1ea7.ent A;2430 1r8n.ent A 1a4m.ent A;2431 1r8n.ent A 4b5w.ent A;2432 1r8n.ent A 3q1n.ent A;2433 1r8n.ent A 3m66.ent A;2434 1r8n.ent A 3gae.ent A;2435 1r8n.ent A 3bcz.ent A;2436 1r8n.ent A 2voa.ent A;2437 1r8n.ent A 2p9h.ent A;2438 1r8n.ent A 2gnu.ent M;2439 1r8n.ent A 2ci1.ent A;2440 1r8n.ent A 1xq6.ent A;2441 1r8n.ent A 1tml.ent A;2442 1r8n.ent A 1qgj.ent A;2443 1r8n.ent A 1nas.ent A;2444 1r8n.ent A 1hvq.ent A;2445 1r8n.ent A 1fts.ent A;2446 1r8n.ent A 1dea.ent A;2447 1r8n.ent A 1b35.ent B;2448 1r8n.ent A 1a0g.ent B;2449 1r8n.ent A 3tt9.ent A;2450 1r8n.ent A 3pnl.ent B;2451 1r8n.ent A 3kvd.ent D;2452 1r8n.ent A 3fj7.ent A;2453 1r8n.ent A 3cjj.ent A;2454 1r8n.ent A 3ai9.ent X;2455 1r8n.ent A 2vg9.ent A;2456 1r8n.ent A 2p0j.ent A;2457 1r8n.ent A 2is9.ent A;2458 1r8n.ent A 2eyi.ent A;2459 1r8n.ent A 2cga.ent A;2460 1r8n.ent A 1ym0.ent A;2461 1r8n.ent A 1twf.ent E;2462 1r8n.ent A 1rc9.ent A;2463 1r8n.ent A 1pn9.ent A;2464 1r8n.ent A 1n7k.ent A;2465 1r8n.ent A 1kg5.ent A;2466 1r8n.ent A 1gis.ent A;2467 1r8n.ent A 1fuj.ent A;2468 1r8n.ent A 1del.ent A;2469 1r8n.ent A 1b77.ent A;2470 1r8n.ent A 4h6a.ent A;2471 1r8n.ent A 4a25.ent B;2472 1r8n.ent A 3q5v.ent A;2473 1r8n.ent A 3nmr.ent A;2474 1r8n.ent A 3kjz.ent A;2475 1r8n.ent A 3g5k.ent A;2476 1r8n.ent A 3csr.ent A;2477 1r8n.ent A 2zk9.ent X;2478 1r8n.ent A 2wmm.ent A;2479 1r8n.ent A 2q7a.ent A;2480 1r8n.ent A 2ill.ent A;2481 1r8n.ent A 2fko.ent A;2482 1r8n.ent A 2cut.ent A;2483 1r8n.ent A 1zv9.ent A;2484 1r8n.ent A 1xt0.ent B;2485 1r8n.ent A 1u7p.ent A;2486 1ocy.ent A 5mdh.ent A;2487 1ocy.ent A 3two.ent B;2488 1ocy.ent A 3n9s.ent A;2489 1ocy.ent A 3exe.ent B;2490 1ocy.ent A 2yy7.ent A;2491 1ocy.ent A 2qx3.ent A;2492 1ocy.ent A 2hk2.ent A;2493 1ocy.ent A 2eih.ent A;2494 1ocy.ent A 1yg9.ent A;2495 1ocy.ent A 1tca.ent A;2496 1ocy.ent A 1pxz.ent A;2497 1ocy.ent A 1llp.ent A;2498 1ocy.ent A 1h6u.ent A;2499 1ocy.ent A 1ea7.ent A;2500 1ocy.ent A 1a4m.ent A;2501 1ocy.ent A 4b5w.ent A;2502 1ocy.ent A 3q1n.ent A;2503 1ocy.ent A 3m66.ent A;2504 1ocy.ent A 3gae.ent A;2505 1ocy.ent A 3bcz.ent A;2506 1ocy.ent A 2voa.ent A;2507 1ocy.ent A 2p9h.ent A;2508 1ocy.ent A 2gnu.ent M;2509 1ocy.ent A 2ci1.ent A;2510 1ocy.ent A 1xq6.ent A;2511 1ocy.ent A 1tml.ent A;2512 1ocy.ent A 1qgj.ent A;2513 1ocy.ent A 1nas.ent A;2514 1ocy.ent A 1hvq.ent A;2515 1ocy.ent A 1fts.ent A;2516 1ocy.ent A 1dea.ent A;2517 1ocy.ent A 1b35.ent B;2518 1ocy.ent A 1a0g.ent B;2519 1ocy.ent A 3tt9.ent A;2520 1ocy.ent A 3pnl.ent B;2521 1ocy.ent A 3kvd.ent D;2522 1ocy.ent A 3fj7.ent A;2523 1ocy.ent A 3cjj.ent A;2524 1ocy.ent A 3ai9.ent X;2525 1ocy.ent A 2vg9.ent A;2526 1ocy.ent A 2p0j.ent A;2527 1ocy.ent A 2is9.ent A;2528 1ocy.ent A 2eyi.ent A;2529 1ocy.ent A 2cga.ent A;2530 1ocy.ent A 1ym0.ent A;2531 1ocy.ent A 1twf.ent E;2532 1ocy.ent A 1rc9.ent A;2533 1ocy.ent A 1pn9.ent A;2534 1ocy.ent A 1n7k.ent A;2535 1ocy.ent A 1kg5.ent A;2536 1ocy.ent A 1gis.ent A;2537 1ocy.ent A 1fuj.ent A;2538 1ocy.ent A 1del.ent A;2539 1ocy.ent A 1b77.ent A;2540 1ocy.ent A 4h6a.ent A;2541 1ocy.ent A 4a25.ent B;2542 1ocy.ent A 3q5v.ent A;2543 1ocy.ent A 3nmr.ent A;2544 1ocy.ent A 3kjz.ent A;2545 1ocy.ent A 3g5k.ent A;2546 1ocy.ent A 3csr.ent A;2547 1ocy.ent A 2zk9.ent X;2548 1ocy.ent A 2wmm.ent A;2549 1ocy.ent A 2q7a.ent A;2550 1ocy.ent A 2ill.ent A;2551 1ocy.ent A 2fko.ent A;2552 1ocy.ent A 2cut.ent A;2553 1ocy.ent A 1zv9.ent A;2554 1ocy.ent A 1xt0.ent B;2555 1ocy.ent A 1u7p.ent A;2556 1ocy.ent A 1r8n.ent A;2557 1m4d.ent A 5mdh.ent A;2558 1m4d.ent A 3two.ent B;2559 1m4d.ent A 3n9s.ent A;2560 1m4d.ent A 3exe.ent B;2561 1m4d.ent A 2yy7.ent A;2562 1m4d.ent A 2qx3.ent A;2563 1m4d.ent A 2hk2.ent A;2564 1m4d.ent A 2eih.ent A;2565 1m4d.ent A 1yg9.ent A;2566 1m4d.ent A 1tca.ent A;2567 1m4d.ent A 1pxz.ent A;2568 1m4d.ent A 1llp.ent A;2569 1m4d.ent A 1h6u.ent A;2570 1m4d.ent A 1ea7.ent A;2571 1m4d.ent A 1a4m.ent A;2572 1m4d.ent A 4b5w.ent A;2573 1m4d.ent A 3q1n.ent A;2574 1m4d.ent A 3m66.ent A;2575 1m4d.ent A 3gae.ent A;2576 1m4d.ent A 3bcz.ent A;2577 1m4d.ent A 2voa.ent A;2578 1m4d.ent A 2p9h.ent A;2579 1m4d.ent A 2gnu.ent M;2580 1m4d.ent A 2ci1.ent A;2581 1m4d.ent A 1xq6.ent A;2582 1m4d.ent A 1tml.ent A;2583 1m4d.ent A 1qgj.ent A;2584 1m4d.ent A 1nas.ent A;2585 1m4d.ent A 1hvq.ent A;2586 1m4d.ent A 1fts.ent A;2587 1m4d.ent A 1dea.ent A;2588 1m4d.ent A 1b35.ent B;2589 1m4d.ent A 1a0g.ent B;2590 1m4d.ent A 3tt9.ent A;2591 1m4d.ent A 3pnl.ent B;2592 1m4d.ent A 3kvd.ent D;2593 1m4d.ent A 3fj7.ent A;2594 1m4d.ent A 3cjj.ent A;2595 1m4d.ent A 3ai9.ent X;2596 1m4d.ent A 2vg9.ent A;2597 1m4d.ent A 2p0j.ent A;2598 1m4d.ent A 2is9.ent A;2599 1m4d.ent A 2eyi.ent A;2600 1m4d.ent A 2cga.ent A;2601 1m4d.ent A 1ym0.ent A;2602 1m4d.ent A 1twf.ent E;2603 1m4d.ent A 1rc9.ent A;2604 1m4d.ent A 1pn9.ent A;2605 1m4d.ent A 1n7k.ent A;2606 1m4d.ent A 1kg5.ent A;2607 1m4d.ent A 1gis.ent A;2608 1m4d.ent A 1fuj.ent A;2609 1m4d.ent A 1del.ent A;2610 1m4d.ent A 1b77.ent A;2611 1m4d.ent A 4h6a.ent A;2612 1m4d.ent A 4a25.ent B;2613 1m4d.ent A 3q5v.ent A;2614 1m4d.ent A 3nmr.ent A;2615 1m4d.ent A 3kjz.ent A;2616 1m4d.ent A 3g5k.ent A;2617 1m4d.ent A 3csr.ent A;2618 1m4d.ent A 2zk9.ent X;2619 1m4d.ent A 2wmm.ent A;2620 1m4d.ent A 2q7a.ent A;2621 1m4d.ent A 2ill.ent A;2622 1m4d.ent A 2fko.ent A;2623 1m4d.ent A 2cut.ent A;2624 1m4d.ent A 1zv9.ent A;2625 1m4d.ent A 1xt0.ent B;2626 1m4d.ent A 1u7p.ent A;2627 1m4d.ent A 1r8n.ent A;2628 1m4d.ent A 1ocy.ent A;2629 1iqq.ent A 5mdh.ent A;2630 1iqq.ent A 3two.ent B;2631 1iqq.ent A 3n9s.ent A;2632 1iqq.ent A 3exe.ent B;2633 1iqq.ent A 2yy7.ent A;2634 1iqq.ent A 2qx3.ent A;2635 1iqq.ent A 2hk2.ent A;2636 1iqq.ent A 2eih.ent A;2637 1iqq.ent A 1yg9.ent A;2638 1iqq.ent A 1tca.ent A;2639 1iqq.ent A 1pxz.ent A;2640 1iqq.ent A 1llp.ent A;2641 1iqq.ent A 1h6u.ent A;2642 1iqq.ent A 1ea7.ent A;2643 1iqq.ent A 1a4m.ent A;2644 1iqq.ent A 4b5w.ent A;2645 1iqq.ent A 3q1n.ent A;2646 1iqq.ent A 3m66.ent A;2647 1iqq.ent A 3gae.ent A;2648 1iqq.ent A 3bcz.ent A;2649 1iqq.ent A 2voa.ent A;2650 1iqq.ent A 2p9h.ent A;2651 1iqq.ent A 2gnu.ent M;2652 1iqq.ent A 2ci1.ent A;2653 1iqq.ent A 1xq6.ent A;2654 1iqq.ent A 1tml.ent A;2655 1iqq.ent A 1qgj.ent A;2656 1iqq.ent A 1nas.ent A;2657 1iqq.ent A 1hvq.ent A;2658 1iqq.ent A 1fts.ent A;2659 1iqq.ent A 1dea.ent A;2660 1iqq.ent A 1b35.ent B;2661 1iqq.ent A 1a0g.ent B;2662 1iqq.ent A 3tt9.ent A;2663 1iqq.ent A 3pnl.ent B;2664 1iqq.ent A 3kvd.ent D;2665 1iqq.ent A 3fj7.ent A;2666 1iqq.ent A 3cjj.ent A;2667 1iqq.ent A 3ai9.ent X;2668 1iqq.ent A 2vg9.ent A;2669 1iqq.ent A 2p0j.ent A;2670 1iqq.ent A 2is9.ent A;2671 1iqq.ent A 2eyi.ent A;2672 1iqq.ent A 2cga.ent A;2673 1iqq.ent A 1ym0.ent A;2674 1iqq.ent A 1twf.ent E;2675 1iqq.ent A 1rc9.ent A;2676 1iqq.ent A 1pn9.ent A;2677 1iqq.ent A 1n7k.ent A;2678 1iqq.ent A 1kg5.ent A;2679 1iqq.ent A 1gis.ent A;2680 1iqq.ent A 1fuj.ent A;2681 1iqq.ent A 1del.ent A;2682 1iqq.ent A 1b77.ent A;2683 1iqq.ent A 4h6a.ent A;2684 1iqq.ent A 4a25.ent B;2685 1iqq.ent A 3q5v.ent A;2686 1iqq.ent A 3nmr.ent A;2687 1iqq.ent A 3kjz.ent A;2688 1iqq.ent A 3g5k.ent A;2689 1iqq.ent A 3csr.ent A;2690 1iqq.ent A 2zk9.ent X;2691 1iqq.ent A 2wmm.ent A;2692 1iqq.ent A 2q7a.ent A;2693 1iqq.ent A 2ill.ent A;2694 1iqq.ent A 2fko.ent A;2695 1iqq.ent A 2cut.ent A;2696 1iqq.ent A 1zv9.ent A;2697 1iqq.ent A 1xt0.ent B;2698 1iqq.ent A 1u7p.ent A;2699 1iqq.ent A 1r8n.ent A;2700 1iqq.ent A 1ocy.ent A;2701 1iqq.ent A 1m4d.ent A;2702 1gui.ent A 5mdh.ent A;2703 1gui.ent A 3two.ent B;2704 1gui.ent A 3n9s.ent A;2705 1gui.ent A 3exe.ent B;2706 1gui.ent A 2yy7.ent A;2707 1gui.ent A 2qx3.ent A;2708 1gui.ent A 2hk2.ent A;2709 1gui.ent A 2eih.ent A;2710 1gui.ent A 1yg9.ent A;2711 1gui.ent A 1tca.ent A;2712 1gui.ent A 1pxz.ent A;2713 1gui.ent A 1llp.ent A;2714 1gui.ent A 1h6u.ent A;2715 1gui.ent A 1ea7.ent A;2716 1gui.ent A 1a4m.ent A;2717 1gui.ent A 4b5w.ent A;2718 1gui.ent A 3q1n.ent A;2719 1gui.ent A 3m66.ent A;2720 1gui.ent A 3gae.ent A;2721 1gui.ent A 3bcz.ent A;2722 1gui.ent A 2voa.ent A;2723 1gui.ent A 2p9h.ent A;2724 1gui.ent A 2gnu.ent M;2725 1gui.ent A 2ci1.ent A;2726 1gui.ent A 1xq6.ent A;2727 1gui.ent A 1tml.ent A;2728 1gui.ent A 1qgj.ent A;2729 1gui.ent A 1nas.ent A;2730 1gui.ent A 1hvq.ent A;2731 1gui.ent A 1fts.ent A;2732 1gui.ent A 1dea.ent A;2733 1gui.ent A 1b35.ent B;2734 1gui.ent A 1a0g.ent B;2735 1gui.ent A 3tt9.ent A;2736 1gui.ent A 3pnl.ent B;2737 1gui.ent A 3kvd.ent D;2738 1gui.ent A 3fj7.ent A;2739 1gui.ent A 3cjj.ent A;2740 1gui.ent A 3ai9.ent X;2741 1gui.ent A 2vg9.ent A;2742 1gui.ent A 2p0j.ent A;2743 1gui.ent A 2is9.ent A;2744 1gui.ent A 2eyi.ent A;2745 1gui.ent A 2cga.ent A;2746 1gui.ent A 1ym0.ent A;2747 1gui.ent A 1twf.ent E;2748 1gui.ent A 1rc9.ent A;2749 1gui.ent A 1pn9.ent A;2750 1gui.ent A 1n7k.ent A;2751 1gui.ent A 1kg5.ent A;2752 1gui.ent A 1gis.ent A;2753 1gui.ent A 1fuj.ent A;2754 1gui.ent A 1del.ent A;2755 1gui.ent A 1b77.ent A;2756 1gui.ent A 4h6a.ent A;2757 1gui.ent A 4a25.ent B;2758 1gui.ent A 3q5v.ent A;2759 1gui.ent A 3nmr.ent A;2760 1gui.ent A 3kjz.ent A;2761 1gui.ent A 3g5k.ent A;2762 1gui.ent A 3csr.ent A;2763 1gui.ent A 2zk9.ent X;2764 1gui.ent A 2wmm.ent A;2765 1gui.ent A 2q7a.ent A;2766 1gui.ent A 2ill.ent A;2767 1gui.ent A 2fko.ent A;2768 1gui.ent A 2cut.ent A;2769 1gui.ent A 1zv9.ent A;2770 1gui.ent A 1xt0.ent B;2771 1gui.ent A 1u7p.ent A;2772 1gui.ent A 1r8n.ent A;2773 1gui.ent A 1ocy.ent A;2774 1gui.ent A 1m4d.ent A;2775 1gui.ent A 1iqq.ent A;2776 1ffk.ent T 5mdh.ent A;2777 1ffk.ent T 3two.ent B;2778 1ffk.ent T 3n9s.ent A;2779 1ffk.ent T 3exe.ent B;2780 1ffk.ent T 2yy7.ent A;2781 1ffk.ent T 2qx3.ent A;2782 1ffk.ent T 2hk2.ent A;2783 1ffk.ent T 2eih.ent A;2784 1ffk.ent T 1yg9.ent A;2785 1ffk.ent T 1tca.ent A;2786 1ffk.ent T 1pxz.ent A;2787 1ffk.ent T 1llp.ent A;2788 1ffk.ent T 1h6u.ent A;2789 1ffk.ent T 1ea7.ent A;2790 1ffk.ent T 1a4m.ent A;2791 1ffk.ent T 4b5w.ent A;2792 1ffk.ent T 3q1n.ent A;2793 1ffk.ent T 3m66.ent A;2794 1ffk.ent T 3gae.ent A;2795 1ffk.ent T 3bcz.ent A;2796 1ffk.ent T 2voa.ent A;2797 1ffk.ent T 2p9h.ent A;2798 1ffk.ent T 2gnu.ent M;2799 1ffk.ent T 2ci1.ent A;2800 1ffk.ent T 1xq6.ent A;2801 1ffk.ent T 1tml.ent A;2802 1ffk.ent T 1qgj.ent A;2803 1ffk.ent T 1nas.ent A;2804 1ffk.ent T 1hvq.ent A;2805 1ffk.ent T 1fts.ent A;2806 1ffk.ent T 1dea.ent A;2807 1ffk.ent T 1b35.ent B;2808 1ffk.ent T 1a0g.ent B;2809 1ffk.ent T 3tt9.ent A;2810 1ffk.ent T 3pnl.ent B;2811 1ffk.ent T 3kvd.ent D;2812 1ffk.ent T 3fj7.ent A;2813 1ffk.ent T 3cjj.ent A;2814 1ffk.ent T 3ai9.ent X;2815 1ffk.ent T 2vg9.ent A;2816 1ffk.ent T 2p0j.ent A;2817 1ffk.ent T 2is9.ent A;2818 1ffk.ent T 2eyi.ent A;2819 1ffk.ent T 2cga.ent A;2820 1ffk.ent T 1ym0.ent A;2821 1ffk.ent T 1twf.ent E;2822 1ffk.ent T 1rc9.ent A;2823 1ffk.ent T 1pn9.ent A;2824 1ffk.ent T 1n7k.ent A;2825 1ffk.ent T 1kg5.ent A;2826 1ffk.ent T 1gis.ent A;2827 1ffk.ent T 1fuj.ent A;2828 1ffk.ent T 1del.ent A;2829 1ffk.ent T 1b77.ent A;2830 1ffk.ent T 4h6a.ent A;2831 1ffk.ent T 4a25.ent B;2832 1ffk.ent T 3q5v.ent A;2833 1ffk.ent T 3nmr.ent A;2834 1ffk.ent T 3kjz.ent A;2835 1ffk.ent T 3g5k.ent A;2836 1ffk.ent T 3csr.ent A;2837 1ffk.ent T 2zk9.ent X;2838 1ffk.ent T 2wmm.ent A;2839 1ffk.ent T 2q7a.ent A;2840 1ffk.ent T 2ill.ent A;2841 1ffk.ent T 2fko.ent A;2842 1ffk.ent T 2cut.ent A;2843 1ffk.ent T 1zv9.ent A;2844 1ffk.ent T 1xt0.ent B;2845 1ffk.ent T 1u7p.ent A;2846 1ffk.ent T 1r8n.ent A;2847 1ffk.ent T 1ocy.ent A;2848 1ffk.ent T 1m4d.ent A;2849 1ffk.ent T 1iqq.ent A;2850 1ffk.ent T 1gui.ent A;2851 1dow.ent A 5mdh.ent A;2852 1dow.ent A 3two.ent B;2853 1dow.ent A 3n9s.ent A;2854 1dow.ent A 3exe.ent B;2855 1dow.ent A 2yy7.ent A;2856 1dow.ent A 2qx3.ent A;2857 1dow.ent A 2hk2.ent A;2858 1dow.ent A 2eih.ent A;2859 1dow.ent A 1yg9.ent A;2860 1dow.ent A 1tca.ent A;2861 1dow.ent A 1pxz.ent A;2862 1dow.ent A 1llp.ent A;2863 1dow.ent A 1h6u.ent A;2864 1dow.ent A 1ea7.ent A;2865 1dow.ent A 1a4m.ent A;2866 1dow.ent A 4b5w.ent A;2867 1dow.ent A 3q1n.ent A;2868 1dow.ent A 3m66.ent A;2869 1dow.ent A 3gae.ent A;2870 1dow.ent A 3bcz.ent A;2871 1dow.ent A 2voa.ent A;2872 1dow.ent A 2p9h.ent A;2873 1dow.ent A 2gnu.ent M;2874 1dow.ent A 2ci1.ent A;2875 1dow.ent A 1xq6.ent A;2876 1dow.ent A 1tml.ent A;2877 1dow.ent A 1qgj.ent A;2878 1dow.ent A 1nas.ent A;2879 1dow.ent A 1hvq.ent A;2880 1dow.ent A 1fts.ent A;2881 1dow.ent A 1dea.ent A;2882 1dow.ent A 1b35.ent B;2883 1dow.ent A 1a0g.ent B;2884 1dow.ent A 3tt9.ent A;2885 1dow.ent A 3pnl.ent B;2886 1dow.ent A 3kvd.ent D;2887 1dow.ent A 3fj7.ent A;2888 1dow.ent A 3cjj.ent A;2889 1dow.ent A 3ai9.ent X;2890 1dow.ent A 2vg9.ent A;2891 1dow.ent A 2p0j.ent A;2892 1dow.ent A 2is9.ent A;2893 1dow.ent A 2eyi.ent A;2894 1dow.ent A 2cga.ent A;2895 1dow.ent A 1ym0.ent A;2896 1dow.ent A 1twf.ent E;2897 1dow.ent A 1rc9.ent A;2898 1dow.ent A 1pn9.ent A;2899 1dow.ent A 1n7k.ent A;2900 1dow.ent A 1kg5.ent A;2901 1dow.ent A 1gis.ent A;2902 1dow.ent A 1fuj.ent A;2903 1dow.ent A 1del.ent A;2904 1dow.ent A 1b77.ent A;2905 1dow.ent A 4h6a.ent A;2906 1dow.ent A 4a25.ent B;2907 1dow.ent A 3q5v.ent A;2908 1dow.ent A 3nmr.ent A;2909 1dow.ent A 3kjz.ent A;2910 1dow.ent A 3g5k.ent A;2911 1dow.ent A 3csr.ent A;2912 1dow.ent A 2zk9.ent X;2913 1dow.ent A 2wmm.ent A;2914 1dow.ent A 2q7a.ent A;2915 1dow.ent A 2ill.ent A;2916 1dow.ent A 2fko.ent A;2917 1dow.ent A 2cut.ent A;2918 1dow.ent A 1zv9.ent A;2919 1dow.ent A 1xt0.ent B;2920 1dow.ent A 1u7p.ent A;2921 1dow.ent A 1r8n.ent A;2922 1dow.ent A 1ocy.ent A;2923 1dow.ent A 1m4d.ent A;2924 1dow.ent A 1iqq.ent A;2925 1dow.ent A 1gui.ent A;2926 1dow.ent A 1ffk.ent T;2927 1cau.ent A 5mdh.ent A;2928 1cau.ent A 3two.ent B;2929 1cau.ent A 3n9s.ent A;2930 1cau.ent A 3exe.ent B;2931 1cau.ent A 2yy7.ent A;2932 1cau.ent A 2qx3.ent A;2933 1cau.ent A 2hk2.ent A;2934 1cau.ent A 2eih.ent A;2935 1cau.ent A 1yg9.ent A;2936 1cau.ent A 1tca.ent A;2937 1cau.ent A 1pxz.ent A;2938 1cau.ent A 1llp.ent A;2939 1cau.ent A 1h6u.ent A;2940 1cau.ent A 1ea7.ent A;2941 1cau.ent A 1a4m.ent A;2942 1cau.ent A 4b5w.ent A;2943 1cau.ent A 3q1n.ent A;2944 1cau.ent A 3m66.ent A;2945 1cau.ent A 3gae.ent A;2946 1cau.ent A 3bcz.ent A;2947 1cau.ent A 2voa.ent A;2948 1cau.ent A 2p9h.ent A;2949 1cau.ent A 2gnu.ent M;2950 1cau.ent A 2ci1.ent A;2951 1cau.ent A 1xq6.ent A;2952 1cau.ent A 1tml.ent A;2953 1cau.ent A 1qgj.ent A;2954 1cau.ent A 1nas.ent A;2955 1cau.ent A 1hvq.ent A;2956 1cau.ent A 1fts.ent A;2957 1cau.ent A 1dea.ent A;2958 1cau.ent A 1b35.ent B;2959 1cau.ent A 1a0g.ent B;2960 1cau.ent A 3tt9.ent A;2961 1cau.ent A 3pnl.ent B;2962 1cau.ent A 3kvd.ent D;2963 1cau.ent A 3fj7.ent A;2964 1cau.ent A 3cjj.ent A;2965 1cau.ent A 3ai9.ent X;2966 1cau.ent A 2vg9.ent A;2967 1cau.ent A 2p0j.ent A;2968 1cau.ent A 2is9.ent A;2969 1cau.ent A 2eyi.ent A;2970 1cau.ent A 2cga.ent A;2971 1cau.ent A 1ym0.ent A;2972 1cau.ent A 1twf.ent E;2973 1cau.ent A 1rc9.ent A;2974 1cau.ent A 1pn9.ent A;2975 1cau.ent A 1n7k.ent A;2976 1cau.ent A 1kg5.ent A;2977 1cau.ent A 1gis.ent A;2978 1cau.ent A 1fuj.ent A;2979 1cau.ent A 1del.ent A;2980 1cau.ent A 1b77.ent A;2981 1cau.ent A 4h6a.ent A;2982 1cau.ent A 4a25.ent B;2983 1cau.ent A 3q5v.ent A;2984 1cau.ent A 3nmr.ent A;2985 1cau.ent A 3kjz.ent A;2986 1cau.ent A 3g5k.ent A;2987 1cau.ent A 3csr.ent A;2988 1cau.ent A 2zk9.ent X;2989 1cau.ent A 2wmm.ent A;2990 1cau.ent A 2q7a.ent A;2991 1cau.ent A 2ill.ent A;2992 1cau.ent A 2fko.ent A;2993 1cau.ent A 2cut.ent A;2994 1cau.ent A 1zv9.ent A;2995 1cau.ent A 1xt0.ent B;2996 1cau.ent A 1u7p.ent A;2997 1cau.ent A 1r8n.ent A;2998 1cau.ent A 1ocy.ent A;2999 1cau.ent A 1m4d.ent A;3000 1cau.ent A 1iqq.ent A;3001 1cau.ent A 1gui.ent A;3002 1cau.ent A 1ffk.ent T;3003 1cau.ent A 1dow.ent A;3004 1ava.ent C 5mdh.ent A;3005 1ava.ent C 3two.ent B;3006 1ava.ent C 3n9s.ent A;3007 1ava.ent C 3exe.ent B;3008 1ava.ent C 2yy7.ent A;3009 1ava.ent C 2qx3.ent A;3010 1ava.ent C 2hk2.ent A;3011 1ava.ent C 2eih.ent A;3012 1ava.ent C 1yg9.ent A;3013 1ava.ent C 1tca.ent A;3014 1ava.ent C 1pxz.ent A;3015 1ava.ent C 1llp.ent A;3016 1ava.ent C 1h6u.ent A;3017 1ava.ent C 1ea7.ent A;3018 1ava.ent C 1a4m.ent A;3019 1ava.ent C 4b5w.ent A;3020 1ava.ent C 3q1n.ent A;3021 1ava.ent C 3m66.ent A;3022 1ava.ent C 3gae.ent A;3023 1ava.ent C 3bcz.ent A;3024 1ava.ent C 2voa.ent A;3025 1ava.ent C 2p9h.ent A;3026 1ava.ent C 2gnu.ent M;3027 1ava.ent C 2ci1.ent A;3028 1ava.ent C 1xq6.ent A;3029 1ava.ent C 1tml.ent A;3030 1ava.ent C 1qgj.ent A;3031 1ava.ent C 1nas.ent A;3032 1ava.ent C 1hvq.ent A;3033 1ava.ent C 1fts.ent A;3034 1ava.ent C 1dea.ent A;3035 1ava.ent C 1b35.ent B;3036 1ava.ent C 1a0g.ent B;3037 1ava.ent C 3tt9.ent A;3038 1ava.ent C 3pnl.ent B;3039 1ava.ent C 3kvd.ent D;3040 1ava.ent C 3fj7.ent A;3041 1ava.ent C 3cjj.ent A;3042 1ava.ent C 3ai9.ent X;3043 1ava.ent C 2vg9.ent A;3044 1ava.ent C 2p0j.ent A;3045 1ava.ent C 2is9.ent A;3046 1ava.ent C 2eyi.ent A;3047 1ava.ent C 2cga.ent A;3048 1ava.ent C 1ym0.ent A;3049 1ava.ent C 1twf.ent E;3050 1ava.ent C 1rc9.ent A;3051 1ava.ent C 1pn9.ent A;3052 1ava.ent C 1n7k.ent A;3053 1ava.ent C 1kg5.ent A;3054 1ava.ent C 1gis.ent A;3055 1ava.ent C 1fuj.ent A;3056 1ava.ent C 1del.ent A;3057 1ava.ent C 1b77.ent A;3058 1ava.ent C 4h6a.ent A;3059 1ava.ent C 4a25.ent B;3060 1ava.ent C 3q5v.ent A;3061 1ava.ent C 3nmr.ent A;3062 1ava.ent C 3kjz.ent A;3063 1ava.ent C 3g5k.ent A;3064 1ava.ent C 3csr.ent A;3065 1ava.ent C 2zk9.ent X;3066 1ava.ent C 2wmm.ent A;3067 1ava.ent C 2q7a.ent A;3068 1ava.ent C 2ill.ent A;3069 1ava.ent C 2fko.ent A;3070 1ava.ent C 2cut.ent A;3071 1ava.ent C 1zv9.ent A;3072 1ava.ent C 1xt0.ent B;3073 1ava.ent C 1u7p.ent A;3074 1ava.ent C 1r8n.ent A;3075 1ava.ent C 1ocy.ent A;3076 1ava.ent C 1m4d.ent A;3077 1ava.ent C 1iqq.ent A;3078 1ava.ent C 1gui.ent A;3079 1ava.ent C 1ffk.ent T;3080 1ava.ent C 1dow.ent A;3081 1ava.ent C 1cau.ent A;3082 4fei.ent A 5mdh.ent A;3083 4fei.ent A 3two.ent B;3084 4fei.ent A 3n9s.ent A;3085 4fei.ent A 3exe.ent B;3086 4fei.ent A 2yy7.ent A;3087 4fei.ent A 2qx3.ent A;3088 4fei.ent A 2hk2.ent A;3089 4fei.ent A 2eih.ent A;3090 4fei.ent A 1yg9.ent A;3091 4fei.ent A 1tca.ent A;3092 4fei.ent A 1pxz.ent A;3093 4fei.ent A 1llp.ent A;3094 4fei.ent A 1h6u.ent A;3095 4fei.ent A 1ea7.ent A;3096 4fei.ent A 1a4m.ent A;3097 4fei.ent A 4b5w.ent A;3098 4fei.ent A 3q1n.ent A;3099 4fei.ent A 3m66.ent A;3100 4fei.ent A 3gae.ent A;3101 4fei.ent A 3bcz.ent A;3102 4fei.ent A 2voa.ent A;3103 4fei.ent A 2p9h.ent A;3104 4fei.ent A 2gnu.ent M;3105 4fei.ent A 2ci1.ent A;3106 4fei.ent A 1xq6.ent A;3107 4fei.ent A 1tml.ent A;3108 4fei.ent A 1qgj.ent A;3109 4fei.ent A 1nas.ent A;3110 4fei.ent A 1hvq.ent A;3111 4fei.ent A 1fts.ent A;3112 4fei.ent A 1dea.ent A;3113 4fei.ent A 1b35.ent B;3114 4fei.ent A 1a0g.ent B;3115 4fei.ent A 3tt9.ent A;3116 4fei.ent A 3pnl.ent B;3117 4fei.ent A 3kvd.ent D;3118 4fei.ent A 3fj7.ent A;3119 4fei.ent A 3cjj.ent A;3120 4fei.ent A 3ai9.ent X;3121 4fei.ent A 2vg9.ent A;3122 4fei.ent A 2p0j.ent A;3123 4fei.ent A 2is9.ent A;3124 4fei.ent A 2eyi.ent A;3125 4fei.ent A 2cga.ent A;3126 4fei.ent A 1ym0.ent A;3127 4fei.ent A 1twf.ent E;3128 4fei.ent A 1rc9.ent A;3129 4fei.ent A 1pn9.ent A;3130 4fei.ent A 1n7k.ent A;3131 4fei.ent A 1kg5.ent A;3132 4fei.ent A 1gis.ent A;3133 4fei.ent A 1fuj.ent A;3134 4fei.ent A 1del.ent A;3135 4fei.ent A 1b77.ent A;3136 4fei.ent A 4h6a.ent A;3137 4fei.ent A 4a25.ent B;3138 4fei.ent A 3q5v.ent A;3139 4fei.ent A 3nmr.ent A;3140 4fei.ent A 3kjz.ent A;3141 4fei.ent A 3g5k.ent A;3142 4fei.ent A 3csr.ent A;3143 4fei.ent A 2zk9.ent X;3144 4fei.ent A 2wmm.ent A;3145 4fei.ent A 2q7a.ent A;3146 4fei.ent A 2ill.ent A;3147 4fei.ent A 2fko.ent A;3148 4fei.ent A 2cut.ent A;3149 4fei.ent A 1zv9.ent A;3150 4fei.ent A 1xt0.ent B;3151 4fei.ent A 1u7p.ent A;3152 4fei.ent A 1r8n.ent A;3153 4fei.ent A 1ocy.ent A;3154 4fei.ent A 1m4d.ent A;3155 4fei.ent A 1iqq.ent A;3156 4fei.ent A 1gui.ent A;3157 4fei.ent A 1ffk.ent T;3158 4fei.ent A 1dow.ent A;3159 4fei.ent A 1cau.ent A;3160 4fei.ent A 1ava.ent C;3161 3zqx.ent A 5mdh.ent A;3162 3zqx.ent A 3two.ent B;3163 3zqx.ent A 3n9s.ent A;3164 3zqx.ent A 3exe.ent B;3165 3zqx.ent A 2yy7.ent A;3166 3zqx.ent A 2qx3.ent A;3167 3zqx.ent A 2hk2.ent A;3168 3zqx.ent A 2eih.ent A;3169 3zqx.ent A 1yg9.ent A;3170 3zqx.ent A 1tca.ent A;3171 3zqx.ent A 1pxz.ent A;3172 3zqx.ent A 1llp.ent A;3173 3zqx.ent A 1h6u.ent A;3174 3zqx.ent A 1ea7.ent A;3175 3zqx.ent A 1a4m.ent A;3176 3zqx.ent A 4b5w.ent A;3177 3zqx.ent A 3q1n.ent A;3178 3zqx.ent A 3m66.ent A;3179 3zqx.ent A 3gae.ent A;3180 3zqx.ent A 3bcz.ent A;3181 3zqx.ent A 2voa.ent A;3182 3zqx.ent A 2p9h.ent A;3183 3zqx.ent A 2gnu.ent M;3184 3zqx.ent A 2ci1.ent A;3185 3zqx.ent A 1xq6.ent A;3186 3zqx.ent A 1tml.ent A;3187 3zqx.ent A 1qgj.ent A;3188 3zqx.ent A 1nas.ent A;3189 3zqx.ent A 1hvq.ent A;3190 3zqx.ent A 1fts.ent A;3191 3zqx.ent A 1dea.ent A;3192 3zqx.ent A 1b35.ent B;3193 3zqx.ent A 1a0g.ent B;3194 3zqx.ent A 3tt9.ent A;3195 3zqx.ent A 3pnl.ent B;3196 3zqx.ent A 3kvd.ent D;3197 3zqx.ent A 3fj7.ent A;3198 3zqx.ent A 3cjj.ent A;3199 3zqx.ent A 3ai9.ent X;3200 3zqx.ent A 2vg9.ent A;3201 3zqx.ent A 2p0j.ent A;3202 3zqx.ent A 2is9.ent A;3203 3zqx.ent A 2eyi.ent A;3204 3zqx.ent A 2cga.ent A;3205 3zqx.ent A 1ym0.ent A;3206 3zqx.ent A 1twf.ent E;3207 3zqx.ent A 1rc9.ent A;3208 3zqx.ent A 1pn9.ent A;3209 3zqx.ent A 1n7k.ent A;3210 3zqx.ent A 1kg5.ent A;3211 3zqx.ent A 1gis.ent A;3212 3zqx.ent A 1fuj.ent A;3213 3zqx.ent A 1del.ent A;3214 3zqx.ent A 1b77.ent A;3215 3zqx.ent A 4h6a.ent A;3216 3zqx.ent A 4a25.ent B;3217 3zqx.ent A 3q5v.ent A;3218 3zqx.ent A 3nmr.ent A;3219 3zqx.ent A 3kjz.ent A;3220 3zqx.ent A 3g5k.ent A;3221 3zqx.ent A 3csr.ent A;3222 3zqx.ent A 2zk9.ent X;3223 3zqx.ent A 2wmm.ent A;3224 3zqx.ent A 2q7a.ent A;3225 3zqx.ent A 2ill.ent A;3226 3zqx.ent A 2fko.ent A;3227 3zqx.ent A 2cut.ent A;3228 3zqx.ent A 1zv9.ent A;3229 3zqx.ent A 1xt0.ent B;3230 3zqx.ent A 1u7p.ent A;3231 3zqx.ent A 1r8n.ent A;3232 3zqx.ent A 1ocy.ent A;3233 3zqx.ent A 1m4d.ent A;3234 3zqx.ent A 1iqq.ent A;3235 3zqx.ent A 1gui.ent A;3236 3zqx.ent A 1ffk.ent T;3237 3zqx.ent A 1dow.ent A;3238 3zqx.ent A 1cau.ent A;3239 3zqx.ent A 1ava.ent C;3240 3zqx.ent A 4fei.ent A;3241 3sek.ent B 5mdh.ent A;3242 3sek.ent B 3two.ent B;3243 3sek.ent B 3n9s.ent A;3244 3sek.ent B 3exe.ent B;3245 3sek.ent B 2yy7.ent A;3246 3sek.ent B 2qx3.ent A;3247 3sek.ent B 2hk2.ent A;3248 3sek.ent B 2eih.ent A;3249 3sek.ent B 1yg9.ent A;3250 3sek.ent B 1tca.ent A;3251 3sek.ent B 1pxz.ent A;3252 3sek.ent B 1llp.ent A;3253 3sek.ent B 1h6u.ent A;3254 3sek.ent B 1ea7.ent A;3255 3sek.ent B 1a4m.ent A;3256 3sek.ent B 4b5w.ent A;3257 3sek.ent B 3q1n.ent A;3258 3sek.ent B 3m66.ent A;3259 3sek.ent B 3gae.ent A;3260 3sek.ent B 3bcz.ent A;3261 3sek.ent B 2voa.ent A;3262 3sek.ent B 2p9h.ent A;3263 3sek.ent B 2gnu.ent M;3264 3sek.ent B 2ci1.ent A;3265 3sek.ent B 1xq6.ent A;3266 3sek.ent B 1tml.ent A;3267 3sek.ent B 1qgj.ent A;3268 3sek.ent B 1nas.ent A;3269 3sek.ent B 1hvq.ent A;3270 3sek.ent B 1fts.ent A;3271 3sek.ent B 1dea.ent A;3272 3sek.ent B 1b35.ent B;3273 3sek.ent B 1a0g.ent B;3274 3sek.ent B 3tt9.ent A;3275 3sek.ent B 3pnl.ent B;3276 3sek.ent B 3kvd.ent D;3277 3sek.ent B 3fj7.ent A;3278 3sek.ent B 3cjj.ent A;3279 3sek.ent B 3ai9.ent X;3280 3sek.ent B 2vg9.ent A;3281 3sek.ent B 2p0j.ent A;3282 3sek.ent B 2is9.ent A;3283 3sek.ent B 2eyi.ent A;3284 3sek.ent B 2cga.ent A;3285 3sek.ent B 1ym0.ent A;3286 3sek.ent B 1twf.ent E;3287 3sek.ent B 1rc9.ent A;3288 3sek.ent B 1pn9.ent A;3289 3sek.ent B 1n7k.ent A;3290 3sek.ent B 1kg5.ent A;3291 3sek.ent B 1gis.ent A;3292 3sek.ent B 1fuj.ent A;3293 3sek.ent B 1del.ent A;3294 3sek.ent B 1b77.ent A;3295 3sek.ent B 4h6a.ent A;3296 3sek.ent B 4a25.ent B;3297 3sek.ent B 3q5v.ent A;3298 3sek.ent B 3nmr.ent A;3299 3sek.ent B 3kjz.ent A;3300 3sek.ent B 3g5k.ent A;3301 3sek.ent B 3csr.ent A;3302 3sek.ent B 2zk9.ent X;3303 3sek.ent B 2wmm.ent A;3304 3sek.ent B 2q7a.ent A;3305 3sek.ent B 2ill.ent A;3306 3sek.ent B 2fko.ent A;3307 3sek.ent B 2cut.ent A;3308 3sek.ent B 1zv9.ent A;3309 3sek.ent B 1xt0.ent B;3310 3sek.ent B 1u7p.ent A;3311 3sek.ent B 1r8n.ent A;3312 3sek.ent B 1ocy.ent A;3313 3sek.ent B 1m4d.ent A;3314 3sek.ent B 1iqq.ent A;3315 3sek.ent B 1gui.ent A;3316 3sek.ent B 1ffk.ent T;3317 3sek.ent B 1dow.ent A;3318 3sek.ent B 1cau.ent A;3319 3sek.ent B 1ava.ent C;3320 3sek.ent B 4fei.ent A;3321 3sek.ent B 3zqx.ent A;3322 3obl.ent A 5mdh.ent A;3323 3obl.ent A 3two.ent B;3324 3obl.ent A 3n9s.ent A;3325 3obl.ent A 3exe.ent B;3326 3obl.ent A 2yy7.ent A;3327 3obl.ent A 2qx3.ent A;3328 3obl.ent A 2hk2.ent A;3329 3obl.ent A 2eih.ent A;3330 3obl.ent A 1yg9.ent A;3331 3obl.ent A 1tca.ent A;3332 3obl.ent A 1pxz.ent A;3333 3obl.ent A 1llp.ent A;3334 3obl.ent A 1h6u.ent A;3335 3obl.ent A 1ea7.ent A;3336 3obl.ent A 1a4m.ent A;3337 3obl.ent A 4b5w.ent A;3338 3obl.ent A 3q1n.ent A;3339 3obl.ent A 3m66.ent A;3340 3obl.ent A 3gae.ent A;3341 3obl.ent A 3bcz.ent A;3342 3obl.ent A 2voa.ent A;3343 3obl.ent A 2p9h.ent A;3344 3obl.ent A 2gnu.ent M;3345 3obl.ent A 2ci1.ent A;3346 3obl.ent A 1xq6.ent A;3347 3obl.ent A 1tml.ent A;3348 3obl.ent A 1qgj.ent A;3349 3obl.ent A 1nas.ent A;3350 3obl.ent A 1hvq.ent A;3351 3obl.ent A 1fts.ent A;3352 3obl.ent A 1dea.ent A;3353 3obl.ent A 1b35.ent B;3354 3obl.ent A 1a0g.ent B;3355 3obl.ent A 3tt9.ent A;3356 3obl.ent A 3pnl.ent B;3357 3obl.ent A 3kvd.ent D;3358 3obl.ent A 3fj7.ent A;3359 3obl.ent A 3cjj.ent A;3360 3obl.ent A 3ai9.ent X;3361 3obl.ent A 2vg9.ent A;3362 3obl.ent A 2p0j.ent A;3363 3obl.ent A 2is9.ent A;3364 3obl.ent A 2eyi.ent A;3365 3obl.ent A 2cga.ent A;3366 3obl.ent A 1ym0.ent A;3367 3obl.ent A 1twf.ent E;3368 3obl.ent A 1rc9.ent A;3369 3obl.ent A 1pn9.ent A;3370 3obl.ent A 1n7k.ent A;3371 3obl.ent A 1kg5.ent A;3372 3obl.ent A 1gis.ent A;3373 3obl.ent A 1fuj.ent A;3374 3obl.ent A 1del.ent A;3375 3obl.ent A 1b77.ent A;3376 3obl.ent A 4h6a.ent A;3377 3obl.ent A 4a25.ent B;3378 3obl.ent A 3q5v.ent A;3379 3obl.ent A 3nmr.ent A;3380 3obl.ent A 3kjz.ent A;3381 3obl.ent A 3g5k.ent A;3382 3obl.ent A 3csr.ent A;3383 3obl.ent A 2zk9.ent X;3384 3obl.ent A 2wmm.ent A;3385 3obl.ent A 2q7a.ent A;3386 3obl.ent A 2ill.ent A;3387 3obl.ent A 2fko.ent A;3388 3obl.ent A 2cut.ent A;3389 3obl.ent A 1zv9.ent A;3390 3obl.ent A 1xt0.ent B;3391 3obl.ent A 1u7p.ent A;3392 3obl.ent A 1r8n.ent A;3393 3obl.ent A 1ocy.ent A;3394 3obl.ent A 1m4d.ent A;3395 3obl.ent A 1iqq.ent A;3396 3obl.ent A 1gui.ent A;3397 3obl.ent A 1ffk.ent T;3398 3obl.ent A 1dow.ent A;3399 3obl.ent A 1cau.ent A;3400 3obl.ent A 1ava.ent C;3401 3obl.ent A 4fei.ent A;3402 3obl.ent A 3zqx.ent A;3403 3obl.ent A 3sek.ent B;3404 3mao.ent A 5mdh.ent A;3405 3mao.ent A 3two.ent B;3406 3mao.ent A 3n9s.ent A;3407 3mao.ent A 3exe.ent B;3408 3mao.ent A 2yy7.ent A;3409 3mao.ent A 2qx3.ent A;3410 3mao.ent A 2hk2.ent A;3411 3mao.ent A 2eih.ent A;3412 3mao.ent A 1yg9.ent A;3413 3mao.ent A 1tca.ent A;3414 3mao.ent A 1pxz.ent A;3415 3mao.ent A 1llp.ent A;3416 3mao.ent A 1h6u.ent A;3417 3mao.ent A 1ea7.ent A;3418 3mao.ent A 1a4m.ent A;3419 3mao.ent A 4b5w.ent A;3420 3mao.ent A 3q1n.ent A;3421 3mao.ent A 3m66.ent A;3422 3mao.ent A 3gae.ent A;3423 3mao.ent A 3bcz.ent A;3424 3mao.ent A 2voa.ent A;3425 3mao.ent A 2p9h.ent A;3426 3mao.ent A 2gnu.ent M;3427 3mao.ent A 2ci1.ent A;3428 3mao.ent A 1xq6.ent A;3429 3mao.ent A 1tml.ent A;3430 3mao.ent A 1qgj.ent A;3431 3mao.ent A 1nas.ent A;3432 3mao.ent A 1hvq.ent A;3433 3mao.ent A 1fts.ent A;3434 3mao.ent A 1dea.ent A;3435 3mao.ent A 1b35.ent B;3436 3mao.ent A 1a0g.ent B;3437 3mao.ent A 3tt9.ent A;3438 3mao.ent A 3pnl.ent B;3439 3mao.ent A 3kvd.ent D;3440 3mao.ent A 3fj7.ent A;3441 3mao.ent A 3cjj.ent A;3442 3mao.ent A 3ai9.ent X;3443 3mao.ent A 2vg9.ent A;3444 3mao.ent A 2p0j.ent A;3445 3mao.ent A 2is9.ent A;3446 3mao.ent A 2eyi.ent A;3447 3mao.ent A 2cga.ent A;3448 3mao.ent A 1ym0.ent A;3449 3mao.ent A 1twf.ent E;3450 3mao.ent A 1rc9.ent A;3451 3mao.ent A 1pn9.ent A;3452 3mao.ent A 1n7k.ent A;3453 3mao.ent A 1kg5.ent A;3454 3mao.ent A 1gis.ent A;3455 3mao.ent A 1fuj.ent A;3456 3mao.ent A 1del.ent A;3457 3mao.ent A 1b77.ent A;3458 3mao.ent A 4h6a.ent A;3459 3mao.ent A 4a25.ent B;3460 3mao.ent A 3q5v.ent A;3461 3mao.ent A 3nmr.ent A;3462 3mao.ent A 3kjz.ent A;3463 3mao.ent A 3g5k.ent A;3464 3mao.ent A 3csr.ent A;3465 3mao.ent A 2zk9.ent X;3466 3mao.ent A 2wmm.ent A;3467 3mao.ent A 2q7a.ent A;3468 3mao.ent A 2ill.ent A;3469 3mao.ent A 2fko.ent A;3470 3mao.ent A 2cut.ent A;3471 3mao.ent A 1zv9.ent A;3472 3mao.ent A 1xt0.ent B;3473 3mao.ent A 1u7p.ent A;3474 3mao.ent A 1r8n.ent A;3475 3mao.ent A 1ocy.ent A;3476 3mao.ent A 1m4d.ent A;3477 3mao.ent A 1iqq.ent A;3478 3mao.ent A 1gui.ent A;3479 3mao.ent A 1ffk.ent T;3480 3mao.ent A 1dow.ent A;3481 3mao.ent A 1cau.ent A;3482 3mao.ent A 1ava.ent C;3483 3mao.ent A 4fei.ent A;3484 3mao.ent A 3zqx.ent A;3485 3mao.ent A 3sek.ent B;3486 3mao.ent A 3obl.ent A;3487 3kk4.ent A 5mdh.ent A;3488 3kk4.ent A 3two.ent B;3489 3kk4.ent A 3n9s.ent A;3490 3kk4.ent A 3exe.ent B;3491 3kk4.ent A 2yy7.ent A;3492 3kk4.ent A 2qx3.ent A;3493 3kk4.ent A 2hk2.ent A;3494 3kk4.ent A 2eih.ent A;3495 3kk4.ent A 1yg9.ent A;3496 3kk4.ent A 1tca.ent A;3497 3kk4.ent A 1pxz.ent A;3498 3kk4.ent A 1llp.ent A;3499 3kk4.ent A 1h6u.ent A;3500 3kk4.ent A 1ea7.ent A;3501 3kk4.ent A 1a4m.ent A;3502 3kk4.ent A 4b5w.ent A;3503 3kk4.ent A 3q1n.ent A;3504 3kk4.ent A 3m66.ent A;3505 3kk4.ent A 3gae.ent A;3506 3kk4.ent A 3bcz.ent A;3507 3kk4.ent A 2voa.ent A;3508 3kk4.ent A 2p9h.ent A;3509 3kk4.ent A 2gnu.ent M;3510 3kk4.ent A 2ci1.ent A;3511 3kk4.ent A 1xq6.ent A;3512 3kk4.ent A 1tml.ent A;3513 3kk4.ent A 1qgj.ent A;3514 3kk4.ent A 1nas.ent A;3515 3kk4.ent A 1hvq.ent A;3516 3kk4.ent A 1fts.ent A;3517 3kk4.ent A 1dea.ent A;3518 3kk4.ent A 1b35.ent B;3519 3kk4.ent A 1a0g.ent B;3520 3kk4.ent A 3tt9.ent A;3521 3kk4.ent A 3pnl.ent B;3522 3kk4.ent A 3kvd.ent D;3523 3kk4.ent A 3fj7.ent A;3524 3kk4.ent A 3cjj.ent A;3525 3kk4.ent A 3ai9.ent X;3526 3kk4.ent A 2vg9.ent A;3527 3kk4.ent A 2p0j.ent A;3528 3kk4.ent A 2is9.ent A;3529 3kk4.ent A 2eyi.ent A;3530 3kk4.ent A 2cga.ent A;3531 3kk4.ent A 1ym0.ent A;3532 3kk4.ent A 1twf.ent E;3533 3kk4.ent A 1rc9.ent A;3534 3kk4.ent A 1pn9.ent A;3535 3kk4.ent A 1n7k.ent A;3536 3kk4.ent A 1kg5.ent A;3537 3kk4.ent A 1gis.ent A;3538 3kk4.ent A 1fuj.ent A;3539 3kk4.ent A 1del.ent A;3540 3kk4.ent A 1b77.ent A;3541 3kk4.ent A 4h6a.ent A;3542 3kk4.ent A 4a25.ent B;3543 3kk4.ent A 3q5v.ent A;3544 3kk4.ent A 3nmr.ent A;3545 3kk4.ent A 3kjz.ent A;3546 3kk4.ent A 3g5k.ent A;3547 3kk4.ent A 3csr.ent A;3548 3kk4.ent A 2zk9.ent X;3549 3kk4.ent A 2wmm.ent A;3550 3kk4.ent A 2q7a.ent A;3551 3kk4.ent A 2ill.ent A;3552 3kk4.ent A 2fko.ent A;3553 3kk4.ent A 2cut.ent A;3554 3kk4.ent A 1zv9.ent A;3555 3kk4.ent A 1xt0.ent B;3556 3kk4.ent A 1u7p.ent A;3557 3kk4.ent A 1r8n.ent A;3558 3kk4.ent A 1ocy.ent A;3559 3kk4.ent A 1m4d.ent A;3560 3kk4.ent A 1iqq.ent A;3561 3kk4.ent A 1gui.ent A;3562 3kk4.ent A 1ffk.ent T;3563 3kk4.ent A 1dow.ent A;3564 3kk4.ent A 1cau.ent A;3565 3kk4.ent A 1ava.ent C;3566 3kk4.ent A 4fei.ent A;3567 3kk4.ent A 3zqx.ent A;3568 3kk4.ent A 3sek.ent B;3569 3kk4.ent A 3obl.ent A;3570 3kk4.ent A 3mao.ent A;3571 3gef.ent A 5mdh.ent A;3572 3gef.ent A 3two.ent B;3573 3gef.ent A 3n9s.ent A;3574 3gef.ent A 3exe.ent B;3575 3gef.ent A 2yy7.ent A;3576 3gef.ent A 2qx3.ent A;3577 3gef.ent A 2hk2.ent A;3578 3gef.ent A 2eih.ent A;3579 3gef.ent A 1yg9.ent A;3580 3gef.ent A 1tca.ent A;3581 3gef.ent A 1pxz.ent A;3582 3gef.ent A 1llp.ent A;3583 3gef.ent A 1h6u.ent A;3584 3gef.ent A 1ea7.ent A;3585 3gef.ent A 1a4m.ent A;3586 3gef.ent A 4b5w.ent A;3587 3gef.ent A 3q1n.ent A;3588 3gef.ent A 3m66.ent A;3589 3gef.ent A 3gae.ent A;3590 3gef.ent A 3bcz.ent A;3591 3gef.ent A 2voa.ent A;3592 3gef.ent A 2p9h.ent A;3593 3gef.ent A 2gnu.ent M;3594 3gef.ent A 2ci1.ent A;3595 3gef.ent A 1xq6.ent A;3596 3gef.ent A 1tml.ent A;3597 3gef.ent A 1qgj.ent A;3598 3gef.ent A 1nas.ent A;3599 3gef.ent A 1hvq.ent A;3600 3gef.ent A 1fts.ent A;3601 3gef.ent A 1dea.ent A;3602 3gef.ent A 1b35.ent B;3603 3gef.ent A 1a0g.ent B;3604 3gef.ent A 3tt9.ent A;3605 3gef.ent A 3pnl.ent B;3606 3gef.ent A 3kvd.ent D;3607 3gef.ent A 3fj7.ent A;3608 3gef.ent A 3cjj.ent A;3609 3gef.ent A 3ai9.ent X;3610 3gef.ent A 2vg9.ent A;3611 3gef.ent A 2p0j.ent A;3612 3gef.ent A 2is9.ent A;3613 3gef.ent A 2eyi.ent A;3614 3gef.ent A 2cga.ent A;3615 3gef.ent A 1ym0.ent A;3616 3gef.ent A 1twf.ent E;3617 3gef.ent A 1rc9.ent A;3618 3gef.ent A 1pn9.ent A;3619 3gef.ent A 1n7k.ent A;3620 3gef.ent A 1kg5.ent A;3621 3gef.ent A 1gis.ent A;3622 3gef.ent A 1fuj.ent A;3623 3gef.ent A 1del.ent A;3624 3gef.ent A 1b77.ent A;3625 3gef.ent A 4h6a.ent A;3626 3gef.ent A 4a25.ent B;3627 3gef.ent A 3q5v.ent A;3628 3gef.ent A 3nmr.ent A;3629 3gef.ent A 3kjz.ent A;3630 3gef.ent A 3g5k.ent A;3631 3gef.ent A 3csr.ent A;3632 3gef.ent A 2zk9.ent X;3633 3gef.ent A 2wmm.ent A;3634 3gef.ent A 2q7a.ent A;3635 3gef.ent A 2ill.ent A;3636 3gef.ent A 2fko.ent A;3637 3gef.ent A 2cut.ent A;3638 3gef.ent A 1zv9.ent A;3639 3gef.ent A 1xt0.ent B;3640 3gef.ent A 1u7p.ent A;3641 3gef.ent A 1r8n.ent A;3642 3gef.ent A 1ocy.ent A;3643 3gef.ent A 1m4d.ent A;3644 3gef.ent A 1iqq.ent A;3645 3gef.ent A 1gui.ent A;3646 3gef.ent A 1ffk.ent T;3647 3gef.ent A 1dow.ent A;3648 3gef.ent A 1cau.ent A;3649 3gef.ent A 1ava.ent C;3650 3gef.ent A 4fei.ent A;3651 3gef.ent A 3zqx.ent A;3652 3gef.ent A 3sek.ent B;3653 3gef.ent A 3obl.ent A;3654 3gef.ent A 3mao.ent A;3655 3gef.ent A 3kk4.ent A;3656 3ent.ent A 5mdh.ent A;3657 3ent.ent A 3two.ent B;3658 3ent.ent A 3n9s.ent A;3659 3ent.ent A 3exe.ent B;3660 3ent.ent A 2yy7.ent A;3661 3ent.ent A 2qx3.ent A;3662 3ent.ent A 2hk2.ent A;3663 3ent.ent A 2eih.ent A;3664 3ent.ent A 1yg9.ent A;3665 3ent.ent A 1tca.ent A;3666 3ent.ent A 1pxz.ent A;3667 3ent.ent A 1llp.ent A;3668 3ent.ent A 1h6u.ent A;3669 3ent.ent A 1ea7.ent A;3670 3ent.ent A 1a4m.ent A;3671 3ent.ent A 4b5w.ent A;3672 3ent.ent A 3q1n.ent A;3673 3ent.ent A 3m66.ent A;3674 3ent.ent A 3gae.ent A;3675 3ent.ent A 3bcz.ent A;3676 3ent.ent A 2voa.ent A;3677 3ent.ent A 2p9h.ent A;3678 3ent.ent A 2gnu.ent M;3679 3ent.ent A 2ci1.ent A;3680 3ent.ent A 1xq6.ent A;3681 3ent.ent A 1tml.ent A;3682 3ent.ent A 1qgj.ent A;3683 3ent.ent A 1nas.ent A;3684 3ent.ent A 1hvq.ent A;3685 3ent.ent A 1fts.ent A;3686 3ent.ent A 1dea.ent A;3687 3ent.ent A 1b35.ent B;3688 3ent.ent A 1a0g.ent B;3689 3ent.ent A 3tt9.ent A;3690 3ent.ent A 3pnl.ent B;3691 3ent.ent A 3kvd.ent D;3692 3ent.ent A 3fj7.ent A;3693 3ent.ent A 3cjj.ent A;3694 3ent.ent A 3ai9.ent X;3695 3ent.ent A 2vg9.ent A;3696 3ent.ent A 2p0j.ent A;3697 3ent.ent A 2is9.ent A;3698 3ent.ent A 2eyi.ent A;3699 3ent.ent A 2cga.ent A;3700 3ent.ent A 1ym0.ent A;3701 3ent.ent A 1twf.ent E;3702 3ent.ent A 1rc9.ent A;3703 3ent.ent A 1pn9.ent A;3704 3ent.ent A 1n7k.ent A;3705 3ent.ent A 1kg5.ent A;3706 3ent.ent A 1gis.ent A;3707 3ent.ent A 1fuj.ent A;3708 3ent.ent A 1del.ent A;3709 3ent.ent A 1b77.ent A;3710 3ent.ent A 4h6a.ent A;3711 3ent.ent A 4a25.ent B;3712 3ent.ent A 3q5v.ent A;3713 3ent.ent A 3nmr.ent A;3714 3ent.ent A 3kjz.ent A;3715 3ent.ent A 3g5k.ent A;3716 3ent.ent A 3csr.ent A;3717 3ent.ent A 2zk9.ent X;3718 3ent.ent A 2wmm.ent A;3719 3ent.ent A 2q7a.ent A;3720 3ent.ent A 2ill.ent A;3721 3ent.ent A 2fko.ent A;3722 3ent.ent A 2cut.ent A;3723 3ent.ent A 1zv9.ent A;3724 3ent.ent A 1xt0.ent B;3725 3ent.ent A 1u7p.ent A;3726 3ent.ent A 1r8n.ent A;3727 3ent.ent A 1ocy.ent A;3728 3ent.ent A 1m4d.ent A;3729 3ent.ent A 1iqq.ent A;3730 3ent.ent A 1gui.ent A;3731 3ent.ent A 1ffk.ent T;3732 3ent.ent A 1dow.ent A;3733 3ent.ent A 1cau.ent A;3734 3ent.ent A 1ava.ent C;3735 3ent.ent A 4fei.ent A;3736 3ent.ent A 3zqx.ent A;3737 3ent.ent A 3sek.ent B;3738 3ent.ent A 3obl.ent A;3739 3ent.ent A 3mao.ent A;3740 3ent.ent A 3kk4.ent A;3741 3ent.ent A 3gef.ent A;3742 3bdq.ent B 5mdh.ent A;3743 3bdq.ent B 3two.ent B;3744 3bdq.ent B 3n9s.ent A;3745 3bdq.ent B 3exe.ent B;3746 3bdq.ent B 2yy7.ent A;3747 3bdq.ent B 2qx3.ent A;3748 3bdq.ent B 2hk2.ent A;3749 3bdq.ent B 2eih.ent A;3750 3bdq.ent B 1yg9.ent A;3751 3bdq.ent B 1tca.ent A;3752 3bdq.ent B 1pxz.ent A;3753 3bdq.ent B 1llp.ent A;3754 3bdq.ent B 1h6u.ent A;3755 3bdq.ent B 1ea7.ent A;3756 3bdq.ent B 1a4m.ent A;3757 3bdq.ent B 4b5w.ent A;3758 3bdq.ent B 3q1n.ent A;3759 3bdq.ent B 3m66.ent A;3760 3bdq.ent B 3gae.ent A;3761 3bdq.ent B 3bcz.ent A;3762 3bdq.ent B 2voa.ent A;3763 3bdq.ent B 2p9h.ent A;3764 3bdq.ent B 2gnu.ent M;3765 3bdq.ent B 2ci1.ent A;3766 3bdq.ent B 1xq6.ent A;3767 3bdq.ent B 1tml.ent A;3768 3bdq.ent B 1qgj.ent A;3769 3bdq.ent B 1nas.ent A;3770 3bdq.ent B 1hvq.ent A;3771 3bdq.ent B 1fts.ent A;3772 3bdq.ent B 1dea.ent A;3773 3bdq.ent B 1b35.ent B;3774 3bdq.ent B 1a0g.ent B;3775 3bdq.ent B 3tt9.ent A;3776 3bdq.ent B 3pnl.ent B;3777 3bdq.ent B 3kvd.ent D;3778 3bdq.ent B 3fj7.ent A;3779 3bdq.ent B 3cjj.ent A;3780 3bdq.ent B 3ai9.ent X;3781 3bdq.ent B 2vg9.ent A;3782 3bdq.ent B 2p0j.ent A;3783 3bdq.ent B 2is9.ent A;3784 3bdq.ent B 2eyi.ent A;3785 3bdq.ent B 2cga.ent A;3786 3bdq.ent B 1ym0.ent A;3787 3bdq.ent B 1twf.ent E;3788 3bdq.ent B 1rc9.ent A;3789 3bdq.ent B 1pn9.ent A;3790 3bdq.ent B 1n7k.ent A;3791 3bdq.ent B 1kg5.ent A;3792 3bdq.ent B 1gis.ent A;3793 3bdq.ent B 1fuj.ent A;3794 3bdq.ent B 1del.ent A;3795 3bdq.ent B 1b77.ent A;3796 3bdq.ent B 4h6a.ent A;3797 3bdq.ent B 4a25.ent B;3798 3bdq.ent B 3q5v.ent A;3799 3bdq.ent B 3nmr.ent A;3800 3bdq.ent B 3kjz.ent A;3801 3bdq.ent B 3g5k.ent A;3802 3bdq.ent B 3csr.ent A;3803 3bdq.ent B 2zk9.ent X;3804 3bdq.ent B 2wmm.ent A;3805 3bdq.ent B 2q7a.ent A;3806 3bdq.ent B 2ill.ent A;3807 3bdq.ent B 2fko.ent A;3808 3bdq.ent B 2cut.ent A;3809 3bdq.ent B 1zv9.ent A;3810 3bdq.ent B 1xt0.ent B;3811 3bdq.ent B 1u7p.ent A;3812 3bdq.ent B 1r8n.ent A;3813 3bdq.ent B 1ocy.ent A;3814 3bdq.ent B 1m4d.ent A;3815 3bdq.ent B 1iqq.ent A;3816 3bdq.ent B 1gui.ent A;3817 3bdq.ent B 1ffk.ent T;3818 3bdq.ent B 1dow.ent A;3819 3bdq.ent B 1cau.ent A;3820 3bdq.ent B 1ava.ent C;3821 3bdq.ent B 4fei.ent A;3822 3bdq.ent B 3zqx.ent A;3823 3bdq.ent B 3sek.ent B;3824 3bdq.ent B 3obl.ent A;3825 3bdq.ent B 3mao.ent A;3826 3bdq.ent B 3kk4.ent A;3827 3bdq.ent B 3gef.ent A;3828 3bdq.ent B 3ent.ent A;3829 2z7j.ent A 5mdh.ent A;3830 2z7j.ent A 3two.ent B;3831 2z7j.ent A 3n9s.ent A;3832 2z7j.ent A 3exe.ent B;3833 2z7j.ent A 2yy7.ent A;3834 2z7j.ent A 2qx3.ent A;3835 2z7j.ent A 2hk2.ent A;3836 2z7j.ent A 2eih.ent A;3837 2z7j.ent A 1yg9.ent A;3838 2z7j.ent A 1tca.ent A;3839 2z7j.ent A 1pxz.ent A;3840 2z7j.ent A 1llp.ent A;3841 2z7j.ent A 1h6u.ent A;3842 2z7j.ent A 1ea7.ent A;3843 2z7j.ent A 1a4m.ent A;3844 2z7j.ent A 4b5w.ent A;3845 2z7j.ent A 3q1n.ent A;3846 2z7j.ent A 3m66.ent A;3847 2z7j.ent A 3gae.ent A;3848 2z7j.ent A 3bcz.ent A;3849 2z7j.ent A 2voa.ent A;3850 2z7j.ent A 2p9h.ent A;3851 2z7j.ent A 2gnu.ent M;3852 2z7j.ent A 2ci1.ent A;3853 2z7j.ent A 1xq6.ent A;3854 2z7j.ent A 1tml.ent A;3855 2z7j.ent A 1qgj.ent A;3856 2z7j.ent A 1nas.ent A;3857 2z7j.ent A 1hvq.ent A;3858 2z7j.ent A 1fts.ent A;3859 2z7j.ent A 1dea.ent A;3860 2z7j.ent A 1b35.ent B;3861 2z7j.ent A 1a0g.ent B;3862 2z7j.ent A 3tt9.ent A;3863 2z7j.ent A 3pnl.ent B;3864 2z7j.ent A 3kvd.ent D;3865 2z7j.ent A 3fj7.ent A;3866 2z7j.ent A 3cjj.ent A;3867 2z7j.ent A 3ai9.ent X;3868 2z7j.ent A 2vg9.ent A;3869 2z7j.ent A 2p0j.ent A;3870 2z7j.ent A 2is9.ent A;3871 2z7j.ent A 2eyi.ent A;3872 2z7j.ent A 2cga.ent A;3873 2z7j.ent A 1ym0.ent A;3874 2z7j.ent A 1twf.ent E;3875 2z7j.ent A 1rc9.ent A;3876 2z7j.ent A 1pn9.ent A;3877 2z7j.ent A 1n7k.ent A;3878 2z7j.ent A 1kg5.ent A;3879 2z7j.ent A 1gis.ent A;3880 2z7j.ent A 1fuj.ent A;3881 2z7j.ent A 1del.ent A;3882 2z7j.ent A 1b77.ent A;3883 2z7j.ent A 4h6a.ent A;3884 2z7j.ent A 4a25.ent B;3885 2z7j.ent A 3q5v.ent A;3886 2z7j.ent A 3nmr.ent A;3887 2z7j.ent A 3kjz.ent A;3888 2z7j.ent A 3g5k.ent A;3889 2z7j.ent A 3csr.ent A;3890 2z7j.ent A 2zk9.ent X;3891 2z7j.ent A 2wmm.ent A;3892 2z7j.ent A 2q7a.ent A;3893 2z7j.ent A 2ill.ent A;3894 2z7j.ent A 2fko.ent A;3895 2z7j.ent A 2cut.ent A;3896 2z7j.ent A 1zv9.ent A;3897 2z7j.ent A 1xt0.ent B;3898 2z7j.ent A 1u7p.ent A;3899 2z7j.ent A 1r8n.ent A;3900 2z7j.ent A 1ocy.ent A;3901 2z7j.ent A 1m4d.ent A;3902 2z7j.ent A 1iqq.ent A;3903 2z7j.ent A 1gui.ent A;3904 2z7j.ent A 1ffk.ent T;3905 2z7j.ent A 1dow.ent A;3906 2z7j.ent A 1cau.ent A;3907 2z7j.ent A 1ava.ent C;3908 2z7j.ent A 4fei.ent A;3909 2z7j.ent A 3zqx.ent A;3910 2z7j.ent A 3sek.ent B;3911 2z7j.ent A 3obl.ent A;3912 2z7j.ent A 3mao.ent A;3913 2z7j.ent A 3kk4.ent A;3914 2z7j.ent A 3gef.ent A;3915 2z7j.ent A 3ent.ent A;3916 2z7j.ent A 3bdq.ent B;3917 2w80.ent A 5mdh.ent A;3918 2w80.ent A 3two.ent B;3919 2w80.ent A 3n9s.ent A;3920 2w80.ent A 3exe.ent B;3921 2w80.ent A 2yy7.ent A;3922 2w80.ent A 2qx3.ent A;3923 2w80.ent A 2hk2.ent A;3924 2w80.ent A 2eih.ent A;3925 2w80.ent A 1yg9.ent A;3926 2w80.ent A 1tca.ent A;3927 2w80.ent A 1pxz.ent A;3928 2w80.ent A 1llp.ent A;3929 2w80.ent A 1h6u.ent A;3930 2w80.ent A 1ea7.ent A;3931 2w80.ent A 1a4m.ent A;3932 2w80.ent A 4b5w.ent A;3933 2w80.ent A 3q1n.ent A;3934 2w80.ent A 3m66.ent A;3935 2w80.ent A 3gae.ent A;3936 2w80.ent A 3bcz.ent A;3937 2w80.ent A 2voa.ent A;3938 2w80.ent A 2p9h.ent A;3939 2w80.ent A 2gnu.ent M;3940 2w80.ent A 2ci1.ent A;3941 2w80.ent A 1xq6.ent A;3942 2w80.ent A 1tml.ent A;3943 2w80.ent A 1qgj.ent A;3944 2w80.ent A 1nas.ent A;3945 2w80.ent A 1hvq.ent A;3946 2w80.ent A 1fts.ent A;3947 2w80.ent A 1dea.ent A;3948 2w80.ent A 1b35.ent B;3949 2w80.ent A 1a0g.ent B;3950 2w80.ent A 3tt9.ent A;3951 2w80.ent A 3pnl.ent B;3952 2w80.ent A 3kvd.ent D;3953 2w80.ent A 3fj7.ent A;3954 2w80.ent A 3cjj.ent A;3955 2w80.ent A 3ai9.ent X;3956 2w80.ent A 2vg9.ent A;3957 2w80.ent A 2p0j.ent A;3958 2w80.ent A 2is9.ent A;3959 2w80.ent A 2eyi.ent A;3960 2w80.ent A 2cga.ent A;3961 2w80.ent A 1ym0.ent A;3962 2w80.ent A 1twf.ent E;3963 2w80.ent A 1rc9.ent A;3964 2w80.ent A 1pn9.ent A;3965 2w80.ent A 1n7k.ent A;3966 2w80.ent A 1kg5.ent A;3967 2w80.ent A 1gis.ent A;3968 2w80.ent A 1fuj.ent A;3969 2w80.ent A 1del.ent A;3970 2w80.ent A 1b77.ent A;3971 2w80.ent A 4h6a.ent A;3972 2w80.ent A 4a25.ent B;3973 2w80.ent A 3q5v.ent A;3974 2w80.ent A 3nmr.ent A;3975 2w80.ent A 3kjz.ent A;3976 2w80.ent A 3g5k.ent A;3977 2w80.ent A 3csr.ent A;3978 2w80.ent A 2zk9.ent X;3979 2w80.ent A 2wmm.ent A;3980 2w80.ent A 2q7a.ent A;3981 2w80.ent A 2ill.ent A;3982 2w80.ent A 2fko.ent A;3983 2w80.ent A 2cut.ent A;3984 2w80.ent A 1zv9.ent A;3985 2w80.ent A 1xt0.ent B;3986 2w80.ent A 1u7p.ent A;3987 2w80.ent A 1r8n.ent A;3988 2w80.ent A 1ocy.ent A;3989 2w80.ent A 1m4d.ent A;3990 2w80.ent A 1iqq.ent A;3991 2w80.ent A 1gui.ent A;3992 2w80.ent A 1ffk.ent T;3993 2w80.ent A 1dow.ent A;3994 2w80.ent A 1cau.ent A;3995 2w80.ent A 1ava.ent C;3996 2w80.ent A 4fei.ent A;3997 2w80.ent A 3zqx.ent A;3998 2w80.ent A 3sek.ent B;3999 2w80.ent A 3obl.ent A;4000 2w80.ent A 3mao.ent A;4001 2w80.ent A 3kk4.ent A;4002 2w80.ent A 3gef.ent A;4003 2w80.ent A 3ent.ent A;4004 2w80.ent A 3bdq.ent B;4005 2w80.ent A 2z7j.ent A;4006 2rcq.ent A 5mdh.ent A;4007 2rcq.ent A 3two.ent B;4008 2rcq.ent A 3n9s.ent A;4009 2rcq.ent A 3exe.ent B;4010 2rcq.ent A 2yy7.ent A;4011 2rcq.ent A 2qx3.ent A;4012 2rcq.ent A 2hk2.ent A;4013 2rcq.ent A 2eih.ent A;4014 2rcq.ent A 1yg9.ent A;4015 2rcq.ent A 1tca.ent A;4016 2rcq.ent A 1pxz.ent A;4017 2rcq.ent A 1llp.ent A;4018 2rcq.ent A 1h6u.ent A;4019 2rcq.ent A 1ea7.ent A;4020 2rcq.ent A 1a4m.ent A;4021 2rcq.ent A 4b5w.ent A;4022 2rcq.ent A 3q1n.ent A;4023 2rcq.ent A 3m66.ent A;4024 2rcq.ent A 3gae.ent A;4025 2rcq.ent A 3bcz.ent A;4026 2rcq.ent A 2voa.ent A;4027 2rcq.ent A 2p9h.ent A;4028 2rcq.ent A 2gnu.ent M;4029 2rcq.ent A 2ci1.ent A;4030 2rcq.ent A 1xq6.ent A;4031 2rcq.ent A 1tml.ent A;4032 2rcq.ent A 1qgj.ent A;4033 2rcq.ent A 1nas.ent A;4034 2rcq.ent A 1hvq.ent A;4035 2rcq.ent A 1fts.ent A;4036 2rcq.ent A 1dea.ent A;4037 2rcq.ent A 1b35.ent B;4038 2rcq.ent A 1a0g.ent B;4039 2rcq.ent A 3tt9.ent A;4040 2rcq.ent A 3pnl.ent B;4041 2rcq.ent A 3kvd.ent D;4042 2rcq.ent A 3fj7.ent A;4043 2rcq.ent A 3cjj.ent A;4044 2rcq.ent A 3ai9.ent X;4045 2rcq.ent A 2vg9.ent A;4046 2rcq.ent A 2p0j.ent A;4047 2rcq.ent A 2is9.ent A;4048 2rcq.ent A 2eyi.ent A;4049 2rcq.ent A 2cga.ent A;4050 2rcq.ent A 1ym0.ent A;4051 2rcq.ent A 1twf.ent E;4052 2rcq.ent A 1rc9.ent A;4053 2rcq.ent A 1pn9.ent A;4054 2rcq.ent A 1n7k.ent A;4055 2rcq.ent A 1kg5.ent A;4056 2rcq.ent A 1gis.ent A;4057 2rcq.ent A 1fuj.ent A;4058 2rcq.ent A 1del.ent A;4059 2rcq.ent A 1b77.ent A;4060 2rcq.ent A 4h6a.ent A;4061 2rcq.ent A 4a25.ent B;4062 2rcq.ent A 3q5v.ent A;4063 2rcq.ent A 3nmr.ent A;4064 2rcq.ent A 3kjz.ent A;4065 2rcq.ent A 3g5k.ent A;4066 2rcq.ent A 3csr.ent A;4067 2rcq.ent A 2zk9.ent X;4068 2rcq.ent A 2wmm.ent A;4069 2rcq.ent A 2q7a.ent A;4070 2rcq.ent A 2ill.ent A;4071 2rcq.ent A 2fko.ent A;4072 2rcq.ent A 2cut.ent A;4073 2rcq.ent A 1zv9.ent A;4074 2rcq.ent A 1xt0.ent B;4075 2rcq.ent A 1u7p.ent A;4076 2rcq.ent A 1r8n.ent A;4077 2rcq.ent A 1ocy.ent A;4078 2rcq.ent A 1m4d.ent A;4079 2rcq.ent A 1iqq.ent A;4080 2rcq.ent A 1gui.ent A;4081 2rcq.ent A 1ffk.ent T;4082 2rcq.ent A 1dow.ent A;4083 2rcq.ent A 1cau.ent A;4084 2rcq.ent A 1ava.ent C;4085 2rcq.ent A 4fei.ent A;4086 2rcq.ent A 3zqx.ent A;4087 2rcq.ent A 3sek.ent B;4088 2rcq.ent A 3obl.ent A;4089 2rcq.ent A 3mao.ent A;4090 2rcq.ent A 3kk4.ent A;4091 2rcq.ent A 3gef.ent A;4092 2rcq.ent A 3ent.ent A;4093 2rcq.ent A 3bdq.ent B;4094 2rcq.ent A 2z7j.ent A;4095 2rcq.ent A 2w80.ent A;4096 2p8v.ent A 5mdh.ent A;4097 2p8v.ent A 3two.ent B;4098 2p8v.ent A 3n9s.ent A;4099 2p8v.ent A 3exe.ent B;4100 2p8v.ent A 2yy7.ent A;4101 2p8v.ent A 2qx3.ent A;4102 2p8v.ent A 2hk2.ent A;4103 2p8v.ent A 2eih.ent A;4104 2p8v.ent A 1yg9.ent A;4105 2p8v.ent A 1tca.ent A;4106 2p8v.ent A 1pxz.ent A;4107 2p8v.ent A 1llp.ent A;4108 2p8v.ent A 1h6u.ent A;4109 2p8v.ent A 1ea7.ent A;4110 2p8v.ent A 1a4m.ent A;4111 2p8v.ent A 4b5w.ent A;4112 2p8v.ent A 3q1n.ent A;4113 2p8v.ent A 3m66.ent A;4114 2p8v.ent A 3gae.ent A;4115 2p8v.ent A 3bcz.ent A;4116 2p8v.ent A 2voa.ent A;4117 2p8v.ent A 2p9h.ent A;4118 2p8v.ent A 2gnu.ent M;4119 2p8v.ent A 2ci1.ent A;4120 2p8v.ent A 1xq6.ent A;4121 2p8v.ent A 1tml.ent A;4122 2p8v.ent A 1qgj.ent A;4123 2p8v.ent A 1nas.ent A;4124 2p8v.ent A 1hvq.ent A;4125 2p8v.ent A 1fts.ent A;4126 2p8v.ent A 1dea.ent A;4127 2p8v.ent A 1b35.ent B;4128 2p8v.ent A 1a0g.ent B;4129 2p8v.ent A 3tt9.ent A;4130 2p8v.ent A 3pnl.ent B;4131 2p8v.ent A 3kvd.ent D;4132 2p8v.ent A 3fj7.ent A;4133 2p8v.ent A 3cjj.ent A;4134 2p8v.ent A 3ai9.ent X;4135 2p8v.ent A 2vg9.ent A;4136 2p8v.ent A 2p0j.ent A;4137 2p8v.ent A 2is9.ent A;4138 2p8v.ent A 2eyi.ent A;4139 2p8v.ent A 2cga.ent A;4140 2p8v.ent A 1ym0.ent A;4141 2p8v.ent A 1twf.ent E;4142 2p8v.ent A 1rc9.ent A;4143 2p8v.ent A 1pn9.ent A;4144 2p8v.ent A 1n7k.ent A;4145 2p8v.ent A 1kg5.ent A;4146 2p8v.ent A 1gis.ent A;4147 2p8v.ent A 1fuj.ent A;4148 2p8v.ent A 1del.ent A;4149 2p8v.ent A 1b77.ent A;4150 2p8v.ent A 4h6a.ent A;4151 2p8v.ent A 4a25.ent B;4152 2p8v.ent A 3q5v.ent A;4153 2p8v.ent A 3nmr.ent A;4154 2p8v.ent A 3kjz.ent A;4155 2p8v.ent A 3g5k.ent A;4156 2p8v.ent A 3csr.ent A;4157 2p8v.ent A 2zk9.ent X;4158 2p8v.ent A 2wmm.ent A;4159 2p8v.ent A 2q7a.ent A;4160 2p8v.ent A 2ill.ent A;4161 2p8v.ent A 2fko.ent A;4162 2p8v.ent A 2cut.ent A;4163 2p8v.ent A 1zv9.ent A;4164 2p8v.ent A 1xt0.ent B;4165 2p8v.ent A 1u7p.ent A;4166 2p8v.ent A 1r8n.ent A;4167 2p8v.ent A 1ocy.ent A;4168 2p8v.ent A 1m4d.ent A;4169 2p8v.ent A 1iqq.ent A;4170 2p8v.ent A 1gui.ent A;4171 2p8v.ent A 1ffk.ent T;4172 2p8v.ent A 1dow.ent A;4173 2p8v.ent A 1cau.ent A;4174 2p8v.ent A 1ava.ent C;4175 2p8v.ent A 4fei.ent A;4176 2p8v.ent A 3zqx.ent A;4177 2p8v.ent A 3sek.ent B;4178 2p8v.ent A 3obl.ent A;4179 2p8v.ent A 3mao.ent A;4180 2p8v.ent A 3kk4.ent A;4181 2p8v.ent A 3gef.ent A;4182 2p8v.ent A 3ent.ent A;4183 2p8v.ent A 3bdq.ent B;4184 2p8v.ent A 2z7j.ent A;4185 2p8v.ent A 2w80.ent A;4186 2p8v.ent A 2rcq.ent A;4187 2jj6.ent A 5mdh.ent A;4188 2jj6.ent A 3two.ent B;4189 2jj6.ent A 3n9s.ent A;4190 2jj6.ent A 3exe.ent B;4191 2jj6.ent A 2yy7.ent A;4192 2jj6.ent A 2qx3.ent A;4193 2jj6.ent A 2hk2.ent A;4194 2jj6.ent A 2eih.ent A;4195 2jj6.ent A 1yg9.ent A;4196 2jj6.ent A 1tca.ent A;4197 2jj6.ent A 1pxz.ent A;4198 2jj6.ent A 1llp.ent A;4199 2jj6.ent A 1h6u.ent A;4200 2jj6.ent A 1ea7.ent A;4201 2jj6.ent A 1a4m.ent A;4202 2jj6.ent A 4b5w.ent A;4203 2jj6.ent A 3q1n.ent A;4204 2jj6.ent A 3m66.ent A;4205 2jj6.ent A 3gae.ent A;4206 2jj6.ent A 3bcz.ent A;4207 2jj6.ent A 2voa.ent A;4208 2jj6.ent A 2p9h.ent A;4209 2jj6.ent A 2gnu.ent M;4210 2jj6.ent A 2ci1.ent A;4211 2jj6.ent A 1xq6.ent A;4212 2jj6.ent A 1tml.ent A;4213 2jj6.ent A 1qgj.ent A;4214 2jj6.ent A 1nas.ent A;4215 2jj6.ent A 1hvq.ent A;4216 2jj6.ent A 1fts.ent A;4217 2jj6.ent A 1dea.ent A;4218 2jj6.ent A 1b35.ent B;4219 2jj6.ent A 1a0g.ent B;4220 2jj6.ent A 3tt9.ent A;4221 2jj6.ent A 3pnl.ent B;4222 2jj6.ent A 3kvd.ent D;4223 2jj6.ent A 3fj7.ent A;4224 2jj6.ent A 3cjj.ent A;4225 2jj6.ent A 3ai9.ent X;4226 2jj6.ent A 2vg9.ent A;4227 2jj6.ent A 2p0j.ent A;4228 2jj6.ent A 2is9.ent A;4229 2jj6.ent A 2eyi.ent A;4230 2jj6.ent A 2cga.ent A;4231 2jj6.ent A 1ym0.ent A;4232 2jj6.ent A 1twf.ent E;4233 2jj6.ent A 1rc9.ent A;4234 2jj6.ent A 1pn9.ent A;4235 2jj6.ent A 1n7k.ent A;4236 2jj6.ent A 1kg5.ent A;4237 2jj6.ent A 1gis.ent A;4238 2jj6.ent A 1fuj.ent A;4239 2jj6.ent A 1del.ent A;4240 2jj6.ent A 1b77.ent A;4241 2jj6.ent A 4h6a.ent A;4242 2jj6.ent A 4a25.ent B;4243 2jj6.ent A 3q5v.ent A;4244 2jj6.ent A 3nmr.ent A;4245 2jj6.ent A 3kjz.ent A;4246 2jj6.ent A 3g5k.ent A;4247 2jj6.ent A 3csr.ent A;4248 2jj6.ent A 2zk9.ent X;4249 2jj6.ent A 2wmm.ent A;4250 2jj6.ent A 2q7a.ent A;4251 2jj6.ent A 2ill.ent A;4252 2jj6.ent A 2fko.ent A;4253 2jj6.ent A 2cut.ent A;4254 2jj6.ent A 1zv9.ent A;4255 2jj6.ent A 1xt0.ent B;4256 2jj6.ent A 1u7p.ent A;4257 2jj6.ent A 1r8n.ent A;4258 2jj6.ent A 1ocy.ent A;4259 2jj6.ent A 1m4d.ent A;4260 2jj6.ent A 1iqq.ent A;4261 2jj6.ent A 1gui.ent A;4262 2jj6.ent A 1ffk.ent T;4263 2jj6.ent A 1dow.ent A;4264 2jj6.ent A 1cau.ent A;4265 2jj6.ent A 1ava.ent C;4266 2jj6.ent A 4fei.ent A;4267 2jj6.ent A 3zqx.ent A;4268 2jj6.ent A 3sek.ent B;4269 2jj6.ent A 3obl.ent A;4270 2jj6.ent A 3mao.ent A;4271 2jj6.ent A 3kk4.ent A;4272 2jj6.ent A 3gef.ent A;4273 2jj6.ent A 3ent.ent A;4274 2jj6.ent A 3bdq.ent B;4275 2jj6.ent A 2z7j.ent A;4276 2jj6.ent A 2w80.ent A;4277 2jj6.ent A 2rcq.ent A;4278 2jj6.ent A 2p8v.ent A;4279 2hnx.ent A 5mdh.ent A;4280 2hnx.ent A 3two.ent B;4281 2hnx.ent A 3n9s.ent A;4282 2hnx.ent A 3exe.ent B;4283 2hnx.ent A 2yy7.ent A;4284 2hnx.ent A 2qx3.ent A;4285 2hnx.ent A 2hk2.ent A;4286 2hnx.ent A 2eih.ent A;4287 2hnx.ent A 1yg9.ent A;4288 2hnx.ent A 1tca.ent A;4289 2hnx.ent A 1pxz.ent A;4290 2hnx.ent A 1llp.ent A;4291 2hnx.ent A 1h6u.ent A;4292 2hnx.ent A 1ea7.ent A;4293 2hnx.ent A 1a4m.ent A;4294 2hnx.ent A 4b5w.ent A;4295 2hnx.ent A 3q1n.ent A;4296 2hnx.ent A 3m66.ent A;4297 2hnx.ent A 3gae.ent A;4298 2hnx.ent A 3bcz.ent A;4299 2hnx.ent A 2voa.ent A;4300 2hnx.ent A 2p9h.ent A;4301 2hnx.ent A 2gnu.ent M;4302 2hnx.ent A 2ci1.ent A;4303 2hnx.ent A 1xq6.ent A;4304 2hnx.ent A 1tml.ent A;4305 2hnx.ent A 1qgj.ent A;4306 2hnx.ent A 1nas.ent A;4307 2hnx.ent A 1hvq.ent A;4308 2hnx.ent A 1fts.ent A;4309 2hnx.ent A 1dea.ent A;4310 2hnx.ent A 1b35.ent B;4311 2hnx.ent A 1a0g.ent B;4312 2hnx.ent A 3tt9.ent A;4313 2hnx.ent A 3pnl.ent B;4314 2hnx.ent A 3kvd.ent D;4315 2hnx.ent A 3fj7.ent A;4316 2hnx.ent A 3cjj.ent A;4317 2hnx.ent A 3ai9.ent X;4318 2hnx.ent A 2vg9.ent A;4319 2hnx.ent A 2p0j.ent A;4320 2hnx.ent A 2is9.ent A;4321 2hnx.ent A 2eyi.ent A;4322 2hnx.ent A 2cga.ent A;4323 2hnx.ent A 1ym0.ent A;4324 2hnx.ent A 1twf.ent E;4325 2hnx.ent A 1rc9.ent A;4326 2hnx.ent A 1pn9.ent A;4327 2hnx.ent A 1n7k.ent A;4328 2hnx.ent A 1kg5.ent A;4329 2hnx.ent A 1gis.ent A;4330 2hnx.ent A 1fuj.ent A;4331 2hnx.ent A 1del.ent A;4332 2hnx.ent A 1b77.ent A;4333 2hnx.ent A 4h6a.ent A;4334 2hnx.ent A 4a25.ent B;4335 2hnx.ent A 3q5v.ent A;4336 2hnx.ent A 3nmr.ent A;4337 2hnx.ent A 3kjz.ent A;4338 2hnx.ent A 3g5k.ent A;4339 2hnx.ent A 3csr.ent A;4340 2hnx.ent A 2zk9.ent X;4341 2hnx.ent A 2wmm.ent A;4342 2hnx.ent A 2q7a.ent A;4343 2hnx.ent A 2ill.ent A;4344 2hnx.ent A 2fko.ent A;4345 2hnx.ent A 2cut.ent A;4346 2hnx.ent A 1zv9.ent A;4347 2hnx.ent A 1xt0.ent B;4348 2hnx.ent A 1u7p.ent A;4349 2hnx.ent A 1r8n.ent A;4350 2hnx.ent A 1ocy.ent A;4351 2hnx.ent A 1m4d.ent A;4352 2hnx.ent A 1iqq.ent A;4353 2hnx.ent A 1gui.ent A;4354 2hnx.ent A 1ffk.ent T;4355 2hnx.ent A 1dow.ent A;4356 2hnx.ent A 1cau.ent A;4357 2hnx.ent A 1ava.ent C;4358 2hnx.ent A 4fei.ent A;4359 2hnx.ent A 3zqx.ent A;4360 2hnx.ent A 3sek.ent B;4361 2hnx.ent A 3obl.ent A;4362 2hnx.ent A 3mao.ent A;4363 2hnx.ent A 3kk4.ent A;4364 2hnx.ent A 3gef.ent A;4365 2hnx.ent A 3ent.ent A;4366 2hnx.ent A 3bdq.ent B;4367 2hnx.ent A 2z7j.ent A;4368 2hnx.ent A 2w80.ent A;4369 2hnx.ent A 2rcq.ent A;4370 2hnx.ent A 2p8v.ent A;4371 2hnx.ent A 2jj6.ent A;4372 2f06.ent A 5mdh.ent A;4373 2f06.ent A 3two.ent B;4374 2f06.ent A 3n9s.ent A;4375 2f06.ent A 3exe.ent B;4376 2f06.ent A 2yy7.ent A;4377 2f06.ent A 2qx3.ent A;4378 2f06.ent A 2hk2.ent A;4379 2f06.ent A 2eih.ent A;4380 2f06.ent A 1yg9.ent A;4381 2f06.ent A 1tca.ent A;4382 2f06.ent A 1pxz.ent A;4383 2f06.ent A 1llp.ent A;4384 2f06.ent A 1h6u.ent A;4385 2f06.ent A 1ea7.ent A;4386 2f06.ent A 1a4m.ent A;4387 2f06.ent A 4b5w.ent A;4388 2f06.ent A 3q1n.ent A;4389 2f06.ent A 3m66.ent A;4390 2f06.ent A 3gae.ent A;4391 2f06.ent A 3bcz.ent A;4392 2f06.ent A 2voa.ent A;4393 2f06.ent A 2p9h.ent A;4394 2f06.ent A 2gnu.ent M;4395 2f06.ent A 2ci1.ent A;4396 2f06.ent A 1xq6.ent A;4397 2f06.ent A 1tml.ent A;4398 2f06.ent A 1qgj.ent A;4399 2f06.ent A 1nas.ent A;4400 2f06.ent A 1hvq.ent A;4401 2f06.ent A 1fts.ent A;4402 2f06.ent A 1dea.ent A;4403 2f06.ent A 1b35.ent B;4404 2f06.ent A 1a0g.ent B;4405 2f06.ent A 3tt9.ent A;4406 2f06.ent A 3pnl.ent B;4407 2f06.ent A 3kvd.ent D;4408 2f06.ent A 3fj7.ent A;4409 2f06.ent A 3cjj.ent A;4410 2f06.ent A 3ai9.ent X;4411 2f06.ent A 2vg9.ent A;4412 2f06.ent A 2p0j.ent A;4413 2f06.ent A 2is9.ent A;4414 2f06.ent A 2eyi.ent A;4415 2f06.ent A 2cga.ent A;4416 2f06.ent A 1ym0.ent A;4417 2f06.ent A 1twf.ent E;4418 2f06.ent A 1rc9.ent A;4419 2f06.ent A 1pn9.ent A;4420 2f06.ent A 1n7k.ent A;4421 2f06.ent A 1kg5.ent A;4422 2f06.ent A 1gis.ent A;4423 2f06.ent A 1fuj.ent A;4424 2f06.ent A 1del.ent A;4425 2f06.ent A 1b77.ent A;4426 2f06.ent A 4h6a.ent A;4427 2f06.ent A 4a25.ent B;4428 2f06.ent A 3q5v.ent A;4429 2f06.ent A 3nmr.ent A;4430 2f06.ent A 3kjz.ent A;4431 2f06.ent A 3g5k.ent A;4432 2f06.ent A 3csr.ent A;4433 2f06.ent A 2zk9.ent X;4434 2f06.ent A 2wmm.ent A;4435 2f06.ent A 2q7a.ent A;4436 2f06.ent A 2ill.ent A;4437 2f06.ent A 2fko.ent A;4438 2f06.ent A 2cut.ent A;4439 2f06.ent A 1zv9.ent A;4440 2f06.ent A 1xt0.ent B;4441 2f06.ent A 1u7p.ent A;4442 2f06.ent A 1r8n.ent A;4443 2f06.ent A 1ocy.ent A;4444 2f06.ent A 1m4d.ent A;4445 2f06.ent A 1iqq.ent A;4446 2f06.ent A 1gui.ent A;4447 2f06.ent A 1ffk.ent T;4448 2f06.ent A 1dow.ent A;4449 2f06.ent A 1cau.ent A;4450 2f06.ent A 1ava.ent C;4451 2f06.ent A 4fei.ent A;4452 2f06.ent A 3zqx.ent A;4453 2f06.ent A 3sek.ent B;4454 2f06.ent A 3obl.ent A;4455 2f06.ent A 3mao.ent A;4456 2f06.ent A 3kk4.ent A;4457 2f06.ent A 3gef.ent A;4458 2f06.ent A 3ent.ent A;4459 2f06.ent A 3bdq.ent B;4460 2f06.ent A 2z7j.ent A;4461 2f06.ent A 2w80.ent A;4462 2f06.ent A 2rcq.ent A;4463 2f06.ent A 2p8v.ent A;4464 2f06.ent A 2jj6.ent A;4465 2f06.ent A 2hnx.ent A;4466 2d00.ent A 5mdh.ent A;4467 2d00.ent A 3two.ent B;4468 2d00.ent A 3n9s.ent A;4469 2d00.ent A 3exe.ent B;4470 2d00.ent A 2yy7.ent A;4471 2d00.ent A 2qx3.ent A;4472 2d00.ent A 2hk2.ent A;4473 2d00.ent A 2eih.ent A;4474 2d00.ent A 1yg9.ent A;4475 2d00.ent A 1tca.ent A;4476 2d00.ent A 1pxz.ent A;4477 2d00.ent A 1llp.ent A;4478 2d00.ent A 1h6u.ent A;4479 2d00.ent A 1ea7.ent A;4480 2d00.ent A 1a4m.ent A;4481 2d00.ent A 4b5w.ent A;4482 2d00.ent A 3q1n.ent A;4483 2d00.ent A 3m66.ent A;4484 2d00.ent A 3gae.ent A;4485 2d00.ent A 3bcz.ent A;4486 2d00.ent A 2voa.ent A;4487 2d00.ent A 2p9h.ent A;4488 2d00.ent A 2gnu.ent M;4489 2d00.ent A 2ci1.ent A;4490 2d00.ent A 1xq6.ent A;4491 2d00.ent A 1tml.ent A;4492 2d00.ent A 1qgj.ent A;4493 2d00.ent A 1nas.ent A;4494 2d00.ent A 1hvq.ent A;4495 2d00.ent A 1fts.ent A;4496 2d00.ent A 1dea.ent A;4497 2d00.ent A 1b35.ent B;4498 2d00.ent A 1a0g.ent B;4499 2d00.ent A 3tt9.ent A;4500 2d00.ent A 3pnl.ent B;4501 2d00.ent A 3kvd.ent D;4502 2d00.ent A 3fj7.ent A;4503 2d00.ent A 3cjj.ent A;4504 2d00.ent A 3ai9.ent X;4505 2d00.ent A 2vg9.ent A;4506 2d00.ent A 2p0j.ent A;4507 2d00.ent A 2is9.ent A;4508 2d00.ent A 2eyi.ent A;4509 2d00.ent A 2cga.ent A;4510 2d00.ent A 1ym0.ent A;4511 2d00.ent A 1twf.ent E;4512 2d00.ent A 1rc9.ent A;4513 2d00.ent A 1pn9.ent A;4514 2d00.ent A 1n7k.ent A;4515 2d00.ent A 1kg5.ent A;4516 2d00.ent A 1gis.ent A;4517 2d00.ent A 1fuj.ent A;4518 2d00.ent A 1del.ent A;4519 2d00.ent A 1b77.ent A;4520 2d00.ent A 4h6a.ent A;4521 2d00.ent A 4a25.ent B;4522 2d00.ent A 3q5v.ent A;4523 2d00.ent A 3nmr.ent A;4524 2d00.ent A 3kjz.ent A;4525 2d00.ent A 3g5k.ent A;4526 2d00.ent A 3csr.ent A;4527 2d00.ent A 2zk9.ent X;4528 2d00.ent A 2wmm.ent A;4529 2d00.ent A 2q7a.ent A;4530 2d00.ent A 2ill.ent A;4531 2d00.ent A 2fko.ent A;4532 2d00.ent A 2cut.ent A;4533 2d00.ent A 1zv9.ent A;4534 2d00.ent A 1xt0.ent B;4535 2d00.ent A 1u7p.ent A;4536 2d00.ent A 1r8n.ent A;4537 2d00.ent A 1ocy.ent A;4538 2d00.ent A 1m4d.ent A;4539 2d00.ent A 1iqq.ent A;4540 2d00.ent A 1gui.ent A;4541 2d00.ent A 1ffk.ent T;4542 2d00.ent A 1dow.ent A;4543 2d00.ent A 1cau.ent A;4544 2d00.ent A 1ava.ent C;4545 2d00.ent A 4fei.ent A;4546 2d00.ent A 3zqx.ent A;4547 2d00.ent A 3sek.ent B;4548 2d00.ent A 3obl.ent A;4549 2d00.ent A 3mao.ent A;4550 2d00.ent A 3kk4.ent A;4551 2d00.ent A 3gef.ent A;4552 2d00.ent A 3ent.ent A;4553 2d00.ent A 3bdq.ent B;4554 2d00.ent A 2z7j.ent A;4555 2d00.ent A 2w80.ent A;4556 2d00.ent A 2rcq.ent A;4557 2d00.ent A 2p8v.ent A;4558 2d00.ent A 2jj6.ent A;4559 2d00.ent A 2hnx.ent A;4560 2d00.ent A 2f06.ent A;4561 2awg.ent A 5mdh.ent A;4562 2awg.ent A 3two.ent B;4563 2awg.ent A 3n9s.ent A;4564 2awg.ent A 3exe.ent B;4565 2awg.ent A 2yy7.ent A;4566 2awg.ent A 2qx3.ent A;4567 2awg.ent A 2hk2.ent A;4568 2awg.ent A 2eih.ent A;4569 2awg.ent A 1yg9.ent A;4570 2awg.ent A 1tca.ent A;4571 2awg.ent A 1pxz.ent A;4572 2awg.ent A 1llp.ent A;4573 2awg.ent A 1h6u.ent A;4574 2awg.ent A 1ea7.ent A;4575 2awg.ent A 1a4m.ent A;4576 2awg.ent A 4b5w.ent A;4577 2awg.ent A 3q1n.ent A;4578 2awg.ent A 3m66.ent A;4579 2awg.ent A 3gae.ent A;4580 2awg.ent A 3bcz.ent A;4581 2awg.ent A 2voa.ent A;4582 2awg.ent A 2p9h.ent A;4583 2awg.ent A 2gnu.ent M;4584 2awg.ent A 2ci1.ent A;4585 2awg.ent A 1xq6.ent A;4586 2awg.ent A 1tml.ent A;4587 2awg.ent A 1qgj.ent A;4588 2awg.ent A 1nas.ent A;4589 2awg.ent A 1hvq.ent A;4590 2awg.ent A 1fts.ent A;4591 2awg.ent A 1dea.ent A;4592 2awg.ent A 1b35.ent B;4593 2awg.ent A 1a0g.ent B;4594 2awg.ent A 3tt9.ent A;4595 2awg.ent A 3pnl.ent B;4596 2awg.ent A 3kvd.ent D;4597 2awg.ent A 3fj7.ent A;4598 2awg.ent A 3cjj.ent A;4599 2awg.ent A 3ai9.ent X;4600 2awg.ent A 2vg9.ent A;4601 2awg.ent A 2p0j.ent A;4602 2awg.ent A 2is9.ent A;4603 2awg.ent A 2eyi.ent A;4604 2awg.ent A 2cga.ent A;4605 2awg.ent A 1ym0.ent A;4606 2awg.ent A 1twf.ent E;4607 2awg.ent A 1rc9.ent A;4608 2awg.ent A 1pn9.ent A;4609 2awg.ent A 1n7k.ent A;4610 2awg.ent A 1kg5.ent A;4611 2awg.ent A 1gis.ent A;4612 2awg.ent A 1fuj.ent A;4613 2awg.ent A 1del.ent A;4614 2awg.ent A 1b77.ent A;4615 2awg.ent A 4h6a.ent A;4616 2awg.ent A 4a25.ent B;4617 2awg.ent A 3q5v.ent A;4618 2awg.ent A 3nmr.ent A;4619 2awg.ent A 3kjz.ent A;4620 2awg.ent A 3g5k.ent A;4621 2awg.ent A 3csr.ent A;4622 2awg.ent A 2zk9.ent X;4623 2awg.ent A 2wmm.ent A;4624 2awg.ent A 2q7a.ent A;4625 2awg.ent A 2ill.ent A;4626 2awg.ent A 2fko.ent A;4627 2awg.ent A 2cut.ent A;4628 2awg.ent A 1zv9.ent A;4629 2awg.ent A 1xt0.ent B;4630 2awg.ent A 1u7p.ent A;4631 2awg.ent A 1r8n.ent A;4632 2awg.ent A 1ocy.ent A;4633 2awg.ent A 1m4d.ent A;4634 2awg.ent A 1iqq.ent A;4635 2awg.ent A 1gui.ent A;4636 2awg.ent A 1ffk.ent T;4637 2awg.ent A 1dow.ent A;4638 2awg.ent A 1cau.ent A;4639 2awg.ent A 1ava.ent C;4640 2awg.ent A 4fei.ent A;4641 2awg.ent A 3zqx.ent A;4642 2awg.ent A 3sek.ent B;4643 2awg.ent A 3obl.ent A;4644 2awg.ent A 3mao.ent A;4645 2awg.ent A 3kk4.ent A;4646 2awg.ent A 3gef.ent A;4647 2awg.ent A 3ent.ent A;4648 2awg.ent A 3bdq.ent B;4649 2awg.ent A 2z7j.ent A;4650 2awg.ent A 2w80.ent A;4651 2awg.ent A 2rcq.ent A;4652 2awg.ent A 2p8v.ent A;4653 2awg.ent A 2jj6.ent A;4654 2awg.ent A 2hnx.ent A;4655 2awg.ent A 2f06.ent A;4656 2awg.ent A 2d00.ent A;4657 1yq5.ent A 5mdh.ent A;4658 1yq5.ent A 3two.ent B;4659 1yq5.ent A 3n9s.ent A;4660 1yq5.ent A 3exe.ent B;4661 1yq5.ent A 2yy7.ent A;4662 1yq5.ent A 2qx3.ent A;4663 1yq5.ent A 2hk2.ent A;4664 1yq5.ent A 2eih.ent A;4665 1yq5.ent A 1yg9.ent A;4666 1yq5.ent A 1tca.ent A;4667 1yq5.ent A 1pxz.ent A;4668 1yq5.ent A 1llp.ent A;4669 1yq5.ent A 1h6u.ent A;4670 1yq5.ent A 1ea7.ent A;4671 1yq5.ent A 1a4m.ent A;4672 1yq5.ent A 4b5w.ent A;4673 1yq5.ent A 3q1n.ent A;4674 1yq5.ent A 3m66.ent A;4675 1yq5.ent A 3gae.ent A;4676 1yq5.ent A 3bcz.ent A;4677 1yq5.ent A 2voa.ent A;4678 1yq5.ent A 2p9h.ent A;4679 1yq5.ent A 2gnu.ent M;4680 1yq5.ent A 2ci1.ent A;4681 1yq5.ent A 1xq6.ent A;4682 1yq5.ent A 1tml.ent A;4683 1yq5.ent A 1qgj.ent A;4684 1yq5.ent A 1nas.ent A;4685 1yq5.ent A 1hvq.ent A;4686 1yq5.ent A 1fts.ent A;4687 1yq5.ent A 1dea.ent A;4688 1yq5.ent A 1b35.ent B;4689 1yq5.ent A 1a0g.ent B;4690 1yq5.ent A 3tt9.ent A;4691 1yq5.ent A 3pnl.ent B;4692 1yq5.ent A 3kvd.ent D;4693 1yq5.ent A 3fj7.ent A;4694 1yq5.ent A 3cjj.ent A;4695 1yq5.ent A 3ai9.ent X;4696 1yq5.ent A 2vg9.ent A;4697 1yq5.ent A 2p0j.ent A;4698 1yq5.ent A 2is9.ent A;4699 1yq5.ent A 2eyi.ent A;4700 1yq5.ent A 2cga.ent A;4701 1yq5.ent A 1ym0.ent A;4702 1yq5.ent A 1twf.ent E;4703 1yq5.ent A 1rc9.ent A;4704 1yq5.ent A 1pn9.ent A;4705 1yq5.ent A 1n7k.ent A;4706 1yq5.ent A 1kg5.ent A;4707 1yq5.ent A 1gis.ent A;4708 1yq5.ent A 1fuj.ent A;4709 1yq5.ent A 1del.ent A;4710 1yq5.ent A 1b77.ent A;4711 1yq5.ent A 4h6a.ent A;4712 1yq5.ent A 4a25.ent B;4713 1yq5.ent A 3q5v.ent A;4714 1yq5.ent A 3nmr.ent A;4715 1yq5.ent A 3kjz.ent A;4716 1yq5.ent A 3g5k.ent A;4717 1yq5.ent A 3csr.ent A;4718 1yq5.ent A 2zk9.ent X;4719 1yq5.ent A 2wmm.ent A;4720 1yq5.ent A 2q7a.ent A;4721 1yq5.ent A 2ill.ent A;4722 1yq5.ent A 2fko.ent A;4723 1yq5.ent A 2cut.ent A;4724 1yq5.ent A 1zv9.ent A;4725 1yq5.ent A 1xt0.ent B;4726 1yq5.ent A 1u7p.ent A;4727 1yq5.ent A 1r8n.ent A;4728 1yq5.ent A 1ocy.ent A;4729 1yq5.ent A 1m4d.ent A;4730 1yq5.ent A 1iqq.ent A;4731 1yq5.ent A 1gui.ent A;4732 1yq5.ent A 1ffk.ent T;4733 1yq5.ent A 1dow.ent A;4734 1yq5.ent A 1cau.ent A;4735 1yq5.ent A 1ava.ent C;4736 1yq5.ent A 4fei.ent A;4737 1yq5.ent A 3zqx.ent A;4738 1yq5.ent A 3sek.ent B;4739 1yq5.ent A 3obl.ent A;4740 1yq5.ent A 3mao.ent A;4741 1yq5.ent A 3kk4.ent A;4742 1yq5.ent A 3gef.ent A;4743 1yq5.ent A 3ent.ent A;4744 1yq5.ent A 3bdq.ent B;4745 1yq5.ent A 2z7j.ent A;4746 1yq5.ent A 2w80.ent A;4747 1yq5.ent A 2rcq.ent A;4748 1yq5.ent A 2p8v.ent A;4749 1yq5.ent A 2jj6.ent A;4750 1yq5.ent A 2hnx.ent A;4751 1yq5.ent A 2f06.ent A;4752 1yq5.ent A 2d00.ent A;4753 1yq5.ent A 2awg.ent A;4754 1wo8.ent A 5mdh.ent A;4755 1wo8.ent A 3two.ent B;4756 1wo8.ent A 3n9s.ent A;4757 1wo8.ent A 3exe.ent B;4758 1wo8.ent A 2yy7.ent A;4759 1wo8.ent A 2qx3.ent A;4760 1wo8.ent A 2hk2.ent A;4761 1wo8.ent A 2eih.ent A;4762 1wo8.ent A 1yg9.ent A;4763 1wo8.ent A 1tca.ent A;4764 1wo8.ent A 1pxz.ent A;4765 1wo8.ent A 1llp.ent A;4766 1wo8.ent A 1h6u.ent A;4767 1wo8.ent A 1ea7.ent A;4768 1wo8.ent A 1a4m.ent A;4769 1wo8.ent A 4b5w.ent A;4770 1wo8.ent A 3q1n.ent A;4771 1wo8.ent A 3m66.ent A;4772 1wo8.ent A 3gae.ent A;4773 1wo8.ent A 3bcz.ent A;4774 1wo8.ent A 2voa.ent A;4775 1wo8.ent A 2p9h.ent A;4776 1wo8.ent A 2gnu.ent M;4777 1wo8.ent A 2ci1.ent A;4778 1wo8.ent A 1xq6.ent A;4779 1wo8.ent A 1tml.ent A;4780 1wo8.ent A 1qgj.ent A;4781 1wo8.ent A 1nas.ent A;4782 1wo8.ent A 1hvq.ent A;4783 1wo8.ent A 1fts.ent A;4784 1wo8.ent A 1dea.ent A;4785 1wo8.ent A 1b35.ent B;4786 1wo8.ent A 1a0g.ent B;4787 1wo8.ent A 3tt9.ent A;4788 1wo8.ent A 3pnl.ent B;4789 1wo8.ent A 3kvd.ent D;4790 1wo8.ent A 3fj7.ent A;4791 1wo8.ent A 3cjj.ent A;4792 1wo8.ent A 3ai9.ent X;4793 1wo8.ent A 2vg9.ent A;4794 1wo8.ent A 2p0j.ent A;4795 1wo8.ent A 2is9.ent A;4796 1wo8.ent A 2eyi.ent A;4797 1wo8.ent A 2cga.ent A;4798 1wo8.ent A 1ym0.ent A;4799 1wo8.ent A 1twf.ent E;4800 1wo8.ent A 1rc9.ent A;4801 1wo8.ent A 1pn9.ent A;4802 1wo8.ent A 1n7k.ent A;4803 1wo8.ent A 1kg5.ent A;4804 1wo8.ent A 1gis.ent A;4805 1wo8.ent A 1fuj.ent A;4806 1wo8.ent A 1del.ent A;4807 1wo8.ent A 1b77.ent A;4808 1wo8.ent A 4h6a.ent A;4809 1wo8.ent A 4a25.ent B;4810 1wo8.ent A 3q5v.ent A;4811 1wo8.ent A 3nmr.ent A;4812 1wo8.ent A 3kjz.ent A;4813 1wo8.ent A 3g5k.ent A;4814 1wo8.ent A 3csr.ent A;4815 1wo8.ent A 2zk9.ent X;4816 1wo8.ent A 2wmm.ent A;4817 1wo8.ent A 2q7a.ent A;4818 1wo8.ent A 2ill.ent A;4819 1wo8.ent A 2fko.ent A;4820 1wo8.ent A 2cut.ent A;4821 1wo8.ent A 1zv9.ent A;4822 1wo8.ent A 1xt0.ent B;4823 1wo8.ent A 1u7p.ent A;4824 1wo8.ent A 1r8n.ent A;4825 1wo8.ent A 1ocy.ent A;4826 1wo8.ent A 1m4d.ent A;4827 1wo8.ent A 1iqq.ent A;4828 1wo8.ent A 1gui.ent A;4829 1wo8.ent A 1ffk.ent T;4830 1wo8.ent A 1dow.ent A;4831 1wo8.ent A 1cau.ent A;4832 1wo8.ent A 1ava.ent C;4833 1wo8.ent A 4fei.ent A;4834 1wo8.ent A 3zqx.ent A;4835 1wo8.ent A 3sek.ent B;4836 1wo8.ent A 3obl.ent A;4837 1wo8.ent A 3mao.ent A;4838 1wo8.ent A 3kk4.ent A;4839 1wo8.ent A 3gef.ent A;4840 1wo8.ent A 3ent.ent A;4841 1wo8.ent A 3bdq.ent B;4842 1wo8.ent A 2z7j.ent A;4843 1wo8.ent A 2w80.ent A;4844 1wo8.ent A 2rcq.ent A;4845 1wo8.ent A 2p8v.ent A;4846 1wo8.ent A 2jj6.ent A;4847 1wo8.ent A 2hnx.ent A;4848 1wo8.ent A 2f06.ent A;4849 1wo8.ent A 2d00.ent A;4850 1wo8.ent A 2awg.ent A;4851 1wo8.ent A 1yq5.ent A;4852 1uku.ent A 5mdh.ent A;4853 1uku.ent A 3two.ent B;4854 1uku.ent A 3n9s.ent A;4855 1uku.ent A 3exe.ent B;4856 1uku.ent A 2yy7.ent A;4857 1uku.ent A 2qx3.ent A;4858 1uku.ent A 2hk2.ent A;4859 1uku.ent A 2eih.ent A;4860 1uku.ent A 1yg9.ent A;4861 1uku.ent A 1tca.ent A;4862 1uku.ent A 1pxz.ent A;4863 1uku.ent A 1llp.ent A;4864 1uku.ent A 1h6u.ent A;4865 1uku.ent A 1ea7.ent A;4866 1uku.ent A 1a4m.ent A;4867 1uku.ent A 4b5w.ent A;4868 1uku.ent A 3q1n.ent A;4869 1uku.ent A 3m66.ent A;4870 1uku.ent A 3gae.ent A;4871 1uku.ent A 3bcz.ent A;4872 1uku.ent A 2voa.ent A;4873 1uku.ent A 2p9h.ent A;4874 1uku.ent A 2gnu.ent M;4875 1uku.ent A 2ci1.ent A;4876 1uku.ent A 1xq6.ent A;4877 1uku.ent A 1tml.ent A;4878 1uku.ent A 1qgj.ent A;4879 1uku.ent A 1nas.ent A;4880 1uku.ent A 1hvq.ent A;4881 1uku.ent A 1fts.ent A;4882 1uku.ent A 1dea.ent A;4883 1uku.ent A 1b35.ent B;4884 1uku.ent A 1a0g.ent B;4885 1uku.ent A 3tt9.ent A;4886 1uku.ent A 3pnl.ent B;4887 1uku.ent A 3kvd.ent D;4888 1uku.ent A 3fj7.ent A;4889 1uku.ent A 3cjj.ent A;4890 1uku.ent A 3ai9.ent X;4891 1uku.ent A 2vg9.ent A;4892 1uku.ent A 2p0j.ent A;4893 1uku.ent A 2is9.ent A;4894 1uku.ent A 2eyi.ent A;4895 1uku.ent A 2cga.ent A;4896 1uku.ent A 1ym0.ent A;4897 1uku.ent A 1twf.ent E;4898 1uku.ent A 1rc9.ent A;4899 1uku.ent A 1pn9.ent A;4900 1uku.ent A 1n7k.ent A;4901 1uku.ent A 1kg5.ent A;4902 1uku.ent A 1gis.ent A;4903 1uku.ent A 1fuj.ent A;4904 1uku.ent A 1del.ent A;4905 1uku.ent A 1b77.ent A;4906 1uku.ent A 4h6a.ent A;4907 1uku.ent A 4a25.ent B;4908 1uku.ent A 3q5v.ent A;4909 1uku.ent A 3nmr.ent A;4910 1uku.ent A 3kjz.ent A;4911 1uku.ent A 3g5k.ent A;4912 1uku.ent A 3csr.ent A;4913 1uku.ent A 2zk9.ent X;4914 1uku.ent A 2wmm.ent A;4915 1uku.ent A 2q7a.ent A;4916 1uku.ent A 2ill.ent A;4917 1uku.ent A 2fko.ent A;4918 1uku.ent A 2cut.ent A;4919 1uku.ent A 1zv9.ent A;4920 1uku.ent A 1xt0.ent B;4921 1uku.ent A 1u7p.ent A;4922 1uku.ent A 1r8n.ent A;4923 1uku.ent A 1ocy.ent A;4924 1uku.ent A 1m4d.ent A;4925 1uku.ent A 1iqq.ent A;4926 1uku.ent A 1gui.ent A;4927 1uku.ent A 1ffk.ent T;4928 1uku.ent A 1dow.ent A;4929 1uku.ent A 1cau.ent A;4930 1uku.ent A 1ava.ent C;4931 1uku.ent A 4fei.ent A;4932 1uku.ent A 3zqx.ent A;4933 1uku.ent A 3sek.ent B;4934 1uku.ent A 3obl.ent A;4935 1uku.ent A 3mao.ent A;4936 1uku.ent A 3kk4.ent A;4937 1uku.ent A 3gef.ent A;4938 1uku.ent A 3ent.ent A;4939 1uku.ent A 3bdq.ent B;4940 1uku.ent A 2z7j.ent A;4941 1uku.ent A 2w80.ent A;4942 1uku.ent A 2rcq.ent A;4943 1uku.ent A 2p8v.ent A;4944 1uku.ent A 2jj6.ent A;4945 1uku.ent A 2hnx.ent A;4946 1uku.ent A 2f06.ent A;4947 1uku.ent A 2d00.ent A;4948 1uku.ent A 2awg.ent A;4949 1uku.ent A 1yq5.ent A;4950 1uku.ent A 1wo8.ent A;4951 1spp.ent A 5mdh.ent A;4952 1spp.ent A 3two.ent B;4953 1spp.ent A 3n9s.ent A;4954 1spp.ent A 3exe.ent B;4955 1spp.ent A 2yy7.ent A;4956 1spp.ent A 2qx3.ent A;4957 1spp.ent A 2hk2.ent A;4958 1spp.ent A 2eih.ent A;4959 1spp.ent A 1yg9.ent A;4960 1spp.ent A 1tca.ent A;4961 1spp.ent A 1pxz.ent A;4962 1spp.ent A 1llp.ent A;4963 1spp.ent A 1h6u.ent A;4964 1spp.ent A 1ea7.ent A;4965 1spp.ent A 1a4m.ent A;4966 1spp.ent A 4b5w.ent A;4967 1spp.ent A 3q1n.ent A;4968 1spp.ent A 3m66.ent A;4969 1spp.ent A 3gae.ent A;4970 1spp.ent A 3bcz.ent A;4971 1spp.ent A 2voa.ent A;4972 1spp.ent A 2p9h.ent A;4973 1spp.ent A 2gnu.ent M;4974 1spp.ent A 2ci1.ent A;4975 1spp.ent A 1xq6.ent A;4976 1spp.ent A 1tml.ent A;4977 1spp.ent A 1qgj.ent A;4978 1spp.ent A 1nas.ent A;4979 1spp.ent A 1hvq.ent A;4980 1spp.ent A 1fts.ent A;4981 1spp.ent A 1dea.ent A;4982 1spp.ent A 1b35.ent B;4983 1spp.ent A 1a0g.ent B;4984 1spp.ent A 3tt9.ent A;4985 1spp.ent A 3pnl.ent B;4986 1spp.ent A 3kvd.ent D;4987 1spp.ent A 3fj7.ent A;4988 1spp.ent A 3cjj.ent A;4989 1spp.ent A 3ai9.ent X;4990 1spp.ent A 2vg9.ent A;4991 1spp.ent A 2p0j.ent A;4992 1spp.ent A 2is9.ent A;4993 1spp.ent A 2eyi.ent A;4994 1spp.ent A 2cga.ent A;4995 1spp.ent A 1ym0.ent A;4996 1spp.ent A 1twf.ent E;4997 1spp.ent A 1rc9.ent A;4998 1spp.ent A 1pn9.ent A;4999 1spp.ent A 1n7k.ent A;5000 1spp.ent A 1kg5.ent A;5001 1spp.ent A 1gis.ent A;5002 1spp.ent A 1fuj.ent A;5003 1spp.ent A 1del.ent A;5004 1spp.ent A 1b77.ent A;5005 1spp.ent A 4h6a.ent A;5006 1spp.ent A 4a25.ent B;5007 1spp.ent A 3q5v.ent A;5008 1spp.ent A 3nmr.ent A;5009 1spp.ent A 3kjz.ent A;5010 1spp.ent A 3g5k.ent A;5011 1spp.ent A 3csr.ent A;5012 1spp.ent A 2zk9.ent X;5013 1spp.ent A 2wmm.ent A;5014 1spp.ent A 2q7a.ent A;5015 1spp.ent A 2ill.ent A;5016 1spp.ent A 2fko.ent A;5017 1spp.ent A 2cut.ent A;5018 1spp.ent A 1zv9.ent A;5019 1spp.ent A 1xt0.ent B;5020 1spp.ent A 1u7p.ent A;5021 1spp.ent A 1r8n.ent A;5022 1spp.ent A 1ocy.ent A;5023 1spp.ent A 1m4d.ent A;5024 1spp.ent A 1iqq.ent A;5025 1spp.ent A 1gui.ent A;5026 1spp.ent A 1ffk.ent T;5027 1spp.ent A 1dow.ent A;5028 1spp.ent A 1cau.ent A;5029 1spp.ent A 1ava.ent C;5030 1spp.ent A 4fei.ent A;5031 1spp.ent A 3zqx.ent A;5032 1spp.ent A 3sek.ent B;5033 1spp.ent A 3obl.ent A;5034 1spp.ent A 3mao.ent A;5035 1spp.ent A 3kk4.ent A;5036 1spp.ent A 3gef.ent A;5037 1spp.ent A 3ent.ent A;5038 1spp.ent A 3bdq.ent B;5039 1spp.ent A 2z7j.ent A;5040 1spp.ent A 2w80.ent A;5041 1spp.ent A 2rcq.ent A;5042 1spp.ent A 2p8v.ent A;5043 1spp.ent A 2jj6.ent A;5044 1spp.ent A 2hnx.ent A;5045 1spp.ent A 2f06.ent A;5046 1spp.ent A 2d00.ent A;5047 1spp.ent A 2awg.ent A;5048 1spp.ent A 1yq5.ent A;5049 1spp.ent A 1wo8.ent A;5050 1spp.ent A 1uku.ent A;5051 1qj8.ent A 5mdh.ent A;5052 1qj8.ent A 3two.ent B;5053 1qj8.ent A 3n9s.ent A;5054 1qj8.ent A 3exe.ent B;5055 1qj8.ent A 2yy7.ent A;5056 1qj8.ent A 2qx3.ent A;5057 1qj8.ent A 2hk2.ent A;5058 1qj8.ent A 2eih.ent A;5059 1qj8.ent A 1yg9.ent A;5060 1qj8.ent A 1tca.ent A;5061 1qj8.ent A 1pxz.ent A;5062 1qj8.ent A 1llp.ent A;5063 1qj8.ent A 1h6u.ent A;5064 1qj8.ent A 1ea7.ent A;5065 1qj8.ent A 1a4m.ent A;5066 1qj8.ent A 4b5w.ent A;5067 1qj8.ent A 3q1n.ent A;5068 1qj8.ent A 3m66.ent A;5069 1qj8.ent A 3gae.ent A;5070 1qj8.ent A 3bcz.ent A;5071 1qj8.ent A 2voa.ent A;5072 1qj8.ent A 2p9h.ent A;5073 1qj8.ent A 2gnu.ent M;5074 1qj8.ent A 2ci1.ent A;5075 1qj8.ent A 1xq6.ent A;5076 1qj8.ent A 1tml.ent A;5077 1qj8.ent A 1qgj.ent A;5078 1qj8.ent A 1nas.ent A;5079 1qj8.ent A 1hvq.ent A;5080 1qj8.ent A 1fts.ent A;5081 1qj8.ent A 1dea.ent A;5082 1qj8.ent A 1b35.ent B;5083 1qj8.ent A 1a0g.ent B;5084 1qj8.ent A 3tt9.ent A;5085 1qj8.ent A 3pnl.ent B;5086 1qj8.ent A 3kvd.ent D;5087 1qj8.ent A 3fj7.ent A;5088 1qj8.ent A 3cjj.ent A;5089 1qj8.ent A 3ai9.ent X;5090 1qj8.ent A 2vg9.ent A;5091 1qj8.ent A 2p0j.ent A;5092 1qj8.ent A 2is9.ent A;5093 1qj8.ent A 2eyi.ent A;5094 1qj8.ent A 2cga.ent A;5095 1qj8.ent A 1ym0.ent A;5096 1qj8.ent A 1twf.ent E;5097 1qj8.ent A 1rc9.ent A;5098 1qj8.ent A 1pn9.ent A;5099 1qj8.ent A 1n7k.ent A;5100 1qj8.ent A 1kg5.ent A;5101 1qj8.ent A 1gis.ent A;5102 1qj8.ent A 1fuj.ent A;5103 1qj8.ent A 1del.ent A;5104 1qj8.ent A 1b77.ent A;5105 1qj8.ent A 4h6a.ent A;5106 1qj8.ent A 4a25.ent B;5107 1qj8.ent A 3q5v.ent A;5108 1qj8.ent A 3nmr.ent A;5109 1qj8.ent A 3kjz.ent A;5110 1qj8.ent A 3g5k.ent A;5111 1qj8.ent A 3csr.ent A;5112 1qj8.ent A 2zk9.ent X;5113 1qj8.ent A 2wmm.ent A;5114 1qj8.ent A 2q7a.ent A;5115 1qj8.ent A 2ill.ent A;5116 1qj8.ent A 2fko.ent A;5117 1qj8.ent A 2cut.ent A;5118 1qj8.ent A 1zv9.ent A;5119 1qj8.ent A 1xt0.ent B;5120 1qj8.ent A 1u7p.ent A;5121 1qj8.ent A 1r8n.ent A;5122 1qj8.ent A 1ocy.ent A;5123 1qj8.ent A 1m4d.ent A;5124 1qj8.ent A 1iqq.ent A;5125 1qj8.ent A 1gui.ent A;5126 1qj8.ent A 1ffk.ent T;5127 1qj8.ent A 1dow.ent A;5128 1qj8.ent A 1cau.ent A;5129 1qj8.ent A 1ava.ent C;5130 1qj8.ent A 4fei.ent A;5131 1qj8.ent A 3zqx.ent A;5132 1qj8.ent A 3sek.ent B;5133 1qj8.ent A 3obl.ent A;5134 1qj8.ent A 3mao.ent A;5135 1qj8.ent A 3kk4.ent A;5136 1qj8.ent A 3gef.ent A;5137 1qj8.ent A 3ent.ent A;5138 1qj8.ent A 3bdq.ent B;5139 1qj8.ent A 2z7j.ent A;5140 1qj8.ent A 2w80.ent A;5141 1qj8.ent A 2rcq.ent A;5142 1qj8.ent A 2p8v.ent A;5143 1qj8.ent A 2jj6.ent A;5144 1qj8.ent A 2hnx.ent A;5145 1qj8.ent A 2f06.ent A;5146 1qj8.ent A 2d00.ent A;5147 1qj8.ent A 2awg.ent A;5148 1qj8.ent A 1yq5.ent A;5149 1qj8.ent A 1wo8.ent A;5150 1qj8.ent A 1uku.ent A;5151 1qj8.ent A 1spp.ent A;5152 1oqw.ent A 5mdh.ent A;5153 1oqw.ent A 3two.ent B;5154 1oqw.ent A 3n9s.ent A;5155 1oqw.ent A 3exe.ent B;5156 1oqw.ent A 2yy7.ent A;5157 1oqw.ent A 2qx3.ent A;5158 1oqw.ent A 2hk2.ent A;5159 1oqw.ent A 2eih.ent A;5160 1oqw.ent A 1yg9.ent A;5161 1oqw.ent A 1tca.ent A;5162 1oqw.ent A 1pxz.ent A;5163 1oqw.ent A 1llp.ent A;5164 1oqw.ent A 1h6u.ent A;5165 1oqw.ent A 1ea7.ent A;5166 1oqw.ent A 1a4m.ent A;5167 1oqw.ent A 4b5w.ent A;5168 1oqw.ent A 3q1n.ent A;5169 1oqw.ent A 3m66.ent A;5170 1oqw.ent A 3gae.ent A;5171 1oqw.ent A 3bcz.ent A;5172 1oqw.ent A 2voa.ent A;5173 1oqw.ent A 2p9h.ent A;5174 1oqw.ent A 2gnu.ent M;5175 1oqw.ent A 2ci1.ent A;5176 1oqw.ent A 1xq6.ent A;5177 1oqw.ent A 1tml.ent A;5178 1oqw.ent A 1qgj.ent A;5179 1oqw.ent A 1nas.ent A;5180 1oqw.ent A 1hvq.ent A;5181 1oqw.ent A 1fts.ent A;5182 1oqw.ent A 1dea.ent A;5183 1oqw.ent A 1b35.ent B;5184 1oqw.ent A 1a0g.ent B;5185 1oqw.ent A 3tt9.ent A;5186 1oqw.ent A 3pnl.ent B;5187 1oqw.ent A 3kvd.ent D;5188 1oqw.ent A 3fj7.ent A;5189 1oqw.ent A 3cjj.ent A;5190 1oqw.ent A 3ai9.ent X;5191 1oqw.ent A 2vg9.ent A;5192 1oqw.ent A 2p0j.ent A;5193 1oqw.ent A 2is9.ent A;5194 1oqw.ent A 2eyi.ent A;5195 1oqw.ent A 2cga.ent A;5196 1oqw.ent A 1ym0.ent A;5197 1oqw.ent A 1twf.ent E;5198 1oqw.ent A 1rc9.ent A;5199 1oqw.ent A 1pn9.ent A;5200 1oqw.ent A 1n7k.ent A;5201 1oqw.ent A 1kg5.ent A;5202 1oqw.ent A 1gis.ent A;5203 1oqw.ent A 1fuj.ent A;5204 1oqw.ent A 1del.ent A;5205 1oqw.ent A 1b77.ent A;5206 1oqw.ent A 4h6a.ent A;5207 1oqw.ent A 4a25.ent B;5208 1oqw.ent A 3q5v.ent A;5209 1oqw.ent A 3nmr.ent A;5210 1oqw.ent A 3kjz.ent A;5211 1oqw.ent A 3g5k.ent A;5212 1oqw.ent A 3csr.ent A;5213 1oqw.ent A 2zk9.ent X;5214 1oqw.ent A 2wmm.ent A;5215 1oqw.ent A 2q7a.ent A;5216 1oqw.ent A 2ill.ent A;5217 1oqw.ent A 2fko.ent A;5218 1oqw.ent A 2cut.ent A;5219 1oqw.ent A 1zv9.ent A;5220 1oqw.ent A 1xt0.ent B;5221 1oqw.ent A 1u7p.ent A;5222 1oqw.ent A 1r8n.ent A;5223 1oqw.ent A 1ocy.ent A;5224 1oqw.ent A 1m4d.ent A;5225 1oqw.ent A 1iqq.ent A;5226 1oqw.ent A 1gui.ent A;5227 1oqw.ent A 1ffk.ent T;5228 1oqw.ent A 1dow.ent A;5229 1oqw.ent A 1cau.ent A;5230 1oqw.ent A 1ava.ent C;5231 1oqw.ent A 4fei.ent A;5232 1oqw.ent A 3zqx.ent A;5233 1oqw.ent A 3sek.ent B;5234 1oqw.ent A 3obl.ent A;5235 1oqw.ent A 3mao.ent A;5236 1oqw.ent A 3kk4.ent A;5237 1oqw.ent A 3gef.ent A;5238 1oqw.ent A 3ent.ent A;5239 1oqw.ent A 3bdq.ent B;5240 1oqw.ent A 2z7j.ent A;5241 1oqw.ent A 2w80.ent A;5242 1oqw.ent A 2rcq.ent A;5243 1oqw.ent A 2p8v.ent A;5244 1oqw.ent A 2jj6.ent A;5245 1oqw.ent A 2hnx.ent A;5246 1oqw.ent A 2f06.ent A;5247 1oqw.ent A 2d00.ent A;5248 1oqw.ent A 2awg.ent A;5249 1oqw.ent A 1yq5.ent A;5250 1oqw.ent A 1wo8.ent A;5251 1oqw.ent A 1uku.ent A;5252 1oqw.ent A 1spp.ent A;5253 1oqw.ent A 1qj8.ent A;5254 1mbu.ent A 5mdh.ent A;5255 1mbu.ent A 3two.ent B;5256 1mbu.ent A 3n9s.ent A;5257 1mbu.ent A 3exe.ent B;5258 1mbu.ent A 2yy7.ent A;5259 1mbu.ent A 2qx3.ent A;5260 1mbu.ent A 2hk2.ent A;5261 1mbu.ent A 2eih.ent A;5262 1mbu.ent A 1yg9.ent A;5263 1mbu.ent A 1tca.ent A;5264 1mbu.ent A 1pxz.ent A;5265 1mbu.ent A 1llp.ent A;5266 1mbu.ent A 1h6u.ent A;5267 1mbu.ent A 1ea7.ent A;5268 1mbu.ent A 1a4m.ent A;5269 1mbu.ent A 4b5w.ent A;5270 1mbu.ent A 3q1n.ent A;5271 1mbu.ent A 3m66.ent A;5272 1mbu.ent A 3gae.ent A;5273 1mbu.ent A 3bcz.ent A;5274 1mbu.ent A 2voa.ent A;5275 1mbu.ent A 2p9h.ent A;5276 1mbu.ent A 2gnu.ent M;5277 1mbu.ent A 2ci1.ent A;5278 1mbu.ent A 1xq6.ent A;5279 1mbu.ent A 1tml.ent A;5280 1mbu.ent A 1qgj.ent A;5281 1mbu.ent A 1nas.ent A;5282 1mbu.ent A 1hvq.ent A;5283 1mbu.ent A 1fts.ent A;5284 1mbu.ent A 1dea.ent A;5285 1mbu.ent A 1b35.ent B;5286 1mbu.ent A 1a0g.ent B;5287 1mbu.ent A 3tt9.ent A;5288 1mbu.ent A 3pnl.ent B;5289 1mbu.ent A 3kvd.ent D;5290 1mbu.ent A 3fj7.ent A;5291 1mbu.ent A 3cjj.ent A;5292 1mbu.ent A 3ai9.ent X;5293 1mbu.ent A 2vg9.ent A;5294 1mbu.ent A 2p0j.ent A;5295 1mbu.ent A 2is9.ent A;5296 1mbu.ent A 2eyi.ent A;5297 1mbu.ent A 2cga.ent A;5298 1mbu.ent A 1ym0.ent A;5299 1mbu.ent A 1twf.ent E;5300 1mbu.ent A 1rc9.ent A;5301 1mbu.ent A 1pn9.ent A;5302 1mbu.ent A 1n7k.ent A;5303 1mbu.ent A 1kg5.ent A;5304 1mbu.ent A 1gis.ent A;5305 1mbu.ent A 1fuj.ent A;5306 1mbu.ent A 1del.ent A;5307 1mbu.ent A 1b77.ent A;5308 1mbu.ent A 4h6a.ent A;5309 1mbu.ent A 4a25.ent B;5310 1mbu.ent A 3q5v.ent A;5311 1mbu.ent A 3nmr.ent A;5312 1mbu.ent A 3kjz.ent A;5313 1mbu.ent A 3g5k.ent A;5314 1mbu.ent A 3csr.ent A;5315 1mbu.ent A 2zk9.ent X;5316 1mbu.ent A 2wmm.ent A;5317 1mbu.ent A 2q7a.ent A;5318 1mbu.ent A 2ill.ent A;5319 1mbu.ent A 2fko.ent A;5320 1mbu.ent A 2cut.ent A;5321 1mbu.ent A 1zv9.ent A;5322 1mbu.ent A 1xt0.ent B;5323 1mbu.ent A 1u7p.ent A;5324 1mbu.ent A 1r8n.ent A;5325 1mbu.ent A 1ocy.ent A;5326 1mbu.ent A 1m4d.ent A;5327 1mbu.ent A 1iqq.ent A;5328 1mbu.ent A 1gui.ent A;5329 1mbu.ent A 1ffk.ent T;5330 1mbu.ent A 1dow.ent A;5331 1mbu.ent A 1cau.ent A;5332 1mbu.ent A 1ava.ent C;5333 1mbu.ent A 4fei.ent A;5334 1mbu.ent A 3zqx.ent A;5335 1mbu.ent A 3sek.ent B;5336 1mbu.ent A 3obl.ent A;5337 1mbu.ent A 3mao.ent A;5338 1mbu.ent A 3kk4.ent A;5339 1mbu.ent A 3gef.ent A;5340 1mbu.ent A 3ent.ent A;5341 1mbu.ent A 3bdq.ent B;5342 1mbu.ent A 2z7j.ent A;5343 1mbu.ent A 2w80.ent A;5344 1mbu.ent A 2rcq.ent A;5345 1mbu.ent A 2p8v.ent A;5346 1mbu.ent A 2jj6.ent A;5347 1mbu.ent A 2hnx.ent A;5348 1mbu.ent A 2f06.ent A;5349 1mbu.ent A 2d00.ent A;5350 1mbu.ent A 2awg.ent A;5351 1mbu.ent A 1yq5.ent A;5352 1mbu.ent A 1wo8.ent A;5353 1mbu.ent A 1uku.ent A;5354 1mbu.ent A 1spp.ent A;5355 1mbu.ent A 1qj8.ent A;5356 1mbu.ent A 1oqw.ent A;5357 1kpt.ent A 5mdh.ent A;5358 1kpt.ent A 3two.ent B;5359 1kpt.ent A 3n9s.ent A;5360 1kpt.ent A 3exe.ent B;5361 1kpt.ent A 2yy7.ent A;5362 1kpt.ent A 2qx3.ent A;5363 1kpt.ent A 2hk2.ent A;5364 1kpt.ent A 2eih.ent A;5365 1kpt.ent A 1yg9.ent A;5366 1kpt.ent A 1tca.ent A;5367 1kpt.ent A 1pxz.ent A;5368 1kpt.ent A 1llp.ent A;5369 1kpt.ent A 1h6u.ent A;5370 1kpt.ent A 1ea7.ent A;5371 1kpt.ent A 1a4m.ent A;5372 1kpt.ent A 4b5w.ent A;5373 1kpt.ent A 3q1n.ent A;5374 1kpt.ent A 3m66.ent A;5375 1kpt.ent A 3gae.ent A;5376 1kpt.ent A 3bcz.ent A;5377 1kpt.ent A 2voa.ent A;5378 1kpt.ent A 2p9h.ent A;5379 1kpt.ent A 2gnu.ent M;5380 1kpt.ent A 2ci1.ent A;5381 1kpt.ent A 1xq6.ent A;5382 1kpt.ent A 1tml.ent A;5383 1kpt.ent A 1qgj.ent A;5384 1kpt.ent A 1nas.ent A;5385 1kpt.ent A 1hvq.ent A;5386 1kpt.ent A 1fts.ent A;5387 1kpt.ent A 1dea.ent A;5388 1kpt.ent A 1b35.ent B;5389 1kpt.ent A 1a0g.ent B;5390 1kpt.ent A 3tt9.ent A;5391 1kpt.ent A 3pnl.ent B;5392 1kpt.ent A 3kvd.ent D;5393 1kpt.ent A 3fj7.ent A;5394 1kpt.ent A 3cjj.ent A;5395 1kpt.ent A 3ai9.ent X;5396 1kpt.ent A 2vg9.ent A;5397 1kpt.ent A 2p0j.ent A;5398 1kpt.ent A 2is9.ent A;5399 1kpt.ent A 2eyi.ent A;5400 1kpt.ent A 2cga.ent A;5401 1kpt.ent A 1ym0.ent A;5402 1kpt.ent A 1twf.ent E;5403 1kpt.ent A 1rc9.ent A;5404 1kpt.ent A 1pn9.ent A;5405 1kpt.ent A 1n7k.ent A;5406 1kpt.ent A 1kg5.ent A;5407 1kpt.ent A 1gis.ent A;5408 1kpt.ent A 1fuj.ent A;5409 1kpt.ent A 1del.ent A;5410 1kpt.ent A 1b77.ent A;5411 1kpt.ent A 4h6a.ent A;5412 1kpt.ent A 4a25.ent B;5413 1kpt.ent A 3q5v.ent A;5414 1kpt.ent A 3nmr.ent A;5415 1kpt.ent A 3kjz.ent A;5416 1kpt.ent A 3g5k.ent A;5417 1kpt.ent A 3csr.ent A;5418 1kpt.ent A 2zk9.ent X;5419 1kpt.ent A 2wmm.ent A;5420 1kpt.ent A 2q7a.ent A;5421 1kpt.ent A 2ill.ent A;5422 1kpt.ent A 2fko.ent A;5423 1kpt.ent A 2cut.ent A;5424 1kpt.ent A 1zv9.ent A;5425 1kpt.ent A 1xt0.ent B;5426 1kpt.ent A 1u7p.ent A;5427 1kpt.ent A 1r8n.ent A;5428 1kpt.ent A 1ocy.ent A;5429 1kpt.ent A 1m4d.ent A;5430 1kpt.ent A 1iqq.ent A;5431 1kpt.ent A 1gui.ent A;5432 1kpt.ent A 1ffk.ent T;5433 1kpt.ent A 1dow.ent A;5434 1kpt.ent A 1cau.ent A;5435 1kpt.ent A 1ava.ent C;5436 1kpt.ent A 4fei.ent A;5437 1kpt.ent A 3zqx.ent A;5438 1kpt.ent A 3sek.ent B;5439 1kpt.ent A 3obl.ent A;5440 1kpt.ent A 3mao.ent A;5441 1kpt.ent A 3kk4.ent A;5442 1kpt.ent A 3gef.ent A;5443 1kpt.ent A 3ent.ent A;5444 1kpt.ent A 3bdq.ent B;5445 1kpt.ent A 2z7j.ent A;5446 1kpt.ent A 2w80.ent A;5447 1kpt.ent A 2rcq.ent A;5448 1kpt.ent A 2p8v.ent A;5449 1kpt.ent A 2jj6.ent A;5450 1kpt.ent A 2hnx.ent A;5451 1kpt.ent A 2f06.ent A;5452 1kpt.ent A 2d00.ent A;5453 1kpt.ent A 2awg.ent A;5454 1kpt.ent A 1yq5.ent A;5455 1kpt.ent A 1wo8.ent A;5456 1kpt.ent A 1uku.ent A;5457 1kpt.ent A 1spp.ent A;5458 1kpt.ent A 1qj8.ent A;5459 1kpt.ent A 1oqw.ent A;5460 1kpt.ent A 1mbu.ent A;5461 1iu9.ent A 5mdh.ent A;5462 1iu9.ent A 3two.ent B;5463 1iu9.ent A 3n9s.ent A;5464 1iu9.ent A 3exe.ent B;5465 1iu9.ent A 2yy7.ent A;5466 1iu9.ent A 2qx3.ent A;5467 1iu9.ent A 2hk2.ent A;5468 1iu9.ent A 2eih.ent A;5469 1iu9.ent A 1yg9.ent A;5470 1iu9.ent A 1tca.ent A;5471 1iu9.ent A 1pxz.ent A;5472 1iu9.ent A 1llp.ent A;5473 1iu9.ent A 1h6u.ent A;5474 1iu9.ent A 1ea7.ent A;5475 1iu9.ent A 1a4m.ent A;5476 1iu9.ent A 4b5w.ent A;5477 1iu9.ent A 3q1n.ent A;5478 1iu9.ent A 3m66.ent A;5479 1iu9.ent A 3gae.ent A;5480 1iu9.ent A 3bcz.ent A;5481 1iu9.ent A 2voa.ent A;5482 1iu9.ent A 2p9h.ent A;5483 1iu9.ent A 2gnu.ent M;5484 1iu9.ent A 2ci1.ent A;5485 1iu9.ent A 1xq6.ent A;5486 1iu9.ent A 1tml.ent A;5487 1iu9.ent A 1qgj.ent A;5488 1iu9.ent A 1nas.ent A;5489 1iu9.ent A 1hvq.ent A;5490 1iu9.ent A 1fts.ent A;5491 1iu9.ent A 1dea.ent A;5492 1iu9.ent A 1b35.ent B;5493 1iu9.ent A 1a0g.ent B;5494 1iu9.ent A 3tt9.ent A;5495 1iu9.ent A 3pnl.ent B;5496 1iu9.ent A 3kvd.ent D;5497 1iu9.ent A 3fj7.ent A;5498 1iu9.ent A 3cjj.ent A;5499 1iu9.ent A 3ai9.ent X;5500 1iu9.ent A 2vg9.ent A;5501 1iu9.ent A 2p0j.ent A;5502 1iu9.ent A 2is9.ent A;5503 1iu9.ent A 2eyi.ent A;5504 1iu9.ent A 2cga.ent A;5505 1iu9.ent A 1ym0.ent A;5506 1iu9.ent A 1twf.ent E;5507 1iu9.ent A 1rc9.ent A;5508 1iu9.ent A 1pn9.ent A;5509 1iu9.ent A 1n7k.ent A;5510 1iu9.ent A 1kg5.ent A;5511 1iu9.ent A 1gis.ent A;5512 1iu9.ent A 1fuj.ent A;5513 1iu9.ent A 1del.ent A;5514 1iu9.ent A 1b77.ent A;5515 1iu9.ent A 4h6a.ent A;5516 1iu9.ent A 4a25.ent B;5517 1iu9.ent A 3q5v.ent A;5518 1iu9.ent A 3nmr.ent A;5519 1iu9.ent A 3kjz.ent A;5520 1iu9.ent A 3g5k.ent A;5521 1iu9.ent A 3csr.ent A;5522 1iu9.ent A 2zk9.ent X;5523 1iu9.ent A 2wmm.ent A;5524 1iu9.ent A 2q7a.ent A;5525 1iu9.ent A 2ill.ent A;5526 1iu9.ent A 2fko.ent A;5527 1iu9.ent A 2cut.ent A;5528 1iu9.ent A 1zv9.ent A;5529 1iu9.ent A 1xt0.ent B;5530 1iu9.ent A 1u7p.ent A;5531 1iu9.ent A 1r8n.ent A;5532 1iu9.ent A 1ocy.ent A;5533 1iu9.ent A 1m4d.ent A;5534 1iu9.ent A 1iqq.ent A;5535 1iu9.ent A 1gui.ent A;5536 1iu9.ent A 1ffk.ent T;5537 1iu9.ent A 1dow.ent A;5538 1iu9.ent A 1cau.ent A;5539 1iu9.ent A 1ava.ent C;5540 1iu9.ent A 4fei.ent A;5541 1iu9.ent A 3zqx.ent A;5542 1iu9.ent A 3sek.ent B;5543 1iu9.ent A 3obl.ent A;5544 1iu9.ent A 3mao.ent A;5545 1iu9.ent A 3kk4.ent A;5546 1iu9.ent A 3gef.ent A;5547 1iu9.ent A 3ent.ent A;5548 1iu9.ent A 3bdq.ent B;5549 1iu9.ent A 2z7j.ent A;5550 1iu9.ent A 2w80.ent A;5551 1iu9.ent A 2rcq.ent A;5552 1iu9.ent A 2p8v.ent A;5553 1iu9.ent A 2jj6.ent A;5554 1iu9.ent A 2hnx.ent A;5555 1iu9.ent A 2f06.ent A;5556 1iu9.ent A 2d00.ent A;5557 1iu9.ent A 2awg.ent A;5558 1iu9.ent A 1yq5.ent A;5559 1iu9.ent A 1wo8.ent A;5560 1iu9.ent A 1uku.ent A;5561 1iu9.ent A 1spp.ent A;5562 1iu9.ent A 1qj8.ent A;5563 1iu9.ent A 1oqw.ent A;5564 1iu9.ent A 1mbu.ent A;5565 1iu9.ent A 1kpt.ent A;5566 1gyw.ent B 5mdh.ent A;5567 1gyw.ent B 3two.ent B;5568 1gyw.ent B 3n9s.ent A;5569 1gyw.ent B 3exe.ent B;5570 1gyw.ent B 2yy7.ent A;5571 1gyw.ent B 2qx3.ent A;5572 1gyw.ent B 2hk2.ent A;5573 1gyw.ent B 2eih.ent A;5574 1gyw.ent B 1yg9.ent A;5575 1gyw.ent B 1tca.ent A;5576 1gyw.ent B 1pxz.ent A;5577 1gyw.ent B 1llp.ent A;5578 1gyw.ent B 1h6u.ent A;5579 1gyw.ent B 1ea7.ent A;5580 1gyw.ent B 1a4m.ent A;5581 1gyw.ent B 4b5w.ent A;5582 1gyw.ent B 3q1n.ent A;5583 1gyw.ent B 3m66.ent A;5584 1gyw.ent B 3gae.ent A;5585 1gyw.ent B 3bcz.ent A;5586 1gyw.ent B 2voa.ent A;5587 1gyw.ent B 2p9h.ent A;5588 1gyw.ent B 2gnu.ent M;5589 1gyw.ent B 2ci1.ent A;5590 1gyw.ent B 1xq6.ent A;5591 1gyw.ent B 1tml.ent A;5592 1gyw.ent B 1qgj.ent A;5593 1gyw.ent B 1nas.ent A;5594 1gyw.ent B 1hvq.ent A;5595 1gyw.ent B 1fts.ent A;5596 1gyw.ent B 1dea.ent A;5597 1gyw.ent B 1b35.ent B;5598 1gyw.ent B 1a0g.ent B;5599 1gyw.ent B 3tt9.ent A;5600 1gyw.ent B 3pnl.ent B;5601 1gyw.ent B 3kvd.ent D;5602 1gyw.ent B 3fj7.ent A;5603 1gyw.ent B 3cjj.ent A;5604 1gyw.ent B 3ai9.ent X;5605 1gyw.ent B 2vg9.ent A;5606 1gyw.ent B 2p0j.ent A;5607 1gyw.ent B 2is9.ent A;5608 1gyw.ent B 2eyi.ent A;5609 1gyw.ent B 2cga.ent A;5610 1gyw.ent B 1ym0.ent A;5611 1gyw.ent B 1twf.ent E;5612 1gyw.ent B 1rc9.ent A;5613 1gyw.ent B 1pn9.ent A;5614 1gyw.ent B 1n7k.ent A;5615 1gyw.ent B 1kg5.ent A;5616 1gyw.ent B 1gis.ent A;5617 1gyw.ent B 1fuj.ent A;5618 1gyw.ent B 1del.ent A;5619 1gyw.ent B 1b77.ent A;5620 1gyw.ent B 4h6a.ent A;5621 1gyw.ent B 4a25.ent B;5622 1gyw.ent B 3q5v.ent A;5623 1gyw.ent B 3nmr.ent A;5624 1gyw.ent B 3kjz.ent A;5625 1gyw.ent B 3g5k.ent A;5626 1gyw.ent B 3csr.ent A;5627 1gyw.ent B 2zk9.ent X;5628 1gyw.ent B 2wmm.ent A;5629 1gyw.ent B 2q7a.ent A;5630 1gyw.ent B 2ill.ent A;5631 1gyw.ent B 2fko.ent A;5632 1gyw.ent B 2cut.ent A;5633 1gyw.ent B 1zv9.ent A;5634 1gyw.ent B 1xt0.ent B;5635 1gyw.ent B 1u7p.ent A;5636 1gyw.ent B 1r8n.ent A;5637 1gyw.ent B 1ocy.ent A;5638 1gyw.ent B 1m4d.ent A;5639 1gyw.ent B 1iqq.ent A;5640 1gyw.ent B 1gui.ent A;5641 1gyw.ent B 1ffk.ent T;5642 1gyw.ent B 1dow.ent A;5643 1gyw.ent B 1cau.ent A;5644 1gyw.ent B 1ava.ent C;5645 1gyw.ent B 4fei.ent A;5646 1gyw.ent B 3zqx.ent A;5647 1gyw.ent B 3sek.ent B;5648 1gyw.ent B 3obl.ent A;5649 1gyw.ent B 3mao.ent A;5650 1gyw.ent B 3kk4.ent A;5651 1gyw.ent B 3gef.ent A;5652 1gyw.ent B 3ent.ent A;5653 1gyw.ent B 3bdq.ent B;5654 1gyw.ent B 2z7j.ent A;5655 1gyw.ent B 2w80.ent A;5656 1gyw.ent B 2rcq.ent A;5657 1gyw.ent B 2p8v.ent A;5658 1gyw.ent B 2jj6.ent A;5659 1gyw.ent B 2hnx.ent A;5660 1gyw.ent B 2f06.ent A;5661 1gyw.ent B 2d00.ent A;5662 1gyw.ent B 2awg.ent A;5663 1gyw.ent B 1yq5.ent A;5664 1gyw.ent B 1wo8.ent A;5665 1gyw.ent B 1uku.ent A;5666 1gyw.ent B 1spp.ent A;5667 1gyw.ent B 1qj8.ent A;5668 1gyw.ent B 1oqw.ent A;5669 1gyw.ent B 1mbu.ent A;5670 1gyw.ent B 1kpt.ent A;5671 1gyw.ent B 1iu9.ent A;5672 1fla.ent A 5mdh.ent A;5673 1fla.ent A 3two.ent B;5674 1fla.ent A 3n9s.ent A;5675 1fla.ent A 3exe.ent B;5676 1fla.ent A 2yy7.ent A;5677 1fla.ent A 2qx3.ent A;5678 1fla.ent A 2hk2.ent A;5679 1fla.ent A 2eih.ent A;5680 1fla.ent A 1yg9.ent A;5681 1fla.ent A 1tca.ent A;5682 1fla.ent A 1pxz.ent A;5683 1fla.ent A 1llp.ent A;5684 1fla.ent A 1h6u.ent A;5685 1fla.ent A 1ea7.ent A;5686 1fla.ent A 1a4m.ent A;5687 1fla.ent A 4b5w.ent A;5688 1fla.ent A 3q1n.ent A;5689 1fla.ent A 3m66.ent A;5690 1fla.ent A 3gae.ent A;5691 1fla.ent A 3bcz.ent A;5692 1fla.ent A 2voa.ent A;5693 1fla.ent A 2p9h.ent A;5694 1fla.ent A 2gnu.ent M;5695 1fla.ent A 2ci1.ent A;5696 1fla.ent A 1xq6.ent A;5697 1fla.ent A 1tml.ent A;5698 1fla.ent A 1qgj.ent A;5699 1fla.ent A 1nas.ent A;5700 1fla.ent A 1hvq.ent A;5701 1fla.ent A 1fts.ent A;5702 1fla.ent A 1dea.ent A;5703 1fla.ent A 1b35.ent B;5704 1fla.ent A 1a0g.ent B;5705 1fla.ent A 3tt9.ent A;5706 1fla.ent A 3pnl.ent B;5707 1fla.ent A 3kvd.ent D;5708 1fla.ent A 3fj7.ent A;5709 1fla.ent A 3cjj.ent A;5710 1fla.ent A 3ai9.ent X;5711 1fla.ent A 2vg9.ent A;5712 1fla.ent A 2p0j.ent A;5713 1fla.ent A 2is9.ent A;5714 1fla.ent A 2eyi.ent A;5715 1fla.ent A 2cga.ent A;5716 1fla.ent A 1ym0.ent A;5717 1fla.ent A 1twf.ent E;5718 1fla.ent A 1rc9.ent A;5719 1fla.ent A 1pn9.ent A;5720 1fla.ent A 1n7k.ent A;5721 1fla.ent A 1kg5.ent A;5722 1fla.ent A 1gis.ent A;5723 1fla.ent A 1fuj.ent A;5724 1fla.ent A 1del.ent A;5725 1fla.ent A 1b77.ent A;5726 1fla.ent A 4h6a.ent A;5727 1fla.ent A 4a25.ent B;5728 1fla.ent A 3q5v.ent A;5729 1fla.ent A 3nmr.ent A;5730 1fla.ent A 3kjz.ent A;5731 1fla.ent A 3g5k.ent A;5732 1fla.ent A 3csr.ent A;5733 1fla.ent A 2zk9.ent X;5734 1fla.ent A 2wmm.ent A;5735 1fla.ent A 2q7a.ent A;5736 1fla.ent A 2ill.ent A;5737 1fla.ent A 2fko.ent A;5738 1fla.ent A 2cut.ent A;5739 1fla.ent A 1zv9.ent A;5740 1fla.ent A 1xt0.ent B;5741 1fla.ent A 1u7p.ent A;5742 1fla.ent A 1r8n.ent A;5743 1fla.ent A 1ocy.ent A;5744 1fla.ent A 1m4d.ent A;5745 1fla.ent A 1iqq.ent A;5746 1fla.ent A 1gui.ent A;5747 1fla.ent A 1ffk.ent T;5748 1fla.ent A 1dow.ent A;5749 1fla.ent A 1cau.ent A;5750 1fla.ent A 1ava.ent C;5751 1fla.ent A 4fei.ent A;5752 1fla.ent A 3zqx.ent A;5753 1fla.ent A 3sek.ent B;5754 1fla.ent A 3obl.ent A;5755 1fla.ent A 3mao.ent A;5756 1fla.ent A 3kk4.ent A;5757 1fla.ent A 3gef.ent A;5758 1fla.ent A 3ent.ent A;5759 1fla.ent A 3bdq.ent B;5760 1fla.ent A 2z7j.ent A;5761 1fla.ent A 2w80.ent A;5762 1fla.ent A 2rcq.ent A;5763 1fla.ent A 2p8v.ent A;5764 1fla.ent A 2jj6.ent A;5765 1fla.ent A 2hnx.ent A;5766 1fla.ent A 2f06.ent A;5767 1fla.ent A 2d00.ent A;5768 1fla.ent A 2awg.ent A;5769 1fla.ent A 1yq5.ent A;5770 1fla.ent A 1wo8.ent A;5771 1fla.ent A 1uku.ent A;5772 1fla.ent A 1spp.ent A;5773 1fla.ent A 1qj8.ent A;5774 1fla.ent A 1oqw.ent A;5775 1fla.ent A 1mbu.ent A;5776 1fla.ent A 1kpt.ent A;5777 1fla.ent A 1iu9.ent A;5778 1fla.ent A 1gyw.ent B;5779 1edy.ent A 5mdh.ent A;5780 1edy.ent A 3two.ent B;5781 1edy.ent A 3n9s.ent A;5782 1edy.ent A 3exe.ent B;5783 1edy.ent A 2yy7.ent A;5784 1edy.ent A 2qx3.ent A;5785 1edy.ent A 2hk2.ent A;5786 1edy.ent A 2eih.ent A;5787 1edy.ent A 1yg9.ent A;5788 1edy.ent A 1tca.ent A;5789 1edy.ent A 1pxz.ent A;5790 1edy.ent A 1llp.ent A;5791 1edy.ent A 1h6u.ent A;5792 1edy.ent A 1ea7.ent A;5793 1edy.ent A 1a4m.ent A;5794 1edy.ent A 4b5w.ent A;5795 1edy.ent A 3q1n.ent A;5796 1edy.ent A 3m66.ent A;5797 1edy.ent A 3gae.ent A;5798 1edy.ent A 3bcz.ent A;5799 1edy.ent A 2voa.ent A;5800 1edy.ent A 2p9h.ent A;5801 1edy.ent A 2gnu.ent M;5802 1edy.ent A 2ci1.ent A;5803 1edy.ent A 1xq6.ent A;5804 1edy.ent A 1tml.ent A;5805 1edy.ent A 1qgj.ent A;5806 1edy.ent A 1nas.ent A;5807 1edy.ent A 1hvq.ent A;5808 1edy.ent A 1fts.ent A;5809 1edy.ent A 1dea.ent A;5810 1edy.ent A 1b35.ent B;5811 1edy.ent A 1a0g.ent B;5812 1edy.ent A 3tt9.ent A;5813 1edy.ent A 3pnl.ent B;5814 1edy.ent A 3kvd.ent D;5815 1edy.ent A 3fj7.ent A;5816 1edy.ent A 3cjj.ent A;5817 1edy.ent A 3ai9.ent X;5818 1edy.ent A 2vg9.ent A;5819 1edy.ent A 2p0j.ent A;5820 1edy.ent A 2is9.ent A;5821 1edy.ent A 2eyi.ent A;5822 1edy.ent A 2cga.ent A;5823 1edy.ent A 1ym0.ent A;5824 1edy.ent A 1twf.ent E;5825 1edy.ent A 1rc9.ent A;5826 1edy.ent A 1pn9.ent A;5827 1edy.ent A 1n7k.ent A;5828 1edy.ent A 1kg5.ent A;5829 1edy.ent A 1gis.ent A;5830 1edy.ent A 1fuj.ent A;5831 1edy.ent A 1del.ent A;5832 1edy.ent A 1b77.ent A;5833 1edy.ent A 4h6a.ent A;5834 1edy.ent A 4a25.ent B;5835 1edy.ent A 3q5v.ent A;5836 1edy.ent A 3nmr.ent A;5837 1edy.ent A 3kjz.ent A;5838 1edy.ent A 3g5k.ent A;5839 1edy.ent A 3csr.ent A;5840 1edy.ent A 2zk9.ent X;5841 1edy.ent A 2wmm.ent A;5842 1edy.ent A 2q7a.ent A;5843 1edy.ent A 2ill.ent A;5844 1edy.ent A 2fko.ent A;5845 1edy.ent A 2cut.ent A;5846 1edy.ent A 1zv9.ent A;5847 1edy.ent A 1xt0.ent B;5848 1edy.ent A 1u7p.ent A;5849 1edy.ent A 1r8n.ent A;5850 1edy.ent A 1ocy.ent A;5851 1edy.ent A 1m4d.ent A;5852 1edy.ent A 1iqq.ent A;5853 1edy.ent A 1gui.ent A;5854 1edy.ent A 1ffk.ent T;5855 1edy.ent A 1dow.ent A;5856 1edy.ent A 1cau.ent A;5857 1edy.ent A 1ava.ent C;5858 1edy.ent A 4fei.ent A;5859 1edy.ent A 3zqx.ent A;5860 1edy.ent A 3sek.ent B;5861 1edy.ent A 3obl.ent A;5862 1edy.ent A 3mao.ent A;5863 1edy.ent A 3kk4.ent A;5864 1edy.ent A 3gef.ent A;5865 1edy.ent A 3ent.ent A;5866 1edy.ent A 3bdq.ent B;5867 1edy.ent A 2z7j.ent A;5868 1edy.ent A 2w80.ent A;5869 1edy.ent A 2rcq.ent A;5870 1edy.ent A 2p8v.ent A;5871 1edy.ent A 2jj6.ent A;5872 1edy.ent A 2hnx.ent A;5873 1edy.ent A 2f06.ent A;5874 1edy.ent A 2d00.ent A;5875 1edy.ent A 2awg.ent A;5876 1edy.ent A 1yq5.ent A;5877 1edy.ent A 1wo8.ent A;5878 1edy.ent A 1uku.ent A;5879 1edy.ent A 1spp.ent A;5880 1edy.ent A 1qj8.ent A;5881 1edy.ent A 1oqw.ent A;5882 1edy.ent A 1mbu.ent A;5883 1edy.ent A 1kpt.ent A;5884 1edy.ent A 1iu9.ent A;5885 1edy.ent A 1gyw.ent B;5886 1edy.ent A 1fla.ent A;5887 1dbf.ent A 5mdh.ent A;5888 1dbf.ent A 3two.ent B;5889 1dbf.ent A 3n9s.ent A;5890 1dbf.ent A 3exe.ent B;5891 1dbf.ent A 2yy7.ent A;5892 1dbf.ent A 2qx3.ent A;5893 1dbf.ent A 2hk2.ent A;5894 1dbf.ent A 2eih.ent A;5895 1dbf.ent A 1yg9.ent A;5896 1dbf.ent A 1tca.ent A;5897 1dbf.ent A 1pxz.ent A;5898 1dbf.ent A 1llp.ent A;5899 1dbf.ent A 1h6u.ent A;5900 1dbf.ent A 1ea7.ent A;5901 1dbf.ent A 1a4m.ent A;5902 1dbf.ent A 4b5w.ent A;5903 1dbf.ent A 3q1n.ent A;5904 1dbf.ent A 3m66.ent A;5905 1dbf.ent A 3gae.ent A;5906 1dbf.ent A 3bcz.ent A;5907 1dbf.ent A 2voa.ent A;5908 1dbf.ent A 2p9h.ent A;5909 1dbf.ent A 2gnu.ent M;5910 1dbf.ent A 2ci1.ent A;5911 1dbf.ent A 1xq6.ent A;5912 1dbf.ent A 1tml.ent A;5913 1dbf.ent A 1qgj.ent A;5914 1dbf.ent A 1nas.ent A;5915 1dbf.ent A 1hvq.ent A;5916 1dbf.ent A 1fts.ent A;5917 1dbf.ent A 1dea.ent A;5918 1dbf.ent A 1b35.ent B;5919 1dbf.ent A 1a0g.ent B;5920 1dbf.ent A 3tt9.ent A;5921 1dbf.ent A 3pnl.ent B;5922 1dbf.ent A 3kvd.ent D;5923 1dbf.ent A 3fj7.ent A;5924 1dbf.ent A 3cjj.ent A;5925 1dbf.ent A 3ai9.ent X;5926 1dbf.ent A 2vg9.ent A;5927 1dbf.ent A 2p0j.ent A;5928 1dbf.ent A 2is9.ent A;5929 1dbf.ent A 2eyi.ent A;5930 1dbf.ent A 2cga.ent A;5931 1dbf.ent A 1ym0.ent A;5932 1dbf.ent A 1twf.ent E;5933 1dbf.ent A 1rc9.ent A;5934 1dbf.ent A 1pn9.ent A;5935 1dbf.ent A 1n7k.ent A;5936 1dbf.ent A 1kg5.ent A;5937 1dbf.ent A 1gis.ent A;5938 1dbf.ent A 1fuj.ent A;5939 1dbf.ent A 1del.ent A;5940 1dbf.ent A 1b77.ent A;5941 1dbf.ent A 4h6a.ent A;5942 1dbf.ent A 4a25.ent B;5943 1dbf.ent A 3q5v.ent A;5944 1dbf.ent A 3nmr.ent A;5945 1dbf.ent A 3kjz.ent A;5946 1dbf.ent A 3g5k.ent A;5947 1dbf.ent A 3csr.ent A;5948 1dbf.ent A 2zk9.ent X;5949 1dbf.ent A 2wmm.ent A;5950 1dbf.ent A 2q7a.ent A;5951 1dbf.ent A 2ill.ent A;5952 1dbf.ent A 2fko.ent A;5953 1dbf.ent A 2cut.ent A;5954 1dbf.ent A 1zv9.ent A;5955 1dbf.ent A 1xt0.ent B;5956 1dbf.ent A 1u7p.ent A;5957 1dbf.ent A 1r8n.ent A;5958 1dbf.ent A 1ocy.ent A;5959 1dbf.ent A 1m4d.ent A;5960 1dbf.ent A 1iqq.ent A;5961 1dbf.ent A 1gui.ent A;5962 1dbf.ent A 1ffk.ent T;5963 1dbf.ent A 1dow.ent A;5964 1dbf.ent A 1cau.ent A;5965 1dbf.ent A 1ava.ent C;5966 1dbf.ent A 4fei.ent A;5967 1dbf.ent A 3zqx.ent A;5968 1dbf.ent A 3sek.ent B;5969 1dbf.ent A 3obl.ent A;5970 1dbf.ent A 3mao.ent A;5971 1dbf.ent A 3kk4.ent A;5972 1dbf.ent A 3gef.ent A;5973 1dbf.ent A 3ent.ent A;5974 1dbf.ent A 3bdq.ent B;5975 1dbf.ent A 2z7j.ent A;5976 1dbf.ent A 2w80.ent A;5977 1dbf.ent A 2rcq.ent A;5978 1dbf.ent A 2p8v.ent A;5979 1dbf.ent A 2jj6.ent A;5980 1dbf.ent A 2hnx.ent A;5981 1dbf.ent A 2f06.ent A;5982 1dbf.ent A 2d00.ent A;5983 1dbf.ent A 2awg.ent A;5984 1dbf.ent A 1yq5.ent A;5985 1dbf.ent A 1wo8.ent A;5986 1dbf.ent A 1uku.ent A;5987 1dbf.ent A 1spp.ent A;5988 1dbf.ent A 1qj8.ent A;5989 1dbf.ent A 1oqw.ent A;5990 1dbf.ent A 1mbu.ent A;5991 1dbf.ent A 1kpt.ent A;5992 1dbf.ent A 1iu9.ent A;5993 1dbf.ent A 1gyw.ent B;5994 1dbf.ent A 1fla.ent A;5995 1dbf.ent A 1edy.ent A;5996 1c08.ent B 5mdh.ent A;5997 1c08.ent B 3two.ent B;5998 1c08.ent B 3n9s.ent A;5999 1c08.ent B 3exe.ent B;6000 1c08.ent B 2yy7.ent A;
